# Development of a sequential workflow based on LC-PRM for the verification of endometrial cancer protein biomarkers in uterine aspirate samples

**DOI:** 10.18632/oncotarget.10632

**Published:** 2016-07-16

**Authors:** Elena Martinez-Garcia, Antoine Lesur, Laura Devis, Alexandre Campos, Silvia Cabrera, Jan van Oostrum, Xavier Matias-Guiu, Antonio Gil-Moreno, Jaume Reventos, Eva Colas, Bruno Domon

**Affiliations:** ^1^ Biomedical Research Group in Gynecology, Vall Hebron Research Institute (VHIR), Universitat Autònoma de Barcelona, Barcelona, Spain; ^2^ Luxembourg Clinical Proteomics Center (LCP), Luxembourg Institute of Health (LIH), Strassen, Luxembourg; ^3^ Sanford-Burnham Medical Research Institute, La Jolla, California, USA; ^4^ Gynecological Oncology Department, Vall Hebron University Hospital, Barcelona, Spain; ^5^ Pathological Oncology Group and Pathology Department, Hospital Arnau de Vilanova, Lleida, Spain; ^6^ Basic Sciences Department, International University of Catalonia, Barcelona, Spain

**Keywords:** uterine aspirate, endometrial cancer, biomarker verification, high resolution accurate mass spectrometry, parallel reaction monitoring

## Abstract

About 30% of endometrial cancer (EC) patients are diagnosed at an advanced stage of the disease, which is associated with a drastic decrease in the 5-year survival rate. The identification of biomarkers in uterine aspirate samples, which are collected by a minimally invasive procedure, would improve early diagnosis of EC. We present a sequential workflow to select from a list of potential EC biomarkers, those which are the most promising to enter a validation study. After the elimination of confounding contributions by residual blood proteins, 52 potential biomarkers were analyzed in uterine aspirates from 20 EC patients and 18 non-EC controls by a high-resolution accurate mass spectrometer operated in parallel reaction monitoring mode. The differential abundance of 26 biomarkers was observed, and among them ten proteins showed a high sensitivity and specificity (AUC > 0.9). The study demonstrates that uterine aspirates are valuable samples for EC protein biomarkers screening. It also illustrates the importance of a biomarker verification phase to fill the gap between discovery and validation studies and highlights the benefits of high resolution mass spectrometry for this purpose. The proteins verified in this study have an increased likelihood to become a clinical assay after a subsequent validation phase.

## INTRODUCTION

Endometrial cancer (EC) is the most frequently observed invasive tumor of the female genital tract and the fourth most common cancer in women in developed countries, accounting for 60,050 diagnosed cases and 10,470 estimated deaths in 2016 in the United States [1]. Postmenopausal women represent 86% of diagnosed EC cases, with a median age of 63 years [2]. Nowadays, 70% of the EC cases are diagnosed at early stages of the disease where the tumor is still localized within the endometrium and is associated with an overall 5-year survival rate of 96%. However, 30% of EC patients are diagnosed at an advanced stage of the disease associated with a drastic decrease in the 5-year survival rate, which is reduced to 68% when myometrial invasion and/or lymph node affectation is already present, and to 17% in cases of distant metastasis [1]. Improving early diagnosis is hence a major issue to appropriately manage EC and decrease mortality associated with the disease.

Early detection of EC patients is favored by the presence of symptoms like abnormal vaginal bleeding, present in 93% of women diagnosed with EC. However, many other benign disorders generate similar symptoms [3]. Discrimination of patients with benign endometrial pathologies and with EC is only achieved after a tedious diagnostic process consisting of a pelvic examination and a transvaginal ultrasonography followed by a confirmatory histopathological examination of an endometrial biopsy. The preferable biopsy used in this procedure is named uterine aspirate or pipelle biopsy and is obtained by a minimally invasive aspiration of endometrial fluid from inside the uterine cavity using a Cornier pipelle. Using this sampling, this process has unfortunately a diagnostic failure and an inadequate sampling rate of 8% and 15%, respectively; which is increased in postmenopausal women up to 12% and 22% [4]. In those undiagnosed cases, a biopsy guided by hysteroscopy needs to be performed, but this invasive technique presents more complications, including uterine perforation, hemorrhage and possible damage to other organs [5]. Implementation of biomarkers in early stages of the diagnostic process would improve detection of EC.

From a biological point of view, proteins are key players of many cellular processes and variations of their abundance can be associated with pathologies such as cancer. Proteins are detectable in biofluids and thus are valuable disease indicators for the development of non-invasive diagnostic tests. In this regard, uterine aspirates are specially promising as a source of EC biomarkers thanks to the direct contact of this fluid with the endometrium. Nevertheless, the search of protein EC biomarkers has been mostly based on tissue analyses [6–8], hampering the translation into clinical practice. Only few proteomic studies have been performed on uterine aspirates without a focus on EC biomarkers [9–11].

The ideal diagnostic biomarker pipeline consists of sequential phases of discovery, verification and validation. The discovery produces large lists of differentially abundant proteins (i.e. 100 s–1000 s) between simplified biological conditions using a limited number of samples, mostly tissue specimens. In contrast, the validation phase requires a precise and accurate quantification of the most promising biomarker candidates (typically a dozen), in a large set of samples, which is normally a preferred biofluid [12]. Up to date in EC, the vast majority of biomarker studies cover either discovery phases that generates large lists of biomarker candidates [13, 14], most of which have never been further validated; or validation studies focus on a specific protein [15–18], with an increased risk of not generating concrete application and hampering the search of biomarker panels that improve the diagnostic performance of individual proteins. The intermediate verification phase is crucial for the prioritization of biomarker candidates to enter a validation phase in order to increase the likelihood of identifying clinically relevant biomarkers [19]. The lack of methods to guide the prioritization of candidates in the verification phase has been identified as one of the factors of poor translation of biomarker discovery into clinics [20, 21]. The liquid chromatography mass spectrometry (LC-MS) platform, operated in targeted acquisition mode, is ideal to achieve this task as proteins can be reliably quantified in a highly multiplexed fashion and at a fast throughput.

In this study we aimed to i) demonstrate the efficiency of a stepwise verification workflow that prioritizes, from a list of potential biomarkers derived from published discovery studies, the most promising to enter into a further validation phase; ii) evaluate the performance of the parallel reaction monitoring (PRM), a targeted acquisition method employed on a high resolution accurate mass spectrometer, in clinical samples of uterine aspirates; and iii) assess the potential of the soluble fraction of uterine aspirates as a source of protein EC biomarkers.

## RESULTS

### LC-PRM method development: Selection of the biomarker candidates

**Figure 1:**
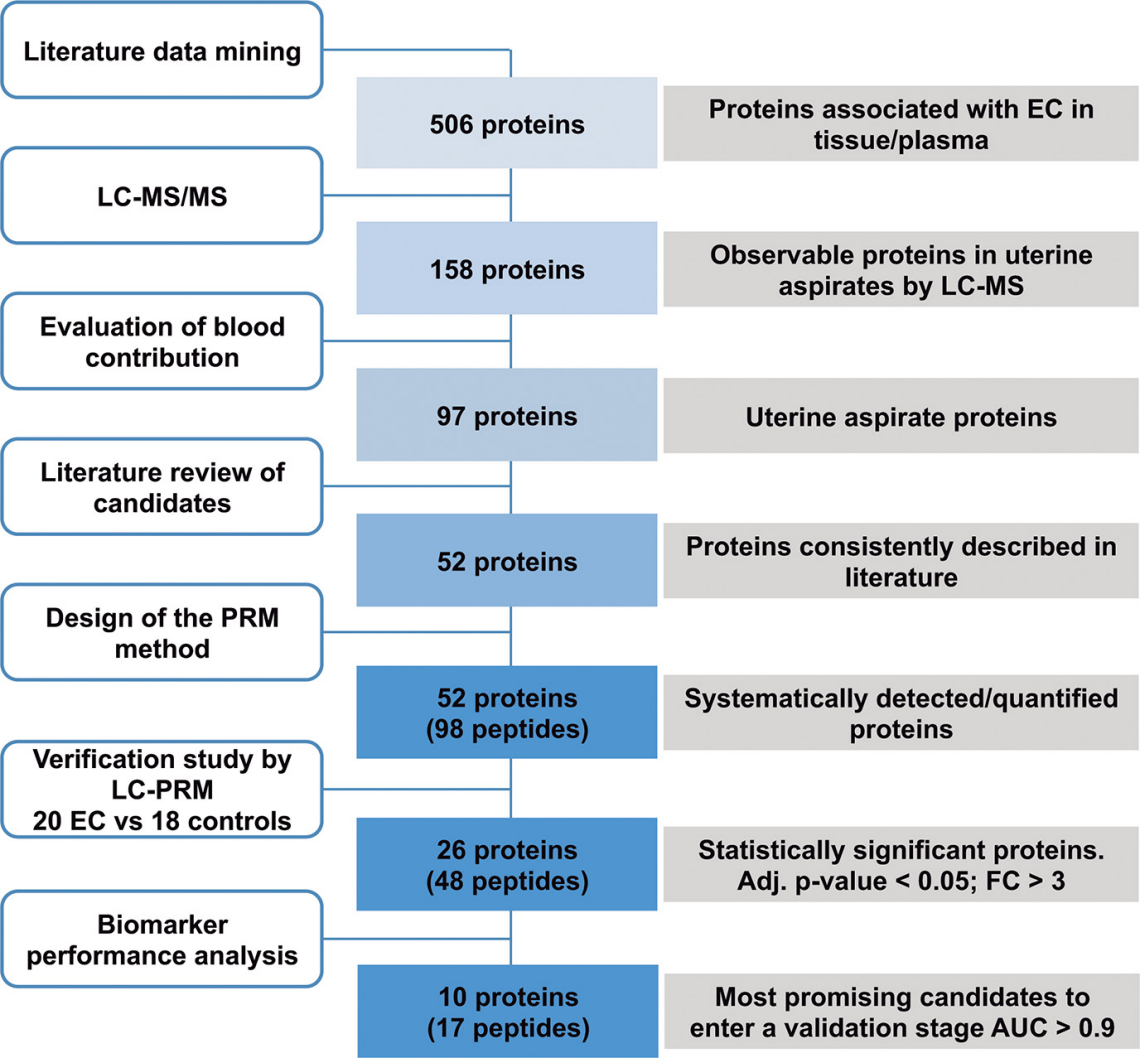
Experimental design Stepwise workflow for the selection and prioritization of endometrial cancer biomarker candidates, and their verification in uterine aspirates by LC-PRM. DDA, data-dependent acquisition; PRM, parallel-reaction monitoring; EC, endometrial cancer; Adj *p*-value, adjusted *p*-value; AUC, Area under the ROC curve.

A targeted MS-based approach was selected for the verification of potential EC biomarkers in uterine aspirates as it enables the quantification of multiple peptides within a single analysis. The LC-PRM is a hypothesis driven methodology that differs from the unsupervised MS-based approaches (*e.g.* data dependent and data independent acquisition) as the proteins must be selected prior the actual MS acquisition. With LC-PRM, the number of targets is limited to approximately 100–150 peptides per analysis, but in return, the mass accuracy and the high resolving power of the orbitrap analyzer, in conjunction with the use of isotope labeled peptides as internal standard, allows for systematic quantitative measurements in all samples achieved with a high degree of selectivity and precision. Therefore, a preselection of the protein candidates to be measured is required. Starting from 506 protein candidates found in the literature from previous studies mainly using EC tissue samples as biological material, we proposed a workflow to reduce step by step this number down to 52 biomarker candidates, leading to 98 pairs of light/heavy peptides that can be measured by a single LC-PRM method. We verified those candidates in uterine aspirates from a cohort of 20 EC patients and 18 controls by LC-PRM (Figure 1).

The first step of this study was to select proteins indicated as potential biomarkers for EC from an extensive literature review performed in the PUBMED bibliographic database. The search included articles published from 1990 to 2014, combined with the text words“*endometrial cancer”* and *“biomarker”*. We obtained a first list of 506 proteins associated with EC, which were mostly derived from studies performed in endometrial tissue samples. The second step of selection consisted in the assessment of the LC-MS detectability of those 506 potential biomarkers in four samples of uterine aspirates by repeated DDA analysis. The main goal of this step was to reduce the list of protein candidates initially described in tissue samples to those that can be effectively detected in uterine aspirate samples. From a total of 1,086 proteins identified in the four uterine aspirates, 158 proteins out of the initial 506 potential biomarkers list were detected (Supplementary Table 1). This first screening indicated that one third of the potential biomarkers could be easily detected by LC-MS techniques in uterine aspirates samples, thus confirming the potential of uterine aspirates as a source of protein EC biomarkers.

**Figure 2:**
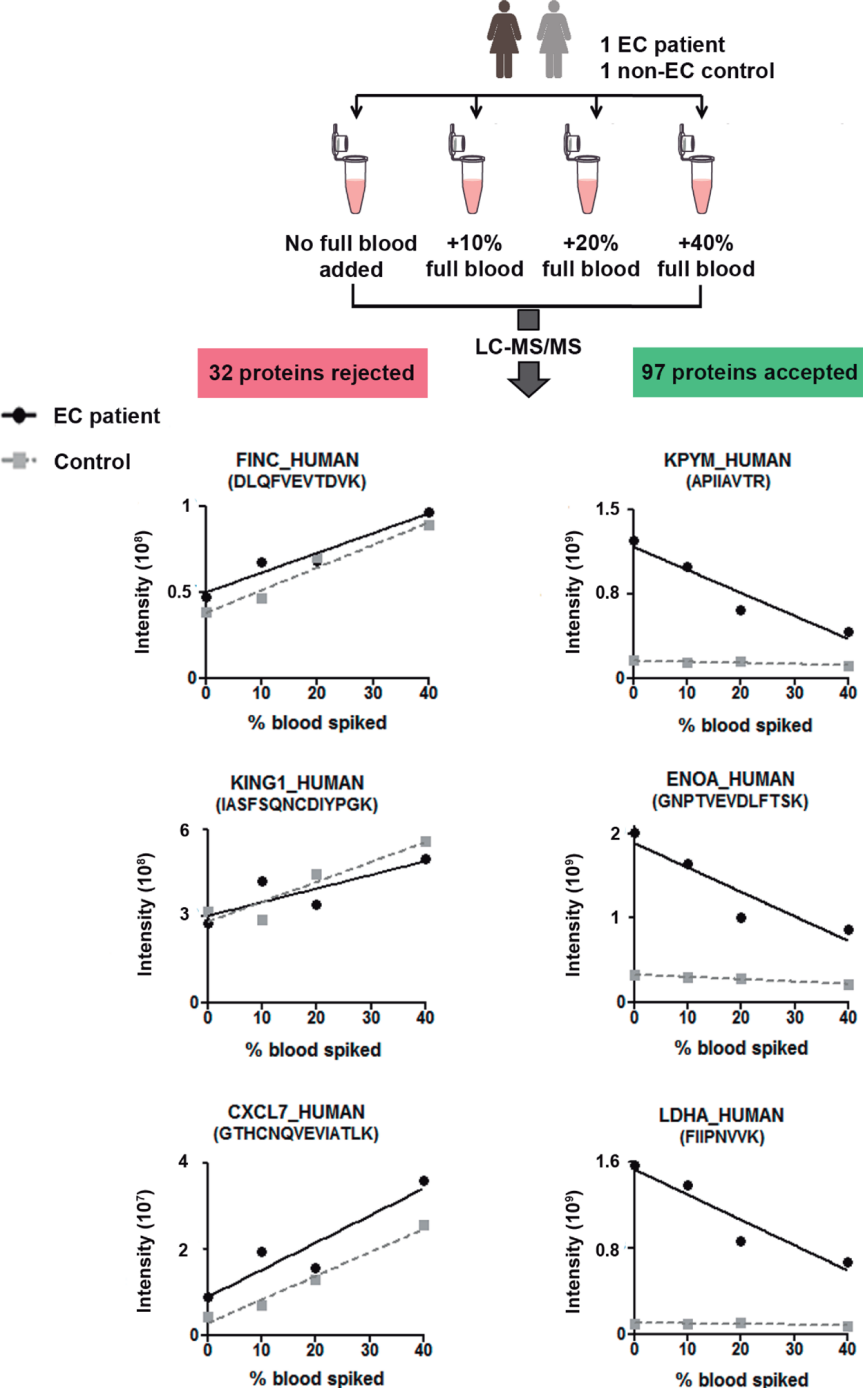
Effect of blood content on biomarker candidate detection Experimental design and examples of concentration profiles of 3 potential biomarkers showing increasing and 3 decreasing profiles when uterine aspirate is diluted by increasing amount of full blood. The 32 candidates showing an increasing profile were rejected for further steps in the study.

Blood contamination of biological samples is a recurrent problem in bioanalyses, particularly in the field of biomarker research in some biofluids [22, 23]. Understanding that uterine aspirates display a variable amount of blood between samples, we introduced a third step of selection to evaluate the interference of blood components during LC-MS detection of the potential biomarkers in uterine aspirates. To do that, the uterine aspirates of two patients (one control and one EC patient) were split into four equal-volume aliquots and spiked with increasing volumes of full blood: 0, 10, 20, 40% (v/v). All samples were digested and analyzed by LC-MS/MS in duplicate. We excluded those proteins whose peptides displayed an increasing profile with an increasing concentration of spiked-in blood and maintained those proteins showing no effect or diminished levels (Figure 2). This criterion was used in order to discriminate protein biomarkers belonging to the endometrial tissue contained in uterine aspirates rather than proteins contained in the blood proteome. Moreover, by excluding abundant proteins of blood, we reduced analytical problems related to variable blood contamination among the samples. As a result of this analysis, 32 proteins were likely to be derived from the blood contamination of the uterine aspirates and were excluded from further analysis (Supplementary Figure 1).

The remaining 97 uterine aspirate specific candidates, and seven additional proteins detected in uterine aspirate samples but not in full blood (data not shown), were scaled down to 52 proteins based on their consistency in literature. The 52 candidates had undergone at least one level of additional validation using a different technology or biospecimen type, or an independent cohort of cases and controls whether in the context of the same publication or in an independent report. A total of two peptides per each of these 52 proteins (104 peptides) were selected according to their uniqueness, detection and chromatographic behavior.

### Quality control of the LC-PRM data

**Figure 3:**
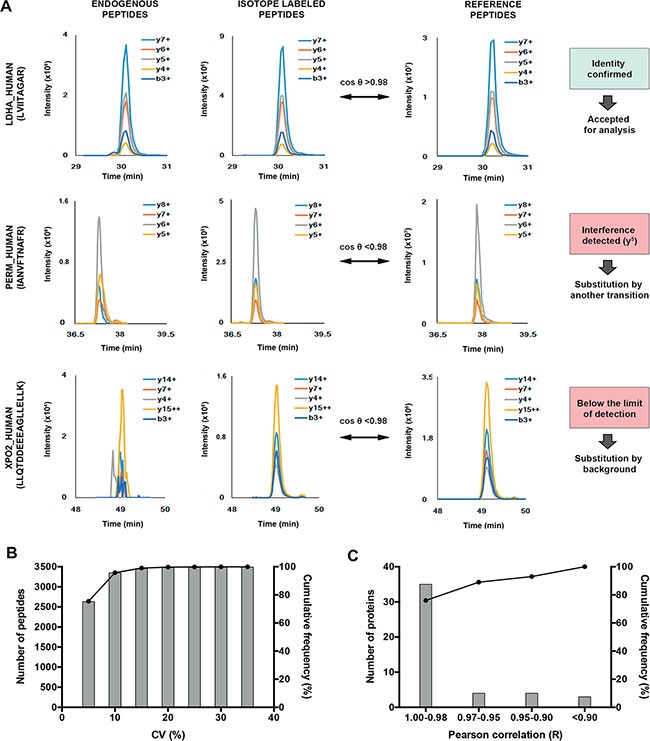
Principle of PRM data quality control (**A**) Peptide identity confirmation by comparison between PRM elution profiles of endogenous and internal standards of each biomarker candidate in the samples and a reference acquisition using the cosine of the spectral contrast angle (θ). A PRM measurement was accepted if the cos (θ) of both endogenous and internal standard are > 0.98. Values below 0.98 due to interferences were solved by the substitution of the interfered XICs. Values below 0.98 due to the limit of detection were substituted by background. (**B**) Reproducibility of the analytical workflow. The sample preparation was duplicated and the coefficient of variation (CV) was below 15% for 99% of the detected peptides. (**C**) Pearson correlation between signatures peptides coming from the same protein. The score below 0.90 for three proteins is due to isoform specific peptides.

The 52 proteins of interest were verified in the soluble fraction of uterine aspirates by targeted MS. Uterine aspirates from 20 EC patients and 18 non-EC controls were digested in duplicate and analyzed by a quadrupole-orbitrap MS operated in PRM mode using a mix of the stable isotopes labeled (SIL) peptides of the 104 peptides (*i.e.,* heavy peptides) as internal standards. Four of these SIL peptides could not be synthesized, leading to a final list of 100 monitored peptides in the method (Supplementary Table 2). The signals of the five most intense product ions for each precursor were extracted from the MS2 spectra to generate elution profiles (*i.e.* Extracted Ion Chromatograms (XICs) of selected product ions) (Supplementary Table 3). The identity of the peptides, as well as the potential interferences on the PRM traces, were evaluated by a similarity score based on the cosine of the spectral contrast angle (cos θ) calculated with the top five fragment ions of each precursor. This score was calculated against a reference LC-PRM analysis of the isotopically labeled peptides without biological matrix (Figure 3A). The signal of a peptide was accepted if the cos θ was higher than 0.98 for both the endogenous and the stable isotope labeled standard [24]. Values lower than 0.98 due to an interfered PRM XIC were replaced by the next most intense available XIC of product ion. Six peptides were monitored by XICs of four product ions due to the absence of a clean fifth product ion (Supplementary Table 2). Following this, a positive spectral matching was achieved for 95.1% of a total of 7,350 pairs (ratio light/heavy). The unmatched 4.9% pairs were due to two conditions: i) measurements below the limits of detection (4.7%), which were replaced with an estimation of the background value. In this account, peptides below the limit of detection in more than 50% of the samples, only two peptides -VHITSLLPTPEDNLEIVLHR and VTILELFR- fulfilled this condition, were removed from the study; and ii) measurements for which less than four clean XICs of product ions were detected (0.2%). These 0.2% were due to data very close to, but below, cos θ = 0.98 and only one replicate was affected in all cases; thus the value of the accepted replicates was kept. These results illustrate the efficiency of the PRM acquisition in complex clinical samples. The use of internal standards and the availability of all XICs of product ions guarantee the correct identification of each peptide, reduce interferences and fasten the detection and exclusion of potential interferences in large datasets. The mean between duplicates in the cleansed dataset was calculated (Supplementary Table 4), as well as the correspondent coefficient of variation (CV%). The CV% of the duplicated sample preparation for each uterine aspirate sample was below 15% for 99% of the detected peptides, with an averaged CV of 3.6%. This confirmed the high reproducibility level of the full process (Figure 3B). Finally, the correlation between the peptides derived from the same protein was evaluated by a Pearson correlation coefficient (Figure 3C) and 39 out of the 46 (85%) proteins monitored with two peptides showed a very high correlation, with a R coefficient over 0.95. Only 3 proteins -ROA2, OSTP, and KPYM- presented an R coefficient below 0.9, which were due to the specificity of the monitored peptides to different isoforms of the same protein.

### Differentially abundant proteins between endometrial cancer and control uterine aspirates

In order to assess the potential of the 52 selected proteins to detect EC, we compared the abundance of each biomarker candidate between 20 EC patients and 18 non-EC controls. Importantly, both patients and controls were postmenopausal women suffering from an abnormal vaginal bleeding, as these clinical features are present in 93% of patients suffering from EC. However, only 15% of those will be finally diagnosed with EC [25].

Based on the Bradford assays, 250 ng of the total protein concentration after albumin and IgGs depletion was injected for each sample. The constant amount of injected protein among samples was further confirmed by the integration of the total ion chromatogram of the MS1 scans, as shown in Supplementary Figure 2. After MS data curation, the relative levels (light/heavy ratios) of the final 98 monitored peptides in MS2 were subjected to Mann Whitney test for their comparison between tumor and control samples. Forty eight peptides corresponding to 26 proteins showed significant differences between the two groups with adjusted *p*-value < 0.05 (Benjamini corrected) and fold change greater than 3: PERM, CADH1, SPIT1, ENOA, MMP9, LDHA, CASP3, KPYM, PRDX1, OSTP, PDIA1, NAMPT, MIF, CTNB1, K2C8, ANXA2, CAPG, FABP5, MUC1, CAYP1, XPO2, NGAL, SG2A1, ANXA1, HSPB1, PIGR. All these proteins showed higher levels in tumor samples as compared to control samples (Table 1; Supplementary Figure 3).

**Table 1:** Proteins showing statistical differences between EC and control patients with adjusted *p*-value < 0.05 and fold change > 3

Uniprot Accession Number	Entrez Gene Name	Protein ID	Peptide	FC	Adjusted *P*-value	Tumor. Q1(25%)-Q3(75%)	Control. Q1(25%)- Q3( 75%)	AUC	Sensitivity (%)	Specificity (%)	Sensitivity (%) when 95% Specificity	Location
P05164	Myeloperoxidase	PERM	IANVFTNAFR	14.1	6.E–05	0.56–2.18	0.04–0.13	0.97	95	89	80	Cytoplasm
			VVLEGGIDPILR	13.3	1.E–04	0.94–4.00	0.08–0.29	0.95	95	89	70	
P12830	E-cadherin	CADH1	VFYSITGQGADTPPVGVFIIER	3.8	9.E–05	0.55–1.27	0.11–0.28	0.94	95	89	85	Plasma Membrane
			NLVQIK	3.3	2.E–04	0.44–1.09	0.12–0.25	0.93	85	94	85	
O43278	Kunitz-type protease inhibitor 1	SPIT1	SFVYGGCLGNK	3.3	1.E–04	0.33–0.66	0.07–0.20	0.93	95	94	95	Extracellular Space
			WYYDPTEQICK	3.3	1.E–04	0.30–0.55	0.06–0.18	0.93	90	94	90	
P06733	Alpha-enolase	ENOA	YISPDQLADLYK	3.8	1.E–04	13.43–25.66	2.89–5.76	0.92	75	94	75	Cytoplasm
			TIAPALVSK	4.0	2.E–04	6.85–18.62	1.51–3.23	0.89	80	83	70	
P14780	Metalloproteinase 9	MMP9	SLGPALLLLQK	5.7	1.E–04	0.52–2.42	0.05–0.19	0.91	95	83	60	Extracellular Space
			AFALWSAVTPLTFTR	5.5	1.E–04	0.35–1.60	0.03–0.14	0.91	90	83	60	
P00338	Lactate dehydrogenase A	LDHA	LVIITAGAR	6.2	1.E–04	3.55–7.23	0.26–0.78	0.91	85	89	65	Cytoplasm
			VTLTSEEEAR	5.7	1.E–04	11.32–22.52	0.94–2.75	0.91	85	89	60	
P42574	Caspase-3	CASP3	SGTDVDAANLR	4.9	2.E–04	0.04–0.11	0.00–0.02	0.91	90	89	65	Cytoplasm
P14618	Pyruvate kinase	KPYM_Isoform M1-M2	NTGIICTIGPASR	5.4	1.E–04	10.82–41.42	1.29–5.52	0.91	85	89	75	Cytoplasm
		KPYM: Isoform M1-M3	APIIAVTR	3.1	1.E–02	0.43–1.39	0.10–0.51	0.75	60	89	50	
Q06830	Peroxiredoxin-1	PRDX1	LVQAFQFTDK	4.2	2.E–04	11.08–27.24	2.06–7.32	0.90	75	94	75	Cytoplasm
			ADEGISFR	4.2	2.E–04	0.80–1.93	0.16–0.52	0.90	75	94	75	
P10451	Osteopontin	OSTP_Isoform A	ANDESNEHSDVIDSQELSK	11.4	2.E–04	0.11–0.44	0.00–0.05	0.90	80	94	80	Extracellular Space
		OSTP_Isoform A, B, D	AIPVAQDLNAPSDWDSR	9.0	4.E–04	0.10–0.56	0.01–0.07	0.87	80	83	50	
P07237	Protein disulfide-isomerase	PDIA1	ILEFFGLK	3.3	3.E–04	0.16–0.41	0.03–0.13	0.89	75	89	65	Cytoplasm
			ALAPEYAK	3.0	3.E–04	0.26–0.65	0.06–0.22	0.88	75	89	65	
P43490	Visfatin	NAMPT	YLLETSGNLDGLEYK	4.2	3.E–04	0.31–1.04	0.01–0.16	0.88	90	83	40	Extracellular Space
			YDGHLPIEIK	4.0	3.E–04	0.57–2.05	0.08–0.32	0.88	90	83	40	
P14174	Macrophage migration inhibitory factor	MIF	VYINYYDMNAANVGWNNSTFA	4.2	3.E–04	0.91–1.89	0.05–0.45	0.88	75	94	75	Extracellular Space
			LLCGLLAER	3.1	3.E–04	45.14–98.96	11.49–27.40	0.87	70	94	70	
P35222	Beta-catenin	CTNB1	LLNDEDQVVVNK	4.2	3.E–04	0.06–0.21	0.00–0.04	0.88	85	89	70	Nucleus
			LVQLLVR	4.2	3.E–04	0.07–0.27	0.00–0.04	0.87	85	89	65	
P05787	Keratin, type II cytoskeletal 8	K2C8	LSELEAALQR	3.6	3.E–04	1.04–2.99	0.17–0.92	0.88	95	67	65	Cytoplasm
			WSLLQQQK	3.1	6.E–04	0.45–1.25	0.09–0.45	0.85	60	94	60	
P07355	Annexin A2	ANXA2	GVDEVTIVNILTNR	4.8	4.E–04	5.60–20.24	1.36–4.74	0.87	75	89	45	Plasma Membrane
			QDIAFAYQR	5.1	5.E–04	0.26–1.07	0.05–0.25	0.86	95	61	50	
P40121	Macrophage-capping protein	CAPG	EGNPEEDLTADK	3.6	5.E–04	0.32–1.11	0.05–0.17	0.85	85	83	45	Nucleus
			YQEGGVESAFHK	3.5	6.E–04	0.44–1.63	0.08–0.27	0.85	80	83	45	
Q01469	Fatty acid binding protein 5, epidermal	FABP5	LVVECVMNNVTCTR	3.9	6.E–04	2.27–8.29	0.84–1.56	0.85	90	78	45	Cytoplasm
			ELGVGIALR	3.6	6.E–04	0.03–0.10	0.01–0.02	0.85	90	78	45	
P15941	Mucin-1	MUC1	QGGFLGLSNIK	3.6	1.E–03	5.11–12.65	1.18–4.04	0.84	85	78	45	Plasma Membrane
Q13938	Calcyphosine	CAYP1	SGDGVVTVDDLR	3.4	1.E–03	4.04–19.35	1.16–3.48	0.83	85	78	45	Cytoplasm
P55060	Exportin-2	XPO2	ANIVHLMLSSPEQIQK	4.0	1.E–03	0.05–0.19	0.00–0.04	0.83	75	89	25	Nucleus
			LLQTDDEEEAGLLELLK	4.4	2.E–03	0.04–0.22	0.00–0.04	0.81	70	89	40	
P80188	Lipocalin2	NGAL	VPLQQNFQDNQFQGK	5.0	1.E–03	2.19–8.19	0.29–2.03	0.83	75	89	35	Extracellular Space
			ELTSELK	4.4	4.E–03	2.09–9.13	0.35–2.04	0.79	70	83	30	
O75556	Mammaglobin-B	SG2A1	ELLQEFIDSDAAAEAMGK	3.3	3.E–03	0.15–0.35	0.02–0.17	0.80	90	72	30	Extracellular Space
			TINSDISIPEYK	3.2	5.E–03	0.12–0.30	0.02–0.14	0.78	90	67	40	
P04083	Annexin A1	ANXA1	DITSDTSGDFR	4.8	3.E–03	1.12–4.44	0.33–1.16	0.80	60	100	60	Plasma Membrane
			GGPGSAVSPYPTFNPSSDVAALHK	3.9	7.E–03	1.33–6.02	0.51–2.02	0.77	55	100	55	
P04792	Heat shock 27kDa protein 1	HSPB1	LFDQAFGLPR	3.6	4.E–03	1.31–7.31	0.60–1.78	0.79	85	67	40	Cytoplasm
			LATQSNEITIPVTFESR	3.1	4.E–03	2.74–13.66	1.27–3.67	0.79	85	67	40	
P01833	Polymeric immunoglobulin receptor	PIGR	VYTVDLGR	3.4	7.E–03	38.67–128.80	15.43–37.38	0.77	80	78	30	Plasma Membrane

FC, fold change; AUC, area under the curve; Q1, first quartile; Q3, third quartile.

**Figure 4:**
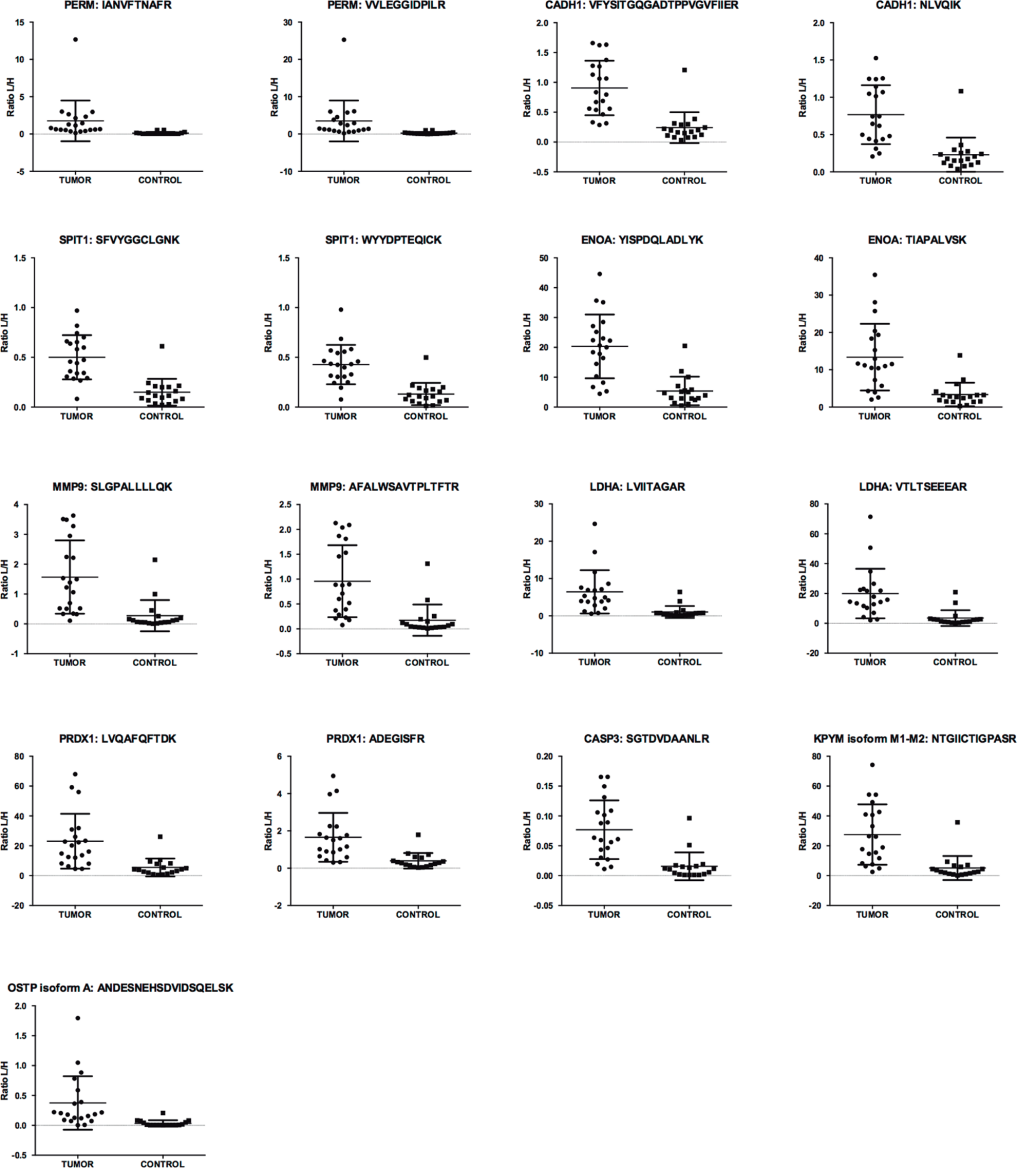
Scattering plots of the abundance of 17 peptides coming from 10 biomarkers in the verification study Scattering plots depicting the distribution of the light/heavy (L/H) ratios across the 20 EC patients and 18 controls of the best individual performing peptides (AUC > 0.9) belonging to 10 biomarkers.

To further evaluate their performance as biomarkers for EC diagnosis, we performed a ROC analysis to determine the sensitivity and specificity of each biomarker. Interestingly, these differentially abundant proteins showed Area Under the ROC Curve (AUC) values for discriminating between EC and controls patients ranging from 0.75 to 0.97. The 10 best-performing individual proteins were PERM, CADH1, SPIT1, ENOA, MMP9, LDHA, CASP3, KPYM isoform M1-M2, PRDX1 and OSTP isoform A, all of them with AUC values higher than 0.9 (Figure 4). Among those proteins, PERM, CADH1, SPIT1 and OSTP isoform A were of special interest as each of them presented sensitivities higher than 80% when specificity was fixed to 95% (Table 1). This is particularly important in EC diagnosis, as biomarkers with high specificity could complement the output of low invasive techniques such as the transvaginal ultrasonography, which currently presents very high sensitivity but lack of specificity [26].

Furthermore, we conducted a bioinformatics analysis using Ingenuity Pathway Analysis (IPA) to better understand the association of these proteins with cancer and their origin regarding the subcellular location. As expected, integration of the data resulted in the identification of cancer, inflammatory disease, organismal injury and abnormalities, and reproductive system disease as the top diseases associated to these biomarkers. The top five molecular and cellular functions involved with these proteins included cellular movement, cellular death and survival, cellular development, cellular growth and proliferation, and celltocell signaling and interaction, all of them important processes altered in cancer. These proteins are mainly found in the cytoplasm, plasma membrane and extracellular space (Table 1), indicating that they are coming either from secretion of the epithelial and inflammatory cells of the endometrium or by necrosis of cells in the proximal tissue. This is in concordance with the observation that all biomarkers in this study were found more abundant in EC patients as compared to controls, as both processes are related to the higher proliferation rate of epithelial cells in EC.

## DISCUSSION

About 30% of EC patients are diagnosed at advanced stages of the disease, associated with a drastic increase in the mortality and morbidity [1]. Therefore, the identification of sensitive and specific biomarkers to improve early detection of EC is a crucial clinical need. Despite the major effort and investments made to identify EC biomarkers, no protein has yet reached the stage of clinical application. The poor translation of the results produced by those studies in the clinic can be explained by two determinant factors: on the one side, the lack of studies in biofluids to identify accessible EC biomarkers. Most of the studies were based in tissues, and/or those that used biofluids were limited to serum or plasma [27–29]. Blood presents several important advantages, as it is in direct contact with all body tissues, and its collection is rapid, easy and minimally invasive. However, the search of biomarkers in plasma or serum is extremely challenging due to the low concentration of the potential biomarkers and the wide dynamic range in protein abundance [30]. On the other side, the lack of verification studies as a bridge between discovery and validation phases, which has been defined as the current bottleneck of the biomarker pipeline [19, 21]. Discovery studies generate large lists of differentially abundant proteins. Many of those biomarker candidates are never validated or turn to be false positives due to the small number of samples analyzed, the biological variability or the limited quantitative performance of the technologies employed in this phase. There is a need to verify and refine those lists to the best candidates that can enter a validation phase. This is the critical role of the verification phase. In order to overcome these limitations, we presented a stepwise workflow to select potential EC biomarkers and verified them by targeted MS-based analysis in uterine aspirate samples.

Targeted MS-based approaches have gained in popularity for biomarker verification in complex clinical samples because they combine precision, sensitivity, multiplexing and absence of missing values. Among those, selected reaction monitoring (SRM) acquisition mode performed on a triple quadrupole mass spectrometer has been the reference method for the accurate quantification of peptides in biological matrices [31–33]. However, SRM is limited in selectivity and requires a substantial method development. We implemented the PRM acquisition, a new generation of targeted MS-based approach, performed on high-resolution accurate mass spectrometers (HRAM) such as the hybrid quadrupole-orbitrap. To date, the advantage of PRM for large scale analysis has been evaluated [24, 34] but not yet commonly introduced as a technique for biomarker searches in clinical [35] or cell lines samples [36–38]. The high resolution and the accurate mass (i.e. 35,000 at 200 m/z and below 5 ppm) of the orbitrap analyzer decrease the risk of inferences due to the complexity of the chemical background and we obtained clear and easily readable chromatograms profiles. This aspect, in conjunction with the use of spectral matching as a quality metric, significantly facilitates the data processing to compare with SRM data, for instance only 0.2% of the chromatographic peak needed to be manually curated. A straightforward and highly automatable data processing is an important feature of large scale studies. The PRM acquisition allowed the quantification of 100 pairs of peptides at the reasonable throughput of one analysis per hour with an excellent precision (i.e. the CV% between full workflow duplicates was below 15% for 99% of the detected peptides). Finally, the design of an LC-PRM method is easier and faster than for LC-SRM, as the selection of the product ions to quantify is performed post-acquisition and the list of XICs can be refined iteratively to remove potential interferences coming from the background, without the need of a new analysis [39].

Another strength of this study is the use of uterine aspirates as a biological sample for biomarker detection. A useful diagnostic biomarker not only has to ameliorate the discrimination between patients suffering the disease and benign cases, but also should be economically profitable and advantageous in the clinical scenario. In the case of diagnostic biomarkers for EC, fasten the diagnostic process, improving the comfort of patients, and reducing the sanitary costs are very important values. Therefore, the search of biomarkers in easy-to-access biofluids is highly recommended. Uterine aspirates seem an interesting alternative to other biofluids, such as blood or plasma, as they are in direct contact with the tumor in the endometrium, being enriched in proteins secreted from the epithelial cells in the tumor. Additionally, they are collected in the current process of EC diagnosis prior to subsequent more invasive diagnostic techniques. We here demonstrated the convenience of the soluble fraction of uterine aspirates as a source of EC biomarkers and the feasibility of its analysis by MS. The use of the soluble fraction is expected to overcome the diagnostic failure of 22% associated to this sampling, as the current diagnostic procedure relies on the cellular material in the sample [4].

Our final achievement was to eliminate doubtful biomarker candidates derived from the variable amounts of blood contamination in uterine aspirates and successfully verify the differential abundance of 26 EC biomarkers in this sample. A bioinformatics analysis confirmed their individually and collectively association with cancer, and showed that they maintain a strong association with commonly altered molecular processes in cancer such as cellular movement, cellular death and survival, etc. Among all candidates, ten provided high sensitivity and specificity, with AUC values over 0.9, and four of those, PERM, CADH1, SPIT1 and OSTP, were highlighted as they achieved sensitivity over 80% when fixing a specificity of 95%. The protein biomarkers verified in this study merit further validation in an extended study with a larger cohort of patients and controls. A prospective large multicentric study has been initiated with the aim to confirm the diagnostic power of these biomarkers and hence, to evaluate their validity and clinical applications. A limitation of the present study is that we did not use combinations of multiple markers to avoid overfitting due to the relatively small number of subjects included [40]. However, the AUC for individual proteins were already very high.

In conclusion, this study brings forward the proteomic search of biomarkers in uterine aspirates following an appropriate workflow, and so, could be expanded to other types of gynecological diseases such as endometriosis and ovarian cancer. Moreover, this study proves the efficiency of high resolution MS in order to verify a large number of potential biomarkers to fill the gap between discovery and validation studies. The described workflow permitted to reduce step by step an initial list of 506 potential biomarkers down to 10 proteins with an increased likelihood to reach the stage of a clinical assay after a subsequent validation phase.

## MATERIALS AND METHODS

### Reagents

Albumin and IgG Depletion SpinTrap columns were purchased from GE Healthcare (cat.no. 28-9480-20). Sequencing grade modified trypsin was obtained from Promega (cat.no. V5111) and LysC endoproteinase MS grade was purchased from Thermo Scientific (cat.no. 90051). Solid phase extraction cartridges, Sep Pak tC18, 50 mg, were obtained from Waters (cat.no.WAT054960). SIL peptides were synthetized with a heavy C terminal lysine or arginine (C terminal arginine, ^13^C6, ^15^N4, Δm = 10 Da, C terminal lysine ^13^C6, ^15^N2, Δm = 8 Da) or when it was not applicable with a heavy leucine ^13^C6, ^15^N1, Δm = 7 Da or phenylalanine ^13^C9, ^15^N1, Δm = 10 Da. (Thermo Fisher, crude quality). The synthetic peptides were mixed together from the stock solutions (50% acetonitrile, 0.1% TFA), aliquoted in Eppendorf Low Bind tubes and stored at 80°C before single use. All other reagents were obtained from Sigma-Aldrich.

### Patients and sample collection

A total of 42 patients (22 women suffering from EC and 20 non-EC controls, *i.e.,* women having EC symptoms but not diagnosed with EC) were recruited in the Vall d'Hebron University Hospital (Barcelona, Spain) during 2012 to 2015. Informed consent forms, approved by the Vall d´Hebron Ethical Committee, were signed by all patients (approval number: PR_AMI_50-2012). The clinical and pathological characteristics of the patients are described in Supplementary Table 5. Inclusion criteria were postmenopause, a minimum age of 50 years and vaginal bleeding. Women who had been treated previously for gynecological pelvic cancer were excluded. Patients known to be positive for the human immunodeficiency virus and/or the hepatitis virus were excluded for safety reasons.

Uterine aspirates were collected by aspiration with a Cornier Pipelle (Eurogine Ref. 03040200) in the office of the clinician or in the operating room prior to surgery and transferred to 1.5 ml microtubes. Phosphate buffer saline was added in a 1:1 (v/v) ratio and centrifuged at 2,500 rcf for 20 min in order to separate the soluble fraction (supernatant) from the solid fraction (pellet). The separated fractions were kept at −80°C until use. From the 42 supernatants collected, samples coming from four patients were used for potential biomarker selection process and the development of the LC-PRM method. The list of selected biomarker candidates was then verified in the 20 EC and 18 nonEC remaining samples by LCPRM analysis.

### Evaluation of the detectability of potential protein biomarkers in uterine aspirates by LC-MS/MS analysis

Uterine aspirate supernatants from two patients diagnosed with EC and two non-EC controls were sonicated (Labsonic M, Sartorius Stedim Biotech) at 100% amplitude during 8 cycles of 15 seconds and 50 μl of each sample was depleted from albumin and IgG using the Albumin & IgG depletion spin trap kit according to the manufacturer's instructions. Total protein concentration was measured by the Bradford assay, performed in triplicate. Proteins were purified by acetone precipitation overnight at −20ºC, resuspended with 0.2% *Rapigest* surfactant (Waters), sequentially digested at 37ºC by Lys-C (protease/total protein amount ratio of 1/150 w/w) and trypsin (1/50 w/w) overnight, and finally desalted onto SPE cartridges. The LC-MS detectability of 506 potential biomarkers in the supernatants of uterine aspirate samples was then evaluated using a LTQ-Orbitrap Velos mass spectrometer (Thermo Scientific) operated in data dependent acquisition (DDA) mode. The liquid chromatography system consisted of an UltiMate 3000 RSLC nano configured in binary gradient mode. The setup was operated in column switching mode and samples were loaded onto a trap column (Acclaim PepMap100 2 cm × 75 μm i.d., C18, 3 μm, 100 Å) for 3 min at 5 μl/min by an aqueous solution containing 0.05% trifluoroacetic acid and 1% acetonitrile (v/v). Peptides were then eluted onto an analytical column (Acclaim PepMap RSLC 15 cm × 75 μm i.d., C18, 2 μm, 100 Å) by applying a 66 min linear gradient from 2 to 35% solvent B at 300 nl/min. The solvents A and B consisted of water with 0.1% (v/v) formic acid and acetonitrile with 0.1% (v/v) formic acid, respectively. The electrospray ionization was performed through a fused silica emitter by applying a voltage of 1.5 kV. The DDA method was based on a high resolution survey scan (60,000 at 400 m/z) followed by the fragmentation and analysis of the 6 most intense precursor ions in the LTQ ion trap at normalized collision energy of 35. Dynamic exclusion of precursors already selected for MS/MS experiments was set to 90 s. Peptides and related proteins identification was performed using Proteome Discoverer software (v1.4) (Thermo Scientific, Waltham, MA, USA) by Mascot search engine using Swiss-Prot human database (SwissProt 201108 with 531473 sequences entries, restricted to the 20,245 entries of the human taxonomy). Trypsin specificity was set to cleave after arginine and lysine residues excepted when flanked by a proline on the C-terminal side. A fragment ion mass tolerance of 0.8 Da and a precursor mass tolerance of 10 ppm were applied. Up to one tryptic missed cleavage was tolerated and carbamidomethylation of cystein and oxidation of methionine were specified as dynamic modifications. Results were filtered by Proteome Discoverer using one peptide per protein, a maximum search engine rank of 1 and a false discovery rate (FDR) below 0.01 (calculated by the node “Target decoy PSM validator”). The expectation value for accepting a spectrum was below 4 * 10^−3 to set FDR at 0.01.^

### Effect of differential blood content on biomarker candidate detection in uterine aspirates

Uterine aspirates from one EC and one control patients were split into four equal-volume aliquots and spiked with increasing volumes of full blood (0, 10, 20 and 40% (v/v)). Samples were centrifuged at 2500 rcf for 20 min in order to separate the soluble part from the pellet. Supernatants were treated and analyzed by LC-MS/MS as described in the previous paragraph. The elution profile areas of peptides of 129 potential biomarkers identified in the uterine aspirates of these two patients with the different percentage of blood added and/or full blood were extracted from the high resolution survey scans (the identity of peptide was confirmed by MS2) using Skyline software (v3.1) (McCoss Lab, University of Washington, Seattle, WA, USA). The levels of the surrogate peptides of each protein across the four aliquots with increasing percentage of full blood were plotted. The slope of the linear regression was calculated for each peptide and those presenting a positive slope in both patients were rejected from the study.

### Preparation of uterine aspirate samples for the LC-PRM analysis

Supernatants from uterine aspirates coming from 20 EC patients and 18 non-EC controls were sonicated to disrupt potential microvesicles, protein aggregates, and/or mucus by 5 cycles at 100% amplitude during 5 seconds (Labsonic M, Sartorius Stedim Biotech). Albumin and immunoglobulin G were then depleted from 50 μl of supernatant samples using the Albumin & IgG depletion spin trap kit according to the manufacturer's instructions. Total protein concentration was measured by the Bradford assay performed in triplicate. Each of the 38 samples were then separated into two aliquots of 25 μg to generate duplicates for the whole process, with exception of one sample for which the amount of material was not sufficient for duplication. The samples were diluted into a 50 mM solution of ammonium bicarbonate to a final volume of 120 μl and were denatured by addition of 185 μl of 10 M urea suspended in 50 mM ammonium bicarbonate, incubated at 22°C under agitation for 20 min, and followed by 10 min incubation in an ultrasonic bath (Branson 5510, Branson Ultrasonics). The samples were then reduced with 7.8 μl of 200 mM dithiothreitol for 60 min at 37°C, and alkylated with 12.2 μl of 400 mM iodoacetamide at 22°C for 30 min in the dark. The samples were digested for 4 h at 37°C with LysC (protease/total protein amount ratio of 1/150; w/w). Afterwards, the concentration of urea was diluted to 1 M with 50 mM ammonium bicarbonate buffer, and samples were incubated overnight at 37°C with trypsin (protease/total protein amount ratio of 1/50; w/w). The trypsin activity was quenched by addition of 1 μl of neat formic acid per 100 μl of solution. The samples were spiked with the mix of heavy synthetic peptides and then desalted onto solid phase extraction cartridges. The eluates were subsequently evaporated to dryness in a vacuum centrifuge and suspended in 0.1% formic acid before LC-PRM analysis.

### LC-PRM setup

The LCMS setup consisted of a Dionex Ultimate 3000 RSLC chromatography system configured for a high-pressure binary gradient and operated in column switching mode. The mobile phase A consisted of 0.1% formic acid in water, the phase B in 0.1% formic acid in acetonitrile and the loading phase in 0.05% trifluoroacetic acid and 1% acetonitrile in water. The equivalent of 250 ng of each digested sample was injected and loaded onto a trap column (75 μm × 2 cm, C18 pepmap 100, 3 μm) at 5 μl/min and further eluted onto the analytical column (75 μm × 15 cm, C18 pepmap 100, 2μm) at 300 nl/min by a linear gradient starting from 2 % A to 35 % B in 48 min. The MS analysis was performed by a hybrid quadrupole orbitrap mass spectrometer (Q Exactive plus, Thermo Scientific) operated in PRM mode. The MS cycle started with a full MS1 scan performed at a resolving power of 70,000 (at 200 m/z) followed by time scheduled targeted PRM scans acquired at a resolving power of 35,000 (at 200 m/z) with a normalized collision energy of 20. The quadrupole isolation window for the PRM events was set to 1 m/z unit and the duration of the time scheduled windows for each pair of endogenous and isotopically labeled peptides were set to 2 min. *PRM data are accessible in a public database (Panorama server) at: https://panoramaweb.org/labkey/PRM_analysis_of_EC_uterine_aspirate.url;* reviewer account is: panorama+domon@proteinms.net ; Password is: KTjy3~A#.

### PRM data processing

The elution profile of the five most intense fragment ions of each precursor were extracted using Skyline. The selection of the best product ions was supported by a spectral library obtained from a reference LC-PRM acquisition of the synthetic peptide mix injected without biological matrix. The elution profiles of the samples were first manually reviewed and obvious interfered PRM XICs were replaced by the next most intense available product ion. The data set was then refined using the cosine of the spectral contrast angle (cos θ) calculated between the peak areas of the five XICs of product ions of the reference (PRM acquisition of the synthetic peptides mix) and the areas of the corresponding XICs for the endogenous and heavy peptides in the biological samples [41]. The formula is as follows:

$$cos\;(\theta )=\frac{\sum_{i=1}^{n}\left(A_{exp_{i}}\;\times \;A_{ref_{i}}\right)}{\sqrt{{\sum_{i=1}^{n}\left(A_{exp_{i}}\right)}^{2}}\;\times \;\sqrt{\sum_{i=1}^{n}{\left(A_{ref_{i}}\right)}^{2}}}$$

Where *A*_exp_ are the areas of either the endogenous or heavy XICs of selected product ions for a peptide measured in a sample, and *A*_ref_ are the areas of the same XICs measured in a reference synthetic peptides mixture.

Peptides detection and identification were confirmed if the cos θ of the endogenous and the isotope labeled peptide were higher than 0.98 [24]. Scores below 0.98 are principally due to MS measurements below the limit of detection and in such cases the area values were replaced by an estimation of the background. Peptides with cos θ below 0.98 in more than 50% of the 38 samples in duplicates were eliminated from the verification study.

For the quantitative analysis, the area ratios between the endogenous and their corresponding heavy peptides were compared between samples. The area ratios were calculated as the sum of the areas of the XICs of products ions of the endogenous peptide divided by the sum of the XICs of the respective isotope labeled version.

### Statistical analysis

The statistical analysis was performed in SPSS (v20.0) (IBM, Armonk, NY, USA) and Graph Pad Prism (v.6.0) (GraphPad Software, La Jolla, CA, USA). The averaged light/heavy area ratios were calculated between duplicates. The linear correlation between the signature peptides of the same protein was calculated using the Pearson correlation coefficient. Due to the non-normality of the data, assessed by Kolmogorov-Smirnova and Shapiro-Wilk tests, comparison of the abundance of the monitored peptides between tumor and control samples was performed using the non-parametric Mann-Whitney *U* test. *P*-values were adjusted for multiple comparisons using Benjamini-Hochberg FDR method [42]. Adjusted *p*-values lower than 0.05 along with fold changes greater than three were considered statistically significant. Receiver operating characteristic (ROC) curves were used to calculate the relationship between sensitivity and specificity for EC versus non-EC control group and hence, to evaluate the diagnostic performance for each biomarker candidate.

## SUPPLEMENTARY MATERIALS FIGURES AND TABLES





## Abbreviations

EC: endometrial cancer
MS: mass spectrometry
HRAM: high-resolution accurate mass spectrometers
PRM: parallel reaction monitoring
SRM: selected reaction monitoring
SIL: stable isotopes labeled peptides
ROC: receiver operating characteristic
DDA: data dependent analysis
AUC: area under the curve
IPA: ingenuity pathway analysis
PERM: myeloperoxidase
CADH1: e-cadherin
SPIT1: kunitz-type protease inhibitor 1
ENOA: alpha-enolase
MMP9: metalloproteinase 9
LDHA: lactate dehydrogenase A
CASP3: caspase-3
KPYM: pyruvate kinase
PRDX1: peroxiredoxin-1
OSTP: osteopontin
PDIA1: protein disulfide-isomerase
NAMPT: visfatin
MIF: macrophage migration inhibitory factor
CTNB1: beta-catenin
K2C8: keratin, type II cytoskeletal 8
ANXA2: annexin A2
CAPG: macrophage-capping protein
FABP5: fatty acid binding protein 5
MUC1: mucin-1
CAYP1: calcyphosine
XPO2: exportin-2
NGAL: lipocalin2
SG2A1: mammaglobin-B
ANXA1: annexin A1
HSPB1: heat shock 27kDa protein 1
PIGR: polymeric immunoglobin receptor.
