# Supplementary material for: Development of a sequential workflow based on LC-PRM for the verification of endometrial cancer protein biomarkers in uterine aspirate samples
Source: Oncotarget. 2016 Jul 16;7(33):53102–15. doi: 10.18632/oncotarget.10632 (PMC5288171; doi:10.18632/oncotarget.10632)
Supplement: Supplementary file 1 [file oncotarget-07-53102-s001.pdf]

Development of a sequential workflow based on LC-PRM for the verification of endometrial cancer protein biomarkers in uterine aspirate samples

Supplementary Materials

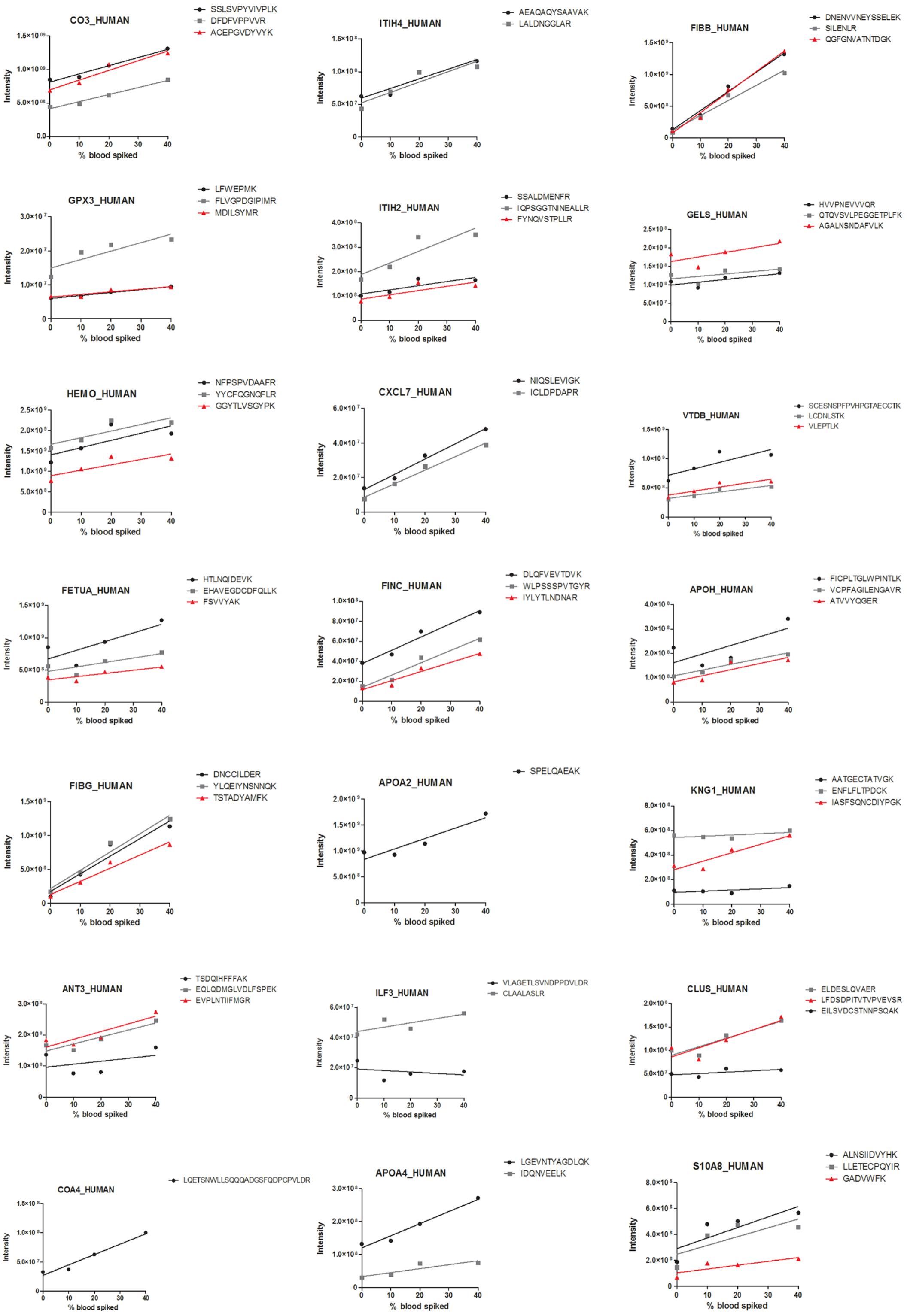

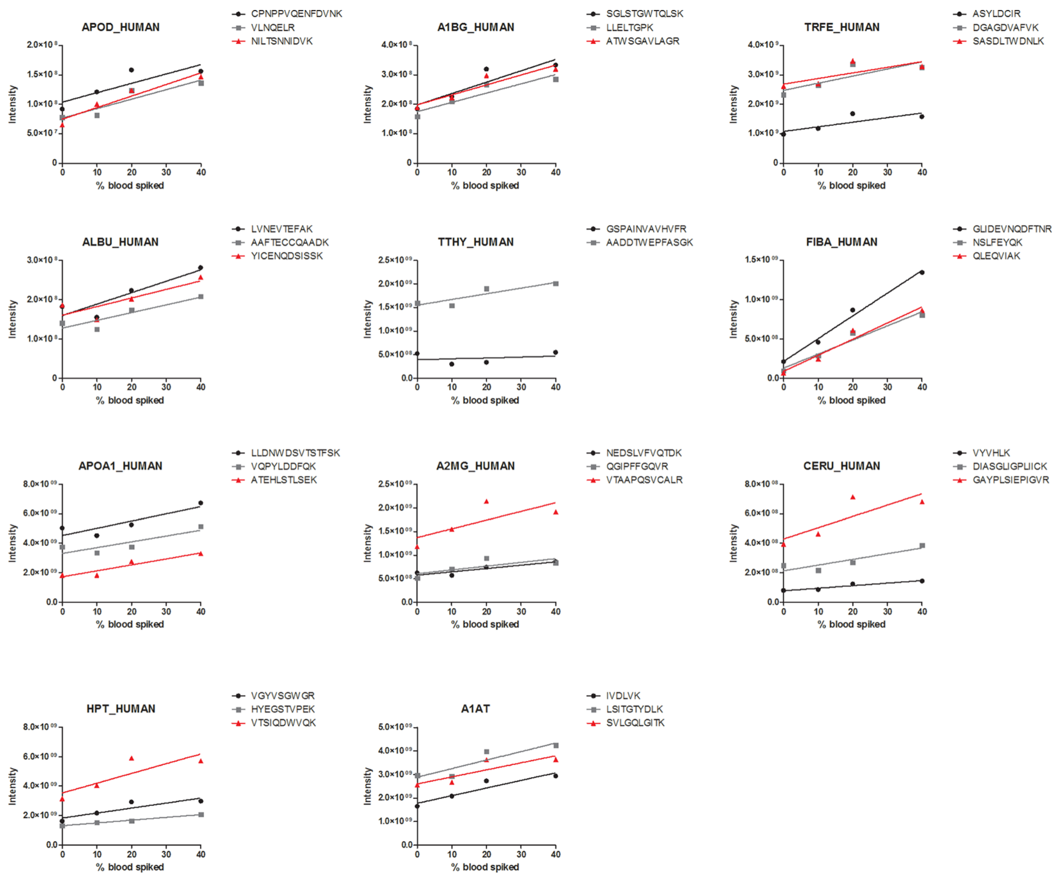

Supplementary Figure S1: Biomarker candidates rejected from the study due to their high abundance in blood. Concentration profile of the peptides coming from 32 proteins showing increasing expression profiles when uterine aspirate samples were diluted with increasing amount of full blood (0, 10, 20, 40% (v/v)).

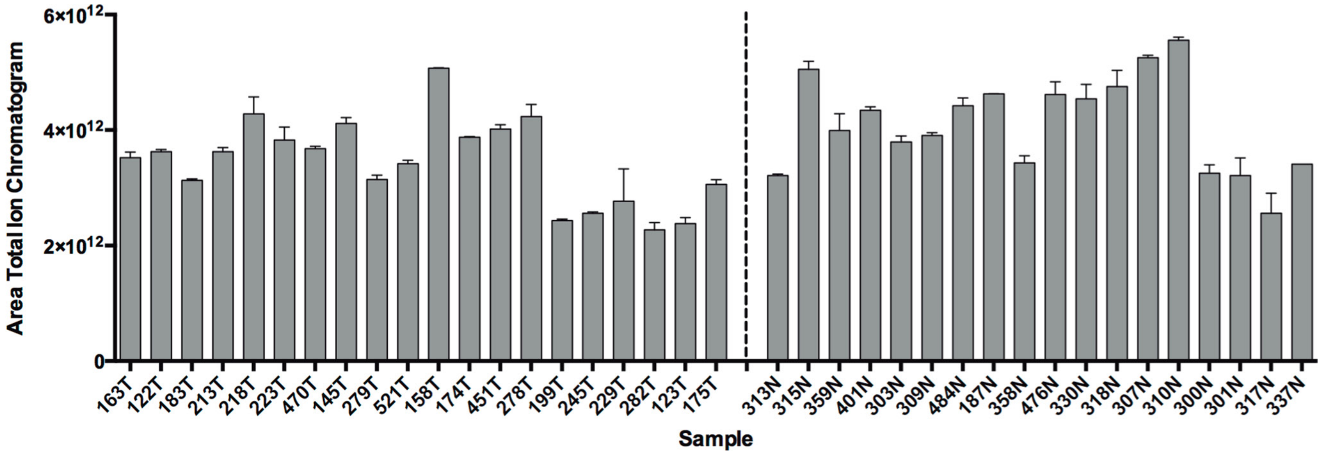

Supplementary Figure S2: TIC of each injected sample to control the total amount of protein injected. Area of the total ion current chromatogram (TIC) of the samples. Samples 199T to 175T and 300N to 337N were acquired in a different batch. T, endometrial cancer patients; N, control women.

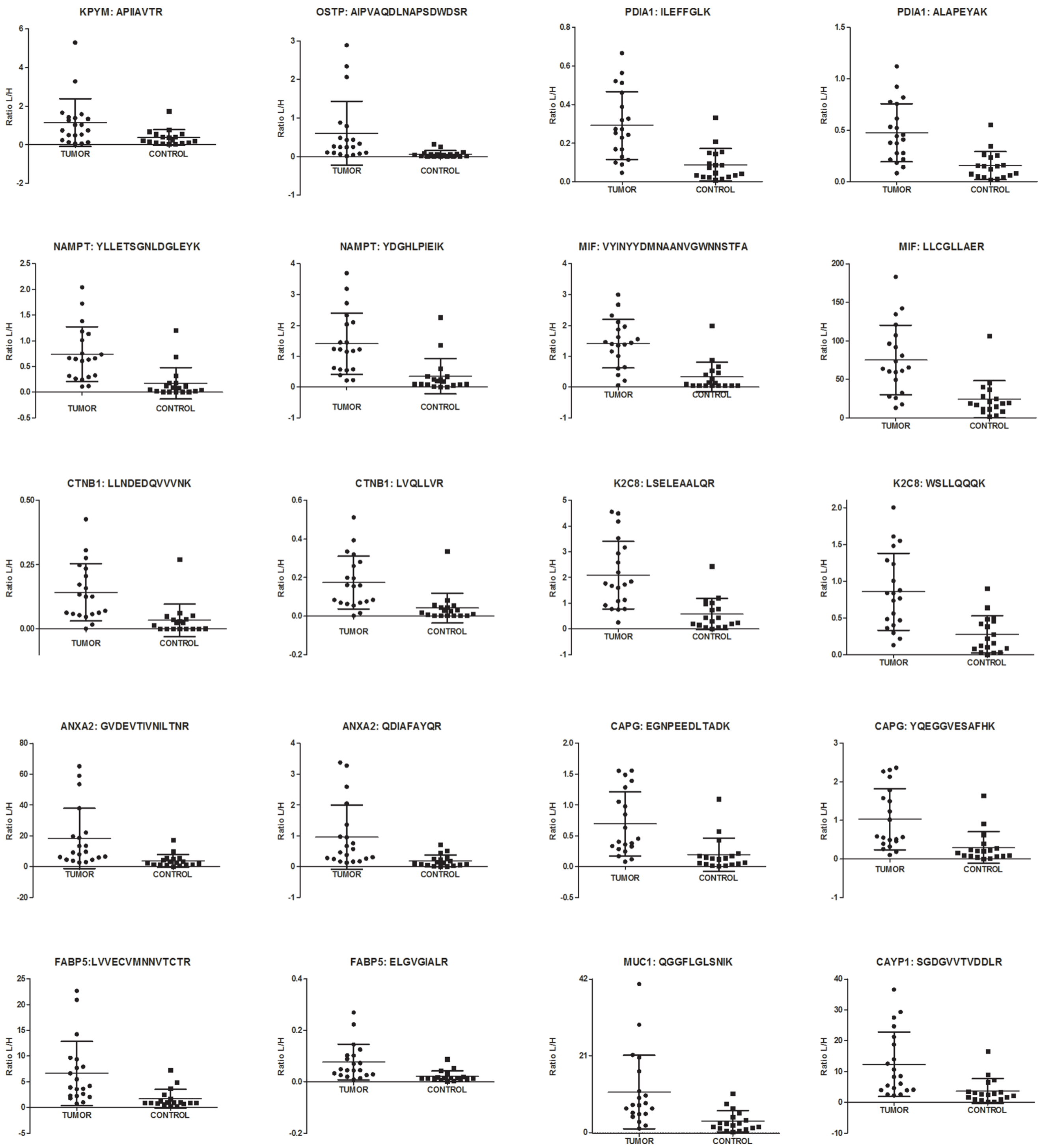
KPYM: APIIAVTR
OSTP: AIPVAQDLNAPSDWDSR
PDIA1: ILEFFGLK
PDIA1: ALAPEYAK
NAMPT: YLLETSGNLDGLEYK
NAMPT: YDGHLPIEIK
MIF: VYINYYDMNAANVGWNNSTFA
MIF: LLCGLLAER
CTNB1: LLNDEDQVVVNK
CTNB1: LVQLLVR
K2C8: LSELEAALQR
K2C8: WSLLQQQK
ANXA2: GVDEVTIVNILTNR
ANXA2: QDIAFAYQR
CAPG: EGNPEEDLTADK
CAPG: YQEGGVESAFHK
FABP5:LVVECVMNNVTCTR
FABP5: ELGVGIALR
MUC1: QGGFLGLSNIK
CAYP1: SGDGVVTVDDLR
Ratio L/H
TUMOR
CONTROL

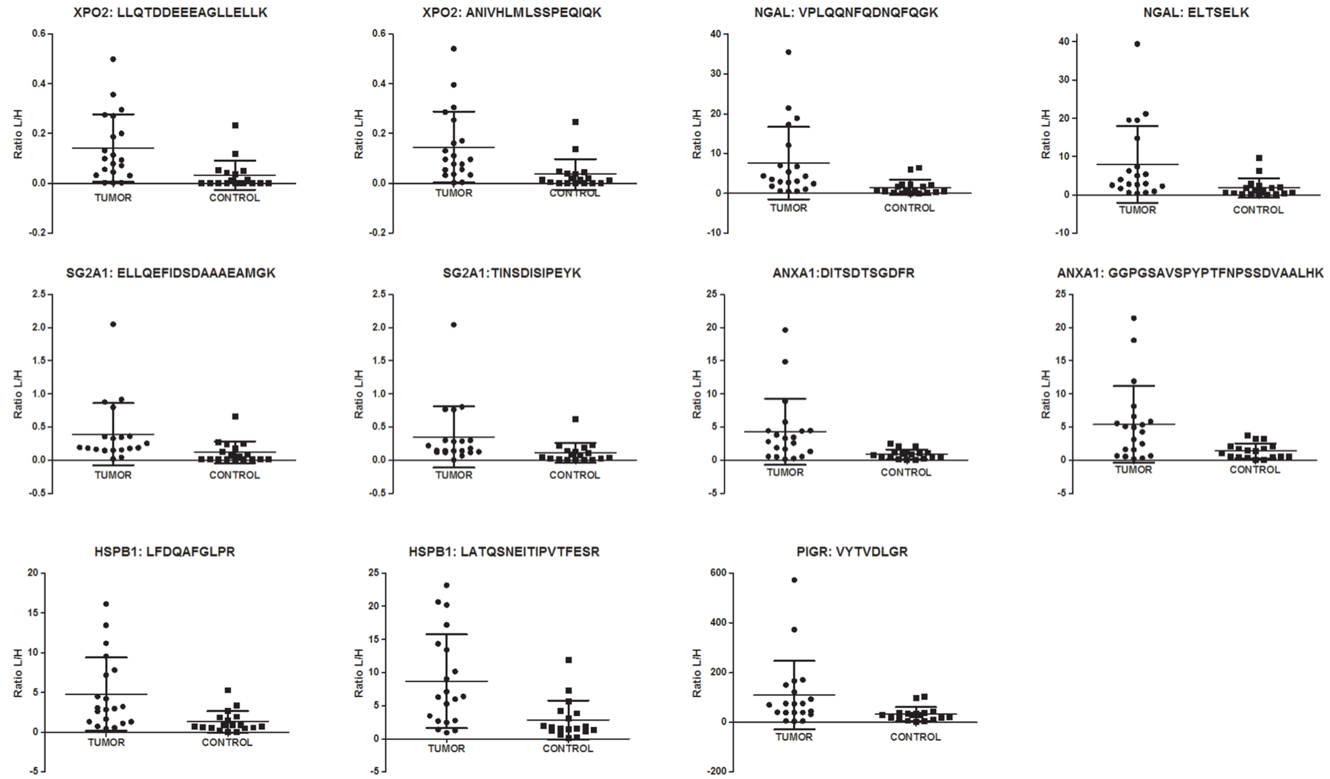

Supplementary Figure S3: Scattering plots of the expression of 31 peptides coming from the 16 differentially expressed biomarkers with AUC between 0.75-0.89. Scattering plots depicting the light/heavy (L/H) ratios across the 20 EC patients and 18 controls of the 16 differentially expressed biomarkers not shown in Figure 4 (*p*-value<0.05 and fold change higher than 3; AUC below 0.9).

Supplementary Table S1: List of proteins and related peptides detected by DDA analysis in uterine aspirates samples. In bold, the 158 proteins identified out of the initial list of 506 biomarker candidates. See Supplementary_File_S1

Supplementary Table S2: List of precursors and related fragment ions extracted from PRM acquisition. See Supplementary_File_S2

Supplementary Table S3: Raw integration data extracted from Skyline (area ratio L/H). See Supplementary_File_S3

Supplementary Table S4: Cleansed integration data, mean of duplicates (area ratio L/H). See Supplementary_File_S4

Supplementary Table S5: Clinico-pathological characteristics of the 42 women enrolled in the study. DDA, data-dependent acquisition; PRM, parallel-reaction monitoring. See Supplementary_File_S5
