# Supplementary material for: Development of a sequential workflow based on LC-PRM for the verification of endometrial cancer protein biomarkers in uterine aspirate samples
Source: Oncotarget. 2016 Jul 16;7(33):53102–15. doi: 10.18632/oncotarget.10632 (PMC5288171; doi:10.18632/oncotarget.10632)
Supplement: Supplementary file 2 [file oncotarget-07-53102-s002.docx]

**Supplementary Table 1.** List of proteins and related peptides detected by DDA analysis in uterine aspirates samples.

| **UNIPROT Accession number** | **Protein name** | **Peptides** |
| --- | --- | --- |
| **O00299** | **Chloride intraCellular Channel protein 1** | **YLSNAYAREEFASTCPDDEEIELAYEQVAK** |
| **O00299** | **Chloride intraCellular Channel protein 1** | **LAALNPESNTAGLDIFAK** |
| **O00299** | **Chloride intraCellular Channel protein 1** | **IGNCPFSQR** |
| **O00299** | **Chloride intraCellular Channel protein 1** | **NSNPALNDNLEK** |
| **O00299** | **Chloride intraCellular Channel protein 1** | **LHIVQVVCK** |
| **O00299** | **Chloride intraCellular Channel protein 1** | **YLSNAYAR** |
| **O00299** | **Chloride intraCellular Channel protein 1** | **GVTFNVTTVDTK** |
| **O00299** | **Chloride intraCellular Channel protein 1** | **FLDGNELTLADCNLLPK** |
| **O00299** | **Chloride intraCellular Channel protein 1** | **VLDNYLTSPLPEEVDETSAEDEGVSQRK** |
| **O00299** | **Chloride intraCellular Channel protein 1** | **GFTIPEAFR** |
| **O00299** | **Chloride intraCellular Channel protein 1** | **KFLDGNELTLADCNLLPK** |
| **O00299** | **Chloride intraCellular Channel protein 1** | **EEFASTCPDDEEIELAYEQVAK** |
| **O00299** | **Chloride intraCellular Channel protein 1** | **VLDNYLTSPLPEEVDETSAEDEGVSQR** |
| **O00299** | **Chloride intraCellular Channel protein 1** | **LHIVQVVCKK** |
| **O43278** | **Kunitz-type protease inhibitor 1** | **QTEDYCLASNK** |
| **O43278** | **Kunitz-type protease inhibitor 1** | **WYYDPTEQICK** |
| **O43490** | **Prominin-1** | **QLPPVDAELDNVNNVLR** |
| **O43490** | **Prominin-1** | **SPAGVNLLSFAYDLEAK** |
| **O43490** | **Prominin-1** | **MNYDSYLAQTGK** |
| **O43490** | **Prominin-1** | **TDLDGLVQQGYQSLNDIPDR** |
| **O43490** | **Prominin-1** | **AFTDLNSINSVLGGGILDR** |
| **O43490** | **Prominin-1** | **VNLNIFLLGAAGR** |
| **O43490** | **Prominin-1** | **LTFEQVYSDCK** |
| **O43490** | **Prominin-1** | **VLDTPYLLNEDWEYYLSGK** |
| **O43490** | **Prominin-1** | **VLPIEQSLSTLYQSVK** |
| **O43490** | **Prominin-1** | **SLHQQSTQLSSSLTSVK** |
| **O43490** | **Prominin-1** | **ATVFLLPALIFAVK** |
| **O43490** | **Prominin-1** | **QTTTVVAGIK** |
| **O43490** | **Prominin-1** | **LSLSQLNSNPELR** |
| **O43490** | **Prominin-1** | **TGNGLLER** |
| **O43490** | **Prominin-1** | **SMATAIKETK** |
| **O43490** | **Prominin-1** | **TDLDGLVQQGYQSLNDIPDRVQR** |
| **O43490** | **Prominin-1** | **NLQDFAACGIDR** |
| **O43490** | **Prominin-1** | **SSLNDPLCLVHPSSETCNSIR** |
| **O43490** | **Prominin-1** | **KLADSNFK** |
| **O43490** | **Prominin-1** | **TLLNETPEQIK** |
| **O43852** | **Calumenin** | **HLVYESDQNK** |
| **O75556** | **Mammaglobin-B** | **ELLQEFIDSDAAAEAMGK** |
| **O75556** | **Mammaglobin-B** | **TINSDISIPEYK** |
| **O94788** | **Retinal dehydrogenase 2** | **ANNSDFGLVAAVFTNDINK** |
| **O94788** | **Retinal dehydrogenase 2** | **GFFIEPTVFSNVTDDMR** |
| **O94788** | **Retinal dehydrogenase 2** | **IFINNEWQNSESGR** |
| **O94788** | **Retinal dehydrogenase 2** | **ILELIQSGVAEGAK** |
| **O94788** | **Retinal dehydrogenase 2** | **SPNIIFADADLDYAVEQAHQGVFFNQGQCCTAGSR** |
| **O94788** | **Retinal dehydrogenase 2** | **LAFSLGSVWR** |
| **O94788** | **Retinal dehydrogenase 2** | **IAFTGSTEVGK** |
| **O94788** | **Retinal dehydrogenase 2** | **IAPALCCGNTVVIKPAEQTPLSALYMGALIK** |
| **O94788** | **Retinal dehydrogenase 2** | **IHGMTIPVDGDYFTFTR** |
| **O94788** | **Retinal dehydrogenase 2** | **EAGFPPGVINILPGYGPTAGAAIASHIGIDK** |
| **O94788** | **Retinal dehydrogenase 2** | **LADLVER** |
| **O94788** | **Retinal dehydrogenase 2** | **TMDEVIER** |
| **O94788** | **Retinal dehydrogenase 2** | **LIQEAAGR** |
| **O94788** | **Retinal dehydrogenase 2** | **IAKEEIFGPVQEILR** |
| **O95994** | **Anterior gradient protein 2 homolog** | **GWGDQLIWTQTYEEALYK** |
| **P00338** | **L-laCtate dehydrogenase A Chain** | **ITVVGVGAVGMACAISILMK** |
| **P00338** | **L-laCtate dehydrogenase A Chain** | **DLADELALVDVIEDK** |
| **P00338** | **L-laCtate dehydrogenase A Chain** | **NRVIGSGCNLDSAR** |
| **P00338** | **L-laCtate dehydrogenase A Chain** | **DQLIYNLLKEEQTPQNK** |
| **P00338** | **L-laCtate dehydrogenase A Chain** | **GYTSWAIGLSVADLAESIMK** |
| **P00338** | **L-laCtate dehydrogenase A Chain** | **QVVESAYEVIK** |
| **P00338** | **L-laCtate dehydrogenase A Chain** | **LVIITAGAR** |
| **P00338** | **L-laCtate dehydrogenase A Chain** | **LGVHPLSCHGWVLGEHGDSSVPVWSGMNVAGVSLK** |
| **P00338** | **L-laCtate dehydrogenase A Chain** | **KSADTLWGIQK** |
| **P00338** | **L-laCtate dehydrogenase A Chain** | **SADTLWGIQK** |
| **P00338** | **L-laCtate dehydrogenase A Chain** | **DQLIYNLLK** |
| **P00338** | **L-laCtate dehydrogenase A Chain** | **DYNVTANSK** |
| **P00338** | **L-laCtate dehydrogenase A Chain** | **GLYGIKDDVFLSVPCILGQNGISDLVK** |
| **P00338** | **L-laCtate dehydrogenase A Chain** | **VTLTSEEEARLK** |
| **P00338** | **L-laCtate dehydrogenase A Chain** | **RVHPVSTMIK** |
| **P00338** | **L-laCtate dehydrogenase A Chain** | **FIIPNVVK** |
| **P00338** | **L-laCtate dehydrogenase A Chain** | **IVSGKDYNVTANSK** |
| **P00338** | **L-laCtate dehydrogenase A Chain** | **VTLTSEEEAR** |
| **P00338** | **L-laCtate dehydrogenase A Chain** | **NVNIFK** |
| **P00352** | **Retinal dehydrogenase 1** | **LYSNAYLNDLAGCIK** |
| **P00352** | **Retinal dehydrogenase 1** | **IFINNEWHDSVSGK** |
| **P00352** | **Retinal dehydrogenase 1** | **IGPALSCGNTVVVKPAEQTPLTALHVASLIK** |
| **P00352** | **Retinal dehydrogenase 1** | **VAFTGSTEVGK** |
| **P00352** | **Retinal dehydrogenase 1** | **IFVEESIYDEFVR** |
| **P00352** | **Retinal dehydrogenase 1** | **QAFQIGSPWR** |
| **P00352** | **Retinal dehydrogenase 1** | **EGAKLECGGGPWGNK** |
| **P00352** | **Retinal dehydrogenase 1** | **ELGEYGFHEYTEVK** |
| **P00352** | **Retinal dehydrogenase 1** | **KYILGNPLTPGVTQGPQIDK** |
| **P00352** | **Retinal dehydrogenase 1** | **RANNTFYGLSAGVFTK** |
| **P00352** | **Retinal dehydrogenase 1** | **TIPIDGNFFTYTR** |
| **P00352** | **Retinal dehydrogenase 1** | **HEPIGVCGQIIPWNFPLVMLIWK** |
| **P00352** | **Retinal dehydrogenase 1** | **EAGFPPGVVNIVPGYGPTAGAAISSHMDIDK** |
| **P00352** | **Retinal dehydrogenase 1** | **LECGGGPWGNK** |
| **P00352** | **Retinal dehydrogenase 1** | **SLDDVIK** |
| **P00352** | **Retinal dehydrogenase 1** | **AITISSALQAGTVWVNCYGVVSAQCPFGGFK** |
| **P00352** | **Retinal dehydrogenase 1** | **RVTLELGGK** |
| **P00352** | **Retinal dehydrogenase 1** | **GYFVQPTVFSNVTDEMR** |
| **P00352** | **Retinal dehydrogenase 1** | **ILDLIESGKK** |
| **P00352** | **Retinal dehydrogenase 1** | **IAKEEIFGPVQQIMK** |
| **P00352** | **Retinal dehydrogenase 1** | **LLLATMESMNGGK** |
| **P00352** | **Retinal dehydrogenase 1** | **FPVFNPATEEELCQVEEGDKEDVDK** |
| **P00352** | **Retinal dehydrogenase 1** | **LADLIERDR** |
| **P00352** | **Retinal dehydrogenase 1** | **YCAGWADK** |
| **P00441** | **Superoxide dismutase [Cu-Zn]** | **HVGDLGNVTADKDGVADVSIEDSVISLSGDHCIIGR** |
| **P00441** | **Superoxide dismutase [Cu-Zn]** | **GDGPVQGIINFEQK** |
| **P00441** | **Superoxide dismutase [Cu-Zn]** | **ADDLGKGGNEESTK** |
| **P00441** | **Superoxide dismutase [Cu-Zn]** | **AVCVLKGDGPVQGIINFEQK** |
| **P00441** | **Superoxide dismutase [Cu-Zn]** | **DGVADVSIEDSVISLSGDHCIIGR** |
| **P00441** | **Superoxide dismutase [Cu-Zn]** | **GLTEGLHGFHVHEFGDNTAGCTSAGPHFNPLSRK** |
| **P00441** | **Superoxide dismutase [Cu-Zn]** | **GLTEGLHGFHVHEFGDNTAGCTSAGPHFNPLSR** |
| **P00441** | **Superoxide dismutase [Cu-Zn]** | **AVCVLK** |
| **P00441** | **Superoxide dismutase [Cu-Zn]** | **HVGDLGNVTADK** |
| **P00441** | **Superoxide dismutase [Cu-Zn]** | **TLVVHEK** |
| **P00450** | **Ceruloplasmin** | **AGLQAFFQVQECNK** |
| **P00450** | **Ceruloplasmin** | **KLISVDTEHSNIYLQNGPDR** |
| **P00450** | **Ceruloplasmin** | **KAEEEHLGILGPQLHADVGDK** |
| **P00450** | **Ceruloplasmin** | **HRGVYSSDVFDIFPGTYQTLEMFPR** |
| **P00450** | **Ceruloplasmin** | **EFYLFPTVFDENESLLLEDNIR** |
| **P00450** | **Ceruloplasmin** | **VNKDDEEFIESNK** |
| **P00450** | **Ceruloplasmin** | **ALYLQYTDETFR** |
| **P00450** | **Ceruloplasmin** | **MYSVNGYTFGSLPGLSMCAEDRVK** |
| **P00450** | **Ceruloplasmin** | **EVGPTNADPVCLAK** |
| **P00450** | **Ceruloplasmin** | **NYRIDTINLFPATLFDAYMVAQNPGEWMLSCQNLNHLK** |
| **P00450** | **Ceruloplasmin** | **HYYIGIIETTWDYASDHGEK** |
| **P00450** | **Ceruloplasmin** | **DLYSGLIGPLIVCR** |
| **P00450** | **Ceruloplasmin** | **NNEGTYYSPNYNPQSR** |
| **P00450** | **Ceruloplasmin** | **DIASGLIGPLIICK** |
| **P00450** | **Ceruloplasmin** | **SGAGTEDSACIPWAYYSTVDQVK** |
| **P00450** | **Ceruloplasmin** | **mYYSAVDPTK** |
| **P00450** | **Ceruloplasmin** | **DIFTGLIGPMK** |
| **P00450** | **Ceruloplasmin** | **TTIEKPVWLGFLGPIIK** |
| **P00450** | **Ceruloplasmin** | **NLASRPYTFHSHGITYYK** |
| **P00450** | **Ceruloplasmin** | **GAYPLSIEPIGVR** |
| **P00450** | **Ceruloplasmin** | **MFTTAPDQVDKEDEDFQESNK** |
| **P00450** | **Ceruloplasmin** | **DIASGLIGPLIICKK** |
| **P00450** | **Ceruloplasmin** | **RQSEDSTFYLGER** |
| **P00450** | **Ceruloplasmin** | **GDSVVWYLFSAGNEADVHGIYFSGNTYLWR** |
| **P00450** | **Ceruloplasmin** | **VYVHLK** |
| **P00450** | **Ceruloplasmin** | **IPERSGAGTEDSACIPWAYYSTVDQVK** |
| **P00450** | **Ceruloplasmin** | **EYTDASFTNRK** |
| **P00450** | **Ceruloplasmin** | **IDTINLFPATLFDAYMVAQNPGEWMLSCQNLNHLK** |
| **P00450** | **Ceruloplasmin** | **ERGPEEEHLGILGPVIWAEVGDTIR** |
| **P00450** | **Ceruloplasmin** | **MHSMNGFMYGNQPGLTMCK** |
| **P00450** | **Ceruloplasmin** | **MYSVNGYTFGSLPGLSMCAEDR** |
| **P00450** | **Ceruloplasmin** | **QSEDSTFYLGER** |
| **P00450** | **Ceruloplasmin** | **IYHSHIDAPK** |
| **P00450** | **Ceruloplasmin** | **QKYTVNQCR** |
| **P00450** | **Ceruloplasmin** | **LISVDTEHSNIYLQNGPDR** |
| **P00450** | **Ceruloplasmin** | **MYYSAVDPTKDIFTGLIGPMK** |
| **P00450** | **Ceruloplasmin** | **TYSDHPEK** |
| **P00450** | **Ceruloplasmin** | **YTVNQCR** |
| **P00450** | **Ceruloplasmin** | **EYTDASFTNR** |
| **P00450** | **Ceruloplasmin** | **MHAINGR** |
| **P00450** | **Ceruloplasmin** | **GVYSSDVFDIFPGTYQTLEMFPR** |
| **P00450** | **Ceruloplasmin** | **VYPGEQYTYMLLATEEQSPGEGDGNCVTR** |
| **P00450** | **Ceruloplasmin** | **NMATRPYSIHAHGVQTESSTVTPTLPGETLTYVWK** |
| **P00450** | **Ceruloplasmin** | **GPEEEHLGILGPVIWAEVGDTIR** |
| **P00450** | **Ceruloplasmin** | **DVDKEFYLFPTVFDENESLLLEDNIR** |
| **P00450** | **Ceruloplasmin** | **DTANLFPQTSLTLHMWPDTEGTFNVECLTTDHYTGGMK** |
| **P00738** | **Haptoglobin** | **YQEDTCYGDAGSAFAVHDLEEDTWYATGILSFDK** |
| **P00738** | **Haptoglobin** | **LRTEGDGVYTLNNEK** |
| **P00738** | **Haptoglobin** | **AVGDKLPECEADDGCPKPPEIAHGYVEHSVR** |
| **P00738** | **Haptoglobin** | **YVMLPVADQDQCIR** |
| **P00738** | **Haptoglobin** | **VTSIQDWVQK** |
| **P00738** | **Haptoglobin** | **VGYVSGWGR** |
| **P00738** | **Haptoglobin** | **TEGDGVYTLNNEK** |
| **P00738** | **Haptoglobin** | **SPVGVQPILNEHTFCAGMSK** |
| **P00738** | **Haptoglobin** | **VVLHPNYSQVDIGLIK** |
| **P00738** | **Haptoglobin** | **FTDHLK** |
| **P00738** | **Haptoglobin** | **HYEGSTVPEKK** |
| **P00738** | **Haptoglobin** | **HYEGSTVPEK** |
| **P00738** | **Haptoglobin** | **VMPICLPSKDYAEVGR** |
| **P00738** | **Haptoglobin** | **QKVSVNER** |
| **P00918** | **CarboniC anhydrase 2** | **AVQQPDGLAVLGIFLK** |
| **P00918** | **CarboniC anhydrase 2** | **VVDVLDSIK** |
| **P00918** | **CarboniC anhydrase 2** | **SADFTNFDPR** |
| **P00918** | **CarboniC anhydrase 2** | **VGSAKPGLQK** |
| **P00918** | **CarboniC anhydrase 2** | **YDPSLKPLSVSYDQATSLR** |
| **P00918** | **CarboniC anhydrase 2** | **ILNNGHAFNVEFDDSQDK** |
| **P00918** | **CarboniC anhydrase 2** | **QSPVDIDTHTAK** |
| **P00918** | **CarboniC anhydrase 2** | **YAAELHLVHWNTK** |
| **P00918** | **CarboniC anhydrase 2** | **LNFNGEGEPEELMVDNWRPAQPLK** |
| **P01008** | **Antithrombin-III** | **ADGESCSASMMYQEGK** |
| **P01008** | **Antithrombin-III** | **EQLQDMGLVDLFSPEK** |
| **P01008** | **Antithrombin-III** | **ITDVIPSEAINELTVLVLVNTIYFK** |
| **P01008** | **Antithrombin-III** | **FRIEDGFSLK** |
| **P01008** | **Antithrombin-III** | **EVPLNTIIFMGR** |
| **P01008** | **Antithrombin-III** | **ELFYKADGESCSASMMYQEGK** |
| **P01008** | **Antithrombin-III** | **LVSANRLFGDK** |
| **P01008** | **Antithrombin-III** | **ANRPFLVFIR** |
| **P01008** | **Antithrombin-III** | **LQPLDFKENAEQSR** |
| **P01008** | **Antithrombin-III** | **FATTFYQHLADSKNDNDNIFLSPLSISTAFAMTK** |
| **P01008** | **Antithrombin-III** | **VWELSK** |
| **P01008** | **Antithrombin-III** | **ATEDEGSEQKIPEATNR** |
| **P01008** | **Antithrombin-III** | **NDNDNIFLSPLSISTAFAMTK** |
| **P01008** | **Antithrombin-III** | **AFLEVNEEGSEAAASTAVVIAGR** |
| **P01009** | **Alpha-1-antitrypsin** | **VFSNGADLSGVTEEAPLK** |
| **P01009** | **Alpha-1-antitrypsin** | **TLNQPDSQLQLTTGNGLFLSEGLK** |
| **P01009** | **Alpha-1-antitrypsin** | **DTEEEDFHVDQVTTVK** |
| **P01009** | **Alpha-1-antitrypsin** | **ITPNLAEFAFSLYR** |
| **P01009** | **Alpha-1-antitrypsin** | **LYHSEAFTVNFGDTEEAKK** |
| **P01009** | **Alpha-1-antitrypsin** | **ELDRDTVFALVNYIFFK** |
| **P01009** | **Alpha-1-antitrypsin** | **LQHLENELTHDIITK** |
| **P01009** | **Alpha-1-antitrypsin** | **SASLHLPK** |
| **P01009** | **Alpha-1-antitrypsin** | **LSITGTYDLK** |
| **P01009** | **Alpha-1-antitrypsin** | **SVLGQLGITK** |
| **P01009** | **Alpha-1-antitrypsin** | **LSSWVLLMK** |
| **P01009** | **Alpha-1-antitrypsin** | **FNKPFVFLMIEQNTK** |
| **P01009** | **Alpha-1-antitrypsin** | **KLSSWVLLMK** |
| **P01009** | **Alpha-1-antitrypsin** | **GTEAAGAMFLEAIPMSIPPEVK** |
| **P01009** | **Alpha-1-antitrypsin** | **IVDLVK** |
| **P01009** | **Alpha-1-antitrypsin** | **RLGMFNIQHCK** |
| **P01009** | **Alpha-1-antitrypsin** | **AVLTIDEK** |
| **P01009** | **Alpha-1-antitrypsin** | **SPLFMGK** |
| **P01009** | **Alpha-1-antitrypsin** | **TDTSHHDQDHPTFNK** |
| **P01009** | **Alpha-1-antitrypsin** | **VPMMK** |
| **P01009** | **Alpha-1-antitrypsin** | **RSASLHLPK** |
| **P01009** | **Alpha-1-antitrypsin** | **FLEDVKK** |
| **P01009** | **Alpha-1-antitrypsin** | **FLENEDRR** |
| **P01009** | **Alpha-1-antitrypsin** | **KQINDYVEK** |
| **P01009** | **Alpha-1-antitrypsin** | **LVDKFLEDVK** |
| **P01009** | **Alpha-1-antitrypsin** | **TLNQPDSQLQLTTGNGLFLSEGLKLVDK** |
| **P01009** | **Alpha-1-antitrypsin** | **LYHSEAFTVNFGDTEEAK** |
| **P01009** | **Alpha-1-antitrypsin** | **LGMFNIQHCKK** |
| **P01009** | **Alpha-1-antitrypsin** | **LGMFNIQHCK** |
| **P01009** | **Alpha-1-antitrypsin** | **AVLTIDEKGTEAAGAMFLEAIPMSIPPEVK** |
| **P01009** | **Alpha-1-antitrypsin** | **VVNPTQK** |
| **P01009** | **Alpha-1-antitrypsin** | **WERPFEVKDTEEEDFHVDQVTTVK** |
| **P01009** | **Alpha-1-antitrypsin** | **FLENEDR** |
| **P01009** | **Alpha-1-antitrypsin** | **FIEDVK** |
| **P01009** | **Alpha-1-antitrypsin** | **QINDYVEK** |
| **P01023** | **Alpha-2-maCroglobulin** | **VSNQTLSLFFTVLQDVPVR** |
| **P01023** | **Alpha-2-maCroglobulin** | **VSVQLEASPAFLAVPVEK** |
| **P01023** | **Alpha-2-maCroglobulin** | **VTGEGCVYLQTSLK** |
| **P01023** | **Alpha-2-maCroglobulin** | **SSSNEEVMFLTVQVK** |
| **P01023** | **Alpha-2-maCroglobulin** | **ALLAYAFALAGNQDK** |
| **P01023** | **Alpha-2-maCroglobulin** | **KYSDASDCHGEDSQAFCEK** |
| **P01023** | **Alpha-2-maCroglobulin** | **VDLSFSPSQSLPASHAHLR** |
| **P01023** | **Alpha-2-maCroglobulin** | **TEVSSNHVLIYLDK** |
| **P01023** | **Alpha-2-maCroglobulin** | **NEDSLVFVQTDK** |
| **P01023** | **Alpha-2-maCroglobulin** | **IAQWQSFQLEGGLK** |
| **P01023** | **Alpha-2-maCroglobulin** | **MCPQLQQYEMHGPEGLR** |
| **P01023** | **Alpha-2-maCroglobulin** | **VYDYYETDEFAIAEYNAPCSK** |
| **P01023** | **Alpha-2-maCroglobulin** | **VGFYESDVMGR** |
| **P01023** | **Alpha-2-maCroglobulin** | **LHTEAQIQEEGTVVELTGR** |
| **P01023** | **Alpha-2-maCroglobulin** | **KLSFYYLIMAK** |
| **P01023** | **Alpha-2-maCroglobulin** | **AAQVTIQSSGTFSSK** |
| **P01023** | **Alpha-2-maCroglobulin** | **QFSFPLSSEPFQGSYK** |
| **P01023** | **Alpha-2-maCroglobulin** | **YDVENCLANK** |
| **P01023** | **Alpha-2-maCroglobulin** | **DmYSFLEDMGLK** |
| **P01023** | **Alpha-2-maCroglobulin** | **TEHPFTVEEFVLPK** |
| **P01023** | **Alpha-2-maCroglobulin** | **YSDASDCHGEDSQAFCEK** |
| **P01023** | **Alpha-2-maCroglobulin** | **VSNQTLSLFFTVLQDVPVRDLKPAIVK** |
| **P01023** | **Alpha-2-maCroglobulin** | **FEVQVTVPK** |
| **P01023** | **Alpha-2-maCroglobulin** | **GGVEDEVTLSAYITIALLEIPLTVTHPVVR** |
| **P01023** | **Alpha-2-maCroglobulin** | **DTVIKPLLVEPEGLEK** |
| **P01023** | **Alpha-2-maCroglobulin** | **GHFSISIPVKSDIAPVAR** |
| **P01023** | **Alpha-2-maCroglobulin** | **LLLQQVSLPELPGEYSMK** |
| **P01023** | **Alpha-2-maCroglobulin** | **LSFYYLIMAK** |
| **P01023** | **Alpha-2-maCroglobulin** | **SLFTDLEAENDVLHCVAFAVPK** |
| **P01023** | **Alpha-2-maCroglobulin** | **TAQEGDHGSHVYTK** |
| **P01023** | **Alpha-2-maCroglobulin** | **FSGQLNSHGCFYQQVK** |
| **P01023** | **Alpha-2-maCroglobulin** | **SASNMAIVDVK** |
| **P01023** | **Alpha-2-maCroglobulin** | **NALFCLESAWK** |
| **P01023** | **Alpha-2-maCroglobulin** | **YNILPEKEEFPFALGVQTLPQTCDEPK** |
| **P01023** | **Alpha-2-maCroglobulin** | **EQAPHCICANGR** |
| **P01023** | **Alpha-2-maCroglobulin** | **TEHPFTVEEFVLPKFEVQVTVPK** |
| **P01023** | **Alpha-2-maCroglobulin** | **FRVVSMDENFHPLNELIPLVYIQDPK** |
| **P01023** | **Alpha-2-maCroglobulin** | **VTAAPQSVCALR** |
| **P01023** | **Alpha-2-maCroglobulin** | **LLIYAVLPTGDVIGDSAK** |
| **P01023** | **Alpha-2-maCroglobulin** | **ETTFNSLLCPSGGEVSEELSLKLPPNVVEESAR** |
| **P01023** | **Alpha-2-maCroglobulin** | **LPPNVVEESAR** |
| **P01023** | **Alpha-2-maCroglobulin** | **QQNAQGGFSSTQDTVVALHALSK** |
| **P01023** | **Alpha-2-maCroglobulin** | **QTVSWAVTPK** |
| **P01023** | **Alpha-2-maCroglobulin** | **AIGYLNTGYQR** |
| **P01023** | **Alpha-2-maCroglobulin** | **GPTQEFK** |
| **P01023** | **Alpha-2-maCroglobulin** | **EEFPFALGVQTLPQTCDEPK** |
| **P01023** | **Alpha-2-maCroglobulin** | **VVSMDENFHPLNELIPLVYIQDPK** |
| **P01023** | **Alpha-2-maCroglobulin** | **SGGRTEHPFTVEEFVLPK** |
| **P01023** | **Alpha-2-maCroglobulin** | **FQVDNNNR** |
| **P01023** | **Alpha-2-maCroglobulin** | **SLNEEAVKK** |
| **P01023** | **Alpha-2-maCroglobulin** | **KYFPETWIWDLVVVNSAGVAEVGVTVPDTITEWK** |
| **P01023** | **Alpha-2-maCroglobulin** | **QGIPFFGQVR** |
| **P01023** | **Alpha-2-maCroglobulin** | **GHFSISIPVK** |
| **P01023** | **Alpha-2-maCroglobulin** | **DRSPCYGYQWVSEEHEEAHHTAYLVFSPSK** |
| **P01023** | **Alpha-2-maCroglobulin** | **ETTFNSLLCPSGGEVSEELSLK** |
| **P01023** | **Alpha-2-maCroglobulin** | **SSGSLLNNAIKGGVEDEVTLSAYITIALLEIPLTVTHPVVR** |
| **P01023** | **Alpha-2-maCroglobulin** | **FQVDNNNRLLLQQVSLPELPGEYSMK** |
| **P01023** | **Alpha-2-maCroglobulin** | **SFVHLEPMSHELPCGHTQTVQAHYILNGGTLLGLK** |
| **P01023** | **Alpha-2-maCroglobulin** | **VIFIR** |
| **P01023** | **Alpha-2-maCroglobulin** | **KDTVIKPLLVEPEGLEK** |
| **P01023** | **Alpha-2-maCroglobulin** | **NQGNTWLTAFVLK** |
| **P01023** | **Alpha-2-maCroglobulin** | **AFQPFFVELTMPYSVIR** |
| **P01023** | **Alpha-2-maCroglobulin** | **HYDGSYSTFGER** |
| **P01023** | **Alpha-2-maCroglobulin** | **ATVLNYLPK** |
| **P01023** | **Alpha-2-maCroglobulin** | **SDIAPVAR** |
| **P01023** | **Alpha-2-maCroglobulin** | **TGTHGLLVKQEDMK** |
| **P01023** | **Alpha-2-maCroglobulin** | **LVHVEEPHTETVR** |
| **P01023** | **Alpha-2-maCroglobulin** | **QSSEITR** |
| **P01023** | **Alpha-2-maCroglobulin** | **GPTQEFKK** |
| **P01023** | **Alpha-2-maCroglobulin** | **AVDQSVLLMKPDAELSASSVYNLLPEK** |
| **P01023** | **Alpha-2-maCroglobulin** | **DNSVHWERPQKPK** |
| **P01023** | **Alpha-2-maCroglobulin** | **DLKPAIVK** |
| **P01023** | **Alpha-2-maCroglobulin** | **HNVYINGITYTPVSSTNEK** |
| **P01023** | **Alpha-2-maCroglobulin** | **YDVENCLANKVDLSFSPSQSLPASHAHLR** |
| **P01023** | **Alpha-2-maCroglobulin** | **AGAFCLSEDAGLGISSTASLR** |
| **P01023** | **Alpha-2-maCroglobulin** | **SSGSLLNNAIK** |
| **P01023** | **Alpha-2-maCroglobulin** | **YGAATFTR** |
| **P01023** | **Alpha-2-maCroglobulin** | **TGTHGLLVK** |
| **P01023** | **Alpha-2-maCroglobulin** | **LVHVEEPHTETVRK** |
| **P01023** | **Alpha-2-maCroglobulin** | **TFAQAR** |
| **P01023** | **Alpha-2-maCroglobulin** | **MVSGFIPLKPTVK** |
| **P01023** | **Alpha-2-maCroglobulin** | **RTTVMVK** |
| **P01024** | **Complement C3** | **DYAGVFSDAGLTFTSSSGQQTAQR** |
| **P01024** | **Complement C3** | **QLYNVEATSYALLALLQLK** |
| **P01024** | **Complement C3** | **VQLSNDFDEYImAIEQTIK** |
| **P01024** | **Complement C3** | **DSITTWEILAVSMSDKK** |
| **P01024** | **Complement C3** | **LVAYYTLIGASGQR** |
| **P01024** | **Complement C3** | **GICVADPFEVTVMQDFFIDLR** |
| **P01024** | **Complement C3** | **VFLDCCNYITELR** |
| **P01024** | **Complement C3** | **VLLDGVQNPR** |
| **P01024** | **Complement C3** | **IPIEDGSGEVVLSR** |
| **P01024** | **Complement C3** | **DTWVEHWPEEDECQDEENQK** |
| **P01024** | **Complement C3** | **DICEEQVNSLPGSITK** |
| **P01024** | **Complement C3** | **ILLQGTPVAQMTEDAVDAER** |
| **P01024** | **Complement C3** | **FVTVQATFGTQVVEK** |
| **P01024** | **Complement C3** | **SEETKENEGFTVTAEGK** |
| **P01024** | **Complement C3** | **TMQALPYSTVGNSNNYLHLSVLR** |
| **P01024** | **Complement C3** | **KQELSEAEQATR** |
| **P01024** | **Complement C3** | **SNLDEDIIAEENIVSR** |
| **P01024** | **Complement C3** | **VVLVSLQSGYLFIQTDK** |
| **P01024** | **Complement C3** | **SGQSEDRQPVPGQQMTLK** |
| **P01024** | **Complement C3** | **VHQYFNVELIQPGAVK** |
| **P01024** | **Complement C3** | **RIPIEDGSGEVVLSR** |
| **P01024** | **Complement C3** | **VSHSEDDCLAFK** |
| **P01024** | **Complement C3** | **TGLQEVEVK** |
| **P01024** | **Complement C3** | **SGSDEVQVGQQR** |
| **P01024** | **Complement C3** | **VLLDGVQNPRAEDLVGK** |
| **P01024** | **Complement C3** | **AGDFLEANYMNLQR** |
| **P01024** | **Complement C3** | **SYTVAIAGYALAQMGR** |
| **P01024** | **Complement C3** | **YYGGGYGSTQATFMVFQALAQYQK** |
| **P01024** | **Complement C3** | **LESEETMVLEAHDAQGDVPVTVTVHDFPGK** |
| **P01024** | **Complement C3** | **EYVLPSFEVIVEPTEK** |
| **P01024** | **Complement C3** | **VPVAVQGEDTVQSLTQGDGVAK** |
| **P01024** | **Complement C3** | **VRVELLHNPAFCSLATTK** |
| **P01024** | **Complement C3** | **NTMILEICTR** |
| **P01024** | **Complement C3** | **TRFISLGEACK** |
| **P01024** | **Complement C3** | **GQGTLSVVTMYHAK** |
| **P01024** | **Complement C3** | **EPGQDLVVLPLSITTDFIPSFR** |
| **P01024** | **Complement C3** | **CAEENCFIQK** |
| **P01024** | **Complement C3** | **SSLSVPYVIVPLK** |
| **P01024** | **Complement C3** | **VYAYYNLEESCTR** |
| **P01024** | **Complement C3** | **ENEGFTVTAEGK** |
| **P01024** | **Complement C3** | **YYTYLIMNK** |
| **P01024** | **Complement C3** | **SLYVSATVILHSGSDMVQAER** |
| **P01024** | **Complement C3** | **DMALTAFVLISLQEAK** |
| **P01024** | **Complement C3** | **AYYENSPQQVFSTEFEVK** |
| **P01024** | **Complement C3** | **TELRPGETLNVNFLLR** |
| **P01024** | **Complement C3** | **GYTQQLAFRQPSSAFAAFVK** |
| **P01024** | **Complement C3** | **GYTQQLAFR** |
| **P01024** | **Complement C3** | **KVEGTAFVIFGIQDGEQR** |
| **P01024** | **Complement C3** | **GICVADPFEVTVMQDFFIDLRLPYSVVR** |
| **P01024** | **Complement C3** | **QVREPGQDLVVLPLSITTDFIPSFR** |
| **P01024** | **Complement C3** | **DQLTCNKFDLK** |
| **P01024** | **Complement C3** | **EVVADSVWVDVK** |
| **P01024** | **Complement C3** | **SDDKVTLEER** |
| **P01024** | **Complement C3** | **TVMVNIENPEGIPVK** |
| **P01024** | **Complement C3** | **EGVQKEDIPPADLSDQVPDTESETR** |
| **P01024** | **Complement C3** | **SGIPIVTSPYQIHFTK** |
| **P01024** | **Complement C3** | **KGYTQQLAFR** |
| **P01024** | **Complement C3** | **QLANGVDR** |
| **P01024** | **Complement C3** | **QCQDLGAFTESMVVFGCPN** |
| **P01024** | **Complement C3** | **IRAYYENSPQQVFSTEFEVK** |
| **P01024** | **Complement C3** | **AVLYNYRQNQELK** |
| **P01024** | **Complement C3** | **DFDFVPPVVR** |
| **P01024** | **Complement C3** | **NEQVEIR** |
| **P01024** | **Complement C3** | **RQGALELIK** |
| **P01024** | **Complement C3** | **QLYNVEATSYALLALLQLKDFDFVPPVVR** |
| **P01024** | **Complement C3** | **DSCVGSLVVK** |
| **P01024** | **Complement C3** | **LMNIFLK** |
| **P01024** | **Complement C3** | **ACEPGVDYVYK** |
| **P01024** | **Complement C3** | **GVFVLNKK** |
| **P01024** | **Complement C3** | **VTIKPAPETEK** |
| **P01024** | **Complement C3** | **AFSDRNTLIIYLDK** |
| **P01024** | **Complement C3** | **LPYSVVR** |
| **P01024** | **Complement C3** | **ADIGCTPGSGK** |
| **P01024** | **Complement C3** | **GPLLNK** |
| **P01024** | **Complement C3** | **IRYYTYLIMNK** |
| **P01024** | **Complement C3** | **VVLVAVDK** |
| **P01024** | **Complement C3** | **VELLHNPAFCSLATTK** |
| **P01024** | **Complement C3** | **AEDLVGK** |
| **P01024** | **Complement C3** | **DAPDHQELNLDVSLQLPSR** |
| **P01024** | **Complement C3** | **FLTTAK** |
| **P01024** | **Complement C3** | **AAVYHHFISDGVRK** |
| **P01024** | **Complement C3** | **YFKPGMPFDLMVFVTNPDGSPAYR** |
| **P01024** | **Complement C3** | **HLIVTPSGCGEQNMIGMTPTVIAVHYLDETEQWEK** |
| **P01024** | **Complement C3** | **LSINTHPSQKPLSITVR** |
| **P01024** | **Complement C3** | **RAPSTWLTAYVVK** |
| **P01024** | **Complement C3** | **ADIGCTPGSGKDYAGVFSDAGLTFTSSSGQQTAQR** |
| **P01024** | **Complement C3** | **TFISPIK** |
| **P01024** | **Complement C3** | **CCEDGMRENPMR** |
| **P01024** | **Complement C3** | **DSITTWEILAVSMSDK** |
| **P01024** | **Complement C3** | **GLEVTITAR** |
| **P01024** | **Complement C3** | **GVFVLNK** |
| **P01024** | **Complement C3** | **NTLIIYLDK** |
| **P01024** | **Complement C3** | **APSTWLTAYVVK** |
| **P01024** | **Complement C3** | **IPIEDGSGEVVLSRK** |
| **P01024** | **Complement C3** | **QKPDGVFQEDAPVIHQEMIGGLR** |
| **P01024** | **Complement C3** | **QELSEAEQATR** |
| **P01024** | **Complement C3** | **ILLQGTPVAQMTEDAVDAERLK** |
| **P01024** | **Complement C3** | **KVLLDGVQNPR** |
| **P01024** | **Complement C3** | **AKDQLTCNK** |
| **P01024** | **Complement C3** | **QGALELIKK** |
| **P01024** | **Complement C3** | **AAVYHHFISDGVR** |
| **P01024** | **Complement C3** | **VTIKPAPETEKRPQDAK** |
| **P01024** | **Complement C3** | **ASHLGLAR** |
| **P01024** | **Complement C3** | **IHWESASLLR** |
| **P01024** | **Complement C3** | **QGALELIK** |
| **P01024** | **Complement C3** | **ISLPESLKR** |
| **P01024** | **Complement C3** | **EALKLEEK** |
| **P01024** | **Complement C3** | **VGKYPK** |
| **P01024** | **Complement C3** | **FGLEKR** |
| **P01024** | **Complement C3** | **FYHPEKEDGK** |
| **P01024** | **Complement C3** | **YELDK** |
| **P01024** | **Complement C3** | **LKGPLLNK** |
| **P01024** | **Complement C3** | **LESEETMVLEAHDAQGDVPVTVTVHDFPGKK** |
| **P01024** | **Complement C3** | **VFSLAVNLIAIDSQVLCGAVK** |
| **P01024** | **Complement C3** | **EVVADSVWVDVKDSCVGSLVVK** |
| **P01024** | **Complement C3** | **WLNEQR** |
| **P01024** | **Complement C3** | **YISKYELDK** |
| **P01024** | **Complement C3** | **YRGDQDATMSILDISMMTGFAPDTDDLK** |
| **P01024** | **Complement C3** | **KLVLSSEK** |
| **P01033** | **Metalloproteinase inhibitor 1** | **GFQALGDAADIR** |
| **P01033** | **Metalloproteinase inhibitor 1** | **LQDGLLHITTCSFVAPWNSLSLAQR** |
| **P01033** | **Metalloproteinase inhibitor 1** | **LQSGTHCLWTDQLLQGSEK** |
| **P01042** | **Kininogen-1** | **SLWNGDTGECTDNAYIDIQLR** |
| **P01042** | **Kininogen-1** | **KYNSQNQSNNQFVLYR** |
| **P01042** | **Kininogen-1** | **AATGECTATVGK** |
| **P01042** | **Kininogen-1** | **YNSQNQSNNQFVLYR** |
| **P01042** | **Kininogen-1** | **KIYPTVNCQPLGMISLMK** |
| **P01042** | **Kininogen-1** | **IASFSQNCDIYPGK** |
| **P01042** | **Kininogen-1** | **YFIDFVAR** |
| **P01042** | **Kininogen-1** | **KLGQSLDCNAEVYVVPWEK** |
| **P01042** | **Kininogen-1** | **TVGSDTFYSFKYEIK** |
| **P01042** | **Kininogen-1** | **QVVAGLNFR** |
| **P01042** | **Kininogen-1** | **DIPTNSPELEETLTHTITK** |
| **P01042** | **Kininogen-1** | **ENFLFLTPDCK** |
| **P01042** | **Kininogen-1** | **ESNEELTESCETK** |
| **P01042** | **Kininogen-1** | **TWQDCEYK** |
| **P01042** | **Kininogen-1** | **TVGSDTFYSFK** |
| **P01042** | **Kininogen-1** | **ETTCSKESNEELTESCETK** |
| **P01042** | **Kininogen-1** | **LGQSLDCNAEVYVVPWEK** |
| **P01042** | **Kininogen-1** | **FSVATQTCQITPAEGPVVTAQYDCLGCVHPISTQSPDLEPILR** |
| **P01042** | **Kininogen-1** | **AATGECTATVGKR** |
| **P01042** | **Kininogen-1** | **IYPTVNCQPLGMISLMK** |
| **P01042** | **Kininogen-1** | **IASFSQNCDIYPGKDFVQPPTK** |
| **P01042** | **Kininogen-1** | **TWQDCEYKDAAK** |
| **P01042** | **Kininogen-1** | **VQVVAGKK** |
| **P01042** | **Kininogen-1** | **AVDAALKK** |
| **P01042** | **Kininogen-1** | **EGDCPVQSGK** |
| **P01042** | **Kininogen-1** | **YEIKEGDCPVQSGK** |
| **P01042** | **Kininogen-1** | **VQVVAGK** |
| **P01042** | **Kininogen-1** | **RPPGFSPFR** |
| **P01833** | **PolymeriC immunoglobulin reCeptor** | **ASVDSGSSEEQGGSSR** |
| **P01833** | **PolymeriC immunoglobulin reCeptor** | **GGCITLISSEGYVSSK** |
| **P01833** | **PolymeriC immunoglobulin reCeptor** | **DAGFYWCLTNGDTLWR** |
| **P01833** | **PolymeriC immunoglobulin reCeptor** | **LDIQGTGQLLFSVVINQLR** |
| **P01833** | **PolymeriC immunoglobulin reCeptor** | **WNNTGCQALPSQDEGPSK** |
| **P01833** | **PolymeriC immunoglobulin reCeptor** | **DGSFSVVITGLR** |
| **P01833** | **PolymeriC immunoglobulin reCeptor** | **QSSGENCDVVVNTLGK** |
| **P01833** | **PolymeriC immunoglobulin reCeptor** | **QGHFYGETAAVYVAVEERK** |
| **P01833** | **PolymeriC immunoglobulin reCeptor** | **LSDAGQYLCQAGDDSNSNKK** |
| **P01833** | **PolymeriC immunoglobulin reCeptor** | **TVTINCPFKTENAQK** |
| **P01833** | **PolymeriC immunoglobulin reCeptor** | **NADLQVLKPEPELVYEDLR** |
| **P01833** | **PolymeriC immunoglobulin reCeptor** | **YWCLWEGAQNGR** |
| **P01833** | **PolymeriC immunoglobulin reCeptor** | **GVAGGSVAVLCPYNRK** |
| **P01833** | **PolymeriC immunoglobulin reCeptor** | **ADEGWYWCGVK** |
| **P01833** | **PolymeriC immunoglobulin reCeptor** | **YLCGAHSDGQLQEGSPIQAWQLFVNEESTIPRSPTVVK** |
| **P01833** | **PolymeriC immunoglobulin reCeptor** | **VLDSGFREIENK** |
| **P01833** | **PolymeriC immunoglobulin reCeptor** | **ALVSTLVPLGLVLAVGAVAVGVAR** |
| **P01833** | **PolymeriC immunoglobulin reCeptor** | **CPLLVDSEGWVK** |
| **P01833** | **PolymeriC immunoglobulin reCeptor** | **LVSLTLNLVTR** |
| **P01833** | **PolymeriC immunoglobulin reCeptor** | **RAPAFEGR** |
| **P01833** | **PolymeriC immunoglobulin reCeptor** | **NADLQVLKPEPELVYEDLRGSVTFHCALGPEVANVAK** |
| **P01833** | **PolymeriC immunoglobulin reCeptor** | **DGSFSVVITGLRK** |
| **P01833** | **PolymeriC immunoglobulin reCeptor** | **YLCGAHSDGQLQEGSPIQAWQLFVNEESTIPR** |
| **P01833** | **PolymeriC immunoglobulin reCeptor** | **ILLNPQDK** |
| **P01833** | **PolymeriC immunoglobulin reCeptor** | **IIEGEPNLK** |
| **P01833** | **PolymeriC immunoglobulin reCeptor** | **IRLDIQGTGQLLFSVVINQLR** |
| **P01833** | **PolymeriC immunoglobulin reCeptor** | **LSDAGQYLCQAGDDSNSNK** |
| **P01833** | **PolymeriC immunoglobulin reCeptor** | **GSVTFHCALGPEVANVAK** |
| **P01833** | **PolymeriC immunoglobulin reCeptor** | **CGLGINSR** |
| **P01833** | **PolymeriC immunoglobulin reCeptor** | **GVAGGSVAVLCPYNR** |
| **P01833** | **PolymeriC immunoglobulin reCeptor** | **YWCLWEGAQNGRCPLLVDSEGWVK** |
| **P01833** | **PolymeriC immunoglobulin reCeptor** | **ILLNPQDKDGSFSVVITGLR** |
| **P01833** | **PolymeriC immunoglobulin reCeptor** | **QIGLYPVLVIDSSGYVNPNYTGR** |
| **P01833** | **PolymeriC immunoglobulin reCeptor** | **AAGSRDVSLAK** |
| **P01833** | **PolymeriC immunoglobulin reCeptor** | **VPCHFPCK** |
| **P01833** | **PolymeriC immunoglobulin reCeptor** | **VYTVDLGR** |
| **P01833** | **PolymeriC immunoglobulin reCeptor** | **FSSYEK** |
| **P01833** | **PolymeriC immunoglobulin reCeptor** | **QSSGENCDVVVNTLGKR** |
| **P01833** | **PolymeriC immunoglobulin reCeptor** | **DVSLAKADAAPDEK** |
| **P01833** | **PolymeriC immunoglobulin reCeptor** | **AFVNCDENSR** |
| **P01833** | **PolymeriC immunoglobulin reCeptor** | **SPIFGPEEVNSVEGNSVSITCYYPPTSVNR** |
| **P01833** | **PolymeriC immunoglobulin reCeptor** | **QGHFYGETAAVYVAVEER** |
| **P01833** | **PolymeriC immunoglobulin reCeptor** | **SPTVVK** |
| **P02452** | **Collagen alpha-1(I) Chain** | **SGEYWIDPNQGCNLDAIK** |
| **P02647** | **Apolipoprotein A-I** | **LLDNWDSVTSTFSK** |
| **P02647** | **Apolipoprotein A-I** | **LREQLGPVTQEFWDNLEK** |
| **P02647** | **Apolipoprotein A-I** | **DSGRDYVSQFEGSALGK** |
| **P02647** | **Apolipoprotein A-I** | **VSFLSALEEYTK** |
| **P02647** | **Apolipoprotein A-I** | **VSFLSALEEYTKK** |
| **P02647** | **Apolipoprotein A-I** | **DLATVYVDVLK** |
| **P02647** | **Apolipoprotein A-I** | **KWQEEMELYR** |
| **P02647** | **Apolipoprotein A-I** | **AKVQPYLDDFQK** |
| **P02647** | **Apolipoprotein A-I** | **LSPLGEEMR** |
| **P02647** | **Apolipoprotein A-I** | **AKPALEDLRQGLLPVLESFK** |
| **P02647** | **Apolipoprotein A-I** | **VEPLRAELQEGAR** |
| **P02647** | **Apolipoprotein A-I** | **AKPALEDLR** |
| **P02647** | **Apolipoprotein A-I** | **THLAPYSDELR** |
| **P02647** | **Apolipoprotein A-I** | **ETEGLRQEMSK** |
| **P02647** | **Apolipoprotein A-I** | **EQLGPVTQEFWDNLEK** |
| **P02647** | **Apolipoprotein A-I** | **QGLLPVLESFK** |
| **P02647** | **Apolipoprotein A-I** | **DLEEVK** |
| **P02647** | **Apolipoprotein A-I** | **THLAPYSDELRQR** |
| **P02647** | **Apolipoprotein A-I** | **LEALKENGGAR** |
| **P02647** | **Apolipoprotein A-I** | **DLATVYVDVLKDSGR** |
| **P02647** | **Apolipoprotein A-I** | **WQEEMELYR** |
| **P02647** | **Apolipoprotein A-I** | **ATEHLSTLSEK** |
| **P02647** | **Apolipoprotein A-I** | **VKDLATVYVDVLK** |
| **P02647** | **Apolipoprotein A-I** | **ARAHVDALR** |
| **P02647** | **Apolipoprotein A-I** | **VQPYLDDFQKK** |
| **P02647** | **Apolipoprotein A-I** | **QKLHELQEK** |
| **P02647** | **Apolipoprotein A-I** | **LAARLEALK** |
| **P02647** | **Apolipoprotein A-I** | **DYVSQFEGSALGK** |
| **P02647** | **Apolipoprotein A-I** | **AELQEGAR** |
| **P02647** | **Apolipoprotein A-I** | **VQPYLDDFQK** |
| **P02649** | **Apolipoprotein E** | **VQAAVGTSAAPVPSDNH** |
| **P02649** | **Apolipoprotein E** | **LGADMEDVCGR** |
| **P02649** | **Apolipoprotein E** | **AATVGSLAGQPLQER** |
| **P02649** | **Apolipoprotein E** | **LEEQAQQIR** |
| **P02649** | **Apolipoprotein E** | **WVQTLSEQVQEELLSSQVTQELR** |
| **P02649** | **Apolipoprotein E** | **AKLEEQAQQIR** |
| **P02649** | **Apolipoprotein E** | **SWFEPLVEDMQR** |
| **P02649** | **Apolipoprotein E** | **LAVYQAGAR** |
| **P02649** | **Apolipoprotein E** | **AYKSELEEQLTPVAEETR** |
| **P02649** | **Apolipoprotein E** | **GEVQAMLGQSTEELR** |
| **P02649** | **Apolipoprotein E** | **DADDLQKR** |
| **P02649** | **Apolipoprotein E** | **LGPLVEQGR** |
| **P02649** | **Apolipoprotein E** | **MEEMGSR** |
| **P02649** | **Apolipoprotein E** | **ARMEEMGSR** |
| **P02649** | **Apolipoprotein E** | **QQTEWQSGQR** |
| **P02652** | **Apolipoprotein A-II** | **KAGTELVNFLSYFVELGTQPATQ** |
| **P02652** | **Apolipoprotein A-II** | **EPCVESLVSQYFQTVTDYGK** |
| **P02652** | **Apolipoprotein A-II** | **AGTELVNFLSYFVELGTQPATQ** |
| **P02652** | **Apolipoprotein A-II** | **SPELQAEAK** |
| **P02652** | **Apolipoprotein A-II** | **SKEQLTPLIK** |
| **P02652** | **Apolipoprotein A-II** | **VKSPELQAEAK** |
| **P02652** | **Apolipoprotein A-II** | **EQLTPLIKK** |
| **P02654** | **Apolipoprotein C-I** | **mREWFSETFQK** |
| **P02654** | **Apolipoprotein C-I** | **ARELISR** |
| **P02654** | **Apolipoprotein C-I** | **IKQSELSAK** |
| **P02671** | **Fibrinogen alpha Chain** | **TFPGFFSPMLGEFVSETESR** |
| **P02671** | **Fibrinogen alpha Chain** | **DSDWPFCSDEDWNYK** |
| **P02671** | **Fibrinogen alpha Chain** | **GLIDEVNQDFTNR** |
| **P02671** | **Fibrinogen alpha Chain** | **HRHPDEAAFFDTASTGK** |
| **P02671** | **Fibrinogen alpha Chain** | **mADEAGSEADHEGTHSTK** |
| **P02671** | **Fibrinogen alpha Chain** | **GSESGIFTNTK** |
| **P02671** | **Fibrinogen alpha Chain** | **QFTSSTSYNRGDSTFESK** |
| **P02671** | **Fibrinogen alpha Chain** | **NSLFEYQK** |
| **P02671** | **Fibrinogen alpha Chain** | **EVVTSEDGSDCPEAMDLGTLSGIGTLDGFR** |
| **P02671** | **Fibrinogen alpha Chain** | **DSHSLTTNIMEILR** |
| **P02671** | **Fibrinogen alpha Chain** | **VQHIQLLQK** |
| **P02671** | **Fibrinogen alpha Chain** | **TVIGPDGHKEVTK** |
| **P02671** | **Fibrinogen alpha Chain** | **GGSTSYGTGSETESPRNPSSAGSWNSGSSGPGSTGNR** |
| **P02671** | **Fibrinogen alpha Chain** | **GLIDEVNQDFTNRINK** |
| **P02671** | **Fibrinogen alpha Chain** | **EVDLKDYEDQQK** |
| **P02671** | **Fibrinogen alpha Chain** | **EKVTSGSTTTTR** |
| **P02671** | **Fibrinogen alpha Chain** | **MADEAGSEADHEGTHSTKR** |
| **P02671** | **Fibrinogen alpha Chain** | **MELERPGGNEITR** |
| **P02671** | **Fibrinogen alpha Chain** | **HPDEAAFFDTASTGK** |
| **P02671** | **Fibrinogen alpha Chain** | **QLEQVIAK** |
| **P02671** | **Fibrinogen alpha Chain** | **ESSSHHPGIAEFPSR** |
| **P02671** | **Fibrinogen alpha Chain** | **VTSGSTTTTRR** |
| **P02671** | **Fibrinogen alpha Chain** | **MKGLIDEVNQDFTNR** |
| **P02671** | **Fibrinogen alpha Chain** | **EVTKEVVTSEDGSDCPEAMDLGTLSGIGTLDGFR** |
| **P02671** | **Fibrinogen alpha Chain** | **ALTDMPQMR** |
| **P02671** | **Fibrinogen alpha Chain** | **TVIGPDGHK** |
| **P02671** | **Fibrinogen alpha Chain** | **QFTSSTSYNR** |
| **P02675** | **Fibrinogen beta Chain** | **DNENVVNEYSSELEK** |
| **P02675** | **Fibrinogen beta Chain** | **NYCGLPGEYWLGNDK** |
| **P02675** | **Fibrinogen beta Chain** | **QGFGNVATNTDGK** |
| **P02675** | **Fibrinogen beta Chain** | **YYWGGQYTWDMAK** |
| **P02675** | **Fibrinogen beta Chain** | **GGETSEMYLIQPDSSVKPYR** |
| **P02675** | **Fibrinogen beta Chain** | **TPCTVSCNIPVVSGKECEEIIR** |
| **P02675** | **Fibrinogen beta Chain** | **TPCTVSCNIPVVSGK** |
| **P02675** | **Fibrinogen beta Chain** | **QDGSVDFGR** |
| **P02675** | **Fibrinogen beta Chain** | **KGGETSEMYLIQPDSSVKPYR** |
| **P02675** | **Fibrinogen beta Chain** | **HQLYIDETVNSNIPTNLR** |
| **P02675** | **Fibrinogen beta Chain** | **HGTDDGVVWMNWK** |
| **P02675** | **Fibrinogen beta Chain** | **AHYGGFTVQNEANK** |
| **P02675** | **Fibrinogen beta Chain** | **VYCDMNTENGGWTVIQNR** |
| **P02675** | **Fibrinogen beta Chain** | **mGPTELLIEMEDWKGDK** |
| **P02675** | **Fibrinogen beta Chain** | **EDGGGWWYNR** |
| **P02675** | **Fibrinogen beta Chain** | **IQKLESDVSAQMEYCR** |
| **P02675** | **Fibrinogen beta Chain** | **LESDVSAQMEYCR** |
| **P02675** | **Fibrinogen beta Chain** | **KAPDAGGCLHADPDLGVLCPTGCQLQEALLQQERPIR** |
| **P02675** | **Fibrinogen beta Chain** | **AHYGGFTVQNEANKYQISVNK** |
| **P02675** | **Fibrinogen beta Chain** | **NSVDELNNNVEAVSQTSSSSFQYMYLLK** |
| **P02675** | **Fibrinogen beta Chain** | **QVKDNENVVNEYSSELEK** |
| **P02675** | **Fibrinogen beta Chain** | **REEAPSLRPAPPPISGGGYR** |
| **P02675** | **Fibrinogen beta Chain** | **GHRPLDK** |
| **P02675** | **Fibrinogen beta Chain** | **ISQLTR** |
| **P02675** | **Fibrinogen beta Chain** | **ECEEIIRK** |
| **P02675** | **Fibrinogen beta Chain** | **GSWYSMR** |
| **P02675** | **Fibrinogen beta Chain** | **APDAGGCLHADPDLGVLCPTGCQLQEALLQQERPIR** |
| **P02675** | **Fibrinogen beta Chain** | **YQISVNK** |
| **P02679** | **Fibrinogen gamma Chain** | **FGSYCPTTCGIADFLSTYQTK** |
| **P02679** | **Fibrinogen gamma Chain** | **VAQLEAQCQEPCK** |
| **P02679** | **Fibrinogen gamma Chain** | **YLQEIYNSNNQK** |
| **P02679** | **Fibrinogen gamma Chain** | **DNCCILDER** |
| **P02679** | **Fibrinogen gamma Chain** | **DNCCILDERFGSYCPTTCGIADFLSTYQTK** |
| **P02679** | **Fibrinogen gamma Chain** | **QSGLYFIKPLK** |
| **P02679** | **Fibrinogen gamma Chain** | **EGFGHLSPTGTTEFWLGNEK** |
| **P02679** | **Fibrinogen gamma Chain** | **VAQLEAQCQEPCKDTVQIHDITGK** |
| **P02679** | **Fibrinogen gamma Chain** | **DTVQIHDITGK** |
| **P02679** | **Fibrinogen gamma Chain** | **ANQQFLVYCEIDGSGNGWTVFQK** |
| **P02679** | **Fibrinogen gamma Chain** | **ASTPNGYDNGIIWATWK** |
| **P02679** | **Fibrinogen gamma Chain** | **LTYAYFAGGDAGDAFDGFDFGDDPSDK** |
| **P02679** | **Fibrinogen gamma Chain** | **TSTADYAMFK** |
| **P02679** | **Fibrinogen gamma Chain** | **LTIGEGQQHHLGGAK** |
| **P02679** | **Fibrinogen gamma Chain** | **IHLISTQSAIPYALR** |
| **P02679** | **Fibrinogen gamma Chain** | **VGPEADKYR** |
| **P02679** | **Fibrinogen gamma Chain** | **AIQLTYNPDESSKPNMIDAATLK** |
| **P02679** | **Fibrinogen gamma Chain** | **CHAGHLNGVYYQGGTYSK** |
| **P02679** | **Fibrinogen gamma Chain** | **YEASILTHDSSIR** |
| **P02679** | **Fibrinogen gamma Chain** | **LDGSVDFKK** |
| **P02679** | **Fibrinogen gamma Chain** | **VELEDWNGR** |
| **P02679** | **Fibrinogen gamma Chain** | **MLEEIMKYEASILTHDSSIR** |
| **P02749** | **Beta-2-glyCoprotein 1** | **CSYTEDAQCIDGTIEVPK** |
| **P02749** | **Beta-2-glyCoprotein 1** | **KCSYTEDAQCIDGTIEVPK** |
| **P02749** | **Beta-2-glyCoprotein 1** | **ATFGCHDGYSLDGPEEIECTK** |
| **P02749** | **Beta-2-glyCoprotein 1** | **VCPFAGILENGAVR** |
| **P02749** | **Beta-2-glyCoprotein 1** | **FICPLTGLWPINTLK** |
| **P02749** | **Beta-2-glyCoprotein 1** | **KATVVYQGER** |
| **P02749** | **Beta-2-glyCoprotein 1** | **TDASDVKPC** |
| **P02749** | **Beta-2-glyCoprotein 1** | **ATVVYQGER** |
| **P02749** | **Beta-2-glyCoprotein 1** | **CTEEGKWSPELPVCAPIICPPPSIPTFATLR** |
| **P02749** | **Beta-2-glyCoprotein 1** | **TCPKPDDLPFSTVVPLK** |
| **P02749** | **Beta-2-glyCoprotein 1** | **TFYEPGEEITYSCKPGYVSR** |
| **P02749** | **Beta-2-glyCoprotein 1** | **DKATFGCHDGYSLDGPEEIECTK** |
| **P02749** | **Beta-2-glyCoprotein 1** | **WSPELPVCAPIICPPPSIPTFATLR** |
| **P02751** | **FibroneCtin** | **NTFAEVTGLSPGVTYYFK** |
| **P02751** | **FibroneCtin** | **VREEVVTVGNSVNEGLNQPTDDSCFDPYTVSHYAVGDEWER** |
| **P02751** | **FibroneCtin** | **NLQPASEYTVSLVAIK** |
| **P02751** | **FibroneCtin** | **EYLGAICSCTCFGGQR** |
| **P02751** | **FibroneCtin** | **GFNCESKPEAEETCFDK** |
| **P02751** | **FibroneCtin** | **TGLDSPTGIDFSDITANSFTVHWIAPR** |
| **P02751** | **FibroneCtin** | **TYLGNALVCTCYGGSR** |
| **P02751** | **FibroneCtin** | **DLQFVEVTDVK** |
| **P02751** | **FibroneCtin** | **FTNIGPDTMR** |
| **P02751** | **FibroneCtin** | **SSPVVIDASTAIDAPSNLR** |
| **P02751** | **FibroneCtin** | **FGFCPMAAHEEICTTNEGVMYR** |
| **P02751** | **FibroneCtin** | **CDNCRRPGGEPSPEGTTGQSYNQYSQR** |
| **P02765** | **Alpha-2-HS-glyCoprotein** | **HTFMGVVSLGSPSGEVSHPR** |
| **P02765** | **Alpha-2-HS-glyCoprotein** | **AQLVPLPPSTYVEFTVSGTDCVAK** |
| **P02765** | **Alpha-2-HS-glyCoprotein** | **TVVQPSVGAAAGPVVPPCPGR** |
| **P02765** | **Alpha-2-HS-glyCoprotein** | **HTLNQIDEVK** |
| **P02765** | **Alpha-2-HS-glyCoprotein** | **EHAVEGDCDFQLLK** |
| **P02765** | **Alpha-2-HS-glyCoprotein** | **CNLLAEK** |
| **P02765** | **Alpha-2-HS-glyCoprotein** | **QPNCDDPETEEAALVAIDYINQNLPWGYK** |
| **P02765** | **Alpha-2-HS-glyCoprotein** | **VWPQQPSGELFEIEIDTLETTCHVLDPTPVAR** |
| **P02765** | **Alpha-2-HS-glyCoprotein** | **CDSSPDSAEDVRK** |
| **P02765** | **Alpha-2-HS-glyCoprotein** | **CDSSPDSAEDVR** |
| **P02765** | **Alpha-2-HS-glyCoprotein** | **FSVVYAK** |
| **P02766** | **Transthyretin** | **AADDTWEPFASGK** |
| **P02766** | **Transthyretin** | **GSPAINVAVHVFRK** |
| **P02766** | **Transthyretin** | **YTIAALLSPYSYSTTAVVTNPKE** |
| **P02766** | **Transthyretin** | **TSESGELHGLTTEEEFVEGIYKVEIDTK** |
| **P02766** | **Transthyretin** | **TSESGELHGLTTEEEFVEGIYK** |
| **P02766** | **Transthyretin** | **GSPAINVAVHVFR** |
| **P02766** | **Transthyretin** | **KAADDTWEPFASGK** |
| **P02766** | **Transthyretin** | **ALGISPFHEHAEVVFTANDSGPR** |
| **P02766** | **Transthyretin** | **YTIAALLSPYSYSTTAVVTNPK** |
| **P02766** | **Transthyretin** | **RYTIAALLSPYSYSTTAVVTNPK** |
| **P02768** | **Serum albumin** | **KVPQVSTPTLVEVSR** |
| **P02768** | **Serum albumin** | **DVFLGMFLYEYAR** |
| **P02768** | **Serum albumin** | **TCVADESAENCDK** |
| **P02768** | **Serum albumin** | **AVMDDFAAFVEK** |
| **P02768** | **Serum albumin** | **EFNAETFTFHADICTLSEK** |
| **P02768** | **Serum albumin** | **LVNEVTEFAK** |
| **P02768** | **Serum albumin** | **YICENQDSISSK** |
| **P02768** | **Serum albumin** | **HPYFYAPELLFFAK** |
| **P02768** | **Serum albumin** | **VFDEFKPLVEEPQNLIK** |
| **P02768** | **Serum albumin** | **RHPYFYAPELLFFAK** |
| **P02768** | **Serum albumin** | **ETYGEMADCCAK** |
| **P02768** | **Serum albumin** | **LCTVATLRETYGEMADCCAK** |
| **P02768** | **Serum albumin** | **LVRPEVDVMCTAFHDNEETFLK** |
| **P02768** | **Serum albumin** | **KLVAASQAALGL** |
| **P02768** | **Serum albumin** | **ADDKETCFAEEGK** |
| **P02768** | **Serum albumin** | **ALVLIAFAQYLQQCPFEDHVK** |
| **P02768** | **Serum albumin** | **RHPDYSVVLLLR** |
| **P02768** | **Serum albumin** | **CCTESLVNR** |
| **P02768** | **Serum albumin** | **SHCIAEVENDEMPADLPSLAADFVESK** |
| **P02768** | **Serum albumin** | **KQTALVELVK** |
| **P02768** | **Serum albumin** | **LVAASQAALGL** |
| **P02768** | **Serum albumin** | **AAFTECCQAADK** |
| **P02768** | **Serum albumin** | **QTALVELVK** |
| **P02768** | **Serum albumin** | **QNCELFEQLGEYK** |
| **P02768** | **Serum albumin** | **RMPCAEDYLSVVLNQLCVLHEK** |
| **P02768** | **Serum albumin** | **FQNALLVR** |
| **P02768** | **Serum albumin** | **YLYEIAR** |
| **P02768** | **Serum albumin** | **CCAAADPHECYAK** |
| **P02768** | **Serum albumin** | **AACLLPK** |
| **P02768** | **Serum albumin** | **KYLYEIAR** |
| **P02768** | **Serum albumin** | **ECCEKPLLEK** |
| **P02768** | **Serum albumin** | **CCTESLVNRRPCFSALEVDETYVPK** |
| **P02768** | **Serum albumin** | **MPCAEDYLSVVLNQLCVLHEK** |
| **P02768** | **Serum albumin** | **VHTECCHGDLLECADDRADLAK** |
| **P02768** | **Serum albumin** | **LDELRDEGK** |
| **P02768** | **Serum albumin** | **AWAVAR** |
| **P02768** | **Serum albumin** | **RPCFSALEVDETYVPK** |
| **P02768** | **Serum albumin** | **VPQVSTPTLVEVSR** |
| **P02768** | **Serum albumin** | **DLGEENFK** |
| **P02768** | **Serum albumin** | **LCTVATLR** |
| **P02768** | **Serum albumin** | **AEFAEVSK** |
| **P02768** | **Serum albumin** | **SLHTLFGDK** |
| **P02768** | **Serum albumin** | **LVTDLTK** |
| **P02768** | **Serum albumin** | **VHTECCHGDLLECADDR** |
| **P02768** | **Serum albumin** | **SHCIAEVENDEMPADLPSLAADFVESKDVCK** |
| **P02774** | **Vitamin D-binding protein** | **SYLSMVGSCCTSASPTVCFLK** |
| **P02774** | **Vitamin D-binding protein** | **TAMDVFVCTYFMPAAQLPELPDVELPTNKDVCDPGNTK** |
| **P02774** | **Vitamin D-binding protein** | **EFSHLGKEDFTSLSLVLYSR** |
| **P02774** | **Vitamin D-binding protein** | **SLGECCDVEDSTTCFNAK** |
| **P02774** | **Vitamin D-binding protein** | **LAQKVPTADLEDVLPLAEDITNILSK** |
| **P02774** | **Vitamin D-binding protein** | **EVVSLTEACCAEGADPDCYDTR** |
| **P02774** | **Vitamin D-binding protein** | **KFPSGTFEQVSQLVK** |
| **P02774** | **Vitamin D-binding protein** | **CCESASEDCMAK** |
| **P02774** | **Vitamin D-binding protein** | **SCESNSPFPVHPGTAECCTK** |
| **P02774** | **Vitamin D-binding protein** | **GQELCADYSENTFTEYKK** |
| **P02774** | **Vitamin D-binding protein** | **VCSQYAAYGEKK** |
| **P02774** | **Vitamin D-binding protein** | **VPTADLEDVLPLAEDITNILSK** |
| **P02774** | **Vitamin D-binding protein** | **VCSQYAAYGEK** |
| **P02774** | **Vitamin D-binding protein** | **ELSSFIDKGQELCADYSENTFTEYK** |
| **P02774** | **Vitamin D-binding protein** | **EYANQFMWEYSTNYGQAPLSLLVSYTK** |
| **P02774** | **Vitamin D-binding protein** | **HLSLLTTLSNR** |
| **P02774** | **Vitamin D-binding protein** | **RSDFASNCCSINSPPLYCDSEIDAELK** |
| **P02774** | **Vitamin D-binding protein** | **HQPQEFPTYVEPTNDEICEAFRK** |
| **P02774** | **Vitamin D-binding protein** | **LCDNLSTK** |
| **P02774** | **Vitamin D-binding protein** | **SRLSNLIK** |
| **P02774** | **Vitamin D-binding protein** | **RTHLPEVFLSK** |
| **P02774** | **Vitamin D-binding protein** | **FPSGTFEQVSQLVK** |
| **P02774** | **Vitamin D-binding protein** | **NSKFEDCCQEK** |
| **P02774** | **Vitamin D-binding protein** | **VMDKYTFELSR** |
| **P02774** | **Vitamin D-binding protein** | **TAMDVFVCTYFMPAAQLPELPDVELPTNK** |
| **P02774** | **Vitamin D-binding protein** | **HQPQEFPTYVEPTNDEICEAFR** |
| **P02774** | **Vitamin D-binding protein** | **FEDCCQEK** |
| **P02774** | **Vitamin D-binding protein** | **EDFTSLSLVLYSR** |
| **P02774** | **Vitamin D-binding protein** | **KLCMAALK** |
| **P02774** | **Vitamin D-binding protein** | **GQELCADYSENTFTEYK** |
| **P02774** | **Vitamin D-binding protein** | **SDFASNCCSINSPPLYCDSEIDAELK** |
| **P02774** | **Vitamin D-binding protein** | **GPLLKK** |
| **P02774** | **Vitamin D-binding protein** | **THLPEVFLSK** |
| **P02774** | **Vitamin D-binding protein** | **YTFELSR** |
| **P02775** | **Platelet basiC protein** | **GKEESLDSDLYAELR** |
| **P02775** | **Platelet basiC protein** | **GTHCNQVEVIATLK** |
| **P02775** | **Platelet basiC protein** | **NIQSLEVIGK** |
| **P02775** | **Platelet basiC protein** | **ICLDPDAPR** |
| **P02776** | **Platelet faCtor 4** | **ICLDLQAPLYKK** |
| **P02787** | **Serotransferrin** | **IMNGEADAMSLDGGFVYIAGK** |
| **P02787** | **Serotransferrin** | **AIAANEADAVTLDAGLVYDAYLAPNNLKPVVAEFYGSK** |
| **P02787** | **Serotransferrin** | **CSTSSLLEACTFR** |
| **P02787** | **Serotransferrin** | **SAGWNIPIGLLYCDLPEPR** |
| **P02787** | **Serotransferrin** | **EDLIWELLNQAQEHFGK** |
| **P02787** | **Serotransferrin** | **FDEFFSEGCAPGSK** |
| **P02787** | **Serotransferrin** | **DGAGDVAFVK** |
| **P02787** | **Serotransferrin** | **mYLGYEYVTAIR** |
| **P02787** | **Serotransferrin** | **LCMGSGLNLCEPNNK** |
| **P02787** | **Serotransferrin** | **LKCDEWSVNSVGK** |
| **P02787** | **Serotransferrin** | **LCMGSGLNLCEPNNKEGYYGYTGAFR** |
| **P02787** | **Serotransferrin** | **WCAVSEHEATK** |
| **P02787** | **Serotransferrin** | **INHCRFDEFFSEGCAPGSK** |
| **P02787** | **Serotransferrin** | **SMGGKEDLIWELLNQAQEHFGK** |
| **P02787** | **Serotransferrin** | **CDEWSVNSVGK** |
| **P02787** | **Serotransferrin** | **DCHLAQVPSHTVVAR** |
| **P02787** | **Serotransferrin** | **DSGFQMNQLR** |
| **P02787** | **Serotransferrin** | **EGTCPEAPTDECKPVK** |
| **P02787** | **Serotransferrin** | **CLVEKGDVAFVK** |
| **P02787** | **Serotransferrin** | **KPVEEYANCHLAR** |
| **P02787** | **Serotransferrin** | **IECVSAETTEDCIAK** |
| **P02787** | **Serotransferrin** | **ASYLDCIR** |
| **P02787** | **Serotransferrin** | **DLLFRDDTVCLAK** |
| **P02787** | **Serotransferrin** | **NLREGTCPEAPTDECKPVK** |
| **P02787** | **Serotransferrin** | **CDEWSVNSVGKIECVSAETTEDCIAK** |
| **P02787** | **Serotransferrin** | **EDPQTFYYAVAVVKK** |
| **P02787** | **Serotransferrin** | **SVIPSDGPSVACVKK** |
| **P02787** | **Serotransferrin** | **EFQLFSSPHGK** |
| **P02787** | **Serotransferrin** | **EDPQTFYYAVAVVK** |
| **P02787** | **Serotransferrin** | **ADRDQYELLCLDNTR** |
| **P02787** | **Serotransferrin** | **QQQHLFGSNVTDCSGNFCLFR** |
| **P02787** | **Serotransferrin** | **FDEFFSEGCAPGSKK** |
| **P02787** | **Serotransferrin** | **DYELLCLDGTR** |
| **P02787** | **Serotransferrin** | **WCALSHHER** |
| **P02787** | **Serotransferrin** | **TAGWNIPmGLLYNK** |
| **P02787** | **Serotransferrin** | **HQTVPQNTGGK** |
| **P02787** | **Serotransferrin** | **EGYYGYTGAFR** |
| **P02787** | **Serotransferrin** | **SASDLTWDNLK** |
| **P02787** | **Serotransferrin** | **KASYLDCIR** |
| **P02787** | **Serotransferrin** | **GDVAFVK** |
| **P02787** | **Serotransferrin** | **HSTIFENLANK** |
| **P02787** | **Serotransferrin** | **HQTVPQNTGGKNPDPWAK** |
| **P02787** | **Serotransferrin** | **DSAHGFLK** |
| **P02787** | **Serotransferrin** | **SVIPSDGPSVACVK** |
| **P02787** | **Serotransferrin** | **KDSGFQMNQLR** |
| **P02787** | **Serotransferrin** | **APNHAVVTRK** |
| **P02787** | **Serotransferrin** | **AVANFFSGSCAPCADGTDFPQLCQLCPGCGCSTLNQYFGYSGAFK** |
| **P02787** | **Serotransferrin** | **KSASDLTWDNLK** |
| **P02787** | **Serotransferrin** | **APNHAVVTR** |
| **P02787** | **Serotransferrin** | **KCSTSSLLEACTFR** |
| **P02787** | **Serotransferrin** | **NLNEKDYELLCLDGTR** |
| **P02787** | **Serotransferrin** | **CLKDGAGDVAFVK** |
| **P02787** | **Serotransferrin** | **SKEFQLFSSPHGK** |
| **P02787** | **Serotransferrin** | **KSCHTGLGR** |
| **P02787** | **Serotransferrin** | **KPVDEYKDCHLAQVPSHTVVAR** |
| **P02787** | **Serotransferrin** | **DSAHGFLKVPPR** |
| **P02787** | **Serotransferrin** | **AVGNLRK** |
| **P02787** | **Serotransferrin** | **LHDRNTYEK** |
| **P02787** | **Serotransferrin** | **YLGEEYVK** |
| **P02787** | **Serotransferrin** | **DDTVCLAK** |
| **P02787** | **Serotransferrin** | **CQSFRDHMK** |
| **P02787** | **Serotransferrin** | **HSTIFENLANKADR** |
| **P02787** | **Serotransferrin** | **DQYELLCLDNTR** |
| **P02787** | **Serotransferrin** | **DYELLCLDGTRKPVEEYANCHLAR** |
| **P02787** | **Serotransferrin** | **DQYELLCLDNTRKPVDEYK** |
| **P02787** | **Serotransferrin** | **SCHTGLGR** |
| **P02788** | **LaCtotransferrin** | **LADFALLCLDGK** |
| **P02788** | **LaCtotransferrin** | **SNLCALCIGDEQGENK** |
| **P02788** | **LaCtotransferrin** | **DVTVLQNTDGNNNEAWAK** |
| **P02788** | **LaCtotransferrin** | **NLLFNDNTECLAR** |
| **P02788** | **LaCtotransferrin** | **SQQSSDPDPNCVDRPVEGYLAVAVVR** |
| **P02788** | **LaCtotransferrin** | **CAFSSQEPYFSYSGAFK** |
| **P02788** | **LaCtotransferrin** | **IDSGLYLGSGYFTAIQNLR** |
| **P02788** | **LaCtotransferrin** | **GEADAMSLDGGYVYTAGK** |
| **P02788** | **LaCtotransferrin** | **CLAENAGDVAFVK** |
| **P02788** | **LaCtotransferrin** | **KGGSFQLNELQGLK** |
| **P02788** | **LaCtotransferrin** | **YLGPQYVAGITNLK** |
| **P02788** | **LaCtotransferrin** | **ADAVTLDGGFIYEAGLAPYK** |
| **P02788** | **LaCtotransferrin** | **CGLVPVLAENYK** |
| **P02788** | **LaCtotransferrin** | **YLGPQYVAGITNLKK** |
| **P02788** | **LaCtotransferrin** | **CLRDGAGDVAFIR** |
| **P02788** | **LaCtotransferrin** | **VPSHAVVAR** |
| **P02788** | **LaCtotransferrin** | **LRPVAAEVYGTERQPR** |
| **P02788** | **LaCtotransferrin** | **FDEYFSQSCAPGSDPR** |
| **P02788** | **LaCtotransferrin** | **FQLFGSPSGQK** |
| **P02788** | **LaCtotransferrin** | **FFSASCVPGADK** |
| **P02788** | **LaCtotransferrin** | **DSPIQCIQAIAENRADAVTLDGGFIYEAGLAPYK** |
| **P02788** | **LaCtotransferrin** | **RSDTSLTWNSVK** |
| **P02788** | **LaCtotransferrin** | **VVWCAVGEQELRK** |
| **P02788** | **LaCtotransferrin** | **YYGYTGAFR** |
| **P02788** | **LaCtotransferrin** | **SCHLAMAPNHAVVSR** |
| **P02788** | **LaCtotransferrin** | **FCLFQSETK** |
| **P02788** | **LaCtotransferrin** | **NGSDCPDKFCLFQSETK** |
| **P02788** | **LaCtotransferrin** | **DGAGDVAFIR** |
| **P02788** | **LaCtotransferrin** | **RSVQWCAVSQPEATK** |
| **P02788** | **LaCtotransferrin** | **LKQVLLHQQAK** |
| **P02788** | **LaCtotransferrin** | **CSTSPLLEACEFLR** |
| **P02788** | **LaCtotransferrin** | **FFSASCVPGADKGQFPNLCR** |
| **P02788** | **LaCtotransferrin** | **LRPVAAEVYGTER** |
| **P02788** | **LaCtotransferrin** | **VVWCAVGEQELR** |
| **P02788** | **LaCtotransferrin** | **GGSFQLNELQGLK** |
| **P02788** | **LaCtotransferrin** | **SVQWCAVSQPEATK** |
| **P02788** | **LaCtotransferrin** | **SVNGKEDAIWNLLR** |
| **P02788** | **LaCtotransferrin** | **CSTSPLLEACEFLRK** |
| **P02788** | **LaCtotransferrin** | **DSPIQCIQAIAENR** |
| **P02788** | **LaCtotransferrin** | **SNLCALCIGDEQGENKCVPNSNER** |
| **P02788** | **LaCtotransferrin** | **LCAGTGENK** |
| **P02788** | **LaCtotransferrin** | **SDTSLTWNSVK** |
| **P02788** | **LaCtotransferrin** | **SEEEVAARR** |
| **P02788** | **LaCtotransferrin** | **QVLLHQQAK** |
| **P02788** | **LaCtotransferrin** | **SCHTAVDR** |
| **P02788** | **LaCtotransferrin** | **KSEEEVAAR** |
| **P02788** | **LaCtotransferrin** | **RKPVTEAR** |
| **P02788** | **LaCtotransferrin** | **ESTVFEDLSDEAERDEYELLCPDNTR** |
| **P02788** | **LaCtotransferrin** | **KPVDKFK** |
| **P02790** | **Hemopexin** | **LYLVQGTQVYVFLTK** |
| **P02790** | **Hemopexin** | **EVGTPHGIILDSVDAAFICPGSSR** |
| **P02790** | **Hemopexin** | **SGAQATWTELPWPHEK** |
| **P02790** | **Hemopexin** | **DGWHSWPIAHQWPQGPSAVDAAFSWEEK** |
| **P02790** | **Hemopexin** | **CSPHLVLSALTSDNHGATYAFSGTHYWR** |
| **P02790** | **Hemopexin** | **GGYTLVSGYPK** |
| **P02790** | **Hemopexin** | **YYCFQGNQFLR** |
| **P02790** | **Hemopexin** | **SLGPNSCSANGPGLYLIHGPNLYCYSDVEK** |
| **P02790** | **Hemopexin** | **RLWWLDLK** |
| **P02790** | **Hemopexin** | **GECQAEGVLFFQGDREWFWDLATGTMK** |
| **P02790** | **Hemopexin** | **LDTSRDGWHSWPIAHQWPQGPSAVDAAFSWEEK** |
| **P02790** | **Hemopexin** | **SGAQATWTELPWPHEKVDGALCMEK** |
| **P02790** | **Hemopexin** | **QGHNSVFLIKGDK** |
| **P02790** | **Hemopexin** | **VDGALCMEK** |
| **P02790** | **Hemopexin** | **DVRDYFMPCPGR** |
| **P02790** | **Hemopexin** | **NFPSPVDAAFR** |
| **P02790** | **Hemopexin** | **QGHNSVFLIK** |
| **P02790** | **Hemopexin** | **DYFMPCPGR** |
| **P04083** | **Annexin A1** | **GTDVNVFNTILTTR** |
| **P04083** | **Annexin A1** | **GVDEATIIDILTK** |
| **P04083** | **Annexin A1** | **QAWFIENEEQEYVQTVK** |
| **P04083** | **Annexin A1** | **ALTGHLEEVVLALLK** |
| **P04083** | **Annexin A1** | **GLGTDEDTLIEILASR** |
| **P04083** | **Annexin A1** | **DITSDTSGDFRNALLSLAK** |
| **P04083** | **Annexin A1** | **TPAQFDADELR** |
| **P04083** | **Annexin A1** | **SEIDMNDIK** |
| **P04083** | **Annexin A1** | **ALYEAGER** |
| **P04083** | **Annexin A1** | **GDRSEDFGVNEDLADSDAR** |
| **P04083** | **Annexin A1** | **DITSDTSGDFR** |
| **P04083** | **Annexin A1** | **ILVALCGGN** |
| **P04083** | **Annexin A1** | **CATSKPAFFAEK** |
| **P04083** | **Annexin A1** | **VLDLELKGDIEK** |
| **P04083** | **Annexin A1** | **AAYLQETGKPLDETLK** |
| **P04083** | **Annexin A1** | **CLTAIVK** |
| **P04083** | **Annexin A1** | **GGPGSAVSPYPTFNPSSDVAALHK** |
| **P04083** | **Annexin A1** | **MYGISLCQAILDETKGDYEK** |
| **P04179** | **Superoxide dismutase [Mn], mitoChondrial** | **AIWNVINWENVTER** |
| **P04179** | **Superoxide dismutase [Mn], mitoChondrial** | **GDVTAQIALQPALK** |
| **P04217** | **Alpha-1B-glyCoprotein** | **VTLTCVAPLSGVDFQLR** |
| **P04217** | **Alpha-1B-glyCoprotein** | **SGLSTGWTQLSK** |
| **P04217** | **Alpha-1B-glyCoprotein** | **TPGAAANLELIFVGPQHAGNYR** |
| **P04217** | **Alpha-1B-glyCoprotein** | **ATWSGAVLAGR** |
| **P04217** | **Alpha-1B-glyCoprotein** | **CRSGLSTGWTQLSK** |
| **P04217** | **Alpha-1B-glyCoprotein** | **LHDNQNGWSGDSAPVELILSDETLPAPEFSPEPESGR** |
| **P04217** | **Alpha-1B-glyCoprotein** | **NGVAQEPVHLDSPAIK** |
| **P04217** | **Alpha-1B-glyCoprotein** | **CEGPIPDVTFELLR** |
| **P04217** | **Alpha-1B-glyCoprotein** | **SLPAPWLSMAPVSWITPGLK** |
| **P04217** | **Alpha-1B-glyCoprotein** | **LLELTGPK** |
| **P04217** | **Alpha-1B-glyCoprotein** | **SWVPHTFESELSDPVELLVAES** |
| **P04217** | **Alpha-1B-glyCoprotein** | **SSTSPDRIFFHLNAVALGDGGHYTCR** |
| **P04217** | **Alpha-1B-glyCoprotein** | **GVTFLLR** |
| **P04217** | **Alpha-1B-glyCoprotein** | **FALVR** |
| **P04217** | **Alpha-1B-glyCoprotein** | **CLAPLEGAR** |
| **P04217** | **Alpha-1B-glyCoprotein** | **IFFHLNAVALGDGGHYTCR** |
| **P04217** | **Alpha-1B-glyCoprotein** | **TDGEGALSEPSATVTIEELAAPPPPVLMHHGESSQVLHPGNK** |
| **P04792** | **Heat shoCk protein beta-1** | **LPEEWSQWLGGSSWPGYVRPLPPAAIESPAVAAPAYSR** |
| **P04792** | **Heat shoCk protein beta-1** | **LFDQAFGLPR** |
| **P04792** | **Heat shoCk protein beta-1** | **QLSSGVSEIR** |
| **P04792** | **Heat shoCk protein beta-1** | **LATQSNEITIPVTFESR** |
| **P04792** | **Heat shoCk protein beta-1** | **AQLGGPEAAK** |
| **P04792** | **Heat shoCk protein beta-1** | **RVPFSLLR** |
| **P04792** | **Heat shoCk protein beta-1** | **VSLDVNHFAPDELTVK** |
| **P04792** | **Heat shoCk protein beta-1** | **KYTLPPGVDPTQVSSSLSPEGTLTVEAPMPK** |
| **P05090** | **Apolipoprotein D** | **KMTVTDQVNCPK** |
| **P05090** | **Apolipoprotein D** | **CPNPPVQENFDVNK** |
| **P05090** | **Apolipoprotein D** | **MTVTDQVNCPK** |
| **P05090** | **Apolipoprotein D** | **VLNQELR** |
| **P05090** | **Apolipoprotein D** | **NILTSNNIDVK** |
| **P05090** | **Apolipoprotein D** | **IPTTFENGR** |
| **P05090** | **Apolipoprotein D** | **NILTSNNIDVKK** |
| **P05109** | **Protein S100-A8** | **LLETECPQYIR** |
| **P05109** | **Protein S100-A8** | **YSLIK** |
| **P05109** | **Protein S100-A8** | **GADVWFK** |
| **P05109** | **Protein S100-A8** | **ALNSIIDVYHK** |
| **P05109** | **Protein S100-A8** | **KLLETECPQYIR** |
| **P05109** | **Protein S100-A8** | **MGVAAHKK** |
| **P05164** | **Myeloperoxidase** | **IICDNTGITTVSK** |
| **P05164** | **Myeloperoxidase** | **AVSNEIVRFPTDQLTPDQER** |
| **P05164** | **Myeloperoxidase** | **VVLEGGIDPILR** |
| **P05164** | **Myeloperoxidase** | **NQINALTSFVDASMVYGSEEPLAR** |
| **P05164** | **Myeloperoxidase** | **FCGLPQPETVGQLGTVLR** |
| **P05164** | **Myeloperoxidase** | **IGLDLPALNMQR** |
| **P05164** | **Myeloperoxidase** | **RSPTLGASNR** |
| **P05164** | **Myeloperoxidase** | **IANVFTNAFR** |
| **P05164** | **Myeloperoxidase** | **IPCFLAGDTR** |
| **P05164** | **Myeloperoxidase** | **LFEQVmR** |
| **P05164** | **Myeloperoxidase** | **QALAQISLPR** |
| **P05164** | **Myeloperoxidase** | **WLPAEYEDGFSLPYGWTPGVK** |
| **P05198** | **EukaryotiC translation initiation faCtor 2 subunit 1** | **VVTDTDETELAR** |
| **P05198** | **EukaryotiC translation initiation faCtor 2 subunit 1** | **TEGLSVLSQAMAVIK** |
| **P05783** | **Keratin, type I CytOSkeletal 18** | **TVQSLEIDLDSMR** |
| **P05783** | **Keratin, type I CytOSkeletal 18** | **QAQEYEALLNIK** |
| **P05783** | **Keratin, type I CytOSkeletal 18** | **AQIFANTVDNAR** |
| **P05783** | **Keratin, type I CytOSkeletal 18** | **IIEDLR** |
| **P05787** | **Keratin, type II CytOSkeletal 8** | **LSELEAALQR** |
| **P05787** | **Keratin, type II CytOSkeletal 8** | **LEGLTDEINFLR** |
| **P05787** | **Keratin, type II CytOSkeletal 8** | **SLDMDSIIAEVK** |
| **P05787** | **Keratin, type II CytOSkeletal 8** | **LVSESSDVLPK** |
| **P05787** | **Keratin, type II CytOSkeletal 8** | **AQYEDIANR** |
| **P05787** | **Keratin, type II CytOSkeletal 8** | **SNMDNMFESYINNLRR** |
| **P05787** | **Keratin, type II CytOSkeletal 8** | **ASLEAAIADAEQRGELAIK** |
| **P06396** | **Gelsolin** | **EVQGFESATFLGYFK** |
| **P06396** | **Gelsolin** | **TPSAAYLWVGTGASEAEK** |
| **P06396** | **Gelsolin** | **TGAQELLR** |
| **P06396** | **Gelsolin** | **AGALNSNDAFVLK** |
| **P06396** | **Gelsolin** | **AQPVQVAEGSEPDGFWEALGGK** |
| **P06396** | **Gelsolin** | **DSQEEEKTEALTSAK** |
| **P06396** | **Gelsolin** | **QTQVSVLPEGGETPLFK** |
| **P06396** | **Gelsolin** | **IEGSNKVPVDPATYGQFYGGDSYIILYNYR** |
| **P06396** | **Gelsolin** | **LFQVR** |
| **P06396** | **Gelsolin** | **VEKFDLVPVPTNLYGDFFTGDAYVILK** |
| **P06396** | **Gelsolin** | **AVEVLPK** |
| **P06396** | **Gelsolin** | **EGGQTAPASTR** |
| **P06396** | **Gelsolin** | **KGGVASGFK** |
| **P06396** | **Gelsolin** | **GASQAGAPQGR** |
| **P06396** | **Gelsolin** | **VPFDAATLHTSTAMAAQHGMDDDGTGQK** |
| **P06396** | **Gelsolin** | **YIETDPANR** |
| **P06396** | **Gelsolin** | **VHVSEEGTEPEAMLQVLGPKPALPAGTEDTAKEDAANR** |
| **P06396** | **Gelsolin** | **AGKEPGLQIWR** |
| **P06396** | **Gelsolin** | **LFACSNK** |
| **P06396** | **Gelsolin** | **LKATQVSK** |
| **P06396** | **Gelsolin** | **NWRDPDQTDGLGLSYLSSHIANVER** |
| **P06396** | **Gelsolin** | **SEDCFILDHGKDGK** |
| **P06396** | **Gelsolin** | **VPEARPNSMVVEHPEFLK** |
| **P06703** | **Protein S100-A6** | **LQDAEIAR** |
| **P06703** | **Protein S100-A6** | **ELTIGSKLQDAEIAR** |
| **P06727** | **Apolipoprotein A-IV** | **LGPHAGDVEGHLSFLEK** |
| **P06727** | **Apolipoprotein A-IV** | **LGEVNTYAGDLQK** |
| **P06727** | **Apolipoprotein A-IV** | **SELTQQLNALFQDK** |
| **P06727** | **Apolipoprotein A-IV** | **ALVQQMEQLR** |
| **P06727** | **Apolipoprotein A-IV** | **KLVPFATELHER** |
| **P06727** | **Apolipoprotein A-IV** | **SLAELGGHLDQQVEEFR** |
| **P06727** | **Apolipoprotein A-IV** | **SLAPYAQDTQEK** |
| **P06727** | **Apolipoprotein A-IV** | **LNHQLEGLTFQMK** |
| **P06727** | **Apolipoprotein A-IV** | **RVEPYGENFNK** |
| **P06727** | **Apolipoprotein A-IV** | **AKIDQNVEELK** |
| **P06727** | **Apolipoprotein A-IV** | **ISASAEELR** |
| **P06727** | **Apolipoprotein A-IV** | **SLAELGGHLDQQVEEFRR** |
| **P06727** | **Apolipoprotein A-IV** | **GRLTPYADEFK** |
| **P06727** | **Apolipoprotein A-IV** | **IDQNVEELK** |
| **P06727** | **Apolipoprotein A-IV** | **LLPHANEVSQK** |
| **P06727** | **Apolipoprotein A-IV** | **VLRENADSLQASLRPHADELK** |
| **P06727** | **Apolipoprotein A-IV** | **LAPLAEDVR** |
| **P06727** | **Apolipoprotein A-IV** | **EAVEHLQK** |
| **P06727** | **Apolipoprotein A-IV** | **VNSFFSTFK** |
| **P06733** | **Alpha-enolase** | **FTASAGIQVVGDDLTVTNPK** |
| **P06733** | **Alpha-enolase** | **GNPTVEVDLFTSK** |
| **P06733** | **Alpha-enolase** | **VVIGMDVAASEFFR** |
| **P06733** | **Alpha-enolase** | **LAQANGWGVMVSHR** |
| **P06733** | **Alpha-enolase** | **HIADLAGNSEVILPVPAFNVINGGSHAGNK** |
| **P06733** | **Alpha-enolase** | **LAMQEFMILPVGAANFR** |
| **P06733** | **Alpha-enolase** | **DATNVGDEGGFAPNILENK** |
| **P06733** | **Alpha-enolase** | **YISPDQLADLYK** |
| **P06733** | **Alpha-enolase** | **AAVPSGASTGIYEALELRDNDK** |
| **P06733** | **Alpha-enolase** | **DATNVGDEGGFAPNILENKEGLELLK** |
| **P06733** | **Alpha-enolase** | **DYPVVSIEDPFDQDDWGAWQK** |
| **P06733** | **Alpha-enolase** | **IGAEVYHNLK** |
| **P06733** | **Alpha-enolase** | **KLNVTEQEK** |
| **P06733** | **Alpha-enolase** | **YNQLLRIEEELGSK** |
| **P06733** | **Alpha-enolase** | **LMIEMDGTENK** |
| **P06733** | **Alpha-enolase** | **SGKYDLDFK** |
| **P06733** | **Alpha-enolase** | **LNVTEQEK** |
| **P06733** | **Alpha-enolase** | **SPDDPSRYISPDQLADLYK** |
| **P06733** | **Alpha-enolase** | **SGETEDTFIADLVVGLCTGQIK** |
| **P06733** | **Alpha-enolase** | **FTASAGIQVVGDDLTVTNPKR** |
| **P06733** | **Alpha-enolase** | **IDKLMIEMDGTENK** |
| **P06733** | **Alpha-enolase** | **SFIKDYPVVSIEDPFDQDDWGAWQK** |
| **P06733** | **Alpha-enolase** | **YGKDATNVGDEGGFAPNILENK** |
| **P06733** | **Alpha-enolase** | **NFRNPLAK** |
| **P06733** | **Alpha-enolase** | **LNVTEQEKIDK** |
| **P06733** | **Alpha-enolase** | **YDLDFKSPDDPSR** |
| **P06733** | **Alpha-enolase** | **IEEELGSK** |
| **P06733** | **Alpha-enolase** | **AVEHINK** |
| **P06733** | **Alpha-enolase** | **TIAPALVSKK** |
| **P06733** | **Alpha-enolase** | **AAVPSGASTGIYEALELR** |
| **P06733** | **Alpha-enolase** | **FGANAILGVSLAVCK** |
| **P06733** | **Alpha-enolase** | **SCNCLLLK** |
| **P07108** | **ACyl-CoA-binding protein** | **TKPSDEEMLFIYGHYK** |
| **P07108** | **ACyl-CoA-binding protein** | **AKWDAWNELK** |
| **P07108** | **ACyl-CoA-binding protein** | **QATVGDINTERPGMLDFTGK** |
| **P07108** | **ACyl-CoA-binding protein** | **AYINKVEELK** |
| **P07237** | **Protein disulfide-isomerase** | **VDATEESDLAQQYGVR** |
| **P07237** | **Protein disulfide-isomerase** | **MDSTANEVEAVK** |
| **P07237** | **Protein disulfide-isomerase** | **SNFAEALAAHK** |
| **P07237** | **Protein disulfide-isomerase** | **ILEFFGLK** |
| **P07237** | **Protein disulfide-isomerase** | **RTGPAATTLPDGAAAESLVESSEVAVIGFFK** |
| **P07237** | **Protein disulfide-isomerase** | **QFLQAAEAIDDIPFGITSNSDVFSK** |
| **P07237** | **Protein disulfide-isomerase** | **LITLEEEMTK** |
| **P07237** | **Protein disulfide-isomerase** | **TGPAATTLPDGAAAESLVESSEVAVIGFFK** |
| **P07237** | **Protein disulfide-isomerase** | **YKPESEELTAER** |
| **P07237** | **Protein disulfide-isomerase** | **YQLDKDGVVLFK** |
| **P07339** | **Cathepsin D** | **AIGAVPLIQGEYMIPCEK** |
| **P07339** | **Cathepsin D** | **FDGILGMAYPR** |
| **P07339** | **Cathepsin D** | **ISVNNVLPVFDNLMQQK** |
| **P07339** | **Cathepsin D** | **EGCEAIVDTGTSLMVGPVDEVRELQK** |
| **P07339** | **Cathepsin D** | **VGFAEAAR** |
| **P07339** | **Cathepsin D** | **EGCEAIVDTGTSLMVGPVDEVR** |
| **P07339** | **Cathepsin D** | **LVDQNIFSFYLSRDPDAQPGGELMLGGTDSK** |
| **P07339** | **Cathepsin D** | **QVFGEATKQPGITFIAAK** |
| **P07355** | **Annexin A2** | **GVDEVTIVNILTNR** |
| **P07355** | **Annexin A2** | **EVKGDLENAFLNLVQCIQNKPLYFADR** |
| **P07355** | **Annexin A2** | **GLGTDEDSLIEIICSR** |
| **P07355** | **Annexin A2** | **SALSGHLETVILGLLK** |
| **P07355** | **Annexin A2** | **AYTNFDAERDALNIETAIK** |
| **P07355** | **Annexin A2** | **SLYYYIQQDTKGDYQK** |
| **P07355** | **Annexin A2** | **QDIAFAYQR** |
| **P07355** | **Annexin A2** | **TPAQYDASELK** |
| **P07355** | **Annexin A2** | **LMVALAK** |
| **P07355** | **Annexin A2** | **SEVDMLK** |
| **P07355** | **Annexin A2** | **GDLENAFLNLVQCIQNKPLYFADR** |
| **P07355** | **Annexin A2** | **TKGVDEVTIVNILTNR** |
| **P07355** | **Annexin A2** | **DIISDTSGDFRK** |
| **P07355** | **Annexin A2** | **AEDGSVIDYELIDQDAR** |
| **P07355** | **Annexin A2** | **LSLEGDHSTPPSAYGSVK** |
| **P07355** | **Annexin A2** | **RAEDGSVIDYELIDQDAR** |
| **P07355** | **Annexin A2** | **SYSPYDMLESIR** |
| **P07355** | **Annexin A2** | **SLYYYIQQDTK** |
| **P07355** | **Annexin A2** | **SYSPYDMLESIRK** |
| **P07355** | **Annexin A2** | **ALLYLCGGDD** |
| **P07858** | **Cathepsin B** | **ILRGQDHCGIESEVVAGIPR** |
| **P07858** | **Cathepsin B** | **HYGYNSYSVSNSEK** |
| **P07858** | **Cathepsin B** | **SGVYQHVTGEMMGGHAIR** |
| **P07858** | **Cathepsin B** | **LPASFDAR** |
| **P07858** | **Cathepsin B** | **VMFTEDLKLPASFDAR** |
| **P07858** | **Cathepsin B** | **ICEPGYSPTYKQDK** |
| **P07951** | **TropomyOSin beta Chain** | **QLEEEQQALQK** |
| **P08107** | **Heat shoCk 70 kDa protein 1A/1B** | **IINEPTAAAIAYGLDR** |
| **P08107** | **Heat shoCk 70 kDa protein 1A/1B** | **NQVALNPQNTVFDAK** |
| **P08107** | **Heat shoCk 70 kDa protein 1A/1B** | **DAGVIAGLNVLR** |
| **P08107** | **Heat shoCk 70 kDa protein 1A/1B** | **ARFEELCSDLFR** |
| **P08107** | **Heat shoCk 70 kDa protein 1A/1B** | **HWPFQVINDGDKPK** |
| **P08107** | **Heat shoCk 70 kDa protein 1A/1B** | **ELEQVCNPIISGLYQGAGGPGPGGFGAQGPK** |
| **P08107** | **Heat shoCk 70 kDa protein 1A/1B** | **AFYPEEISSMVLTK** |
| **P08107** | **Heat shoCk 70 kDa protein 1A/1B** | **NALESYAFNMK** |
| **P08107** | **Heat shoCk 70 kDa protein 1A/1B** | **SAVEDEGLK** |
| **P08107** | **Heat shoCk 70 kDa protein 1A/1B** | **KFGDPVVQSDMK** |
| **P08107** | **Heat shoCk 70 kDa protein 1A/1B** | **VQVSYKGETK** |
| **P08107** | **Heat shoCk 70 kDa protein 1A/1B** | **AQIHDLVLVGGSTR** |
| **P08107** | **Heat shoCk 70 kDa protein 1A/1B** | **SINPDEAVAYGAAVQAAILMGDK** |
| **P08107** | **Heat shoCk 70 kDa protein 1A/1B** | **ATAGDTHLGGEDFDNR** |
| **P08107** | **Heat shoCk 70 kDa protein 1A/1B** | **YKAEDEVQR** |
| **P08253** | **72 kDa type IV Collagenase** | **AVFFAGNEYWIYSASTLER** |
| **P08253** | **72 kDa type IV Collagenase** | **LIADAWNAIPDNLDAVVDLQGGGHSYFFK** |
| **P08253** | **72 kDa type IV Collagenase** | **DKPmGPLLVATFWPELPEKIDAVYEAPQEEK** |
| **P08670** | **Vimentin** | **ILLAELEQLK** |
| **P08670** | **Vimentin** | **KVESLQEEIAFLK** |
| **P08670** | **Vimentin** | **LLEGEESR** |
| **P08670** | **Vimentin** | **EMEENFAVEAANYQDTIGR** |
| **P08670** | **Vimentin** | **DGQVINETSQHHDDLE** |
| **P08670** | **Vimentin** | **QDVDNASLAR** |
| **P08670** | **Vimentin** | **RQVDQLTNDK** |
| **P08670** | **Vimentin** | **FADLSEAANRNNDALR** |
| **P08670** | **Vimentin** | **SSVPGVR** |
| **P08670** | **Vimentin** | **NLQEAEEWYK** |
| **P08670** | **Vimentin** | **LLQDSVDFSLADAINTEFK** |
| **P08670** | **Vimentin** | **LGDLYEEEMR** |
| **P08670** | **Vimentin** | **FANYIDK** |
| **P08670** | **Vimentin** | **TNEKVELQELNDR** |
| **P08670** | **Vimentin** | **ISLPLPNFSSLNLR** |
| **P08670** | **Vimentin** | **EYQDLLNVK** |
| **P08670** | **Vimentin** | **SRLGDLYEEEMR** |
| **P08758** | **Annexin A5** | **VLTEIIASR** |
| **P08758** | **Annexin A5** | **GLGTDEESILTLLTSR** |
| **P08758** | **Annexin A5** | **SIPAYLAETLYYAMK** |
| **P08758** | **Annexin A5** | **LYDAYELK** |
| **P08758** | **Annexin A5** | **NFATSLYSMIK** |
| **P08758** | **Annexin A5** | **GAGTDDHTLIR** |
| **P08758** | **Annexin A5** | **YMTISGFQIEETIDRETSGNLEQLLLAVVK** |
| **P08758** | **Annexin A5** | **FITIFGTR** |
| **P08758** | **Annexin A5** | **QVYEEEYGSSLEDDVVGDTSGYYQR** |
| **P08758** | **Annexin A5** | **AIKQVYEEEYGSSLEDDVVGDTSGYYQR** |
| **P08758** | **Annexin A5** | **ALLLLCGEDD** |
| **P08758** | **Annexin A5** | **GTVTDFPGFDERADAETLR** |
| **P08758** | **Annexin A5** | **SNAQRQEISAAFK** |
| **P08758** | **Annexin A5** | **SEIDLFNIR** |
| **P08758** | **Annexin A5** | **ADAETLRK** |
| **P08758** | **Annexin A5** | **SEIDLFNIRK** |
| **P08758** | **Annexin A5** | **WGTDEEK** |
| **P08758** | **Annexin A5** | **ETSGNLEQLLLAVVK** |
| **P08758** | **Annexin A5** | **DLLDDLKSELTGK** |
| **P08758** | **Annexin A5** | **QEISAAFK** |
| **P08758** | **Annexin A5** | **LIVALMKPSR** |
| **P08758** | **Annexin A5** | **GTVTDFPGFDER** |
| **P09211** | **Glutathione S-transferase P** | **FQDGDLTLYQSNTILR** |
| **P09211** | **Glutathione S-transferase P** | **TLGLYGKDQQEAALVDMVNDGVEDLR** |
| **P09211** | **Glutathione S-transferase P** | **ALPGQLKPFETLLSQNQGGK** |
| **P09211** | **Glutathione S-transferase P** | **ASCLYGQLPK** |
| **P09211** | **Glutathione S-transferase P** | **PPYTVVYFPVR** |
| **P09211** | **Glutathione S-transferase P** | **MLLADQGQSWKEEVVTVETWQEGSLK** |
| **P09211** | **Glutathione S-transferase P** | **DQQEAALVDMVNDGVEDLR** |
| **P09211** | **Glutathione S-transferase P** | **AFLASPEYVNLPINGNGKQ** |
| **P09211** | **Glutathione S-transferase P** | **DQQEAALVDMVNDGVEDLRCK** |
| **P09211** | **Glutathione S-transferase P** | **YISLIYTNYEAGKDDYVK** |
| **P09237** | **Matrilysin** | **RFYLYDSETK** |
| **P09237** | **Matrilysin** | **VVWGTADIMIGFAR** |
| **P09237** | **Matrilysin** | **FFGLPITGMLNSR** |
| **P09237** | **Matrilysin** | **ALNMWGK** |
| **P09237** | **Matrilysin** | **CGVPDVAEYSLFPNSPK** |
| **P09382** | **GaleCtin-1** | **FNAHGDANTIVCNSK** |
| **P09382** | **GaleCtin-1** | **DGGAWGTEQR** |
| **P09382** | **GaleCtin-1** | **DSNNLCLHFNPR** |
| **P09382** | **GaleCtin-1** | **SFVLNLGK** |
| **P09382** | **GaleCtin-1** | **LNLEAINYMAADGDFK** |
| **P09382** | **GaleCtin-1** | **VRGEVAPDAK** |
| **P09382** | **GaleCtin-1** | **EAVFPFQPGSVAEVCITFDQANLTVK** |
| **P09382** | **GaleCtin-1** | **LPDGYEFK** |
| **P09455** | **Retinol-binding protein 1** | **MLVNENFEEYLR** |
| **P09455** | **Retinol-binding protein 1** | **ALDVNVALR** |
| **P09455** | **Retinol-binding protein 1** | **VEGVVCK** |
| **P09455** | **Retinol-binding protein 1** | **ALDVNVALRK** |
| **P09455** | **Retinol-binding protein 1** | **EIVQDGDHMIIR** |
| **P09455** | **Retinol-binding protein 1** | **IANLLKPDKEIVQDGDHMIIR** |
| **P09455** | **Retinol-binding protein 1** | **NYIMDFQVGKEFEEDLTGIDDR** |
| **P09466** | **GlyCodelin** | **VLVEDDEIMQGFIR** |
| **P09466** | **GlyCodelin** | **VLGEKTENPK** |
| **P09493** | **TropomyOSin alpha-1 Chain** | **MEIQEIQLK** |
| **P09603** | **MaCrophage Colony-stimulating faCtor 1** | **AFLLVQDIMEDTMR** |
| **P09603** | **MaCrophage Colony-stimulating faCtor 1** | **FRDNTPNAIAIVQLQELSLR** |
| **P09603** | **MaCrophage Colony-stimulating faCtor 1** | **TFYETPLQLLEK** |
| **P09651** | **Heterogeneous nuClear ribonuCleoprotein A1** | **LFIGGLSFETTDESLR** |
| **P09651** | **Heterogeneous nuClear ribonuCleoprotein A1** | **NQGGYGGSSSSSSYGSGR** |
| **P09651** | **Heterogeneous nuClear ribonuCleoprotein A1** | **RGFAFVTFDDHDSVDK** |
| **P09651** | **Heterogeneous nuClear ribonuCleoprotein A1** | **EDSQRPGAHLTVK** |
| **P0C0L4** | **Complement C4-A** | **LQETSNWLLSQQQADGSFQDPCPVLDR** |
| **P10451** | **OSteopontin** | **AIPVAQDLNAPSDWDSR** |
| **P10451** | **OSteopontin** | **QNLLAPQNAVSSEETNDFKQETLPSK** |
| **P10451** | **OSteopontin** | **FRISHELDSASSEVN** |
| **P10451** | **OSteopontin** | **ISHELDSASSEVN** |
| **P10451** | **OSteopontin** | **KANDESNEHSDVIDSQELSK** |
| **P10451** | **OSteopontin** | **QLYNKYPDAVATWLNPDPSQK** |
| **P10451** | **OSteopontin** | **ANDESNEHSDVIDSQELSK** |
| **P10451** | **OSteopontin** | **GKDSYETSQLDDQSAETHSHK** |
| **P10909** | **Clusterin** | **CREILSVDCSTNNPSQAK** |
| **P10909** | **Clusterin** | **LFDSDPITVTVPVEVSR** |
| **P10909** | **Clusterin** | **LFDSDPITVTVPVEVSRK** |
| **P10909** | **Clusterin** | **EILSVDCSTNNPSQAK** |
| **P10909** | **Clusterin** | **ASSIIDELFQDR** |
| **P10909** | **Clusterin** | **VTTVASHTSDSDVPSGVTEVVVK** |
| **P10909** | **Clusterin** | **TLLSNLEEAK** |
| **P10909** | **Clusterin** | **ELDESLQVAER** |
| **P10909** | **Clusterin** | **TLLSNLEEAKK** |
| **P10909** | **Clusterin** | **YNELLK** |
| **P10909** | **Clusterin** | **KYNELLK** |
| **P10909** | **Clusterin** | **ALQEYR** |
| **P10909** | **Clusterin** | **FMETVAEK** |
| **P10909** | **Clusterin** | **EIQNAVNGVK** |
| **P10909** | **Clusterin** | **SYQWK** |
| **P10909** | **Clusterin** | **KTLLSNLEEAK** |
| **P10909** | **Clusterin** | **EGDDDRTVCR** |
| **P10909** | **Clusterin** | **ALQEYRK** |
| **P11021** | **78 kDa gluCOSe-regulated protein** | **IEIESFYEGEDFSETLTR** |
| **P11021** | **78 kDa gluCOSe-regulated protein** | **NQLTSNPENTVFDAK** |
| **P11021** | **78 kDa gluCOSe-regulated protein** | **TWNDPSVQQDIK** |
| **P11021** | **78 kDa gluCOSe-regulated protein** | **TFAPEEISAMVLTK** |
| **P11021** | **78 kDa gluCOSe-regulated protein** | **ELEEIVQPIISK** |
| **P11021** | **78 kDa gluCOSe-regulated protein** | **DAGTIAGLNVMR** |
| **P11021** | **78 kDa gluCOSe-regulated protein** | **LYGSAGPPPTGEEDTAEKDEL** |
| **P11021** | **78 kDa gluCOSe-regulated protein** | **AKFEELNMDLFR** |
| **P11021** | **78 kDa gluCOSe-regulated protein** | **VYEGERPLTK** |
| **P11021** | **78 kDa gluCOSe-regulated protein** | **NELESYAYSLK** |
| **P11021** | **78 kDa gluCOSe-regulated protein** | **MKETAEAYLGK** |
| **P11021** | **78 kDa gluCOSe-regulated protein** | **IEWLESHQDADIEDFK** |
| **P11021** | **78 kDa gluCOSe-regulated protein** | **RALSSQHQAR** |
| **P11021** | **78 kDa gluCOSe-regulated protein** | **ITITNDQNR** |
| **P11021** | **78 kDa gluCOSe-regulated protein** | **KSQIFSTASDNQPTVTIK** |
| **P11021** | **78 kDa gluCOSe-regulated protein** | **NKITITNDQNR** |
| **P11021** | **78 kDa gluCOSe-regulated protein** | **KVTHAVVTVPAYFNDAQR** |
| **P11021** | **78 kDa gluCOSe-regulated protein** | **TKPYIQVDIGGGQTK** |
| **P11021** | **78 kDa gluCOSe-regulated protein** | **KTKPYIQVDIGGGQTK** |
| **P11166** | **Solute Carrier family 2, faCilitated gluCOSe transporter member 1** | **TFDEIASGFR** |
| **P11388** | **DNA topoisomerase 2-alpha** | **KYDTVLDILR** |
| **P12004** | **Proliferating Cell nuClear antigen** | **YLNFFTK** |
| **P12004** | **Proliferating Cell nuClear antigen** | **FSASGELGNGNIK** |
| **P12004** | **Proliferating Cell nuClear antigen** | **LMDLDVEQLGIPEQEYSCVVK** |
| **P12277** | **Creatine kinase B-type** | **FCTGLTQIETLFK** |
| **P12277** | **Creatine kinase B-type** | **VLTPELYAELR** |
| **P12277** | **Creatine kinase B-type** | **LGFSEVELVQMVVDGVK** |
| **P12277** | **Creatine kinase B-type** | **LLIEMEQR** |
| **P12830** | **Cadherin-1** | **VFYSITGQGADTPPVGVFIIER** |
| **P12830** | **Cadherin-1** | **VFYSITGQGADTPPVGVFIIERETGWLK** |
| **P12830** | **Cadherin-1** | **GQVPENEANVVITTLK** |
| **P12830** | **Cadherin-1** | **DTANWLEINPDTGAISTR** |
| **P12830** | **Cadherin-1** | **VTDADAPNTPAWEAVYTILNDDGGQFVVTTNPVNNDGILK** |
| **P14174** | **MaCrophage migration inhibitory faCtor** | **LLCGLLAER** |
| **P14174** | **MaCrophage migration inhibitory faCtor** | **PMFIVNTNVPR** |
| **P14174** | **MaCrophage migration inhibitory faCtor** | **ISPDRVYINYYDMNAANVGWNNSTFA** |
| **P14174** | **MaCrophage migration inhibitory faCtor** | **VYINYYDMNAANVGWNNSTFA** |
| **P14618** | **Pyruvate kinase isozymes M1/M2** | **LAPITSDPTEATAVGAVEASFK** |
| **P14618** | **Pyruvate kinase isozymes M1/M2** | **FGVEQDVDMVFASFIR** |
| **P14618** | **Pyruvate kinase isozymes M1/M2** | **RFDEILEASDGIMVAR** |
| **P14618** | **Pyruvate kinase isozymes M1/M2** | **GADFLVTEVENGGSLGSK** |
| **P14618** | **Pyruvate kinase isozymes M1/M2** | **GVNLPGAAVDLPAVSEK** |
| **P14618** | **Pyruvate kinase isozymes M1/M2** | **FGVEQDVDMVFASFIRK** |
| **P14618** | **Pyruvate kinase isozymes M1/M2** | **NTGIICTIGPASR** |
| **P14618** | **Pyruvate kinase isozymes M1/M2** | **AEGSDVANAVLDGADCIMLSGETAKGDYPLEAVR** |
| **P14618** | **Pyruvate kinase isozymes M1/M2** | **IYVDDGLISLQVK** |
| **P14618** | **Pyruvate kinase isozymes M1/M2** | **CDENILWLDYK** |
| **P14618** | **Pyruvate kinase isozymes M1/M2** | **LNFSHGTHEYHAETIK** |
| **P14618** | **Pyruvate kinase isozymes M1/M2** | **AGKPVICATQMLESMIK** |
| **P14618** | **Pyruvate kinase isozymes M1/M2** | **KGVNLPGAAVDLPAVSEK** |
| **P14618** | **Pyruvate kinase isozymes M1/M2** | **LDIDSPPITAR** |
| **P14618** | **Pyruvate kinase isozymes M1/M2** | **APIIAVTRNPQTAR** |
| **P14618** | **Pyruvate kinase isozymes M1/M2** | **CCSGAIIVLTK** |
| **P14618** | **Pyruvate kinase isozymes M1/M2** | **KGDVVIVLTGWRPGSGFTNTMR** |
| **P14618** | **Pyruvate kinase isozymes M1/M2** | **GSGTAEVELK** |
| **P14618** | **Pyruvate kinase isozymes M1/M2** | **GDLGIEIPAEK** |
| **P14618** | **Pyruvate kinase isozymes M1/M2** | **TATESFASDPILYRPVAVALDTKGPEIR** |
| **P14618** | **Pyruvate kinase isozymes M1/M2** | **GSGTAEVELKK** |
| **P14618** | **Pyruvate kinase isozymes M1/M2** | **IISKIENHEGVR** |
| **P14618** | **Pyruvate kinase isozymes M1/M2** | **GIFPVLCKDPVQEAWAEDVDLR** |
| **P14618** | **Pyruvate kinase isozymes M1/M2** | **GADFLVTEVENGGSLGSKK** |
| **P14618** | **Pyruvate kinase isozymes M1/M2** | **VFLAQK** |
| **P14618** | **Pyruvate kinase isozymes M1/M2** | **MQHLIAR** |
| **P14618** | **Pyruvate kinase isozymes M1/M2** | **TATESFASDPILYRPVAVALDTK** |
| **P14618** | **Pyruvate kinase isozymes M1/M2** | **ITLDNAYMEK** |
| **P14618** | **Pyruvate kinase isozymes M1/M2** | **FDEILEASDGIMVAR** |
| **P14618** | **Pyruvate kinase isozymes M1/M2** | **QKGADFLVTEVENGGSLGSK** |
| **P14618** | **Pyruvate kinase isozymes M1/M2** | **DPVQEAWAEDVDLR** |
| **P14618** | **Pyruvate kinase isozymes M1/M2** | **SGMNVAR** |
| **P14618** | **Pyruvate kinase isozymes M1/M2** | **APIIAVTR** |
| **P14618** | **Pyruvate kinase isozymes M1/M2** | **ASDVHEVR** |
| **P14618** | **Pyruvate kinase isozymes M1/M2** | **AEGSDVANAVLDGADCIMLSGETAK** |
| **P14618** | **Pyruvate kinase isozymes M1/M2** | **GVNLPGAAVDLPAVSEKDIQDLK** |
| **P14618** | **Pyruvate kinase isozymes M1/M2** | **RLAPITSDPTEATAVGAVEASFK** |
| **P14618** | **Pyruvate kinase isozymes M1/M2** | **IENHEGVR** |
| **P14618** | **Pyruvate kinase isozymes M1/M2** | **GDYPLEAVR** |
| **P14780** | **Matrix metalloproteinase-9** | **SLGPALLLLQK** |
| **P15328** | **Folate reCeptor alpha** | **EDCEQWWEDCR** |
| **P15328** | **Folate reCeptor alpha** | **CIQMWFDPAQGNPNEEVAR** |
| **P15328** | **Folate reCeptor alpha** | **VLNVPLCK** |
| **P15328** | **Folate reCeptor alpha** | **TELLNVCMNAK** |
| **P15328** | **Folate reCeptor alpha** | **FNWNHCGEMAPACK** |
| **P15328** | **Folate reCeptor alpha** | **EDCEQWWEDCRTSYTCK** |
| **P15531** | **NuCleOSide diphOSphate kinase A** | **YMHSGPVVAMVWEGLNVVK** |
| **P15531** | **NuCleOSide diphOSphate kinase A** | **NIIHGSDSVESAEK** |
| **P15531** | **NuCleOSide diphOSphate kinase A** | **DRPFFAGLVK** |
| **P15531** | **NuCleOSide diphOSphate kinase A** | **FMQASEDLLK** |
| **P15941** | **MuCin-1** | **DISEMFLQIYK** |
| **P15941** | **MuCin-1** | **QGGFLGLSNIK** |
| **P15941** | **MuCin-1** | **NYGQLDIFPAR** |
| **P16035** | **Metalloproteinase inhibitor 2** | **EVDSGNDIYGNPIK** |
| **P16070** | **CD44 antigen** | **YGFIEGHVVIPR** |
| **P16949** | **Stathmin** | **RASGQAFELILSPR** |
| **P16949** | **Stathmin** | **AIEENNNFSK** |
| **P16949** | **Stathmin** | **KLEAAEER** |
| **P16949** | **Stathmin** | **ASGQAFELILSPR** |
| **P16949** | **Stathmin** | **ESVPEFPLSPPK** |
| **P16949** | **Stathmin** | **SKESVPEFPLSPPK** |
| **P16949** | **Stathmin** | **DLSLEEIQK** |
| **P16949** | **Stathmin** | **DKHIEEVR** |
| **P16949** | **Stathmin** | **SHEAEVLK** |
| **P17931** | **GaleCtin-3** | **VAVNDAHLLQYNHR** |
| **P17931** | **GaleCtin-3** | **MLITILGTVKPNANR** |
| **P17931** | **GaleCtin-3** | **IALDFQR** |
| **P17931** | **GaleCtin-3** | **IQVLVEPDHFK** |
| **P17931** | **GaleCtin-3** | **GNDVAFHFNPR** |
| **P19338** | **NuCleolin** | **TLVLSNLSYSATEETLQEVFEK** |
| **P19338** | **NuCleolin** | **FGYVDFESAEDLEK** |
| **P19338** | **NuCleolin** | **VEGTEPTTAFNLFVGNLNFNK** |
| **P19338** | **NuCleolin** | **GFGFVDFNSEEDAK** |
| **P19338** | **NuCleolin** | **GLSEDTTEETLK** |
| **P19338** | **NuCleolin** | **EAMEDGEIDGNK** |
| **P19338** | **NuCleolin** | **ALELTGLK** |
| **P19338** | **NuCleolin** | **TGISDVFAK** |
| **P19338** | **NuCleolin** | **QKVEGTEPTTAFNLFVGNLNFNK** |
| **P19338** | **NuCleolin** | **VTQDELKEVFEDAAEIR** |
| **P19338** | **NuCleolin** | **IVTDRETGSSK** |
| **P19338** | **NuCleolin** | **EAMEDGEIDGNKVTLDWAKPK** |
| **P19338** | **NuCleolin** | **SISLYYTGEK** |
| **P19338** | **NuCleolin** | **NDLAVVDVR** |
| **P19338** | **NuCleolin** | **GLSEDTTEETLKESFDGSVR** |
| **P19338** | **NuCleolin** | **AIRLELQGPR** |
| **P19338** | **NuCleolin** | **NSTWSGESK** |
| **P19338** | **NuCleolin** | **EVFEDAAEIR** |
| **P19823** | **Inter-alpha-trypsin inhibitor heavy Chain H2** | **IYGNQDTSSQLK** |
| **P19823** | **Inter-alpha-trypsin inhibitor heavy Chain H2** | **IQPSGGTNINEALLR** |
| **P19823** | **Inter-alpha-trypsin inhibitor heavy Chain H2** | **RLSNENHGIAQR** |
| **P19823** | **Inter-alpha-trypsin inhibitor heavy Chain H2** | **SSALDMENFR** |
| **P19823** | **Inter-alpha-trypsin inhibitor heavy Chain H2** | **LWAYLTINQLLAER** |
| **P19823** | **Inter-alpha-trypsin inhibitor heavy Chain H2** | **VVNNSPQPQNVVFDVQIPK** |
| **P19823** | **Inter-alpha-trypsin inhibitor heavy Chain H2** | **AGELEVFNGYFVHFFAPDNLDPIPK** |
| **P19823** | **Inter-alpha-trypsin inhibitor heavy Chain H2** | **ALYAQAR** |
| **P19823** | **Inter-alpha-trypsin inhibitor heavy Chain H2** | **TILDDLRAEDHFSVIDFNQNIR** |
| **P19823** | **Inter-alpha-trypsin inhibitor heavy Chain H2** | **FYNQVSTPLLR** |
| **P19823** | **Inter-alpha-trypsin inhibitor heavy Chain H2** | **SSALDMENFRTEVNVLPGAK** |
| **P19823** | **Inter-alpha-trypsin inhibitor heavy Chain H2** | **IYLQPGR** |
| **P19823** | **Inter-alpha-trypsin inhibitor heavy Chain H2** | **KFYNQVSTPLLR** |
| **P19823** | **Inter-alpha-trypsin inhibitor heavy Chain H2** | **LSNENHGIAQR** |
| **P19823** | **Inter-alpha-trypsin inhibitor heavy Chain H2** | **MATTMIQSK** |
| **P19823** | **Inter-alpha-trypsin inhibitor heavy Chain H2** | **LGSYEHR** |
| **P19823** | **Inter-alpha-trypsin inhibitor heavy Chain H2** | **IYGNQDTSSQLKK** |
| **P19823** | **Inter-alpha-trypsin inhibitor heavy Chain H2** | **QTVEAMK** |
| **P19823** | **Inter-alpha-trypsin inhibitor heavy Chain H2** | **TWRNDLISATK** |
| **P19823** | **Inter-alpha-trypsin inhibitor heavy Chain H2** | **MLADAPPQDPSCCSGALYYGSK** |
| **P19823** | **Inter-alpha-trypsin inhibitor heavy Chain H2** | **KLGSYEHR** |
| **P19823** | **Inter-alpha-trypsin inhibitor heavy Chain H2** | **MKQTVEAMK** |
| **P19823** | **Inter-alpha-trypsin inhibitor heavy Chain H2** | **FLHVPDTFEGHFDGVPVISK** |
| **P19823** | **Inter-alpha-trypsin inhibitor heavy Chain H2** | **AHVSFKPTVAQQR** |
| **P19823** | **Inter-alpha-trypsin inhibitor heavy Chain H2** | **SILQMSLDHHIVTPLTSLVIENEAGDER** |
| **P19823** | **Inter-alpha-trypsin inhibitor heavy Chain H2** | **ETAVDGELVVLYDVK** |
| **P19823** | **Inter-alpha-trypsin inhibitor heavy Chain H2** | **NVQFNYPHTSVTDVTQNNFHNYFGGSEIVVAGK** |
| **P19823** | **Inter-alpha-trypsin inhibitor heavy Chain H2** | **NDLISATK** |
| **P19823** | **Inter-alpha-trypsin inhibitor heavy Chain H2** | **HADPDFTR** |
| **P19971** | **Thymidine phOSphorylase** | **DVTATVDSLPLITASILSK** |
| **P19971** | **Thymidine phOSphorylase** | **VAAALDDGSALGR** |
| **P19971** | **Thymidine phOSphorylase** | **MLAAQGVDPGLAR** |
| **P19971** | **Thymidine phOSphorylase** | **TLVGVGASLGLR** |
| **P19971** | **Thymidine phOSphorylase** | **VHRDGPALSGPQSR** |
| **P19971** | **Thymidine phOSphorylase** | **AREQEELLAPADGTVELVR** |
| **P19971** | **Thymidine phOSphorylase** | **VSLVLAPALAACGCK** |
| **P19971** | **Thymidine phOSphorylase** | **VAAALTAMDKPLGR** |
| **P19971** | **Thymidine phOSphorylase** | **FGGAAVFPNQEQAR** |
| **P19971** | **Thymidine phOSphorylase** | **ALCSGSPAER** |
| **P21333** | **Filamin-A** | **AFGPGLQGGSAGSPAR** |
| **P21333** | **Filamin-A** | **IANLQTDLSDGLR** |
| **P21333** | **Filamin-A** | **VTAQGPGLEPSGNIANK** |
| **P21333** | **Filamin-A** | **GAGTGGLGLAVEGPSEAK** |
| **P21333** | **Filamin-A** | **TGVAVNKPAEFTVDAK** |
| **P21333** | **Filamin-A** | **VNVGAGSHPNK** |
| **P21333** | **Filamin-A** | **ALGALVDSCAPGLCPDWDSWDASKPVTNAR** |
| **P21333** | **Filamin-A** | **TPCEEILVK** |
| **P21333** | **Filamin-A** | **YGGDEIPFSPYR** |
| **P21333** | **Filamin-A** | **IVGPSGAAVPCKVEPGLGADNSVVR** |
| **P21333** | **Filamin-A** | **SPFSVAVSPSLDLSK** |
| **P21333** | **Filamin-A** | **HGGKAPLR** |
| **P21333** | **Filamin-A** | **VGSAADIPINISETDLSLLTATVVPPSGREEPCLLK** |
| **P21333** | **Filamin-A** | **FGGEHVPNSPFQVTALAGDQPSVQPPLR** |
| **P21333** | **Filamin-A** | **VKVEPSHDASK** |
| **P21333** | **Filamin-A** | **ALTQTGGPHVK** |
| **P21333** | **Filamin-A** | **IQQNTFTR** |
| **P21333** | **Filamin-A** | **VGSAADIPINISETDLSLLTATVVPPSGR** |
| **P21741** | **Midkine** | **YKFENWGACDGGTGTK** |
| **P21741** | **Midkine** | **YNAQCQETIR** |
| **P21741** | **Midkine** | **KGGPGSECAEWAWGPCTPSSK** |
| **P21926** | **CD9 antigen** | **AIHYALNCCGLAGGVEQFISDICPK** |
| **P21926** | **CD9 antigen** | **DVLETFTVK** |
| **P21926** | **CD9 antigen** | **KDVLETFTVK** |
| **P22352** | **Glutathione peroxidase 3** | **FLVGPDGIPIMR** |
| **P22352** | **Glutathione peroxidase 3** | **NSCPPTSELLGTSDR** |
| **P22352** | **Glutathione peroxidase 3** | **MDILSYMR** |
| **P22352** | **Glutathione peroxidase 3** | **YVRPGGGFVPNFQLFEK** |
| **P22392** | **NuCleOSide diphOSphate kinase B** | **YMNSGPVVAMVWEGLNVVK** |
| **P22392** | **NuCleOSide diphOSphate kinase B** | **DRPFFPGLVK** |
| **P22392** | **NuCleOSide diphOSphate kinase B** | **NIIHGSDSVK** |
| **P22392** | **NuCleOSide diphOSphate kinase B** | **EISLWFKPEELVDYK** |
| **P22392** | **NuCleOSide diphOSphate kinase B** | **SCAHDWVYE** |
| **P22626** | **Heterogeneous nuClear ribonuCleoproteins A2/B1** | **QEMQEVQSSR** |
| **P22626** | **Heterogeneous nuClear ribonuCleoproteins A2/B1** | **ALSRQEMQEVQSSR** |
| **P22626** | **Heterogeneous nuClear ribonuCleoproteins A2/B1** | **EESGKPGAHVTVK** |
| **P22626** | **Heterogeneous nuClear ribonuCleoproteins A2/B1** | **GGNFGFGDSR** |
| **P22626** | **Heterogeneous nuClear ribonuCleoproteins A2/B1** | **GGGGNFGPGPGSNFR** |
| **P22626** | **Heterogeneous nuClear ribonuCleoproteins A2/B1** | **LFIGGLSFETTEESLR** |
| **P23142** | **Fibulin-1** | **GYQLSDVDGVTCEDIDECALPTGGHICSYR** |
| **P23142** | **Fibulin-1** | **DTGDEVVCSCFVGYQLLSDGVSCEDVNECITGSHSCR** |
| **P23142** | **Fibulin-1** | **LGESCINTVGSFR** |
| **P23142** | **Fibulin-1** | **SQETGDLDVGGLQETDKIIEVEEEQEDPYLNDR** |
| **P23142** | **Fibulin-1** | **TGYYFDGISR** |
| **P23142** | **Fibulin-1** | **CVDVDECAPPAEPCGK** |
| **P23142** | **Fibulin-1** | **RGYQLSDVDGVTCEDIDECALPTGGHICSYR** |
| **P23142** | **Fibulin-1** | **GYHLNEEGTRCVDVDECAPPAEPCGK** |
| **P23142** | **Fibulin-1** | **DIDECESGIHNCLPDFICQNTLGSFR** |
| **P23142** | **Fibulin-1** | **DCSLPYATESK** |
| **P23142** | **Fibulin-1** | **MCVDVNECQR** |
| **P23142** | **Fibulin-1** | **AITPPHPASQANIIFDITEGNLR** |
| **P23142** | **Fibulin-1** | **SAATLQQEK** |
| **P23142** | **Fibulin-1** | **CCHCCLLGR** |
| **P23142** | **Fibulin-1** | **GYHLNEEGTR** |
| **P23142** | **Fibulin-1** | **MVQEQCCHSQLEELHCATGISLANEQDR** |
| **P23142** | **Fibulin-1** | **RCCHCCLLGR** |
| **P23526** | **AdenOSylhomoCysteinase** | **VPAINVNDSVTK** |
| **P23526** | **AdenOSylhomoCysteinase** | **ALDIAENEMPGLMR** |
| **P23526** | **AdenOSylhomoCysteinase** | **GISEETTTGVHNLYK** |
| **P23526** | **AdenOSylhomoCysteinase** | **KLDEAVAEAHLGK** |
| **P23526** | **AdenOSylhomoCysteinase** | **RATDVMIAGK** |
| **P23526** | **AdenOSylhomoCysteinase** | **SKFDNLYGCR** |
| **P23526** | **AdenOSylhomoCysteinase** | **IILLAEGR** |
| **P23526** | **AdenOSylhomoCysteinase** | **DGPLNMILDDGGDLTNLIHTK** |
| **P23526** | **AdenOSylhomoCysteinase** | **YPQLLPGIR** |
| **P23526** | **AdenOSylhomoCysteinase** | **AGIPVYAWK** |
| **P23526** | **AdenOSylhomoCysteinase** | **VNIKPQVDR** |
| **P24821** | **TenasCin** | **TTLTGLRPGTEYGIGVSAVKEDK** |
| **P24821** | **TenasCin** | **LPVGSQCSVDLESASGEK** |
| **P24821** | **TenasCin** | **TVSGNTVEYALTDLEPATEYTLR** |
| **P24821** | **TenasCin** | **LIPGVEYLVSIIAMK** |
| **P24821** | **TenasCin** | **WQPAIATVDSYVISYTGEK** |
| **P24821** | **TenasCin** | **NMNKEDEGEITK** |
| **P24821** | **TenasCin** | **GLEPGQEYNVLLTAEK** |
| **P24821** | **TenasCin** | **ITYVPITGGTPSMVTVDGTK** |
| **P24821** | **TenasCin** | **ESNPATINAATELDTPK** |
| **P24821** | **TenasCin** | **LSWTADEGVFDNFVLK** |
| **P24821** | **TenasCin** | **DVTDTTALITWFKPLAEIDGIELTYGIK** |
| **P24821** | **TenasCin** | **ETFTTGLDAPR** |
| **P24821** | **TenasCin** | **ITAQGQYELR** |
| **P24821** | **TenasCin** | **INIPR** |
| **P24821** | **TenasCin** | **APTAQVESFR** |
| **P26038** | **Moesin** | **IAQDLEMYGVNYFSIK** |
| **P26038** | **Moesin** | **AQMVQEDLEK** |
| **P26038** | **Moesin** | **ISQLEMAR** |
| **P26038** | **Moesin** | **TQEQLALEMAELTAR** |
| **P26038** | **Moesin** | **FYPEDVSEELIQDITQR** |
| **P26038** | **Moesin** | **EVWFFGLQYQDTK** |
| **P26038** | **Moesin** | **GMLREDAVLEYLK** |
| **P26038** | **Moesin** | **EGILNDDIYCPPETAVLLASYAVQSK** |
| **P26038** | **Moesin** | **EALLQASR** |
| **P26038** | **Moesin** | **TAMSTPHVAEPAENEQDEQDENGAEASADLRADAMAK** |
| **P26038** | **Moesin** | **ALTSELANARDESK** |
| **P26038** | **Moesin** | **KAQQELEEQTR** |
| **P26038** | **Moesin** | **AQQELEEQTR** |
| **P26038** | **Moesin** | **LNKDQWEER** |
| **P26038** | **Moesin** | **ESEAVEWQQK** |
| **P26038** | **Moesin** | **ALELEQER** |
| **P26038** | **Moesin** | **YGDFNK** |
| **P26038** | **Moesin** | **QRIDEFESM** |
| **P26447** | **Protein S100-A4** | **RTDEAAFQK** |
| **P28838** | **CytOSol aminopeptidase** | **LNLPINIIGLAPLCENMPSGK** |
| **P28838** | **CytOSol aminopeptidase** | **GVLFASGQNLAR** |
| **P28838** | **CytOSol aminopeptidase** | **GSPNANEPPLVFVGK** |
| **P28838** | **CytOSol aminopeptidase** | **ADMGGAATICSAIVSAAK** |
| **P28838** | **CytOSol aminopeptidase** | **SWIEEQAMGSFLSVAK** |
| **P28838** | **CytOSol aminopeptidase** | **TLIEFLLR** |
| **P28838** | **CytOSol aminopeptidase** | **EKEDDVPQFTSAGENFDK** |
| **P28838** | **CytOSol aminopeptidase** | **QLMETPANEMTPTR** |
| **P28838** | **CytOSol aminopeptidase** | **ASANMDLMR** |
| **P28838** | **CytOSol aminopeptidase** | **SAGACTAAAFLK** |
| **P28838** | **CytOSol aminopeptidase** | **LYGSGDQEAWQK** |
| **P28838** | **CytOSol aminopeptidase** | **AAGIDEQENWHEGKENIR** |
| **P28838** | **CytOSol aminopeptidase** | **AAGIDEQENWHEGK** |
| **P28838** | **CytOSol aminopeptidase** | **LRETLNISGPPLK** |
| **P28838** | **CytOSol aminopeptidase** | **GLVLGIYSK** |
| **P29508** | **Serpin B3** | **INSWVESQTNEK** |
| **P29508** | **Serpin B3** | **SGNVHHQFQK** |
| **P29966** | **Myristoylated alanine-riCh C-kinase substrate** | **AEDGATPSPSNETPK** |
| **P29966** | **Myristoylated alanine-riCh C-kinase substrate** | **GEAAAERPGEAAVASSPSK** |
| **P29966** | **Myristoylated alanine-riCh C-kinase substrate** | **AAEEPSKVEEK** |
| **P29966** | **Myristoylated alanine-riCh C-kinase substrate** | **TAAKGEAAAERPGEAAVASSPSK** |
| **P29966** | **Myristoylated alanine-riCh C-kinase substrate** | **EAPAEGEAAEPGSPTAAEGEAASAASSTSSPK** |
| **P30041** | **Peroxiredoxin-6** | **LIALSIDSVEDHLAWSK** |
| **P30041** | **Peroxiredoxin-6** | **PGGLLLGDVAPNFEANTTVGR** |
| **P30041** | **Peroxiredoxin-6** | **IRFHDFLGDSWGILFSHPR** |
| **P30041** | **Peroxiredoxin-6** | **DINAYNCEEPTEK** |
| **P30041** | **Peroxiredoxin-6** | **NFDEILR** |
| **P30041** | **Peroxiredoxin-6** | **FHDFLGDSWGILFSHPR** |
| **P30041** | **Peroxiredoxin-6** | **LSILYPATTGR** |
| **P30041** | **Peroxiredoxin-6** | **DFTPVCTTELGR** |
| **P30041** | **Peroxiredoxin-6** | **VATPVDWKDGDSVMVLPTIPEEEAK** |
| **P30041** | **Peroxiredoxin-6** | **VVFVFGPDKK** |
| **P30043** | **Flavin reduCtase** | **TVAGQDAVIVLLGTRNDLSPTTVMSEGAR** |
| **P30043** | **Flavin reduCtase** | **NDLSPTTVMSEGAR** |
| **P30043** | **Flavin reduCtase** | **LQAVTDDHIR** |
| **P30043** | **Flavin reduCtase** | **LPSEGPRPAHVVVGDVLQAADVDK** |
| **P30043** | **Flavin reduCtase** | **YVAVMPPHIGDQPLTGAYTVTLDGR** |
| **P30043** | **Flavin reduCtase** | **DSSRLPSEGPRPAHVVVGDVLQAADVDK** |
| **P30043** | **Flavin reduCtase** | **TVAGQDAVIVLLGTR** |
| **P30043** | **Flavin reduCtase** | **CLTTDEYDGHSTYPSHQYQ** |
| **P30044** | **Peroxiredoxin-5, mitoChondrial** | **VNLAELFK** |
| **P30044** | **Peroxiredoxin-5, mitoChondrial** | **LLADPTGAFGKETDLLLDDSLVSIFGNR** |
| **P30044** | **Peroxiredoxin-5, mitoChondrial** | **THLPGFVEQAEALK** |
| **P30044** | **Peroxiredoxin-5, mitoChondrial** | **FSMVVQDGIVK** |
| **P30044** | **Peroxiredoxin-5, mitoChondrial** | **ALNVEPDGTGLTCSLAPNIISQL** |
| **P30044** | **Peroxiredoxin-5, mitoChondrial** | **ETDLLLDDSLVSIFGNR** |
| **P30044** | **Peroxiredoxin-5, mitoChondrial** | **VGDAIPAVEVFEGEPGNK** |
| **P30044** | **Peroxiredoxin-5, mitoChondrial** | **VGDAIPAVEVFEGEPGNKVNLAELFK** |
| **P30086** | **PhOSphatidylethanolamine-binding protein 1** | **GNDISSGTVLSDYVGSGPPK** |
| **P30086** | **PhOSphatidylethanolamine-binding protein 1** | **APVAGTCYQAEWDDYVPK** |
| **P30086** | **PhOSphatidylethanolamine-binding protein 1** | **WSGPLSLQEVDEQPQHPLHVTYAGAAVDELGK** |
| **P30086** | **PhOSphatidylethanolamine-binding protein 1** | **LYTLVLTDPDAPSR** |
| **P30086** | **PhOSphatidylethanolamine-binding protein 1** | **NRPTSISWDGLDSGK** |
| **P30086** | **PhOSphatidylethanolamine-binding protein 1** | **CDEPILSNR** |
| **P30086** | **PhOSphatidylethanolamine-binding protein 1** | **YVWLVYEQDRPLK** |
| **P30086** | **PhOSphatidylethanolamine-binding protein 1** | **LYEQLSGK** |
| **P30086** | **PhOSphatidylethanolamine-binding protein 1** | **YREWHHFLVVNMK** |
| **P30086** | **PhOSphatidylethanolamine-binding protein 1** | **GNDISSGTVLSDYVGSGPPKGTGLHR** |
| **P30086** | **PhOSphatidylethanolamine-binding protein 1** | **PVDLSK** |
| **P30153** | **Serine/threonine-protein phOSphatase 2A 65 kDa regulatory subunit A alpha isoform** | **IGPILDNSTLQSEVKPILEK** |
| **P30153** | **Serine/threonine-protein phOSphatase 2A 65 kDa regulatory subunit A alpha isoform** | **SALASVIMGLSPILGK** |
| **P30153** | **Serine/threonine-protein phOSphatase 2A 65 kDa regulatory subunit A alpha isoform** | **LNIISNLDCVNEVIGIR** |
| **P31943** | **Heterogeneous nuClear ribonuCleoprotein H** | **STGEAFVQFASQEIAEK** |
| **P31943** | **Heterogeneous nuClear ribonuCleoprotein H** | **HTGPNSPDTANDGFVR** |
| **P31947** | **14-3-3 protein sigma** | **VETELQGVCDTVLGLLDSHLIK** |
| **P31947** | **14-3-3 protein sigma** | **SNEEGSEEKGPEVR** |
| **P31947** | **14-3-3 protein sigma** | **YLAEVATGDDKK** |
| **P31947** | **14-3-3 protein sigma** | **YLAEVATGDDK** |
| **P31947** | **14-3-3 protein sigma** | **GAVEKGEELSCEER** |
| **P31947** | **14-3-3 protein sigma** | **LAEQAERYEDMAAFMK** |
| **P31949** | **Protein S100-A11** | **KLDTNSDGQLDFSEFLNLIGGLAmACHDSFLK** |
| **P31949** | **Protein S100-A11** | **TEFLSFMNTELAAFTK** |
| **P31949** | **Protein S100-A11** | **CIESLIAVFQK** |
| **P31949** | **Protein S100-A11** | **DGYNYTLSKTEFLSFMNTELAAFTK** |
| **P31949** | **Protein S100-A11** | **NQKDPGVLDR** |
| **P31949** | **Protein S100-A11** | **DPGVLDR** |
| **P31949** | **Protein S100-A11** | **DGYNYTLSK** |
| **P31949** | **Protein S100-A11** | **YAGKDGYNYTLSK** |
| **P35222** | **Catenin beta-1** | **LLNDEDQVVVNK** |
| **P35268** | **60S ribOSomal protein L22** | **AGNLGGGVVTIER** |
| **P35268** | **60S ribOSomal protein L22** | **FTLDCTHPVEDGIMDAANFEQFLQER** |
| **P36222** | **Chitinase-3-like protein 1** | **TLLSVGGWNFGSQR** |
| **P36222** | **Chitinase-3-like protein 1** | **ILGQQVPYATK** |
| **P36222** | **Chitinase-3-like protein 1** | **GNQWVGYDDQESVK** |
| **P36222** | **Chitinase-3-like protein 1** | **SFTLASSETGVGAPISGPGIPGR** |
| **P36222** | **Chitinase-3-like protein 1** | **LVMGIPTFGR** |
| **P39060** | **Collagen alpha-1(XVIII) Chain** | **TEAPSATGQASSLLGGR** |
| **P39060** | **Collagen alpha-1(XVIII) Chain** | **GADFQCFQQAR** |
| **P39060** | **Collagen alpha-1(XVIII) Chain** | **LQDLYSIVR** |
| **P40121** | **MaCrophage-Capping protein** | **QAALQVAEGFISR** |
| **P40121** | **MaCrophage-Capping protein** | **EVQGNESDLFMSYFPR** |
| **P40121** | **MaCrophage-Capping protein** | **VSDATGQMNLTK** |
| **P40121** | **MaCrophage-Capping protein** | **YQEGGVESAFHK** |
| **P40121** | **MaCrophage-Capping protein** | **AQVEIVTDGEEPAEMIQVLGPKPALK** |
| **P40121** | **MaCrophage-Capping protein** | **EGNPEEDLTADK** |
| **P40261** | **NiCotinamide N-methyltransferase** | **NLGSLLKPGGFLVIMDALK** |
| **P42330** | **Aldo-keto reduCtase family 1 member C3** | **WVDPNSPVLLEDPVLCALAK** |
| **P42574** | **Caspase-3** | **SGTDVDAANLR** |
| **P43490** | **NiCotinamide phOSphoribOSyltransferase** | **DAFEHIVTQFSSVPVSVVSDSYDIYNACEK** |
| **P43490** | **NiCotinamide phOSphoribOSyltransferase** | **VIQGDGVDINTLQEIVEGMK** |
| **P47756** | **F-aCtin-Capping protein subunit beta** | **SGSGTMNLGGSLTR** |
| **P47756** | **F-aCtin-Capping protein subunit beta** | **STLNEIYFGK** |
| **P47756** | **F-aCtin-Capping protein subunit beta** | **SPWSNKYDPPLEDGAMPSAR** |
| **P47756** | **F-aCtin-Capping protein subunit beta** | **QMEKDETVSDCSPHIANIGR** |
| **P47756** | **F-aCtin-Capping protein subunit beta** | **LVEDMENK** |
| **P47756** | **F-aCtin-Capping protein subunit beta** | **NLSDLIDLVPSLCEDLLSSVDQPLK** |
| **P47756** | **F-aCtin-Capping protein subunit beta** | **RLPPQQIEK** |
| **P49327** | **Fatty aCid synthase** | **ALGLGVEQLPVVFEDVVLHQATILPK** |
| **P50454** | **Serpin H1** | **LYGPSSVSFADDFVR** |
| **P50454** | **Serpin H1** | **SAGLAFSLYQAMAK** |
| **P50454** | **Serpin H1** | **DTQSGSLLFIGR** |
| **P50454** | **Serpin H1** | **DQAVENILVSPVVVASSLGLVSLGGK** |
| **P50454** | **Serpin H1** | **AVLSAEQLRDEEVHAGLGELLR** |
| **P55060** | **Exportin-2** | **IIIPEIQK** |
| **P55072** | **Transitional endoplasmiC retiCulum ATPase** | **IVSQLLTLMDGLK** |
| **P55072** | **Transitional endoplasmiC retiCulum ATPase** | **KYEMFAQTLQQSR** |
| **P55072** | **Transitional endoplasmiC retiCulum ATPase** | **QAAPCVLFFDELDSIAK** |
| **P55072** | **Transitional endoplasmiC retiCulum ATPase** | **NAPAIIFIDELDAIAPK** |
| **P55072** | **Transitional endoplasmiC retiCulum ATPase** | **LGDVISIQPCPDVK** |
| **P55072** | **Transitional endoplasmiC retiCulum ATPase** | **AVANETGAFFFLINGPEIMSK** |
| **P55072** | **Transitional endoplasmiC retiCulum ATPase** | **LIVDEAINEDNSVVSLSQPK** |
| **P55072** | **Transitional endoplasmiC retiCulum ATPase** | **ARQAAPCVLFFDELDSIAK** |
| **P55072** | **Transitional endoplasmiC retiCulum ATPase** | **LAGESESNLR** |
| **P55072** | **Transitional endoplasmiC retiCulum ATPase** | **WALSQSNPSALR** |
| **P55072** | **Transitional endoplasmiC retiCulum ATPase** | **LDQLIYIPLPDEK** |
| **P55072** | **Transitional endoplasmiC retiCulum ATPase** | **MTNGFSGADLTEICQR** |
| **P55072** | **Transitional endoplasmiC retiCulum ATPase** | **GGNIGDGGGAADR** |
| **P55072** | **Transitional endoplasmiC retiCulum ATPase** | **ELQELVQYPVEHPDK** |
| **P55072** | **Transitional endoplasmiC retiCulum ATPase** | **LAGESESNLRK** |
| **P55072** | **Transitional endoplasmiC retiCulum ATPase** | **DVDLEFLAK** |
| **P55072** | **Transitional endoplasmiC retiCulum ATPase** | **QTNPSAMEVEEDDPVPEIRR** |
| **P55072** | **Transitional endoplasmiC retiCulum ATPase** | **VRLGDVISIQPCPDVK** |
| **P55072** | **Transitional endoplasmiC retiCulum ATPase** | **GPELLTmWFGESEANVR** |
| **P60174** | **TriOSephOSphate isomerase** | **QSLGELIGTLNAAK** |
| **P60174** | **TriOSephOSphate isomerase** | **VVLAYEPVWAIGTGK** |
| **P60174** | **TriOSephOSphate isomerase** | **SNVSDAVAQSTR** |
| **P60174** | **TriOSephOSphate isomerase** | **VTNGAFTGEISPGMIK** |
| **P60174** | **TriOSephOSphate isomerase** | **TATPQQAQEVHEK** |
| **P60174** | **TriOSephOSphate isomerase** | **HVFGESDELIGQK** |
| **P60174** | **TriOSephOSphate isomerase** | **RHVFGESDELIGQK** |
| **P60174** | **TriOSephOSphate isomerase** | **IIYGGSVTGATCK** |
| **P60174** | **TriOSephOSphate isomerase** | **DCGATWVVLGHSER** |
| **P60174** | **TriOSephOSphate isomerase** | **IAVAAQNCYK** |
| **P60174** | **TriOSephOSphate isomerase** | **ELASQPDVDGFLVGGASLKPEFVDIINAK** |
| **P60174** | **TriOSephOSphate isomerase** | **ELASQPDVDGFLVGGASLKPEFVDIINAKQ** |
| **P60174** | **TriOSephOSphate isomerase** | **VAHALAEGLGVIACIGEKLDER** |
| **P60174** | **TriOSephOSphate isomerase** | **VAHALAEGLGVIACIGEK** |
| **P60174** | **TriOSephOSphate isomerase** | **KQSLGELIGTLNAAK** |
| **P60174** | **TriOSephOSphate isomerase** | **VPADTEVVCAPPTAYIDFAR** |
| **P60174** | **TriOSephOSphate isomerase** | **QSLGELIGTLNAAKVPADTEVVCAPPTAYIDFAR** |
| **P60174** | **TriOSephOSphate isomerase** | **VIADNVKDWSK** |
| **P60174** | **TriOSephOSphate isomerase** | **LDEREAGITEK** |
| **P60174** | **TriOSephOSphate isomerase** | **FFVGGNWK** |
| **P61604** | **10 kDa heat shoCk protein, mitoChondrial** | **VLQATVVAVGSGSK** |
| **P61604** | **10 kDa heat shoCk protein, mitoChondrial** | **GKGGEIQPVSVK** |
| **P62851** | **40S ribOSomal protein S25** | **AALQELLSK** |
| **P62937** | **Peptidyl-prolyl Cis-trans isomerase A** | **VKEGMNIVEAMER** |
| **P62937** | **Peptidyl-prolyl Cis-trans isomerase A** | **VNPTVFFDIAVDGEPLGR** |
| **P62937** | **Peptidyl-prolyl Cis-trans isomerase A** | **KITIADCGQLE** |
| **P62937** | **Peptidyl-prolyl Cis-trans isomerase A** | **SIYGEKFEDENFILK** |
| **P62937** | **Peptidyl-prolyl Cis-trans isomerase A** | **HTGPGILSMANAGPNTNGSQFFICTAK** |
| **P62937** | **Peptidyl-prolyl Cis-trans isomerase A** | **VSFELFADK** |
| **P62937** | **Peptidyl-prolyl Cis-trans isomerase A** | **VSFELFADKVPK** |
| **P62937** | **Peptidyl-prolyl Cis-trans isomerase A** | **TEWLDGK** |
| **P62937** | **Peptidyl-prolyl Cis-trans isomerase A** | **EGMNIVEAMER** |
| **P62937** | **Peptidyl-prolyl Cis-trans isomerase A** | **IIPGFMCQGGDFTR** |
| **P62937** | **Peptidyl-prolyl Cis-trans isomerase A** | **ITIADCGQLE** |
| **P62937** | **Peptidyl-prolyl Cis-trans isomerase A** | **FEDENFILK** |
| **P67936** | **TropomyOSin alpha-4 Chain** | **LATALQKLEEAEK** |
| **P67936** | **TropomyOSin alpha-4 Chain** | **IQLVEEELDRAQER** |
| **P67936** | **TropomyOSin alpha-4 Chain** | **LAQAKEENVGLHQTLDQTLNELNCI** |
| **P67936** | **TropomyOSin alpha-4 Chain** | **CGDLEEELK** |
| **P67936** | **TropomyOSin alpha-4 Chain** | **IQALQQQADEAEDRAQGLQR** |
| **P67936** | **TropomyOSin alpha-4 Chain** | **EENVGLHQTLDQTLNELNCI** |
| **P67936** | **TropomyOSin alpha-4 Chain** | **SLEAASEK** |
| **P67936** | **TropomyOSin alpha-4 Chain** | **IQALQQQADEAEDR** |
| **P67936** | **TropomyOSin alpha-4 Chain** | **EKAEGDVAALNR** |
| **P80188** | **Neutrophil gelatinase-assoCiated lipoCalin** | **VPLQQNFQDNQFQGK** |
| **P80188** | **Neutrophil gelatinase-assoCiated lipoCalin** | **SLGLPENHIVFPVPIDQCIDG** |
| **P80188** | **Neutrophil gelatinase-assoCiated lipoCalin** | **VVSTNYNQHAMVFFK** |
| **P80188** | **Neutrophil gelatinase-assoCiated lipoCalin** | **TFVPGCQPGEFTLGNIK** |
| **P80188** | **Neutrophil gelatinase-assoCiated lipoCalin** | **KCDYWIR** |
| **P80188** | **Neutrophil gelatinase-assoCiated lipoCalin** | **MYATIYELK** |
| **Q01469** | **Fatty aCid-binding protein, epidermal** | **LVVECVMNNVTCTR** |
| **Q01469** | **Fatty aCid-binding protein, epidermal** | **TQTVCNFTDGALVQHQEWDGK** |
| **Q01469** | **Fatty aCid-binding protein, epidermal** | **MGAMAKPDCIITCDGK** |
| **Q01469** | **Fatty aCid-binding protein, epidermal** | **TTQFSCTLGEK** |
| **Q01469** | **Fatty aCid-binding protein, epidermal** | **ELGVGIALRK** |
| **Q01469** | **Fatty aCid-binding protein, epidermal** | **TQTVCNFTDGALVQHQEWDGKESTITR** |
| **Q01469** | **Fatty aCid-binding protein, epidermal** | **TTQFSCTLGEKFEETTADGR** |
| **Q01469** | **Fatty aCid-binding protein, epidermal** | **ELGVGIALR** |
| **Q01518** | **Adenylyl CyClase-assoCiated protein 1** | **ALLVTASQCQQPAENK** |
| **Q01518** | **Adenylyl CyClase-assoCiated protein 1** | **NSLDCEIVSAK** |
| **Q01518** | **Adenylyl CyClase-assoCiated protein 1** | **KLGLVFDDVVGIVEIINSK** |
| **Q01518** | **Adenylyl CyClase-assoCiated protein 1** | **ALLVTASQCQQPAENKLSDLLAPISEQIK** |
| **Q01518** | **Adenylyl CyClase-assoCiated protein 1** | **EMNDAAMFYTNR** |
| **Q01518** | **Adenylyl CyClase-assoCiated protein 1** | **AGAAPYVQAFDSLLAGPVAEYLK** |
| **Q01995** | **Transgelin** | **TDMFQTVDLFEGK** |
| **Q01995** | **Transgelin** | **EFTESQLQEGK** |
| **Q01995** | **Transgelin** | **TLMALGSLAVTK** |
| **Q01995** | **Transgelin** | **KYDEELEER** |
| **Q01995** | **Transgelin** | **QMEQVAQFLK** |
| **Q01995** | **Transgelin** | **LVNSLYPDGSKPVKVPENPPSMVFK** |
| **Q06830** | **Peroxiredoxin-1** | **LVQAFQFTDK** |
| **Q06830** | **Peroxiredoxin-1** | **HGEVCPAGWKPGSDTIKPDVQK** |
| **Q06830** | **Peroxiredoxin-1** | **ADEGISFR** |
| **Q06830** | **Peroxiredoxin-1** | **RTIAQDYGVLK** |
| **Q06830** | **Peroxiredoxin-1** | **YVVFFFYPLDFTFVCPTEIIAFSDRAEEFK** |
| **Q06830** | **Peroxiredoxin-1** | **GLFIIDDK** |
| **Q06830** | **Peroxiredoxin-1** | **QGGLGPMNIPLVSDPK** |
| **Q06830** | **Peroxiredoxin-1** | **SVDETLR** |
| **Q06830** | **Peroxiredoxin-1** | **ATAVMPDGQFK** |
| **Q06830** | **Peroxiredoxin-1** | **TIAQDYGVLK** |
| **Q06830** | **Peroxiredoxin-1** | **QGGLGPMNIPLVSDPKR** |
| **Q06830** | **Peroxiredoxin-1** | **DISLSDYK** |
| **Q06830** | **Peroxiredoxin-1** | **KLNCQVIGASVDSHFCHLAWVNTPK** |
| **Q06830** | **Peroxiredoxin-1** | **KQGGLGPMNIPLVSDPK** |
| **Q07654** | **Trefoil faCtor 3** | **IPGVPWCFKPLQEAECTF** |
| **Q07654** | **Trefoil faCtor 3** | **GCCFDSR** |
| **Q12906** | **Interleukin enhanCer-binding faCtor 3** | **VLAGETLSVNDPPDVLDR** |
| **Q13421** | **Mesothelin** | **AREIDESLIFYK** |
| **Q13421** | **Mesothelin** | **VNAIPFTYEQLDVLK** |
| **Q13421** | **Mesothelin** | **EIDESLIFYK** |
| **Q13421** | **Mesothelin** | **LRTDAVLPLTVAEVQK** |
| **Q13938** | **CalCyphOSin** | **LGLVLDQAEAEGVCR** |
| **Q13938** | **CalCyphOSin** | **SLDADEFRQGLAK** |
| **Q13938** | **CalCyphOSin** | **NGSGTLDLEEFLR** |
| **Q13938** | **CalCyphOSin** | **FLDNFDSSEK** |
| **Q13938** | **CalCyphOSin** | **EAVIAAAFAK** |
| **Q13938** | **CalCyphOSin** | **SGDGVVTVDDLRGVYSGR** |
| **Q13938** | **CalCyphOSin** | **VRSGEWTEDEVLR** |
| **Q13938** | **CalCyphOSin** | **SGEWTEDEVLR** |
| **Q13938** | **CalCyphOSin** | **SGDGVVTVDDLR** |
| **Q13938** | **CalCyphOSin** | **ALRPPMSQAR** |
| **Q13938** | **CalCyphOSin** | **GASGIQGLAR** |
| **Q14103** | **Heterogeneous nuClear ribonuCleoprotein D0** | **SRGFGFVLFK** |
| **Q14103** | **Heterogeneous nuClear ribonuCleoprotein D0** | **IFVGGLSPDTPEEK** |
| **Q14103** | **Heterogeneous nuClear ribonuCleoprotein D0** | **FGEVVDCTLKLDPITGR** |
| **Q14103** | **Heterogeneous nuClear ribonuCleoprotein D0** | **HSEAATAQREEWK** |
| **Q14103** | **Heterogeneous nuClear ribonuCleoprotein D0** | **FGEVVDCTIK** |
| **Q14103** | **Heterogeneous nuClear ribonuCleoprotein D0** | **IDASKNEEDEGHSNSSPR** |
| **Q14103** | **Heterogeneous nuClear ribonuCleoprotein D0** | **KYHNVGLSK** |
| **Q14508** | **WAP four-disulfide Core domain protein 2** | **CCSAGCATFCSLPNDK** |
| **Q14508** | **WAP four-disulfide Core domain protein 2** | **EGSCPQVNINFPQLGLCR** |
| **Q14508** | **WAP four-disulfide Core domain protein 2** | **DQCQVDSQCPGQMK** |
| **Q14508** | **WAP four-disulfide Core domain protein 2** | **CCSAGCATFCSLPNDKEGSCPQVNINFPQLGLCR** |
| **Q14508** | **WAP four-disulfide Core domain protein 2** | **TGVCPELQADQNCTQECVSDSECADNLK** |
| **Q14624** | **Inter-alpha-trypsin inhibitor heavy Chain H4** | **NGIDIYSLTVDSR** |
| **Q14624** | **Inter-alpha-trypsin inhibitor heavy Chain H4** | **AEAQAQYSAAVAK** |
| **Q14624** | **Inter-alpha-trypsin inhibitor heavy Chain H4** | **AGFSWIEVTFK** |
| **Q14624** | **Inter-alpha-trypsin inhibitor heavy Chain H4** | **ITFELVYEELLK** |
| **Q14624** | **Inter-alpha-trypsin inhibitor heavy Chain H4** | **NPLVWVHASPEHVVVTR** |
| **Q14624** | **Inter-alpha-trypsin inhibitor heavy Chain H4** | **LALDNGGLAR** |
| **Q14624** | **Inter-alpha-trypsin inhibitor heavy Chain H4** | **LGVYELLLK** |
| **Q14624** | **Inter-alpha-trypsin inhibitor heavy Chain H4** | **WKETLFSVMPGLK** |
| **Q14624** | **Inter-alpha-trypsin inhibitor heavy Chain H4** | **SPEQQETVLDGNLIIR** |
| **Q14624** | **Inter-alpha-trypsin inhibitor heavy Chain H4** | **QGPVNLLSDPEQGVEVTGQYEREK** |
| **Q14624** | **Inter-alpha-trypsin inhibitor heavy Chain H4** | **DQFNLIVFSTEATQWRPSLVPASAENVNK** |
| **Q14624** | **Inter-alpha-trypsin inhibitor heavy Chain H4** | **NMEQFQVSVSVAPNAK** |
| **Q14624** | **Inter-alpha-trypsin inhibitor heavy Chain H4** | **ILDDLSPR** |
| **Q14624** | **Inter-alpha-trypsin inhibitor heavy Chain H4** | **VRPQQLVK** |
| **Q14624** | **Inter-alpha-trypsin inhibitor heavy Chain H4** | **LWAYLTIQQLLEQTVSASDADQQALR** |
| **Q14624** | **Inter-alpha-trypsin inhibitor heavy Chain H4** | **AISGGSIQIENGYFVHYFAPEGLTTMPK** |
| **Q14624** | **Inter-alpha-trypsin inhibitor heavy Chain H4** | **ANTVQEATFQMELPK** |
| **Q14624** | **Inter-alpha-trypsin inhibitor heavy Chain H4** | **EKAEAQAQYSAAVAK** |
| **Q14624** | **Inter-alpha-trypsin inhibitor heavy Chain H4** | **QGPVNLLSDPEQGVEVTGQYER** |
| **Q14624** | **Inter-alpha-trypsin inhibitor heavy Chain H4** | **MNFRPGVLSSR** |
| **Q14624** | **Inter-alpha-trypsin inhibitor heavy Chain H4** | **GSEMVVAGK** |
| **Q14624** | **Inter-alpha-trypsin inhibitor heavy Chain H4** | **FKPTLSQQQK** |
| **Q14624** | **Inter-alpha-trypsin inhibitor heavy Chain H4** | **HRQGPVNLLSDPEQGVEVTGQYER** |
| **Q14624** | **Inter-alpha-trypsin inhibitor heavy Chain H4** | **LQDRGPDVLTATVSGK** |
| **Q15121** | **AstroCytiC phOSphoprotein PEA-15** | **DNLSYIEHIFEISR** |
| **Q15121** | **AstroCytiC phOSphoprotein PEA-15** | **ISEEDELDTK** |
| **Q15436** | **Protein transport protein SeC23A** | **MVVPVAALFTPLK** |
| **Q16555** | **Dihydropyrimidinase-related protein 2** | **FQMPDQGMTSADDFFQGTK** |
| **Q16555** | **Dihydropyrimidinase-related protein 2** | **GLYDGPVCEVSVTPK** |
| **Q16555** | **Dihydropyrimidinase-related protein 2** | **AITIANQTNCPLYITK** |
| **Q16555** | **Dihydropyrimidinase-related protein 2** | **MDENQFVAVTSTNAAK** |
| **Q16555** | **Dihydropyrimidinase-related protein 2** | **VFNLYPR** |
| **Q16555** | **Dihydropyrimidinase-related protein 2** | **IAVGSDADLVIWDPDSVK** |
| **Q16555** | **Dihydropyrimidinase-related protein 2** | **ILDLGITGPEGHVLSRPEEVEAEAVNR** |
| **Q16555** | **Dihydropyrimidinase-related protein 2** | **QIGENLIVPGGVK** |
| **Q16658** | **FasCin** | **VGKDELFALEQSCAQVVLQAANER** |
| **Q16658** | **FasCin** | **LVARPEPATGYTLEFR** |
| **Q16658** | **FasCin** | **YLAADKDGNVTCER** |
| **Q16658** | **FasCin** | **LSCFAQTVSPAEK** |
| **Q16658** | **FasCin** | **YWTLTATGGVQSTASSK** |
| **Q16658** | **FasCin** | **YSVQTADHR** |
| **Q16658** | **FasCin** | **ASAETVDPASLWEY** |
| **Q16658** | **FasCin** | **YLKGDHAGVLK** |
| **Q16658** | **FasCin** | **KVTGTLDANR** |
| **Q16658** | **FasCin** | **LINRPIIVFR** |
| **Q16851** | **UTP--gluCOSe-1-phOSphate uridylyltransferase** | **TLDGGLNVIQLETAVGAAIK** |
| **Q16851** | **UTP--gluCOSe-1-phOSphate uridylyltransferase** | **SFENSLGINVPR** |
| **Q6FHJ7** | **SeCreted frizzled-related protein 4** | **SGCNEVTTVVDVK** |
| **Q6FHJ7** | **SeCreted frizzled-related protein 4** | **MMLLENCLVEK** |
| **Q8NBS9** | **Thioredoxin domain-Containing protein 5** | **IAEVDCTAER** |
| **Q8NBS9** | **Thioredoxin domain-Containing protein 5** | **VDCTAHSDVCSAQGVR** |
| **Q8WXI7** | **MuCin-16** | **DGAATGMDAVCLYHPNPK** |
| **Q8WXI7** | **MuCin-16** | **HGAATGVDAICTLR** |
| **Q8WXI7** | **MuCin-16** | **VLQGLLGPMFK** |
| **Q8WXI7** | **MuCin-16** | **ALFSSNLDPSLVEQVFLDK** |
| **Q8WXI7** | **MuCin-16** | **VLQGLLGPIFK** |
| **Q8WXI7** | **MuCin-16** | **VLQGLLSPIFK** |
| **Q8WXI7** | **MuCin-16** | **VLQSLLGPMFK** |
| **Q8WXI7** | **MuCin-16** | **LYWELSQLTNGIK** |
| **Q8WXI7** | **MuCin-16** | **VLQGLLMPLFK** |
| **Q8WXI7** | **MuCin-16** | **LTNDIEELGPYTLDR** |
| **Q8WXI7** | **MuCin-16** | **NSLYVNGFTHR** |
| **Q8WXI7** | **MuCin-16** | **VLQGLLRPLFK** |
| **Q8WXI7** | **MuCin-16** | **SPGVDREQLYWELSQLTNGIK** |
| **Q8WXI7** | **MuCin-16** | **DGAATGVDAICTHR** |
| **Q8WXI7** | **MuCin-16** | **LTLLRSEK** |
| **Q8WXI7** | **MuCin-16** | **VLQGLLKPLFK** |
| **Q8WXI7** | **MuCin-16** | **VLQGLLRPVFK** |
| **Q8WXI7** | **MuCin-16** | **NTSVGPLYSGCR** |
| **Q8WXI7** | **MuCin-16** | **VLQTLLGPMFK** |
| **Q8WXI7** | **MuCin-16** | **LTLLRPEK** |
| **Q99832** | **T-Complex protein 1 subunit eta** | **SQDAEVGDGTTSVTLLAAEFLK** |
| **Q99832** | **T-Complex protein 1 subunit eta** | **INALTAASEAACLIVSVDETIKNPR** |
| **Q99832** | **T-Complex protein 1 subunit eta** | **EGTDSSQGIPQLVSNISACQVIAEAVR** |
| **Q99832** | **T-Complex protein 1 subunit eta** | **INALTAASEAACLIVSVDETIK** |
| **Q9H6S3** | **Epidermal growth faCtor reCeptor kinase substrate 8-like protein 2** | **LLDIESQEELEDFPLPTVQR** |
| **Q9HC84** | **MuCin-5B** | **IVTENIPCGTTGTTCSK** |
| **Q9HC84** | **MuCin-5B** | **AAGGAVCEQPLGLECR** |
| **Q9HC84** | **MuCin-5B** | **AQAQPGVPLRELGQVVECSLDFGLVCR** |
| **Q9HC84** | **MuCin-5B** | **YAYVVDACQPTCR** |
| **Q9HC84** | **MuCin-5B** | **MCFNYEIR** |
| **Q9HC84** | **MuCin-5B** | **YSAEAQAMQHQCTCCQER** |
| **Q9HC84** | **MuCin-5B** | **LTDPNSAFSR** |
| **Q9HC84** | **MuCin-5B** | **NSFEDPCSLSVENENYAR** |
| **Q9HC84** | **MuCin-5B** | **SVVGDALEFGNSWK** |
| **Q9HC84** | **MuCin-5B** | **ELGQVVECSLDFGLVCR** |
| **Q9HC84** | **MuCin-5B** | **VCGLCGNFDDNAINDFATR** |
| **Q9HC84** | **MuCin-5B** | **AENYPEVSIDQVGQVLTCSLETGLTCKNEDQTGR** |
| **Q9HC84** | **MuCin-5B** | **CPTCPCATFVEYSR** |
| **Q9HC84** | **MuCin-5B** | **EEGLILFDQIPVSSGFSK** |
| **Q9HC84** | **MuCin-5B** | **TGCCYSCEEDSCQVR** |
| **Q9HC84** | **MuCin-5B** | **AQAQPGVPLGELGQVVECSLDFGLVCR** |
| **Q9HC84** | **MuCin-5B** | **SEQLGGDVESYDK** |
| **Q9HC84** | **MuCin-5B** | **AENYPEVSIDQVGQVLTCSLETGLTCK** |
| **Q9HC84** | **MuCin-5B** | **TGLLVEQSGDYIK** |
| **Q9HC84** | **MuCin-5B** | **DIECQAESFPNWTLAQVGQK** |
| **Q9HC84** | **MuCin-5B** | **VDCNTCTCR** |
| **Q9HC84** | **MuCin-5B** | **GYQVCPVLADIECR** |
| **Q9HC84** | **MuCin-5B** | **AVTLSLDGGDTAIR** |
| **Q9HC84** | **MuCin-5B** | **GRLEVPCQSLEAYAELCR** |
| **Q9HC84** | **MuCin-5B** | **LEVPCQSLEAYAELCR** |
| **Q9HC84** | **MuCin-5B** | **CQWTEWFDEDYPK** |
| **Q9HC84** | **MuCin-5B** | **NGVLVSVLGTTTMR** |
| **Q9HC84** | **MuCin-5B** | **GPGGDPPYK** |
| **Q9HC84** | **MuCin-5B** | **GVCSDWRGATGGLCDLTCPPTK** |
| **Q9HC84** | **MuCin-5B** | **CQWTEWFDEDYPKSEQLGGDVESYDK** |
| **Q9HC84** | **MuCin-5B** | **CPPSQPFFNEDQmK** |
| **Q9HC84** | **MuCin-5B** | **TCPLNMQHQECGSPCTDTCSNPQR** |
| **Q9HC84** | **MuCin-5B** | **CPELCPR** |
| **Q9HC84** | **MuCin-5B** | **CVAQCGCYDKDGNYYDVGAR** |
| **Q9HC84** | **MuCin-5B** | **FNMCFNYNVR** |
| **Q9HC84** | **MuCin-5B** | **GRVCGLCGNFDDNAINDFATR** |
| **Q9HC84** | **MuCin-5B** | **TLLGPAFAECHALVDSTAYLAACAQDLCR** |
| **Q9HC84** | **MuCin-5B** | **YMQNCPK** |
| **Q9HC84** | **MuCin-5B** | **IRAAGGHLCQQPK** |
| **Q9HC84** | **MuCin-5B** | **CVAQCGCYDK** |
| **Q9HC84** | **MuCin-5B** | **TPDTCPLFCDFYNPHGGCEWHYQPCGAPCLK** |
| **Q9HC84** | **MuCin-5B** | **AQAQPGVPLR** |
| **Q9HC84** | **MuCin-5B** | **TSVFIR** |
| **Q9HC84** | **MuCin-5B** | **mCYNYR** |
| **Q9HC84** | **MuCin-5B** | **NEDQTGRFNmCFNYNVR** |
| **Q9HC84** | **MuCin-5B** | **YSAEAQAMQHQCTCCQERR** |
| **Q9HC84** | **MuCin-5B** | **VLCCSDDHCR** |
| **Q9HC84** | **MuCin-5B** | **AAQLPDMPLEELGQQVDCDR** |
| **Q9HC84** | **MuCin-5B** | **GLMCANSQQSPPLCHDYELR** |
| **Q9HC84** | **MuCin-5B** | **AAGGHLCQQPK** |
| **Q9HC84** | **MuCin-5B** | **TWLVPDSR** |
| **Q9HC84** | **MuCin-5B** | **SEDCLCAALSSYVHACAAK** |
| **Q9HC84** | **MuCin-5B** | **ALSIHYK** |
| **Q9HC84** | **MuCin-5B** | **VCSTWGDFHYK** |
| **Q9NY33** | **Dipeptidyl peptidase 3** | **LAQDFLDSQNLSAYNTR** |
| **Q9NY33** | **Dipeptidyl peptidase 3** | **EGITTYFSGNCTMEDAK** |
| **Q9NY33** | **Dipeptidyl peptidase 3** | **LEGSDVQLLEYEASAAGLIR** |
| **Q9NY33** | **Dipeptidyl peptidase 3** | **LASVLGSEPSLDSEVTSK** |
| **Q9NY33** | **Dipeptidyl peptidase 3** | **GAFNFDQETVINPETGEQIQSWYR** |
| **Q9NY33** | **Dipeptidyl peptidase 3** | **LVASAEQLLK** |
| **Q9NY33** | **Dipeptidyl peptidase 3** | **VILGSEAAQQHPEEVR** |
| **Q9NY33** | **Dipeptidyl peptidase 3** | **FWKGPSEAPSGQA** |
| Q9NRX4 | 14 kDa phOSphohistidine phOSphatase | QGCDCECLGGGR |
| Q9NRX4 | 14 kDa phOSphohistidine phOSphatase | AKYPDYEVTWANDGY |
| P31946 | 14-3-3 protein beta/alpha | IEAELQDICNDVLELLDK |
| P31946 | 14-3-3 protein beta/alpha | AVTEQGHELSNEER |
| P31946 | 14-3-3 protein beta/alpha | YLSEVASGDNK |
| P31946 | 14-3-3 protein beta/alpha | TAFDEAIAELDTLNEESYK |
| P62258 | 14-3-3 protein epsilon | AAFDDAIAELDTLSEESYK |
| P62258 | 14-3-3 protein epsilon | LGLALNFSVFYYEILNSPDR |
| P62258 | 14-3-3 protein epsilon | LICCDILDVLDK |
| P62258 | 14-3-3 protein epsilon | DNLTLWTSDMQGDGEEQNK |
| P62258 | 14-3-3 protein epsilon | AASDIAMTELPPTHPIR |
| P62258 | 14-3-3 protein epsilon | EAAENSLVAYK |
| P62258 | 14-3-3 protein epsilon | IISSIEQKEENK |
| P62258 | 14-3-3 protein epsilon | QMVETELK |
| P62258 | 14-3-3 protein epsilon | DSTLIMQLLRDNLTLWTSDMQGDGEEQNK |
| P62258 | 14-3-3 protein epsilon | HLIPAANTGESK |
| P62258 | 14-3-3 protein epsilon | YLAEFATGNDRK |
| P62258 | 14-3-3 protein epsilon | MKGDYHR |
| Q04917 | 14-3-3 protein eta | QAFDDAIAELDTLNEDSYKDSTLIMQLLR |
| Q04917 | 14-3-3 protein eta | NCNDFQYESK |
| Q04917 | 14-3-3 protein eta | ELETVCNDVLSLLDK |
| P61981 | 14-3-3 protein gamma | ELEAVCQDVLSLLDNYLIK |
| P61981 | 14-3-3 protein gamma | NVTELNEPLSNEER |
| P61981 | 14-3-3 protein gamma | TAFDDAIAELDTLNEDSYK |
| P61981 | 14-3-3 protein gamma | YLAEVATGEK |
| P61981 | 14-3-3 protein gamma | LGLALNYSVFYYEIQNAPEQACHLAK |
| P61981 | 14-3-3 protein gamma | AYSEAHEISK |
| P61981 | 14-3-3 protein gamma | NCSETQYESK |
| P61981 | 14-3-3 protein gamma | ATVVESSEK |
| P61981 | 14-3-3 protein gamma | RATVVESSEK |
| P27348 | 14-3-3 protein theta | SICTTVLELLDK |
| P27348 | 14-3-3 protein theta | YLAEVACGDDRK |
| P27348 | 14-3-3 protein theta | LGLALNFSVFYYEILNNPELACTLAK |
| P27348 | 14-3-3 protein theta | TAFDEAIAELDTLNEDSYK |
| P27348 | 14-3-3 protein theta | AVTEQGAELSNEERNLLSVAYK |
| P27348 | 14-3-3 protein theta | LAEQAERYDDMATCMK |
| P27348 | 14-3-3 protein theta | AVTEQGAELSNEER |
| P27348 | 14-3-3 protein theta | QTIDNSQGAYQEAFDISKK |
| P27348 | 14-3-3 protein theta | YLIANATNPESK |
| P27348 | 14-3-3 protein theta | YLAEVACGDDR |
| P63104 | 14-3-3 protein zeta/delta | GIVDQSQQAYQEAFEISK |
| P63104 | 14-3-3 protein zeta/delta | GIVDQSQQAYQEAFEISKK |
| P63104 | 14-3-3 protein zeta/delta | TAFDEAIAELDTLSEESYK |
| P63104 | 14-3-3 protein zeta/delta | SVTEQGAELSNEER |
| P63104 | 14-3-3 protein zeta/delta | YLAEVAAGDDK |
| P63104 | 14-3-3 protein zeta/delta | VVSSIEQKTEGAEK |
| P63104 | 14-3-3 protein zeta/delta | YLAEVAAGDDKK |
| P63104 | 14-3-3 protein zeta/delta | DICNDVLSLLEK |
| P63104 | 14-3-3 protein zeta/delta | TAFDEAIAELDTLSEESYKDSTLIMQLLR |
| P63104 | 14-3-3 protein zeta/delta | SVTEQGAELSNEERNLLSVAYK |
| P63104 | 14-3-3 protein zeta/delta | LAEQAERYDDMAACMK |
| P63104 | 14-3-3 protein zeta/delta | IETELRDICNDVLSLLEK |
| P63104 | 14-3-3 protein zeta/delta | FLIPNASQAESK |
| P63104 | 14-3-3 protein zeta/delta | YDDMAACMK |
| P63104 | 14-3-3 protein zeta/delta | DNLTLWTSDTQGDEAEAGEGGEN |
| P63104 | 14-3-3 protein zeta/delta | VVSSIEQK |
| P09543 | 2',3'-CyCliC-nuCleotide 3'-phOSphodiesterase | DKPELQFPFLQDEDTVATLLECK |
| P17980 | 26S protease regulatory subunit 6A | VIAATNRVDILDPALLR |
| P17980 | 26S protease regulatory subunit 6A | LAGPQLVQMFIGDGAK |
| P17980 | 26S protease regulatory subunit 6A | QTYFLPVIGLVDAEK |
| Q99460 | 26S proteasome non-ATPase regulatory subunit 1 | TNLYQDDAVTGEAAGLALGLVMLGSK |
| O00487 | 26S proteasome non-ATPase regulatory subunit 14 | LGGGMPGLGQGPPTDAPAVDTAEQVYISSLALLK |
| Q13200 | 26S proteasome non-ATPase regulatory subunit 2 | APVQPQQSPAAAPGGTDEKPSGK |
| Q13442 | 28 kDa heat- and aCid-stable phOSphoprotein | QYTSPEEIDAQLQAEK |
| Q13442 | 28 kDa heat- and aCid-stable phOSphoprotein | KVTQLDLDGPK |
| P14060 | 3 beta-hydroxysteroid dehydrogenase/Delta 5-->4-isomerase type 1 | IIRLLVK |
| O95861 | 3'(2'),5'-bisphOSphate nuCleotidase 1 | LVTDCVAAMNPDAVLR |
| O95861 | 3'(2'),5'-bisphOSphate nuCleotidase 1 | ASAYVFASPGCK |
| O95861 | 3'(2'),5'-bisphOSphate nuCleotidase 1 | IIQLIEGK |
| O95861 | 3'(2'),5'-bisphOSphate nuCleotidase 1 | LVASAYSIAQK |
| P46952 | 3-hydroxyanthranilate 3,4-dioxygenase | DLGTQLAPIIQEFFSSEQYR |
| Q9BUT1 | 3-hydroxybutyrate dehydrogenase type 2 | VIILTAAAQGIGQAAALAFAR |
| Q9BUT1 | 3-hydroxybutyrate dehydrogenase type 2 | SVAADFIQQGIR |
| Q9BUT1 | 3-hydroxybutyrate dehydrogenase type 2 | SGNIINMSSVASSVK |
| Q9BUT1 | 3-hydroxybutyrate dehydrogenase type 2 | CNCVCPGTVDTPSLQER |
| P25325 | 3-merCaptopyruvate sulfurtransferase | DGIEPGHIPGTVNIPFTDFLSQEGLEK |
| P25325 | 3-merCaptopyruvate sulfurtransferase | AQLDPAFIK |
| P46783 | 40S ribOSomal protein S10 | IAIYELLFK |
| P25398 | 40S ribOSomal protein S12 | RQAHLCVLASNCDEPMYVK |
| P25398 | 40S ribOSomal protein S12 | VVGCSCVVVK |
| P25398 | 40S ribOSomal protein S12 | LGEWVGLCK |
| P62263 | 40S ribOSomal protein S14 | TPGPGAQSALR |
| P62841 | 40S ribOSomal protein S15 | DMIILPEMVGSMVGVYNGK |
| P62244 | 40S ribOSomal protein S15a | MNVLADALK |
| P62244 | 40S ribOSomal protein S15a | FDVQLK |
| P62249 | 40S ribOSomal protein S16 | LLEPVLLLGK |
| P62249 | 40S ribOSomal protein S16 | GPLQSVQVFGR |
| P62249 | 40S ribOSomal protein S16 | TATAVAHCKR |
| P62249 | 40S ribOSomal protein S16 | TATAVAHCK |
| P62249 | 40S ribOSomal protein S16 | FAGVDIR |
| P0CW22 | 40S ribOSomal protein S17-like | LLDFGSLSNLQVTQPTVGMNFK |
| P0CW22 | 40S ribOSomal protein S17-like | DNYVPEVSALDQEIIEVDPDTK |
| P0CW22 | 40S ribOSomal protein S17-like | LGNDFHTNKR |
| P62269 | 40S ribOSomal protein S18 | IPDWFLNR |
| P62269 | 40S ribOSomal protein S18 | VITIMQNPR |
| P62269 | 40S ribOSomal protein S18 | AGELTEDEVER |
| P39019 | 40S ribOSomal protein S19 | DVNQQEFVR |
| P39019 | 40S ribOSomal protein S19 | GGAGVGSMTK |
| P39019 | 40S ribOSomal protein S19 | IAGQVAAANK |
| P39019 | 40S ribOSomal protein S19 | ELAPYDENWFYTR |
| P63220 | 40S ribOSomal protein S21 | TYAICGAIR |
| P62847 | 40S ribOSomal protein S24 | TTGFGMIYDSLDYAK |
| P62847 | 40S ribOSomal protein S24 | TTPDVIFVFGFR |
| P62854 | 40S ribOSomal protein S26 | DISEASVFDAYVLPK |
| P42677 | 40S ribOSomal protein S27 | LVQSPNSYFMDVK |
| P42677 | 40S ribOSomal protein S27 | LTEGCSFR |
| P62857 | 40S ribOSomal protein S28 | TGSQGQCTQVR |
| P23396 | 40S ribOSomal protein S3 | ELAEDGYSGVEVR |
| P23396 | 40S ribOSomal protein S3 | FVADGIFKAELNEFLTR |
| P23396 | 40S ribOSomal protein S3 | GLCAIAQAESLR |
| P23396 | 40S ribOSomal protein S3 | FGFPEGSVELYAEK |
| P23396 | 40S ribOSomal protein S3 | AELNEFLTR |
| P23396 | 40S ribOSomal protein S3 | IMLPWDPTGK |
| P62861 | 40S ribOSomal protein S30 | FVNVVPTFGK |
| P61247 | 40S ribOSomal protein S3a | ATGDETGAKVER |
| P61247 | 40S ribOSomal protein S3a | KTSYAQHQQVR |
| P61247 | 40S ribOSomal protein S3a | LIPDSIGKDIEK |
| P61247 | 40S ribOSomal protein S3a | LFCVGFTK |
| P61247 | 40S ribOSomal protein S3a | TTDGYLLR |
| P62701 | 40S ribOSomal protein S4, X isoform | FDTGNLCMVTGGANLGR |
| P62701 | 40S ribOSomal protein S4, X isoform | LSNIFVIGK |
| P62701 | 40S ribOSomal protein S4, X isoform | ERHPGSFDVVHVK |
| P46782 | 40S ribOSomal protein S5 | TIAECLADELINAAK |
| P46782 | 40S ribOSomal protein S5 | HAFEIIHLLTGENPLQVLVNAIINSGPREDSTR |
| P46782 | 40S ribOSomal protein S5 | VNQAIWLLCTGAR |
| P62753 | 40S ribOSomal protein S6 | LNISFPATGCQK |
| P62081 | 40S ribOSomal protein S7 | AIIIFVPVPQLK |
| P62081 | 40S ribOSomal protein S7 | TLTAVHDAILEDLVFPSEIVGK |
| P62241 | 40S ribOSomal protein S8 | IIDVVYNASNNELVR |
| P62241 | 40S ribOSomal protein S8 | ISSLLEEQFQQGK |
| P46781 | 40S ribOSomal protein S9 | KGQGGAGAGDDEEED |
| P46781 | 40S ribOSomal protein S9 | IGVLDEGK |
| P08865 | 40S ribOSomal protein SA | AIVAIENPADVSVISSR |
| P08865 | 40S ribOSomal protein SA | ILLAAR |
| P08865 | 40S ribOSomal protein SA | ADHQPLTEASYVNLPTIALCNTDSPLR |
| P08865 | 40S ribOSomal protein SA | FLAAGTHLGGTNLDFQMEQYIYK |
| P49189 | 4-trimethylaminobutyraldehyde dehydrogenase | VSFTGSVPTGMK |
| P49189 | 4-trimethylaminobutyraldehyde dehydrogenase | VIATFTCSGEK |
| P49189 | 4-trimethylaminobutyraldehyde dehydrogenase | SPLIIFSDCDMNNAVK |
| P49189 | 4-trimethylaminobutyraldehyde dehydrogenase | VLCGGDIYVPEDPK |
| P49189 | 4-trimethylaminobutyraldehyde dehydrogenase | IGDPLLEDTR |
| P49189 | 4-trimethylaminobutyraldehyde dehydrogenase | EVNLAVQNAK |
| P49189 | 4-trimethylaminobutyraldehyde dehydrogenase | ANDTTFGLAAGVFTR |
| P49189 | 4-trimethylaminobutyraldehyde dehydrogenase | GGARVEPADASGTEK |
| P49189 | 4-trimethylaminobutyraldehyde dehydrogenase | ILLEAAR |
| P49189 | 4-trimethylaminobutyraldehyde dehydrogenase | VLGFVK |
| P21589 | 5'-nuCleotidase | GPLASQISGLYLPYK |
| P21589 | 5'-nuCleotidase | TIVYLDGSSQSCR |
| P21589 | 5'-nuCleotidase | VLPVGDEVVGIVGYTSK |
| P21589 | 5'-nuCleotidase | YDAMALGNHEFDNGVEGLIEPLLK |
| P21589 | 5'-nuCleotidase | FRECNMGNLICDAMINNNLR |
| P21589 | 5'-nuCleotidase | VPVVQAYAFGK |
| P21589 | 5'-nuCleotidase | ETPFLSNPGTNLVFEDEITALQPEVDK |
| P21589 | 5'-nuCleotidase | YPFIVTSDDGR |
| P21589 | 5'-nuCleotidase | FPILSANIK |
| P21589 | 5'-nuCleotidase | KVPVVQAYAFGK |
| P21589 | 5'-nuCleotidase | ETPFLSNPGTNLVFEDEITALQPEVDKLK |
| P21589 | 5'-nuCleotidase | AKGPLASQISGLYLPYK |
| P21589 | 5'-nuCleotidase | GAEVAHFMNALR |
| P21589 | 5'-nuCleotidase | VVKLDVLCTK |
| P10809 | 60 kDa heat shoCk protein, mitoChondrial | ALMLQGVDLLADAVAVTMGPK |
| P10809 | 60 kDa heat shoCk protein, mitoChondrial | TALLDAAGVASLLTTAEVVVTEIPKEEK |
| P10155 | 60 kDa SS-A/Ro ribonuCleoprotein | LGLENAEALIR |
| P10155 | 60 kDa SS-A/Ro ribonuCleoprotein | ALLQEMPLTALLR |
| P10155 | 60 kDa SS-A/Ro ribonuCleoprotein | VLGSILNASTVAAAMCMVVTR |
| P05388 | 60S aCidiC ribOSomal protein P0 | TSFFQALGITTK |
| P05388 | 60S aCidiC ribOSomal protein P0 | GHLENNPALEK |
| P05388 | 60S aCidiC ribOSomal protein P0 | IIQLLDDYPK |
| P05388 | 60S aCidiC ribOSomal protein P0 | AGAIAPCEVTVPAQNTGLGPEK |
| P05386 | 60S aCidiC ribOSomal protein P1 | INALIK |
| P05386 | 60S aCidiC ribOSomal protein P1 | AAGVNVEPFWPGLFAK |
| P05386 | 60S aCidiC ribOSomal protein P1 | ALANVNIGSLICNVGAGGPAPAAGAAPAGGPAPSTAAAPAEEK |
| P05387 | 60S aCidiC ribOSomal protein P2 | YVASYLLAALGGNSSPSAK |
| P05387 | 60S aCidiC ribOSomal protein P2 | NIEDVIAQGIGK |
| P05387 | 60S aCidiC ribOSomal protein P2 | LASVPAGGAVAVSAAPGSAAPAAGSAPAAAEEK |
| P27635 | 60S ribOSomal protein L10 | FNADEFEDMVAEK |
| P62913 | 60S ribOSomal protein L11 | YDGIILPGK |
| P62913 | 60S ribOSomal protein L11 | VLEQLTGQTPVFSK |
| P30050 | 60S ribOSomal protein L12 | CTGGEVGATSALAPK |
| P30050 | 60S ribOSomal protein L12 | IGPLGLSPK |
| P30050 | 60S ribOSomal protein L12 | QAQIEVVPSASALIIK |
| P26373 | 60S ribOSomal protein L13 | STESLQANVQR |
| P26373 | 60S ribOSomal protein L13 | LATQLTGPVMPVR |
| P26373 | 60S ribOSomal protein L13 | LILFPR |
| P26373 | 60S ribOSomal protein L13 | RIAPRPASGPIRPIVR |
| P26373 | 60S ribOSomal protein L13 | KGDSSAEELK |
| P50914 | 60S ribOSomal protein L14 | LVAIVDVIDQNR |
| P50914 | 60S ribOSomal protein L14 | CMQLTDFILK |
| P61313 | 60S ribOSomal protein L15 | VLNSYWVGEDSTYK |
| P61313 | 60S ribOSomal protein L15 | SLQSVAEER |
| P61313 | 60S ribOSomal protein L15 | FFEVILIDPFHK |
| Q07020 | 60S ribOSomal protein L18 | ILTFDQLALDSPK |
| Q07020 | 60S ribOSomal protein L18 | GCGTVLLSGPR |
| P84098 | 60S ribOSomal protein L19 | VWLDPNETNEIANANSR |
| P46778 | 60S ribOSomal protein L21 | VYNVTQHAVGIVVNK |
| P62829 | 60S ribOSomal protein L23 | KVHPAVVIR |
| P62829 | 60S ribOSomal protein L23 | ISLGLPVGAVINCADNTGAK |
| P83731 | 60S ribOSomal protein L24 | AITGASLADIMAK |
| P47914 | 60S ribOSomal protein L29 | AQAAAPASVPAQAPK |
| P39023 | 60S ribOSomal protein L3 | NNASTDYDLSDK |
| P62888 | 60S ribOSomal protein L30 | VCTLAIIDPGDSDIIR |
| P62888 | 60S ribOSomal protein L30 | IQLVMK |
| P62888 | 60S ribOSomal protein L30 | LVILANNCPALR |
| P62899 | 60S ribOSomal protein L31 | LYTLVTYVPVTTFK |
| P62899 | 60S ribOSomal protein L31 | RIHGVGFK |
| P18077 | 60S ribOSomal protein L35a | NNTVTPGGKPNK |
| P36578 | 60S ribOSomal protein L4 | AAAAAAALQAK |
| P36578 | 60S ribOSomal protein L4 | YAICSALAASALPALVMSK |
| P36578 | 60S ribOSomal protein L4 | FCIWTESAFR |
| P46777 | 60S ribOSomal protein L5 | VGLTNYAAAYCTGLLLAR |
| Q02878 | 60S ribOSomal protein L6 | HQEGEIFDTEK |
| P18124 | 60S ribOSomal protein L7 | IALTDNALIAR |
| P62424 | 60S ribOSomal protein L7a | TCTTVAFTQVNSEDK |
| P62424 | 60S ribOSomal protein L7a | NFGIGQDIQPK |
| P62424 | 60S ribOSomal protein L7a | KVVNPLFEK |
| P62917 | 60S ribOSomal protein L8 | AVVGVVAGGGRIDKPILK |
| P62917 | 60S ribOSomal protein L8 | AVVGVVAGGGR |
| P17858 | 6-phOSphofruCtokinase, liver type | GQVQEVGWHDVAGWLGR |
| P17858 | 6-phOSphofruCtokinase, liver type | AAAYNLVQHGITNLCVIGGDGSLTGANIFR |
| P52209 | 6-phOSphogluConate dehydrogenase, deCarboxylating | WTAISALEYGVPVTLIGEAVFAR |
| P52209 | 6-phOSphogluConate dehydrogenase, deCarboxylating | VGTGEPCCDWVGDEGAGHFVK |
| P52209 | 6-phOSphogluConate dehydrogenase, deCarboxylating | DVLGMAQDEMAQAFEDWNK |
| P52209 | 6-phOSphogluConate dehydrogenase, deCarboxylating | TVSKVDDFLANEAK |
| P52209 | 6-phOSphogluConate dehydrogenase, deCarboxylating | YGPSLMPGGNK |
| P52209 | 6-phOSphogluConate dehydrogenase, deCarboxylating | GILFVGSGVSGGEEGAR |
| P52209 | 6-phOSphogluConate dehydrogenase, deCarboxylating | SAVENCQDSWR |
| P52209 | 6-phOSphogluConate dehydrogenase, deCarboxylating | CLSSLKDER |
| P52209 | 6-phOSphogluConate dehydrogenase, deCarboxylating | AGQAVDDFIEK |
| P52209 | 6-phOSphogluConate dehydrogenase, deCarboxylating | LVPLLDTGDIIIDGGNSEYR |
| O95336 | 6-phOSphogluConolaCtonase | FALGLSGGSLVSMLAR |
| O95336 | 6-phOSphogluConolaCtonase | ILEDQEENPLPAALVQPHTGK |
| O95336 | 6-phOSphogluConolaCtonase | LPIPESQVITINPELPVEEAAEDYAK |
| O95336 | 6-phOSphogluConolaCtonase | LLTVPFEK |
| Q96IU4 | Abhydrolase domain-Containing protein 14B | TPALIVYGDQDPMGQTSFEHLK |
| Q9BWD1 | ACetyl-CoA aCetyltransferase, CytOSoliC | IVSWSQVGVEPSIMGIGPIPAIK |
| Q92485 | ACid sphingomyelinase-like phOSphodiesterase 3b | IAGDQSTLQR |
| Q92485 | ACid sphingomyelinase-like phOSphodiesterase 3b | MLYDDAGVPISAMFITPGVTPWK |
| P39687 | ACidiC leuCine-riCh nuClear phOSphoprotein 32 family member A | SLDLFNCEVTNLNDYRENVFK |
| P39687 | ACidiC leuCine-riCh nuClear phOSphoprotein 32 family member A | CPNLTHLNLSGNK |
| P39687 | ACidiC leuCine-riCh nuClear phOSphoprotein 32 family member A | SNEGKLEGLTDEFEELEFLSTINVGLTSIANLPK |
| P39687 | ACidiC leuCine-riCh nuClear phOSphoprotein 32 family member A | RIHLELR |
| P39687 | ACidiC leuCine-riCh nuClear phOSphoprotein 32 family member A | IHLELR |
| P39687 | ACidiC leuCine-riCh nuClear phOSphoprotein 32 family member A | DISTLEPLK |
| P39687 | ACidiC leuCine-riCh nuClear phOSphoprotein 32 family member A | VSGGLEVLAEK |
| Q92688 | ACidiC leuCine-riCh nuClear phOSphoprotein 32 family member B | SLDLFNCEVTNLNDYRESVFK |
| Q9BTT0 | ACidiC leuCine-riCh nuClear phOSphoprotein 32 family member E | ELEFLSMANVELSSLAR |
| Q9BTT0 | ACidiC leuCine-riCh nuClear phOSphoprotein 32 family member E | INLELR |
| Q9BTT0 | ACidiC leuCine-riCh nuClear phOSphoprotein 32 family member E | CPNLTYLNLSGNK |
| P68032 | ACtin, alpha CardiaC musCle 1 | YPIEHGIITNWDDMEK |
| P60709 | ACtin, CytoplasmiC 1 | LCYVALDFEQEMATAASSSSLEK |
| P60709 | ACtin, CytoplasmiC 1 | DLYANTVLSGGTTMYPGIADR |
| P60709 | ACtin, CytoplasmiC 1 | CPEALFQPSFLGMESCGIHETTFNSIMK |
| P60709 | ACtin, CytoplasmiC 1 | QEYDESGPSIVHRK |
| P60709 | ACtin, CytoplasmiC 1 | TTGIVMDSGDGVTHTVPIYEGYALPHAILR |
| P60709 | ACtin, CytoplasmiC 1 | FRCPEALFQPSFLGMESCGIHETTFNSIMK |
| P60709 | ACtin, CytoplasmiC 1 | VAPEEHPVLLTEAPLNPK |
| P60709 | ACtin, CytoplasmiC 1 | GYSFTTTAER |
| P60709 | ACtin, CytoplasmiC 1 | KDLYANTVLSGGTTMYPGIADR |
| P60709 | ACtin, CytoplasmiC 1 | GYSFTTTAEREIVR |
| P60709 | ACtin, CytoplasmiC 1 | EKLCYVALDFEQEMATAASSSSLEK |
| P63267 | ACtin, gamma-enteriC smooth musCle | YPIEHGIITNWDDMEK |
| P61160 | ACtin-related protein 2 | LCYVGYNIEQEQK |
| P61160 | ACtin-related protein 2 | HLWDYTFGPEK |
| P61160 | ACtin-related protein 2 | DLMVGDEASELR |
| P61160 | ACtin-related protein 2 | VLKGDVEK |
| P61160 | ACtin-related protein 2 | ILLTEPPMNPTK |
| P61160 | ACtin-related protein 2 | GYAFNHSADFETVR |
| O15143 | ACtin-related protein 2/3 Complex subunit 1B | STVLSLDWHPNNVLLAAGSCDFK |
| O15143 | ACtin-related protein 2/3 Complex subunit 1B | KASSEGGTAAGAGLDSLHK |
| O15143 | ACtin-related protein 2/3 Complex subunit 1B | SLESALK |
| O15143 | ACtin-related protein 2/3 Complex subunit 1B | ASSEGGTAAGAGLDSLHK |
| O15144 | ACtin-related protein 2/3 Complex subunit 2 | ELQAHGADELLK |
| O15144 | ACtin-related protein 2/3 Complex subunit 2 | YFQFQEEGKEGENR |
| O15144 | ACtin-related protein 2/3 Complex subunit 2 | NCFASVFEK |
| O15145 | ACtin-related protein 2/3 Complex subunit 3 | LIGNMALLPIR |
| O15145 | ACtin-related protein 2/3 Complex subunit 3 | ETKDTDIVDEAIYYFK |
| O15145 | ACtin-related protein 2/3 Complex subunit 3 | VFDPQNDKPSK |
| O15145 | ACtin-related protein 2/3 Complex subunit 3 | DTDIVDEAIYYFK |
| O15145 | ACtin-related protein 2/3 Complex subunit 3 | ANVFFK |
| P59998 | ACtin-related protein 2/3 Complex subunit 4 | ATLQAALCLENFSSQVVER |
| P59998 | ACtin-related protein 2/3 Complex subunit 4 | HNKPEVEVR |
| O15511 | ACtin-related protein 2/3 Complex subunit 5 | QGNMTAALQAALK |
| O15511 | ACtin-related protein 2/3 Complex subunit 5 | ALAAGGVGSIVR |
| O15511 | ACtin-related protein 2/3 Complex subunit 5 | AGSIVLK |
| Q9BPX5 | ACtin-related protein 2/3 Complex subunit 5-like protein | ALAVGGLGSIIR |
| P61158 | ACtin-related protein 3 | LGYAGNTEPQFIIPSCIAIK |
| P61158 | ACtin-related protein 3 | LSEELSGGR |
| P61158 | ACtin-related protein 3 | FMEQVIFK |
| P61158 | ACtin-related protein 3 | NIVLSGGSTMFR |
| P61158 | ACtin-related protein 3 | DITYFIQQLLR |
| P61158 | ACtin-related protein 3 | GVDDLDFFIGDEAIEKPTYATK |
| P61158 | ACtin-related protein 3 | TLTGTVIDSGDGVTHVIPVAEGYVIGSCIK |
| P61158 | ACtin-related protein 3 | EFNKYDTDGSK |
| P61158 | ACtin-related protein 3 | HGIVEDWDLMER |
| Q9C0K3 | ACtin-related protein 3C | DITYFIQQLLR |
| P53999 | ACtivated RNA polymerase II transCriptional CoaCtivator p15 | GISLNPEQWSQLK |
| P53999 | ACtivated RNA polymerase II transCriptional CoaCtivator p15 | EQISDIDDAVR |
| P53999 | ACtivated RNA polymerase II transCriptional CoaCtivator p15 | QVAPEKPVKK |
| P13798 | ACylamino-aCid-releasing enzyme | ALDVSASDDEIAR |
| P13798 | ACylamino-aCid-releasing enzyme | VTSVVVDVVPR |
| P13798 | ACylamino-aCid-releasing enzyme | TPLLLMLGQEDR |
| P13798 | ACylamino-aCid-releasing enzyme | QLGENFSGIYCSLLPLGCWSADSQR |
| P13798 | ACylamino-aCid-releasing enzyme | QYLVFHDGDSVVFAGPAGNSVETR |
| O75608 | ACyl-protein thioesterase 1 | LAGVTALSCWLPLR |
| P46108 | Adapter moleCule Crk | IGDQEFDSLPALLEFYK |
| P07741 | Adenine phOSphoribOSyltransferase | LAPVPFFSLLQYE |
| P07741 | Adenine phOSphoribOSyltransferase | AAIGLLAR |
| P07741 | Adenine phOSphoribOSyltransferase | VVVVDDLLATGGTMNAACELLGR |
| P00568 | Adenylate kinase isoenzyme 1 | GQLVPLETVLDMLR |
| P00568 | Adenylate kinase isoenzyme 1 | EVQQGEEFER |
| P30520 | AdenylOSuCCinate synthetase isozyme 2 | VGIGAFPTEQDNEIGELLQTR |
| P61204 | ADP-ribOSylation faCtor 3 | NISFTVWDVGGQDK |
| P61204 | ADP-ribOSylation faCtor 3 | MLAEDELRDAVLLVFANK |
| P61204 | ADP-ribOSylation faCtor 3 | QDLPNAMNAAEITDK |
| P61204 | ADP-ribOSylation faCtor 3 | LGEIVTTIPTIGFNVETVEYK |
| P61204 | ADP-ribOSylation faCtor 3 | ILMVGLDAAGK |
| P18085 | ADP-ribOSylation faCtor 4 | ILMVGLDAAGK |
| P36405 | ADP-ribOSylation faCtor-like protein 3 | ILLLGLDNAGK |
| Q9UKK9 | ADP-sugar pyrophOSphatase | HANAKPFEVPFLKF |
| Q9UKK9 | ADP-sugar pyrophOSphatase | EQTADGVAVIPVLQR |
| P43652 | Afamin | RNPFVFAPTLLTVAVHFEEVAK |
| P43652 | Afamin | AESPEVCFNEESPK |
| P43652 | Afamin | IAPQLSTEELVSLGEK |
| P43652 | Afamin | LKHELTDEELQSLFTNFANVVDK |
| P43652 | Afamin | TINPAVDHCCK |
| P43652 | Afamin | ICAMEGLPQK |
| P43652 | Afamin | KSDVGFLPPFPTLDPEEK |
| P43652 | Afamin | FTDSENVCQERDADPDTFFAK |
| P43652 | Afamin | ELISLVEDVSSNYDGCCEGDVVQCIRDTSK |
| P43652 | Afamin | FTFEYSR |
| P43652 | Afamin | FLVNLVK |
| P43652 | Afamin | GQCIINSNKDDRPK |
| P43652 | Afamin | SDVGFLPPFPTLDPEEK |
| P43652 | Afamin | FTDSENVCQER |
| P43652 | Afamin | VNCLQTR |
| P43652 | Afamin | VMNHICSK |
| P43652 | Afamin | HFQNLGK |
| P43652 | Afamin | CQAYESNR |
| P43652 | Afamin | TNFAFR |
| P43652 | Afamin | HELTDEELQSLFTNFANVVDK |
| P43652 | Afamin | ELISLVEDVSSNYDGCCEGDVVQCIR |
| P43652 | Afamin | LPNNVLQEK |
| O00468 | Agrin | EAACLQQTQIEEAR |
| Q02952 | A-kinase anChor protein 12 | LVQNIIQTAVDQFVR |
| P49588 | Alanyl-tRNA synthetase, CytoplasmiC | NVGCLQEALQLATSFAQLR |
| P49588 | Alanyl-tRNA synthetase, CytoplasmiC | QFIDSNPNQPLVILEMESGASAK |
| P14550 | AlCohol dehydrogenase [NADP+] | AWRDPDEPVLLEEPVVLALAEK |
| P14550 | AlCohol dehydrogenase [NADP+] | GLVQALGLSNFNSR |
| P14550 | AlCohol dehydrogenase [NADP+] | GLEVTAYSPLGSSDR |
| P14550 | AlCohol dehydrogenase [NADP+] | HIDCAAIYGNEPEIGEALKEDVGPGK |
| P14550 | AlCohol dehydrogenase [NADP+] | ALEALVAK |
| P14550 | AlCohol dehydrogenase [NADP+] | VFDFTFSPEEMK |
| P14550 | AlCohol dehydrogenase [NADP+] | YALSVGYR |
| P14550 | AlCohol dehydrogenase [NADP+] | NADGTICYDSTHYK |
| P11766 | AlCohol dehydrogenase Class-3 | VCLLGCGISTGYGAAVNTAK |
| P11766 | AlCohol dehydrogenase Class-3 | AAVAWEAGKPLSIEEIEVAPPK |
| P11766 | AlCohol dehydrogenase Class-3 | IKVDEFVTHNLSFDEINK |
| P11766 | AlCohol dehydrogenase Class-3 | GWGVSVVVGVAASGEEIATRPFQLVTGR |
| P11766 | AlCohol dehydrogenase Class-3 | LEPGSVCAVFGLGGVGLAVIMGCK |
| P11766 | AlCohol dehydrogenase Class-3 | VDEFVTHNLSFDEINK |
| P11766 | AlCohol dehydrogenase Class-3 | AGDTVIPLYIPQCGECK |
| P11766 | AlCohol dehydrogenase Class-3 | AALEACHK |
| P11766 | AlCohol dehydrogenase Class-3 | AFELMHSGK |
| P11766 | AlCohol dehydrogenase Class-3 | IIGVDINKDK |
| Q8IZ83 | Aldehyde dehydrogenase family 16 member A1 | GAAACDLVQR |
| Q8IZ83 | Aldehyde dehydrogenase family 16 member A1 | NSVPCQDPITGENLASCLQAQAEDVAAAVEAAR |
| Q96C23 | AldOSe 1-epimerase | ISPDGEEGYPGELK |
| Q96C23 | AldOSe 1-epimerase | AVFGELPSGGGTVEK |
| Q96C23 | AldOSe 1-epimerase | FQLQSDLLR |
| P15121 | AldOSe reduCtase | HIDCAHVYQNENEVGVAIQEK |
| P15121 | AldOSe reduCtase | VCALLSCTSHK |
| P15121 | AldOSe reduCtase | SPPGQVTEAVK |
| P15121 | AldOSe reduCtase | TTAQVLIR |
| P15121 | AldOSe reduCtase | NLVVIPK |
| P05187 | Alkaline phOSphatase, plaCental type | DSTLDPSLMEMTEAALR |
| P05187 | Alkaline phOSphatase, plaCental type | GNFQTIGLSAAAR |
| P05187 | Alkaline phOSphatase, plaCental type | NWYSDADVPASAR |
| P05187 | Alkaline phOSphatase, plaCental type | DGARPDVTESESGSPEYR |
| P05187 | Alkaline phOSphatase, plaCental type | GSSIFGLAPGK |
| P05187 | Alkaline phOSphatase, plaCental type | KLQPAQTAAK |
| P05187 | Alkaline phOSphatase, plaCental type | NLVQEWLAK |
| P05186 | Alkaline phOSphatase, tissue-nonspeCifiC isozyme | ANEGTVGVSAATER |
| P05186 | Alkaline phOSphatase, tissue-nonspeCifiC isozyme | SVGIVTTTR |
| P02763 | Alpha-1-aCid glyCoprotein 1 | YVGGQEHFAHLLILR |
| P02763 | Alpha-1-aCid glyCoprotein 1 | EQLGEFYEALDCLR |
| P02763 | Alpha-1-aCid glyCoprotein 1 | NWGLSVYADKPETTK |
| P02763 | Alpha-1-aCid glyCoprotein 1 | YVGGQEHFAHLLILRDTK |
| P02763 | Alpha-1-aCid glyCoprotein 1 | NWGLSVYADKPETTKEQLGEFYEALDCLR |
| P02763 | Alpha-1-aCid glyCoprotein 1 | TYMLAFDVNDEK |
| P02763 | Alpha-1-aCid glyCoprotein 1 | SDVVYTDWKK |
| P02763 | Alpha-1-aCid glyCoprotein 1 | EQLGEFYEALDCLRIPK |
| P02763 | Alpha-1-aCid glyCoprotein 1 | SDVVYTDWK |
| P19652 | Alpha-1-aCid glyCoprotein 2 | EQLGEFYEALDCLCIPR |
| P19652 | Alpha-1-aCid glyCoprotein 2 | TLMFGSYLDDEK |
| P19652 | Alpha-1-aCid glyCoprotein 2 | NWGLSFYADKPETTK |
| P19652 | Alpha-1-aCid glyCoprotein 2 | SDVMYTDWKK |
| P19652 | Alpha-1-aCid glyCoprotein 2 | EHVAHLLFLR |
| P01011 | Alpha-1-antiChymotrypsin | ITLLSALVETR |
| P01011 | Alpha-1-antiChymotrypsin | RLYGSEAFATDFQDSAAAK |
| P01011 | Alpha-1-antiChymotrypsin | DLDSQTMMVLVNYIFFK |
| P01011 | Alpha-1-antiChymotrypsin | AVLDVFEEGTEASAATAVK |
| P01011 | Alpha-1-antiChymotrypsin | DYNLNDILLQLGIEEAFTSK |
| P01011 | Alpha-1-antiChymotrypsin | EQLSLLDRFTEDAK |
| P01011 | Alpha-1-antiChymotrypsin | ADLSGITGAR |
| P01011 | Alpha-1-antiChymotrypsin | NLAVSQVVHK |
| P01011 | Alpha-1-antiChymotrypsin | FNRPFLMIIVPTDTQNIFFMSK |
| P01011 | Alpha-1-antiChymotrypsin | KLINDYVK |
| P01011 | Alpha-1-antiChymotrypsin | DSLEFREIGELYLPK |
| P01011 | Alpha-1-antiChymotrypsin | AKWEMPFDPQDTHQSR |
| P01011 | Alpha-1-antiChymotrypsin | ITDLIK |
| P01011 | Alpha-1-antiChymotrypsin | LYGSEAFATDFQDSAAAK |
| P01011 | Alpha-1-antiChymotrypsin | LYGSEAFATDFQDSAAAKK |
| P01011 | Alpha-1-antiChymotrypsin | GKITDLIK |
| P01011 | Alpha-1-antiChymotrypsin | EQLSLLDR |
| P08697 | Alpha-2-antiplasmin | LGNQEPGGQTALK |
| P08697 | Alpha-2-antiplasmin | LCQDLGPGAFR |
| P08697 | Alpha-2-antiplasmin | LQQVLHAGSGPCLPHLLSR |
| P08697 | Alpha-2-antiplasmin | DFLQSLK |
| P08697 | Alpha-2-antiplasmin | HQMDLVATLSQLGLQELFQAPDLR |
| P08697 | Alpha-2-antiplasmin | WFLLEQPEIQVAHFPFK |
| P12814 | Alpha-aCtinin-1 | VGWEQLLTTIAR |
| P12814 | Alpha-aCtinin-1 | GYEEWLLNEIR |
| P12814 | Alpha-aCtinin-1 | TINEVENQILTR |
| P12814 | Alpha-aCtinin-1 | LLETIDQLYLEYAK |
| P12814 | Alpha-aCtinin-1 | ICDQWDNLGALTQK |
| P12814 | Alpha-aCtinin-1 | RDQALTEEHAR |
| O43707 | Alpha-aCtinin-4 | VGWEQLLTTIAR |
| O43707 | Alpha-aCtinin-4 | ETTDTDTADQVIASFK |
| O43707 | Alpha-aCtinin-4 | CQLEINFNTLQTK |
| O43707 | Alpha-aCtinin-4 | FAIQDISVEETSAK |
| O43707 | Alpha-aCtinin-4 | GISQEQMQEFR |
| O43707 | Alpha-aCtinin-4 | TINEVENQILTR |
| O43707 | Alpha-aCtinin-4 | ALDFIASK |
| O43707 | Alpha-aCtinin-4 | HRDYETATLSDIK |
| O43707 | Alpha-aCtinin-4 | MLDAEDIVNTARPDEK |
| O43707 | Alpha-aCtinin-4 | ICDQWDALGSLTHSR |
| O43707 | Alpha-aCtinin-4 | MAPYQGPDAVPGALDYK |
| O43707 | Alpha-aCtinin-4 | AGTQIENIDEDFRDGLK |
| O43707 | Alpha-aCtinin-4 | IAESNHIK |
| O43707 | Alpha-aCtinin-4 | LSGSNPYTTVTPQIINSK |
| O43707 | Alpha-aCtinin-4 | HRPELIEYDK |
| P04745 | Alpha-amylase 1 | TGSGDIENYNDATQVR |
| P04745 | Alpha-amylase 1 | DFPAVPYSGWDFNDGK |
| P04745 | Alpha-amylase 1 | LSGLLDLALGK |
| P61163 | Alpha-CentraCtin | DQLQTFSEEHPVLLTEAPLNPR |
| P61163 | Alpha-CentraCtin | TLFSNIVLSGGSTLFK |
| P37840 | Alpha-synuClein | TVEGAGSIAAATGFVKK |
| P37840 | Alpha-synuClein | EGVVHGVATVAEK |
| P37840 | Alpha-synuClein | TKEQVTNVGGAVVTGVTAVAQK |
| Q12904 | AminoaCyl tRNA synthase Complex-interaCting multifunCtional protein 1 | GAEADQIIEYLK |
| Q12904 | AminoaCyl tRNA synthase Complex-interaCting multifunCtional protein 1 | TVVSGLVNHVPLEQMQNR |
| Q9H4A4 | Aminopeptidase B | AFFPCFDTPAVK |
| Q9H4A4 | Aminopeptidase B | AEFGPPGPGAGSR |
| Q9H4A4 | Aminopeptidase B | ETFASTASQLHSNVVNYVQQIVAPK |
| Q9H4A4 | Aminopeptidase B | KKPFVYTQGQAVLNR |
| Q9H4A4 | Aminopeptidase B | LQVLLTYR |
| P15144 | Aminopeptidase N | DNEETGFGSGTR |
| P05067 | Amyloid beta A4 protein | CLVGEFVSDALLVPDK |
| P12821 | Angiotensin-Converting enzyme | IAFLPFGYLVDQWR |
| P12821 | Angiotensin-Converting enzyme | DMVGLDALDAQPLLK |
| P12821 | Angiotensin-Converting enzyme | IAFIPFSYLVDQWR |
| P12821 | Angiotensin-Converting enzyme | VLQAGSSRPWQEVLK |
| P12821 | Angiotensin-Converting enzyme | YNFDWWYLR |
| P12821 | Angiotensin-Converting enzyme | LNGYVDAGDSWR |
| P12821 | Angiotensin-Converting enzyme | TLGSANLPLAK |
| P12821 | Angiotensin-Converting enzyme | ENYNQEWWSLR |
| P12821 | Angiotensin-Converting enzyme | TQGDFDPGAK |
| P01019 | Angiotensinogen | TIHLTMPQLVLQGSYDLQDLLAQAELPAILHTELNLQK |
| P01019 | Angiotensinogen | QPFVQGLALYTPVVLPR |
| P01019 | Angiotensinogen | VLSALQAVQGLLVAQGR |
| P01019 | Angiotensinogen | ANAGKPKDPTFIPAPIQAK |
| P01019 | Angiotensinogen | ADSQAQLLLSTVVGVFTAPGLHLK |
| P01019 | Angiotensinogen | IDRFMQAVTGWK |
| P01019 | Angiotensinogen | ADSQAQLLLSTVVGVFTAPGLHLKQPFVQGLALYTPVVLPR |
| P01019 | Angiotensinogen | ALQDQLVLVAAK |
| P01019 | Angiotensinogen | FMQAVTGWK |
| P01019 | Angiotensinogen | SLDFTELDVAAEK |
| P01019 | Angiotensinogen | AAMVGMLANFLGFR |
| P01019 | Angiotensinogen | SLDFTELDVAAEKIDR |
| Q8N6S4 | Ankyrin repeat domain-Containing protein 13C | LSSLIR |
| P16157 | Ankyrin-1 | IIALGPTGAQFLSPVIVEIPHFASHGR |
| P16157 | Ankyrin-1 | FLLENGANQNVATEDGFTPLAVALQQGHENVVAHLINYGTK |
| P12429 | Annexin A3 | GAGTNEDALIEILTTR |
| P12429 | Annexin A3 | SDTSGDYEITLLK |
| P12429 | Annexin A3 | GTVRDYPDFSPSVDAEAIQK |
| P12429 | Annexin A3 | QDAQILYK |
| P12429 | Annexin A3 | DYPDFSPSVDAEAIQK |
| P09525 | Annexin A4 | GLGTDEDAIISVLAYR |
| P09525 | Annexin A4 | GAGTDEGCLIEILASR |
| P09525 | Annexin A4 | SELSGNFEQVIVGMMTPTVLYDVQELRR |
| P09525 | Annexin A4 | SETSGSFEDALLAIVK |
| P09525 | Annexin A4 | SLEDDIRSDTSFMFQR |
| P09525 | Annexin A4 | ISQTYQQQYGR |
| P09525 | Annexin A4 | FLTVLCSR |
| P09525 | Annexin A4 | GLGTDDNTLIR |
| P09525 | Annexin A4 | AASGFNAMEDAQTLR |
| P09525 | Annexin A4 | QDAQDLYEAGEK |
| P09525 | Annexin A4 | VLVSLSAGGRDEGNYLDDALVR |
| P09525 | Annexin A4 | AEIDMLDIR |
| P09525 | Annexin A4 | VLLVLCGGDD |
| P09525 | Annexin A4 | DIEQSIKSETSGSFEDALLAIVK |
| P09525 | Annexin A4 | SDTSFMFQR |
| P08133 | Annexin A6 | QRQEVCQSYK |
| P08133 | Annexin A6 | SLHQAIEGDTSGDFLK |
| P08133 | Annexin A6 | SELDMLDIR |
| P08133 | Annexin A6 | AMEGAGTDEK |
| P08133 | Annexin A6 | EAILDIITSR |
| P08133 | Annexin A6 | DAYERDLEADIIGDTSGHFQK |
| P08133 | Annexin A6 | DLMTDLKSEISGDLAR |
| P20073 | Annexin A7 | GAGTDDSTLVR |
| Q8TD06 | Anterior gradient protein 3 homolog | IMFVDPSLTVR |
| P03973 | Antileukoproteinase | CLDPVDTPNPTR |
| P03973 | Antileukoproteinase | RCCPDTCGIK |
| P03973 | Antileukoproteinase | YKKPECQSDWQCPGK |
| P03973 | Antileukoproteinase | AGVCPPKK |
| O14617 | AP-3 Complex subunit delta-1 | DVPVAEEVSALFAGELNPVAPK |
| Q8NCW5 | Apolipoprotein A-I-binding protein | LFGYEPTIYYPK |
| Q8NCW5 | Apolipoprotein A-I-binding protein | GLTVPIASIDIPSGWDVEK |
| Q8NCW5 | Apolipoprotein A-I-binding protein | KSATQFTGR |
| Q8NCW5 | Apolipoprotein A-I-binding protein | GNAGGIQPDLLISLTAPK |
| P04114 | Apolipoprotein B-100 | NLQNNAEWVYQGAIR |
| P04114 | Apolipoprotein B-100 | SVSDGIAALDLNAVANK |
| P04114 | Apolipoprotein B-100 | LLSGGNTLHLVSTTK |
| P04114 | Apolipoprotein B-100 | VPLLLSEPINIIDALEMR |
| P04114 | Apolipoprotein B-100 | LLLQMDSSATAYGSTVSK |
| P04114 | Apolipoprotein B-100 | IGQDGISTSATTNLK |
| P04114 | Apolipoprotein B-100 | NKADYVETVLDSTCSSTVQFLEYELNVLGTHK |
| P04114 | Apolipoprotein B-100 | TQFNNNEYSQDLDAYNTK |
| P04114 | Apolipoprotein B-100 | NFVASHIANILNSEELDIQDLKK |
| P04114 | Apolipoprotein B-100 | LNTDIAGLASAIDMSTNYNSDSLHFSNVFR |
| P04114 | Apolipoprotein B-100 | YTYNYEAESSSGVPGTADSR |
| P04114 | Apolipoprotein B-100 | VIGNMGQTMEQLTPELK |
| P04114 | Apolipoprotein B-100 | DKDQEVLLQTFLDDASPGDK |
| P04114 | Apolipoprotein B-100 | LPQQANDYLNSFNWER |
| P04114 | Apolipoprotein B-100 | IHSGSFQSQVELSNDQEK |
| P04114 | Apolipoprotein B-100 | QTIIVVLENVQR |
| P04114 | Apolipoprotein B-100 | NTLELSNGVIVK |
| P04114 | Apolipoprotein B-100 | NSLFFSAQPFEITASTNNEGNLK |
| P04114 | Apolipoprotein B-100 | IAELSATAQEIIK |
| P04114 | Apolipoprotein B-100 | EYSGTIASEANTYLNSK |
| P04114 | Apolipoprotein B-100 | AALGKLPQQANDYLNSFNWER |
| P04114 | Apolipoprotein B-100 | INNQLTLDSNTK |
| P04114 | Apolipoprotein B-100 | TLADLTLLDSPIK |
| P04114 | Apolipoprotein B-100 | NSEEFAAAMSR |
| P04114 | Apolipoprotein B-100 | ADSVVDLLSYNVQGSGETTYDHK |
| P04114 | Apolipoprotein B-100 | HSITNPLAVLCEFISQSIK |
| P04114 | Apolipoprotein B-100 | NFVASHIANILNSEELDIQDLK |
| P04114 | Apolipoprotein B-100 | NHLQLEGLFFTNGEHTSK |
| P04114 | Apolipoprotein B-100 | GNVATEISTERDLGQCDR |
| P04114 | Apolipoprotein B-100 | VNWEEEAASGLLTSLK |
| P04114 | Apolipoprotein B-100 | YGMVAQVTQTLK |
| P04114 | Apolipoprotein B-100 | GISTSAASPAVGTVGMDMDEDDDFSK |
| P04114 | Apolipoprotein B-100 | AASGTTGTYQEWK |
| P04114 | Apolipoprotein B-100 | SPAFTDLHLR |
| P04114 | Apolipoprotein B-100 | GNVATEISTER |
| P04114 | Apolipoprotein B-100 | AAIQALR |
| P04114 | Apolipoprotein B-100 | IVQILPWEQNEQVK |
| P04114 | Apolipoprotein B-100 | LGNNPVSK |
| P04114 | Apolipoprotein B-100 | LSLESLTSYFSIESSTK |
| P04114 | Apolipoprotein B-100 | AHLDIAGSLEGHLR |
| P04114 | Apolipoprotein B-100 | IADFELPTIIVPEQTIEIPSIK |
| P04114 | Apolipoprotein B-100 | IDDIWNLEVK |
| P04114 | Apolipoprotein B-100 | TGISPLALIK |
| P04114 | Apolipoprotein B-100 | ITENDIQIALDDAK |
| P04114 | Apolipoprotein B-100 | TSSFALNLPTLPEVKFPEVDVLTK |
| P04114 | Apolipoprotein B-100 | SEILAHWSPAK |
| P04114 | Apolipoprotein B-100 | SPSQADINK |
| P04114 | Apolipoprotein B-100 | LPYTIITTPPLK |
| P04114 | Apolipoprotein B-100 | EFNLQNMGLPDFHIPENLFLK |
| P04114 | Apolipoprotein B-100 | QTVNLQLQPYSLVTTLNSDLK |
| P04114 | Apolipoprotein B-100 | AEPLAFTFSHDYK |
| P04114 | Apolipoprotein B-100 | VRESDEETQIK |
| P04114 | Apolipoprotein B-100 | NQDVHSINLPFFETLQEYFER |
| P04114 | Apolipoprotein B-100 | NMEVSVATTTK |
| P04114 | Apolipoprotein B-100 | LDVTTSIGR |
| P04114 | Apolipoprotein B-100 | TPALHFK |
| P04114 | Apolipoprotein B-100 | VPSYTLILPSLELPVLHVPR |
| P04114 | Apolipoprotein B-100 | SNTVASLHTEK |
| P04114 | Apolipoprotein B-100 | EFQVPTFTIPK |
| P04114 | Apolipoprotein B-100 | GFEPTLEALFGK |
| P04114 | Apolipoprotein B-100 | ILGEELGFASLHDLQLLGK |
| P04114 | Apolipoprotein B-100 | ATFQTPDFIVPLTDLR |
| P04114 | Apolipoprotein B-100 | SEYQADYESLR |
| P04114 | Apolipoprotein B-100 | GMTRPLSTLISSSQSCQYTLDAK |
| P04114 | Apolipoprotein B-100 | ATLYALSHAVNNYHK |
| P04114 | Apolipoprotein B-100 | RVHANPLLIDVVTYLVALIPEPSAQQLR |
| P04114 | Apolipoprotein B-100 | VSALLTPAEQTGTWK |
| P04114 | Apolipoprotein B-100 | VELEVPQLCSFILK |
| P04114 | Apolipoprotein B-100 | HVAEAICK |
| P04114 | Apolipoprotein B-100 | KMGLAFESTK |
| P04114 | Apolipoprotein B-100 | VQGVEFSHR |
| P04114 | Apolipoprotein B-100 | SISAALEHK |
| P04114 | Apolipoprotein B-100 | IEGNLIFDPNNYLPK |
| P04114 | Apolipoprotein B-100 | MTSNFPVDLSDYPK |
| P04114 | Apolipoprotein B-100 | FVTQAEGAK |
| P04114 | Apolipoprotein B-100 | FIIPGLK |
| P04114 | Apolipoprotein B-100 | HRHSITNPLAVLCEFISQSIK |
| P04114 | Apolipoprotein B-100 | DLKVEDIPLAR |
| P04114 | Apolipoprotein B-100 | IEIPLPFGGK |
| P04114 | Apolipoprotein B-100 | FSVPAGIVIPSFQALTAR |
| P04114 | Apolipoprotein B-100 | GAYQNNEIK |
| P04114 | Apolipoprotein B-100 | LSLPDFK |
| P04114 | Apolipoprotein B-100 | QVFLYPEKDEPTYILNIK |
| P04114 | Apolipoprotein B-100 | LHVAGNLK |
| P04114 | Apolipoprotein B-100 | KYTYNYEAESSSGVPGTADSR |
| P04114 | Apolipoprotein B-100 | AVSMPSFSILGSDVR |
| P04114 | Apolipoprotein B-100 | LTISEQNIQR |
| P04114 | Apolipoprotein B-100 | NPNGYSFSIPVK |
| P04114 | Apolipoprotein B-100 | AALTELSLGSAYQAMILGVDSK |
| P04114 | Apolipoprotein B-100 | LAPGELTIIL |
| P04114 | Apolipoprotein B-100 | GSTSHHLVSR |
| P04114 | Apolipoprotein B-100 | CSLLVLENELNAELGLSGASMK |
| P04114 | Apolipoprotein B-100 | ALVEQGFTVPEIK |
| P04114 | Apolipoprotein B-100 | QSMTLSSEVQIPDFDVDLGTILR |
| P04114 | Apolipoprotein B-100 | ALYWVNGQVPDGVSK |
| P04114 | Apolipoprotein B-100 | KLTISEQNIQR |
| P04114 | Apolipoprotein B-100 | VLADKFIIPGLK |
| P04114 | Apolipoprotein B-100 | TLQGIPQMIGEVIR |
| P04114 | Apolipoprotein B-100 | INCKVELEVPQLCSFILK |
| P04114 | Apolipoprotein B-100 | LNDLNSVLVMPTFHVPFTDLQVPSCK |
| P04114 | Apolipoprotein B-100 | SKEVPEAR |
| P04114 | Apolipoprotein B-100 | KSISAALEHK |
| P04114 | Apolipoprotein B-100 | QSFDLSVK |
| P04114 | Apolipoprotein B-100 | KMTSNFPVDLSDYPK |
| P04114 | Apolipoprotein B-100 | NSLKIEIPLPFGGK |
| P04114 | Apolipoprotein B-100 | DFSAEYEEDGKYEGLQEWEGK |
| P04114 | Apolipoprotein B-100 | LTLDIQNK |
| P04114 | Apolipoprotein B-100 | NIILPVYDK |
| P04114 | Apolipoprotein B-100 | GIISALLVPPETEEAK |
| P04114 | Apolipoprotein B-100 | TLQELKK |
| P04114 | Apolipoprotein B-100 | LTIFK |
| P04114 | Apolipoprotein B-100 | LTALTKK |
| P04114 | Apolipoprotein B-100 | TILGTMPAFEVSLQALQK |
| P04114 | Apolipoprotein B-100 | IAIANIIDEIIEK |
| P04114 | Apolipoprotein B-100 | YEDGTLSLTSTSDLQSGIIK |
| P04114 | Apolipoprotein B-100 | NIQEYLSILTDPDGK |
| P04114 | Apolipoprotein B-100 | ENFAGEATLQR |
| P04114 | Apolipoprotein B-100 | LELELRPTGEIEQYSVSATYELQR |
| P04114 | Apolipoprotein B-100 | VPQTDMTFR |
| P04114 | Apolipoprotein B-100 | IEDGTLASK |
| P02655 | Apolipoprotein C-II | STAAMSTYTGIFTDQVLSVLK |
| P02655 | Apolipoprotein C-II | STAAMSTYTGIFTDQVLSVLKGEE |
| P02655 | Apolipoprotein C-II | TAAQNLYEK |
| P02655 | Apolipoprotein C-II | ESLSSYWESAK |
| P02656 | Apolipoprotein C-III | DALSSVQESQVAQQAR |
| P02656 | Apolipoprotein C-III | GWVTDGFSSLK |
| Q13790 | Apolipoprotein F | EQAVHNVVQLLPGVGTFYNLGTALYYATQNCLGK |
| O14791 | Apolipoprotein L1 | VTEPISAESGEQVER |
| O14791 | Apolipoprotein L1 | VAQELEEK |
| O95445 | Apolipoprotein M | NQEACELSNN |
| P08519 | Apolipoprotein(a) | TPEYYPNAGLIMNYCR |
| P08519 | Apolipoprotein(a) | GTYSTTVTGR |
| Q9ULZ3 | ApoptOSis-assoCiated speCk-like protein Containing a CARD | VLTDEQYQAVR |
| P53367 | Arfaptin-1 | KYENILK |
| P53365 | Arfaptin-2 | LRGDVAIK |
| P04424 | ArgininOSuCCinate lyase | INVLPLGSGAIAGNPLGVDRELLR |
| Q8N5I2 | Arrestin domain-Containing protein 1 | VVYSPGEPLAGTVR |
| O43776 | Asparaginyl-tRNA synthetase, CytoplasmiC | LMTDTINEPILLCR |
| O43776 | Asparaginyl-tRNA synthetase, CytoplasmiC | IGALEGYR |
| P17174 | Aspartate aminotransferase, CytoplasmiC | IVASTLSNPELFEEWTGNVK |
| P17174 | Aspartate aminotransferase, CytoplasmiC | TPGTWNHITDQIGMFSFTGLNPK |
| P17174 | Aspartate aminotransferase, CytoplasmiC | LALGDDSPALK |
| P17174 | Aspartate aminotransferase, CytoplasmiC | IANDNSLNHEYLPILGLAEFR |
| P17174 | Aspartate aminotransferase, CytoplasmiC | APPSVFAEVPQAQPVLVFK |
| P17174 | Aspartate aminotransferase, CytoplasmiC | VGNLTVVGKEPESILQVLSQMEK |
| Q9ULA0 | Aspartyl aminopeptidase | YASNAVSEALIR |
| Q9ULA0 | Aspartyl aminopeptidase | NINENFGPNTEMHLVPILATAIQEELEK |
| Q9ULA0 | Aspartyl aminopeptidase | GFFELFPSLSHNLLVD |
| P14868 | Aspartyl-tRNA synthetase, CytoplasmiC | VFSIGPVFR |
| P25705 | ATP synthase subunit alpha, mitoChondrial | EIVTNFLAGFEA |
| P25705 | ATP synthase subunit alpha, mitoChondrial | EVAAFAQFGSDLDAATQQLLSR |
| P53396 | ATP-Citrate synthase | DLVSSLTSGLLTIGDRFGGALDAAAK |
| P53396 | ATP-Citrate synthase | IGNTGGMLDNILASK |
| P53396 | ATP-Citrate synthase | AFDSGIIPMEFVNK |
| P53396 | ATP-Citrate synthase | DLVSSLTSGLLTIGDR |
| P53396 | ATP-Citrate synthase | FICTTSAIQNR |
| P53396 | ATP-Citrate synthase | LGLVGVNLTLDGVK |
| P53396 | ATP-Citrate synthase | LGQEATVGK |
| P53396 | ATP-Citrate synthase | SAYDSTMETMNYAQIR |
| P53396 | ATP-Citrate synthase | VDATADYICK |
| P53396 | ATP-Citrate synthase | LTLLNPK |
| Q08211 | ATP-dependent RNA heliCase A | GMTLVTPLQLLLFASK |
| O00148 | ATP-dependent RNA heliCase DDX39A | ILVATNLFGR |
| O75882 | AttraCtin | TACGDCTSGSSECMWCSNMK |
| O75882 | AttraCtin | YDVDTQMWTILK |
| O75882 | AttraCtin | SCALDQNCQWEPR |
| O75882 | AttraCtin | CTWLIEGQPNR |
| O75882 | AttraCtin | CNPGTGQCVCPAGWVGEQCQHCGGR |
| O75882 | AttraCtin | LADDLYRYDVDTQMWTILK |
| O75882 | AttraCtin | LTLTPWVGLR |
| O75882 | AttraCtin | IMQSSQSMSK |
| P20160 | AzuroCidin | HFCGGALIHAR |
| P02730 | Band 3 anion transport protein | GTVLLDLQETSLAGVANQLLDR |
| P02730 | Band 3 anion transport protein | LQEAAELEAVELPVPIR |
| P02730 | Band 3 anion transport protein | NVELQCLDADDAK |
| P02730 | Band 3 anion transport protein | SVTHANALTVMGK |
| P02730 | Band 3 anion transport protein | ADFLEQPVLGFVR |
| P02730 | Band 3 anion transport protein | SGDPSQPLLPQHSSLETQLFCEQGDGGTEGHSPSGILEK |
| P02730 | Band 3 anion transport protein | IPPDSEATLVLVGR |
| P02730 | Band 3 anion transport protein | HSHAGELEALGGVKPAVLTR |
| P02730 | Band 3 anion transport protein | AAATLMSER |
| P02730 | Band 3 anion transport protein | VLLPLIFR |
| P98160 | Basement membrane-speCifiC heparan sulfate proteoglyCan Core protein | LPAVEPTDQAQYLCR |
| P98160 | Basement membrane-speCifiC heparan sulfate proteoglyCan Core protein | TPSGLYLGTCER |
| P98160 | Basement membrane-speCifiC heparan sulfate proteoglyCan Core protein | TCESLGAGGYR |
| P98160 | Basement membrane-speCifiC heparan sulfate proteoglyCan Core protein | LCNECADGSFHLSTR |
| P98160 | Basement membrane-speCifiC heparan sulfate proteoglyCan Core protein | ASYAQQPAESR |
| P98160 | Basement membrane-speCifiC heparan sulfate proteoglyCan Core protein | HCTSSSWSR |
| Q7L1Q6 | BasiC leuCine zipper and W2 domain-Containing protein 1 | FDPTQFQDCIIQGLTETGTDLEAVAK |
| P15291 | Beta-1,4-galaCtOSyltransferase 1 | QQLDYGIYVINQAGDTIFNR |
| P15291 | Beta-1,4-galaCtOSyltransferase 1 | YWLYYLHPVLQR |
| P15291 | Beta-1,4-galaCtOSyltransferase 1 | FGFSLPYVQYFGGVSALSK |
| P15291 | Beta-1,4-galaCtOSyltransferase 1 | LLNVGFQEALK |
| P15291 | Beta-1,4-galaCtOSyltransferase 1 | HISVAMDK |
| P15291 | Beta-1,4-galaCtOSyltransferase 1 | VAIIIPFR |
| P61769 | Beta-2-miCroglobulin | SNFLNCYVSGFHPSDIEVDLLK |
| P61769 | Beta-2-miCroglobulin | IQVYSR |
| P61769 | Beta-2-miCroglobulin | IEKVEHSDLSFSK |
| P61769 | Beta-2-miCroglobulin | VEHSDLSFSK |
| P08236 | Beta-gluCuronidase | RPLWESGPTVDMPVPSSFNDISQDWR |
| P07814 | BifunCtional aminoaCyl-tRNA synthetase | FAGGDYTTTIEAFISASGR |
| P07814 | BifunCtional aminoaCyl-tRNA synthetase | KLGVENCYFPMFVSQSALEK |
| P07814 | BifunCtional aminoaCyl-tRNA synthetase | IVQIPFCGEIDCEDWIK |
| P07814 | BifunCtional aminoaCyl-tRNA synthetase | LTVAENEAETK |
| Q3LXA3 | BifunCtional ATP-dependent dihydroxyaCetone kinase/FAD-AMP lyase (CyClizing) | SPGADLLQVLTK |
| Q3LXA3 | BifunCtional ATP-dependent dihydroxyaCetone kinase/FAD-AMP lyase (CyClizing) | TSLPAWSAAMDAGLEAMQK |
| Q3LXA3 | BifunCtional ATP-dependent dihydroxyaCetone kinase/FAD-AMP lyase (CyClizing) | LIDAETTAAAWPNVAAVSITGR |
| P31939 | BifunCtional purine biOSynthesis protein PURH | LPITVLNGAPGFINLCDALNAWQLVK |
| P31939 | BifunCtional purine biOSynthesis protein PURH | MSSFGDFVALSDVCDVPTAK |
| P31939 | BifunCtional purine biOSynthesis protein PURH | EVSDGIIAPGYEEEALTILSK |
| P31939 | BifunCtional purine biOSynthesis protein PURH | TVASPGVTVEEAVEQIDIGGVTLLR |
| P31939 | BifunCtional purine biOSynthesis protein PURH | DVSELTGFPEMLGGR |
| P31939 | BifunCtional purine biOSynthesis protein PURH | VVACNLYPFVK |
| P31939 | BifunCtional purine biOSynthesis protein PURH | SLFSNVVTK |
| P53004 | Biliverdin reduCtase A | SGSLENVPNVGVNK |
| P43251 | Biotinidase | TSIYPFLDFMPSPQVVR |
| P43251 | Biotinidase | DAQEVHCDEATK |
| P07738 | BisphOSphoglyCerate mutase | AVGPHQFLGDQEAIQAAIK |
| P07738 | BisphOSphoglyCerate mutase | LNSEGMEEAR |
| P07738 | BisphOSphoglyCerate mutase | VCDVPLDQLPR |
| P07738 | BisphOSphoglyCerate mutase | TILISAHGNSSR |
| P07738 | BisphOSphoglyCerate mutase | FCSWVDQK |
| P80723 | Brain aCid soluble protein 1 | KAEGAATEEEGTPK |
| P80723 | Brain aCid soluble protein 1 | AEGAATEEEGTPKESEPQAAAEPAEAK |
| P80723 | Brain aCid soluble protein 1 | AEGAATEEEGTPK |
| P80723 | Brain aCid soluble protein 1 | AQGPAASAEEPKPVEAPAANSDQTVTVKE |
| P80723 | Brain aCid soluble protein 1 | ETPAATEAPSSTPK |
| P80723 | Brain aCid soluble protein 1 | GYNVNDEK |
| P80723 | Brain aCid soluble protein 1 | AEPPKAPEQEQAAPGPAAGGEAPK |
| P80723 | Brain aCid soluble protein 1 | SDGAPASDSKPGSSEAAPSSK |
| P80723 | Brain aCid soluble protein 1 | KTEAPAAPAAQETK |
| P80723 | Brain aCid soluble protein 1 | DAAAAKEEAPK |
| P80723 | Brain aCid soluble protein 1 | ESEPQAAAEPAEAK |
| P54687 | BranChed-Chain-amino-aCid aminotransferase, CytOSoliC | YLTMDDLTTALEGNR |
| P54687 | BranChed-Chain-amino-aCid aminotransferase, CytOSoliC | ATLPVFDKEELLECIQQLVK |
| P54687 | BranChed-Chain-amino-aCid aminotransferase, CytOSoliC | DLIVTPATILK |
| P54687 | BranChed-Chain-amino-aCid aminotransferase, CytOSoliC | LDQEWVPYSTSASLYIRPTFIGTEPSLGVK |
| P54687 | BranChed-Chain-amino-aCid aminotransferase, CytOSoliC | AKDLIVTPATILK |
| Q96CX2 | BTB/POZ domain-Containing protein KCTD12 | MFTQQQPQELAR |
| Q96CX2 | BTB/POZ domain-Containing protein KCTD12 | DLQLVLPDYFPER |
| P04003 | C4b-binding protein alpha Chain | WTPYQGCEALCCPEPK |
| P04003 | C4b-binding protein alpha Chain | SHSTQTLTCNSDGEWVYNTFCIYK |
| P04003 | C4b-binding protein alpha Chain | GSSVIHCDADSK |
| P04003 | C4b-binding protein alpha Chain | FSAICQGDGTWSPR |
| P04003 | C4b-binding protein alpha Chain | SRPANHCVYFYGDEISFSCHETSR |
| P04003 | C4b-binding protein alpha Chain | GVGWSHPLPQCEIVK |
| P04003 | C4b-binding protein alpha Chain | LSCSYSHWSAPAPQCK |
| P04003 | C4b-binding protein alpha Chain | CHPGYKPTTDEPTTVICQK |
| P04003 | C4b-binding protein alpha Chain | MALEVYK |
| P04003 | C4b-binding protein alpha Chain | DTIVFK |
| P04003 | C4b-binding protein alpha Chain | LMQCLPNPEDVK |
| P04003 | C4b-binding protein alpha Chain | LNNGEITQHRK |
| P04003 | C4b-binding protein alpha Chain | LSLEIEQLELQR |
| P04003 | C4b-binding protein alpha Chain | LNNGEITQHR |
| P19022 | Cadherin-2 | YSVTGPGADQPPTGIFIINPISGQLSVTKPLDREQIAR |
| P05937 | Calbindin | LLPVQENFLLK |
| Q99828 | CalCium and integrin-binding protein 1 | AQVPFEQILSLPELK |
| Q9Y2V2 | CalCium-regulated heat stable protein 1 | ASQGPVYK |
| Q9HB71 | CalCyClin-binding protein | KAELLDNEKPAAVVAPITTGYTVK |
| Q9HB71 | CalCyClin-binding protein | SFDLLVK |
| Q05682 | Caldesmon | STHQAAIVSK |
| P62158 | Calmodulin | DGNGYISAAELR |
| P62158 | Calmodulin | EAFSLFDKDGDGTITTK |
| P62158 | Calmodulin | EAFSLFDK |
| P62158 | Calmodulin | VFDKDGNGYISAAELR |
| P62158 | Calmodulin | EADIDGDGQVNYEEFVQMMTAK |
| P62158 | Calmodulin | DTDSEEEIREAFR |
| P27824 | Calnexin | TGIYEEK |
| P04632 | Calpain small subunit 1 | LFAQLAGDDMEVSATELMNILNK |
| P04632 | Calpain small subunit 1 | YSDESGNMDFDNFISCLVR |
| P04632 | Calpain small subunit 1 | TDGFGIDTCR |
| P04632 | Calpain small subunit 1 | ILGGVISAISEAAAQYNPEPPPPR |
| P04632 | Calpain small subunit 1 | THYSNIEANESEEVR |
| P07384 | Calpain-1 CatalytiC subunit | NYPATFWVNPQFK |
| P07384 | Calpain-1 CatalytiC subunit | YLGQDYEQLR |
| P07384 | Calpain-1 CatalytiC subunit | LEICNLTPDALK |
| P20810 | Calpastatin | DTMSDQALEALSASLGTR |
| Q15417 | Calponin-3 | DGIILCELINK |
| P27797 | CalretiCulin | SGTIFDNFLITNDEAYAEEFGNETWGVTK |
| P27797 | CalretiCulin | EQFLDGDGWTSR |
| P27797 | CalretiCulin | HEQNIDCGGGYVK |
| P27797 | CalretiCulin | GQTLVVQFTVK |
| P27797 | CalretiCulin | FYALSASFEPFSNK |
| P27797 | CalretiCulin | GLQTSQDAR |
| P56211 | CAMP-regulated phOSphoprotein 19 | YFDSGDYNMAK |
| Q7L1S5 | Carbohydrate sulfotransferase 9 | STRLLTK |
| P00915 | CarboniC anhydrase 1 | ADGLAVIGVLMK |
| P00915 | CarboniC anhydrase 1 | VLDALQAIK |
| P00915 | CarboniC anhydrase 1 | YSSLAEAASK |
| P00915 | CarboniC anhydrase 1 | ESISVSSEQLAQFR |
| P00915 | CarboniC anhydrase 1 | YSSLAEAASKADGLAVIGVLMK |
| P00915 | CarboniC anhydrase 1 | GGPFSDSYR |
| P00915 | CarboniC anhydrase 1 | HDTSLKPISVSYNPATAK |
| P00915 | CarboniC anhydrase 1 | TSETKHDTSLKPISVSYNPATAK |
| P00915 | CarboniC anhydrase 1 | NGPEQWSK |
| P00915 | CarboniC anhydrase 1 | LYPIANGNNQSPVDIK |
| P00915 | CarboniC anhydrase 1 | SLLSNVEGDNAVPMQHNNRPTQPLK |
| P00915 | CarboniC anhydrase 1 | YSAELHVAHWNSAK |
| P00915 | CarboniC anhydrase 1 | EIINVGHSFHVNFEDNDNR |
| P35219 | CarboniC anhydrase-related protein | AVTEILQDIQYK |
| P16152 | Carbonyl reduCtase [NADPH] 1 | FRSETITEEELVGLMNK |
| P16152 | Carbonyl reduCtase [NADPH] 1 | FHQLDIDDLQSIR |
| P16152 | Carbonyl reduCtase [NADPH] 1 | LFSGDVVLTAR |
| P16152 | Carbonyl reduCtase [NADPH] 1 | VVNVSSIMSVR |
| P16152 | Carbonyl reduCtase [NADPH] 1 | ILLNACCPGWVR |
| P16152 | Carbonyl reduCtase [NADPH] 1 | GIGLAIVR |
| P16152 | Carbonyl reduCtase [NADPH] 1 | SCSPELQQK |
| P16152 | Carbonyl reduCtase [NADPH] 1 | DVCTELLPLIKPQGR |
| P16152 | Carbonyl reduCtase [NADPH] 1 | GQAAVQQLQAEGLSPR |
| O75828 | Carbonyl reduCtase [NADPH] 3 | VALVTGANR |
| Q96IY4 | Carboxypeptidase B2 | DTGTYGFLLPER |
| P14384 | Carboxypeptidase M | IGIPEFK |
| P14384 | Carboxypeptidase M | YVANMHGDETVGR |
| P14384 | Carboxypeptidase M | ENYNQYDLNR |
| P14384 | Carboxypeptidase M | ASLIEYIK |
| P15169 | Carboxypeptidase N CatalytiC Chain | VQNECPGITR |
| P22792 | Carboxypeptidase N subunit 2 | TLNLAQNLLAQLPEELFHPLTSLQTLK |
| P22792 | Carboxypeptidase N subunit 2 | AGGSWDLAVQER |
| P22792 | Carboxypeptidase N subunit 2 | VVFLNTQLCQFRPDAFGGLPR |
| P04040 | Catalase | FNTANDDNVTQVR |
| P04040 | Catalase | AFYVNVLNEEQR |
| P04040 | Catalase | LVNANGEAVYCK |
| P04040 | Catalase | ADVLTTGAGNPVGDK |
| P04040 | Catalase | GAGAFGYFEVTHDITK |
| P04040 | Catalase | GPLLVQDVVFTDEMAHFDR |
| P04040 | Catalase | NFTEVHPDYGSHIQALLDK |
| P04040 | Catalase | LCENIAGHLK |
| P04040 | Catalase | RFNTANDDNVTQVR |
| P04040 | Catalase | LSQEDPDYGIR |
| P04040 | Catalase | DAQIFIQK |
| P04040 | Catalase | AAQKADVLTTGAGNPVGDK |
| P04040 | Catalase | NLSVEDAAR |
| P04040 | Catalase | FSTVAGESGSADTVRDPR |
| P04040 | Catalase | TPIAVR |
| Q9UBR2 | Cathepsin Z | NVDGVNYASITR |
| Q96F85 | CB1 Cannabinoid reCeptor-interaCting protein 1 | IKPSTLQVENISIGGVLVPLELK |
| Q6YHK3 | CD109 antigen | TLTLPSLPLNSADEIYELR |
| Q6YHK3 | CD109 antigen | TNIQVTVTGPSSPSPVK |
| Q6YHK3 | CD109 antigen | ISVTQPDSIVGIVAVDK |
| Q6YHK3 | CD109 antigen | EALNMLTWR |
| Q6YHK3 | CD109 antigen | SNLIQQWLSQQSDLGVISK |
| Q6YHK3 | CD109 antigen | NSLGGFASTQDTTVALK |
| Q6YHK3 | CD109 antigen | IEFPILEDSSELQLK |
| Q6YHK3 | CD109 antigen | ISVFIQTDK |
| Q6YHK3 | CD109 antigen | SSMAVHSLFK |
| Q6YHK3 | CD109 antigen | IPVQLVFK |
| Q6YHK3 | CD109 antigen | VGSPFELVVSGNK |
| Q13740 | CD166 antigen | EMDPVTQLYTMTSTLEYK |
| Q13740 | CD166 antigen | SSPSFSSLHYQDAGNYVCETALQEVEGLK |
| O43866 | CD5 antigen-like | ELGCGAASGTPSGILYEPPAEK |
| O43866 | CD5 antigen-like | CSGEEQSLEQCQHR |
| O43866 | CD5 antigen-like | GVWGSVCDDNWGEKEDQVVCK |
| O43866 | CD5 antigen-like | NTCNHDEDTWVECEDPFDLR |
| O43866 | CD5 antigen-like | LVGGDNLCSGR |
| O43866 | CD5 antigen-like | CEGRVEVEQK |
| O43866 | CD5 antigen-like | AVLTQKR |
| O43866 | CD5 antigen-like | KPIWLSQMSCSGR |
| O43866 | CD5 antigen-like | LADGPGHCK |
| O43866 | CD5 antigen-like | EATLQDCPSGPWGK |
| P13987 | CD59 glyCoprotein | AGLQVYNK |
| P13987 | CD59 glyCoprotein | FEHCNFNDVTTR |
| P13987 | CD59 glyCoprotein | LRENELTYYCCK |
| P60953 | Cell division Control protein 42 homolog | YVECSALTQK |
| P60953 | Cell division Control protein 42 homolog | TPFLLVGTQIDLRDDPSTIEK |
| P60953 | Cell division Control protein 42 homolog | NVFDEAILAALEPPEPK |
| P60953 | Cell division Control protein 42 homolog | QKPITPETAEK |
| P62633 | Cellular nuCleiC aCid-binding protein | CGESGHLAR |
| P29373 | Cellular retinoiC aCid-binding protein 2 | VGEEFEEQTVDGRPCK |
| P29373 | Cellular retinoiC aCid-binding protein 2 | IAVAAASKPAVEIKQEGDTFYIK |
| P29373 | Cellular retinoiC aCid-binding protein 2 | ELTNDGELILTMTADDVVCTR |
| P29373 | Cellular retinoiC aCid-binding protein 2 | VLGVNVMLR |
| Q9NZZ3 | Charged multivesiCular body protein 5 | ISRLDAELVK |
| Q13231 | ChitotriOSidase-1 | FTTLVQDLANAFQQEAQTSGK |
| Q13231 | ChitotriOSidase-1 | ERFTTLVQDLANAFQQEAQTSGK |
| Q13231 | ChitotriOSidase-1 | SFTLASSSDTR |
| Q13231 | ChitotriOSidase-1 | DNQWVGFDDVESFK |
| Q9Y696 | Chloride intraCellular Channel protein 4 | AGSDGESIGNCPFSQR |
| Q9Y696 | Chloride intraCellular Channel protein 4 | NSRPEANEALER |
| Q53GD3 | Choline transporter-like protein 4 | NEFSQTVGEVFYTK |
| Q13185 | Chromobox protein homolog 3 | GFTDADNTWEPEENLDCPELIEAFLNSQK |
| Q13185 | Chromobox protein homolog 3 | IIGATDSSGELMFLMK |
| Q13185 | Chromobox protein homolog 3 | LTWHSCPEDEAQ |
| Q00610 | Clathrin heavy Chain 1 | IYIDSNNNPER |
| Q00610 | Clathrin heavy Chain 1 | LLYNNVSNFGR |
| Q00610 | Clathrin heavy Chain 1 | LASTLVHLGEYQAAVDGAR |
| Q00610 | Clathrin heavy Chain 1 | AQILPIR |
| Q00610 | Clathrin heavy Chain 1 | NLQNLLILTAIK |
| Q00610 | Clathrin heavy Chain 1 | AFMTADLPNELIELLEK |
| Q16630 | Cleavage and polyadenylation speCifiCity faCtor subunit 6 | AVSDASAGDYGSAIETLVTAISLIK |
| Q14019 | CoaCtOSin-like protein | FALITWIGENVSGLQR |
| Q14019 | CoaCtOSin-like protein | KAGGANYDAQTE |
| Q14019 | CoaCtOSin-like protein | YDGSTIVPGEQGAEYQHFIQQCTDDVR |
| Q14019 | CoaCtOSin-like protein | EVVQNFAK |
| P00748 | Coagulation faCtor XII | GRPGPQPWCATTPNFDQDQR |
| P00488 | Coagulation faCtor XIII A Chain | STVLTIPEIIIK |
| P00488 | Coagulation faCtor XIII A Chain | GTYIPVPIVSELQSGK |
| P00488 | Coagulation faCtor XIII A Chain | LALETALMYGAK |
| P00488 | Coagulation faCtor XIII A Chain | GVNLQEFLNVTSVHLFK |
| P00488 | Coagulation faCtor XIII A Chain | LSIQSSPK |
| P00488 | Coagulation faCtor XIII A Chain | LIASMSSDSLR |
| P00488 | Coagulation faCtor XIII A Chain | KDGTHVVENVDATHIGK |
| P53621 | Coatomer subunit alpha | LLQPPAPIMPLDTNWPLLTVSK |
| O14579 | Coatomer subunit epsilon | RMQDLDEDATLTQLATAWVSLATGGEK |
| P23528 | Cofilin-1 | EILVGDVGQTVDDPYATFVK |
| P23528 | Cofilin-1 | LGGSAVISLEGKPL |
| P23528 | Cofilin-1 | HELQANCYEEVK |
| P23528 | Cofilin-1 | NIILEEGKEILVGDVGQTVDDPYATFVK |
| P23528 | Cofilin-1 | YALYDATYETK |
| P23528 | Cofilin-1 | AVLFCLSEDKK |
| P23528 | Cofilin-1 | KSSTPEEVK |
| P23528 | Cofilin-1 | AVLFCLSEDK |
| P23528 | Cofilin-1 | mLPDKDCR |
| Q96JN2 | Coiled-Coil domain-Containing protein 136 | mQLQLQTELR |
| Q8IYT3 | Coiled-Coil domain-Containing protein C6orf97 | SKmLSK |
| Q14011 | Cold-induCible RNA-binding protein | GFGFVTFENIDDAK |
| Q14011 | Cold-induCible RNA-binding protein | YGQISEVVVVK |
| Q14011 | Cold-induCible RNA-binding protein | LFVGGLSFDTNEQSLEQVFSK |
| Q99715 | Collagen alpha-1(XII) Chain | VGVVQYSSDTR |
| Q99715 | Collagen alpha-1(XII) Chain | MIATDPDDTHAYNVADFESLSR |
| Q99715 | Collagen alpha-1(XII) Chain | SLYDDVDTGEK |
| Q05707 | Collagen alpha-1(XIV) Chain | TNQLNLQNTATK |
| P02746 | Complement C1q subComponent subunit B | LEQGENVFLQATDK |
| P02746 | Complement C1q subComponent subunit B | VVTFCDYAYNTFQVTTGGMVLK |
| P02746 | Complement C1q subComponent subunit B | GNLCVNLMR |
| P02746 | Complement C1q subComponent subunit B | IAFSATR |
| P02747 | Complement C1q subComponent subunit C | TNQVNSGGVLLR |
| P02747 | Complement C1q subComponent subunit C | FQSVFTVTR |
| P00736 | Complement C1r subComponent | TLDEFTIIQNLQPQYQFR |
| P00736 | Complement C1r subComponent | LVFQQFDLEPSEGCFYDYVK |
| P00736 | Complement C1r subComponent | LPVANPQACENWLR |
| P00736 | Complement C1r subComponent | IKDCGQPR |
| P00736 | Complement C1r subComponent | LFGEVTSPLFPKPYPNNFETTTVITVPTGYR |
| P00736 | Complement C1r subComponent | YTTTMGVNTYK |
| P00736 | Complement C1r subComponent | QGYQLIEGNQVLHSFTAVCQDDGTWHR |
| P09871 | Complement C1s subComponent | TNFDNDIALVR |
| P09871 | Complement C1s subComponent | LQVIFK |
| P09871 | Complement C1s subComponent | TMQENSTPRED |
| P06681 | Complement C2 | SSGQWQTPGATR |
| P06681 | Complement C2 | LNINLK |
| P06681 | Complement C2 | HAIILLTDGK |
| P0C0L5 | Complement C4-B | LQETSNWLLSQQQADGSFQDLSPVIHR |
| P0C0L5 | Complement C4-B | VLSLAQEQVGGSPEKLQETSNWLLSQQQADGSFQDLSPVIHR |
| P0C0L5 | Complement C4-B | DFALLSLQVPLK |
| P0C0L5 | Complement C4-B | LHLETDSLALVALGALDTALYAAGSK |
| P01031 | Complement C5 | ALVEGVDQLFTDYQIK |
| P01031 | Complement C5 | YGGGFYSTQDTINAIEGLTEYSLLVK |
| P01031 | Complement C5 | AFTECCVVASQLR |
| P01031 | Complement C5 | GGSASTWLTAFALR |
| P01031 | Complement C5 | TSTSEEVCSFYLK |
| P01031 | Complement C5 | TDAPDLPEENQAR |
| P01031 | Complement C5 | VFQFLEK |
| P01031 | Complement C5 | RMPITYDNGFLFIHTDKPVYTPDQSVK |
| P01031 | Complement C5 | MVETTAYALLTSLNLK |
| P01031 | Complement C5 | LQGTLPVEAR |
| P01031 | Complement C5 | LNLVATPLFLKPGIPYPIK |
| P01031 | Complement C5 | SDLGCGAGGGLNNANVFHLAGLTFLTNANADDSQENDEPCK |
| P01031 | Complement C5 | MSAVEGICTSESPVIDHQGTK |
| P01031 | Complement C5 | VTCTNAELVK |
| P01031 | Complement C5 | IDTALIK |
| P01031 | Complement C5 | ELSYYSLEDLNNK |
| P01031 | Complement C5 | EGMLSIMSYR |
| P01031 | Complement C5 | WLSEEQR |
| P01031 | Complement C5 | SIVSALKR |
| P01031 | Complement C5 | ATLLDIYK |
| P13671 | Complement Component C6 | IFDDFGTHYFTSGSLGGVYDLLYQFSSEELK |
| P13671 | Complement Component C6 | NSGLTEEEAK |
| P13671 | Complement Component C6 | ENPAVIDFELAPIVDLVR |
| P13671 | Complement Component C6 | GEVLDNSFTGGICK |
| P13671 | Complement Component C6 | DLTSLGHNENQQGSFSSQGGSSFSVPIFYSSK |
| P13671 | Complement Component C6 | CPINCLLGDFGPWSDCDPCIEK |
| P13671 | Complement Component C6 | GGNQLYCVK |
| P13671 | Complement Component C6 | HRQIVVDK |
| P13671 | Complement Component C6 | YYQENFCEQICSK |
| P13671 | Complement Component C6 | IGESIELTCPK |
| P13671 | Complement Component C6 | ALNHLPLEYNSALYSR |
| P13671 | Complement Component C6 | QLEWGLER |
| P13671 | Complement Component C6 | SVLRPSQFGGQPCTAPLVAFQPCIPSK |
| P10643 | Complement Component C7 | SSGWHFVVK |
| P10643 | Complement Component C7 | AASGTQNNVLR |
| P10643 | Complement Component C7 | SCVGETTESTQCEDEELEHLR |
| P07357 | Complement Component C8 alpha Chain | LGSLGAACEQTQTEGAK |
| P07358 | Complement Component C8 beta Chain | GGASEHITTLAYQELPTADLMQEWGDAVQYNPAIIK |
| P07358 | Complement Component C8 beta Chain | CEGFVCAQTGR |
| P07358 | Complement Component C8 beta Chain | DFGTHYITEAVLGGIYEYTLVMNK |
| P07358 | Complement Component C8 beta Chain | VKVEPLYELVTATDFAYSSTVR |
| P07360 | Complement Component C8 gamma Chain | SLPVSDSVLSGFEQR |
| P07360 | Complement Component C8 gamma Chain | VQEAHLTEDQIFYFPK |
| P02748 | Complement Component C9 | GTVIDVTDFVNWASSINDAPVLISQK |
| P02748 | Complement Component C9 | TSNFNAAISLK |
| P02748 | Complement Component C9 | AIEDYINEFSVR |
| P02748 | Complement Component C9 | VVEESELAR |
| P02748 | Complement Component C9 | CTDAVGDRR |
| P02748 | Complement Component C9 | DRDGNTLTYYR |
| P02748 | Complement Component C9 | TAGYGINILGMDPLSTPFDNEFYNGLCNR |
| P08174 | Complement deCay-aCCelerating faCtor | TSFPEDTVITYK |
| P08174 | Complement deCay-aCCelerating faCtor | QSVTYACNK |
| P08174 | Complement deCay-aCCelerating faCtor | QPYITQNYFPVGTVVEYECRPGYR |
| P08174 | Complement deCay-aCCelerating faCtor | GFTMIGEHSIYCTVNNDEGEWSGPPPECR |
| P00751 | Complement faCtor B | VSEADSSNADWVTK |
| P00751 | Complement faCtor B | YGLVTYATYPK |
| P00751 | Complement faCtor B | WSGQTAICDNGAGYCSNPGIPIGTRK |
| P00751 | Complement faCtor B | EKLQDEDLGFL |
| P00751 | Complement faCtor B | DFHINLFQVLPWLK |
| P00751 | Complement faCtor B | FLCTGGVSPYADPNTCRGDSGGPLIVHK |
| P00751 | Complement faCtor B | CLVNLIEK |
| P00751 | Complement faCtor B | LLQEGQALEYVCPSGFYPYPVQTR |
| P00751 | Complement faCtor B | EAGIPEFYDYDVALIK |
| P00751 | Complement faCtor B | DISEVVTPR |
| P00751 | Complement faCtor B | DMENLEDVFYQMIDESQSLSLCGMVWEHRK |
| P00751 | Complement faCtor B | HVIILMTDGLHNMGGDPITVIDEIRDLLYIGK |
| P00751 | Complement faCtor B | VASYGVKPR |
| P00751 | Complement faCtor B | DNEQHVFK |
| P00751 | Complement faCtor B | LEDSVTYHCSR |
| P00751 | Complement faCtor B | VKDISEVVTPR |
| P00751 | Complement faCtor B | WSGQTAICDNGAGYCSNPGIPIGTR |
| P00751 | Complement faCtor B | FLCTGGVSPYADPNTCR |
| P00751 | Complement faCtor B | YGQTIRPICLPCTEGTTR |
| P00751 | Complement faCtor B | GDSGGPLIVHKR |
| P00751 | Complement faCtor B | VSVGGEKR |
| P00751 | Complement faCtor B | ALFVSEEEKK |
| P00751 | Complement faCtor B | DAQYAPGYDK |
| P00751 | Complement faCtor B | KDNEQHVFK |
| P00751 | Complement faCtor B | QLNEINYEDHK |
| P00751 | Complement faCtor B | ALFVSEEEK |
| P00751 | Complement faCtor B | QKQVPAHAR |
| P00751 | Complement faCtor B | LPPTTTCQQQKEELLPAQDIK |
| P00751 | Complement faCtor B | ISVIRPSK |
| P08603 | Complement faCtor H | SSIDIENGFISESQYTYALK |
| P08603 | Complement faCtor H | LGYVTADGETSGSITCGK |
| P08603 | Complement faCtor H | AGEQVTYTCATYYK |
| P08603 | Complement faCtor H | WSSPPQCEGLPCK |
| P08603 | Complement faCtor H | RPCGHPGDTPFGTFTLTGGNVFEYGVK |
| P08603 | Complement faCtor H | CFEGFGIDGPAIAK |
| P08603 | Complement faCtor H | DTSCVNPPTVQNAYIVSR |
| P08603 | Complement faCtor H | SCDIPVFMNAR |
| P08603 | Complement faCtor H | NTEILTGSWSDQTYPEGTQAIYK |
| P08603 | Complement faCtor H | TKEEYGHSEVVEYYCNPR |
| P08603 | Complement faCtor H | TDCLSLPSFENAIPMGEK |
| P08603 | Complement faCtor H | HRTGDEITYQCR |
| P08603 | Complement faCtor H | SLGNVIMVCR |
| P08603 | Complement faCtor H | SPDVINGSPISQK |
| P08603 | Complement faCtor H | CTSTGWIPAPR |
| P08603 | Complement faCtor H | EQVQSCGPPPELLNGNVK |
| P08603 | Complement faCtor H | VSVLCQENYLIQEGEEITCKDGR |
| P08603 | Complement faCtor H | AVYTCNEGYQLLGEINYRECDTDGWTNDIPICEVVK |
| P08603 | Complement faCtor H | AQTTVTCMENGWSPTPR |
| P08603 | Complement faCtor H | VGEVLK |
| P08603 | Complement faCtor H | VSVLCQENYLIQEGEEITCK |
| P08603 | Complement faCtor H | DGRWQSIPLCVEK |
| P08603 | Complement faCtor H | SCDNPYIPNGDYSPLR |
| P08603 | Complement faCtor H | SSNLIILEEHLK |
| P08603 | Complement faCtor H | IVSSAMEPDREYHFGQAVR |
| P08603 | Complement faCtor H | LSYTCEGGFR |
| P08603 | Complement faCtor H | RPYFPVAVGK |
| P08603 | Complement faCtor H | EIMENYNIALR |
| P08603 | Complement faCtor H | IDVHLVPDR |
| P08603 | Complement faCtor H | SITCIHGVWTQLPQCVAIDK |
| P08603 | Complement faCtor H | DGWSAQPTCIK |
| P08603 | Complement faCtor H | SSQESYAHGTK |
| P08603 | Complement faCtor H | HGGLYHENMR |
| P08603 | Complement faCtor H | ECDTDGWTNDIPICEVVK |
| P08603 | Complement faCtor H | SIDVACHPGYALPK |
| P08603 | Complement faCtor H | SPPEISHGVVAHMSDSYQYGEEVTYK |
| P08603 | Complement faCtor H | TGESVEFVCKR |
| P08603 | Complement faCtor H | EFDHNSNIR |
| P08603 | Complement faCtor H | IIYKENER |
| P08603 | Complement faCtor H | WSHPPSCIK |
| P08603 | Complement faCtor H | WQSIPLCVEK |
| P08603 | Complement faCtor H | TGDEITYQCR |
| P08603 | Complement faCtor H | AVYTCNEGYQLLGEINYR |
| P08603 | Complement faCtor H | TGESVEFVCK |
| P08603 | Complement faCtor H | FSCKPGFTIVGPNSVQCYHFGLSPDLPICK |
| Q03591 | Complement faCtor H-related protein 1 | ITCTEEGWSPTPK |
| P36980 | Complement faCtor H-related protein 2 | ITCAEEGWSPTPK |
| P36980 | Complement faCtor H-related protein 2 | LQNNENNISCVER |
| P36980 | Complement faCtor H-related protein 2 | YKPFSQVPTGEVFYYSCEYNFVSPSK |
| P05156 | Complement faCtor I | TMGYQDFADVVCYTQK |
| P05156 | Complement faCtor I | ACDGINDCGDQSDELCCK |
| P05156 | Complement faCtor I | GLETSLAECTFTK |
| P05156 | Complement faCtor I | YQIWTTVVDWIHPDLK |
| P05156 | Complement faCtor I | VFSLQWGEVK |
| P05156 | Complement faCtor I | RAQLGDLPWQVAIK |
| P05156 | Complement faCtor I | HGNTDSEGIVEVK |
| P05156 | Complement faCtor I | EANVACLDLGFQQGADTQR |
| P05156 | Complement faCtor I | EMECAGTYDGSIDACK |
| P05156 | Complement faCtor I | ADSPMDDFFQCVNGK |
| P05156 | Complement faCtor I | AQLGDLPWQVAIK |
| P05156 | Complement faCtor I | EANVACLDLGFQQGADTQRR |
| P05156 | Complement faCtor I | DNERVFSLQWGEVK |
| P05156 | Complement faCtor I | IVIEYVDR |
| P05156 | Complement faCtor I | RIVIEYVDR |
| P05156 | Complement faCtor I | SSWSMR |
| P05156 | Complement faCtor I | KYTHLSCDK |
| P05156 | Complement faCtor I | DASGITCGGIYIGGCWILTAAHCLR |
| Q12860 | ContaCtin-1 | FIPLIPIPER |
| Q13098 | COP9 signalOSome Complex subunit 1 | FYESK |
| Q99829 | Copine-1 | EALAQTVLAEVPTQLVSYFR |
| O75131 | Copine-3 | VALNVSCANLLDK |
| O75131 | Copine-3 | EALAQCVLAEIPQQVVGYFNTYK |
| Q9UBG3 | Cornulin | AHQTGETVTGSGTQTQAGATQTVEQDSSHQTGR |
| P31146 | Coronin-1A | VSQTTWDSGFCAVNPK |
| P31146 | Coronin-1A | KLQATVQELQK |
| P31146 | Coronin-1A | DAGPLLISLK |
| P31146 | Coronin-1A | KSDLFQEDLYPPTAGPDPALTAEEWLGGR |
| P31146 | Coronin-1A | AAPEASGTPSSDAVSR |
| P31146 | Coronin-1A | HLEEPLSLQELDTSSGVLLPFFDPDTNIVYLCGK |
| Q9BR76 | Coronin-1B | VTWDSTFCAVNPK |
| Q9BR76 | Coronin-1B | NVLSDSRPAMAPGSSHLGAPASTTTAADATPSGSLAR |
| Q9BR76 | Coronin-1B | KSDLFQDDLYPDTAGPEAALEAEEWVSGR |
| P08185 | CortiCOSteroid-binding globulin | WSAGLTSSQVDLYIPK |
| P08185 | CortiCOSteroid-binding globulin | HLVALSPK |
| P08185 | CortiCOSteroid-binding globulin | QINSYVK |
| P08185 | CortiCOSteroid-binding globulin | HLVALSPKK |
| P08185 | CortiCOSteroid-binding globulin | MNTVIAALSR |
| P08185 | CortiCOSteroid-binding globulin | GLASANVDFAFSLYK |
| P08185 | CortiCOSteroid-binding globulin | SETEIHQGFQHLHQLFAK |
| Q9P1F3 | COStars family protein C6orf115 | CANLFEALVGTLK |
| Q86VP6 | Cullin-assoCiated NEDD8-dissoCiated protein 1 | ISGSILNELIGLVR |
| Q86VP6 | Cullin-assoCiated NEDD8-dissoCiated protein 1 | QGGLLVNFHPSILTCLLPQLTSPR |
| Q86VP6 | Cullin-assoCiated NEDD8-dissoCiated protein 1 | IDLRPVLGEGVPILASFLR |
| P01040 | Cystatin-A | TQVVAGTNYYIK |
| P01040 | Cystatin-A | TNETYGKLEAVQYK |
| P04080 | Cystatin-B | SQVVAGTNYFIK |
| P04080 | Cystatin-B | VFQSLPHENKPLTLSNYQTNK |
| P04080 | Cystatin-B | VHVGDEDFVHLR |
| P01034 | Cystatin-C | AFCSFQIYAVPWQGTMTLSK |
| P01034 | Cystatin-C | ALDFAVGEYNK |
| P01034 | Cystatin-C | ASNDMYHSR |
| P01036 | Cystatin-S | IIPGGIYDADLNDEWVQR |
| P01036 | Cystatin-S | QLCSFEIYEVPWEDR |
| P01036 | Cystatin-S | QLCSFEIYEVPWEDRMSLVNSR |
| P01037 | Cystatin-SN | QLCSFEIYEVPWENRR |
| P21291 | Cysteine and glyCine-riCh protein 1 | GFGFGQGAGALVHSE |
| P21291 | Cysteine and glyCine-riCh protein 1 | CSQAVYAAEK |
| P21291 | Cysteine and glyCine-riCh protein 1 | GLESTTLADKDGEIYCK |
| P52943 | Cysteine-riCh protein 2 | ASSVTTFTGEPNTCPR |
| P52943 | Cysteine-riCh protein 2 | GVNIGGAGSYIYEKPLAEGPQVTGPIEVPAAR |
| P21399 | CytoplasmiC aConitate hydratase | FVEFFGPGVAQLSIADR |
| P21399 | CytoplasmiC aConitate hydratase | VILQDFTGVPAVVDFAAMR |
| P21399 | CytoplasmiC aConitate hydratase | QAPQTIHLPSGEILDVFDAAER |
| P21399 | CytoplasmiC aConitate hydratase | DFNDPSQDPDFTQVVELDLK |
| P21399 | CytoplasmiC aConitate hydratase | GPFLLGIK |
| P21399 | CytoplasmiC aConitate hydratase | VLLEAAIR |
| P21399 | CytoplasmiC aConitate hydratase | SPPFFENLTLDLQPPK |
| P21399 | CytoplasmiC aConitate hydratase | IDFEKEPLGVNAK |
| P21399 | CytoplasmiC aConitate hydratase | RGNDAVMAR |
| P21399 | CytoplasmiC aConitate hydratase | SNLVGMGVIPLEYLPGENADALGLTGQER |
| P21399 | CytoplasmiC aConitate hydratase | ANYLASPPLVIAYAIAGTIR |
| Q14204 | CytoplasmiC dynein 1 heavy Chain 1 | LVPLLLEDGGEAPAALEAALEEK |
| Q07065 | CytOSkeleton-assoCiated protein 4 | GAHPSGGADDVAK |
| Q96KP4 | CytOSoliC non-speCifiC dipeptidase | NVMLLPVGSADDGAHSQNEK |
| Q96KP4 | CytOSoliC non-speCifiC dipeptidase | QLGGSVELVDIGK |
| Q96KP4 | CytOSoliC non-speCifiC dipeptidase | WVAIQSVSAWPEK |
| Q96KP4 | CytOSoliC non-speCifiC dipeptidase | GICYFFIEVECSNK |
| Q96KP4 | CytOSoliC non-speCifiC dipeptidase | LYDDIDFDIEEFAK |
| Q96KP4 | CytOSoliC non-speCifiC dipeptidase | DVDYVCISDNYWLGK |
| Q96KP4 | CytOSoliC non-speCifiC dipeptidase | DLHSGVYGGSVHEAMTDLILLMGSLVDK |
| Q96KP4 | CytOSoliC non-speCifiC dipeptidase | LVPNMTPEVVGEQVTSYLTK |
| Q96KP4 | CytOSoliC non-speCifiC dipeptidase | LNRYNYIEGTK |
| Q96KP4 | CytOSoliC non-speCifiC dipeptidase | MMEVAAADVK |
| Q96KP4 | CytOSoliC non-speCifiC dipeptidase | DLHSGVYGGSVHEAMTDLILLMGSLVDKR |
| Q96KP4 | CytOSoliC non-speCifiC dipeptidase | FCLEGMEESGSEGLDELIFARK |
| Q96KP4 | CytOSoliC non-speCifiC dipeptidase | YIDENQDRYIK |
| Q96KP4 | CytOSoliC non-speCifiC dipeptidase | GSTDDKGPVAGWINALEAYQK |
| Q96KP4 | CytOSoliC non-speCifiC dipeptidase | RGNILIPGINEAVAAVTEEEHK |
| Q96KP4 | CytOSoliC non-speCifiC dipeptidase | EGGSIPVTLTFQEATGK |
| Q96KP4 | CytOSoliC non-speCifiC dipeptidase | TGQEIPVNVR |
| Q96KP4 | CytOSoliC non-speCifiC dipeptidase | TVCIYGHLDVQPAALEDGWDSEPFTLVERDGK |
| Q96KP4 | CytOSoliC non-speCifiC dipeptidase | TVFGVEPDLTREGGSIPVTLTFQEATGK |
| Q96KP4 | CytOSoliC non-speCifiC dipeptidase | QKLPDGSEIPLPPILLGR |
| Q96KP4 | CytOSoliC non-speCifiC dipeptidase | LPDGSEIPLPPILLGR |
| Q96KP4 | CytOSoliC non-speCifiC dipeptidase | RMMEVAAADVK |
| Q96KP4 | CytOSoliC non-speCifiC dipeptidase | TVFGVEPDLTR |
| Q96KP4 | CytOSoliC non-speCifiC dipeptidase | LVPNMTPEVVGEQVTSYLTKK |
| Q96KP4 | CytOSoliC non-speCifiC dipeptidase | KKPCITYGLR |
| Q96KP4 | CytOSoliC non-speCifiC dipeptidase | SPNEFK |
| O43175 | D-3-phOSphoglyCerate dehydrogenase | DLPLLLFR |
| Q99489 | D-aspartate oxidase | KmTEAELK |
| P30046 | D-dopaChrome deCarboxylase | PFLELDTNLPANRVPAGLEK |
| P30046 | D-dopaChrome deCarboxylase | RLCAAAASILGKPADR |
| P30046 | D-dopaChrome deCarboxylase | FFPLESWQIGK |
| Q14185 | DediCator of Cytokinesis protein 1 | DDLEKEK |
| Q9UGM3 | Deleted in malignant brain tumors 1 protein | GSWGTVCDDYWDTNDANVVCR |
| Q9UGM3 | Deleted in malignant brain tumors 1 protein | FGQGSGPIVLDDVR |
| Q9UGM3 | Deleted in malignant brain tumors 1 protein | QLGCGWATSAPGNAR |
| P13716 | Delta-aminolevuliniC aCid dehydratase | LAEVALAYAK |
| P13716 | Delta-aminolevuliniC aCid dehydratase | CVLIFGVPSR |
| O43583 | Density-regulated protein | LTVENSPKQEAGISEGQGTAGEEEEK |
| O43598 | DeoxyribonuCleOSide 5'-monophOSphate N-glyCOSidase | FGTVLTEHVAAAELGAR |
| O43598 | DeoxyribonuCleOSide 5'-monophOSphate N-glyCOSidase | VLSAMIR |
| Q14126 | Desmoglein-2 | IVSLEPAYPPVFYLNK |
| P60981 | Destrin | YALYDASFETK |
| P60981 | Destrin | FQGIKHECQANGPEDLNR |
| P09417 | Dihydropteridine reduCtase | GAVHQLCQSLAGK |
| P09417 | Dihydropteridine reduCtase | AALDGTPGMIGYGMAK |
| P09417 | Dihydropteridine reduCtase | EGGLLTLAGAK |
| Q14195 | Dihydropyrimidinase-related protein 3 | AITIASQTNCPLYVTK |
| Q14195 | Dihydropyrimidinase-related protein 3 | GMYDGPVFDLTTTPK |
| Q14195 | Dihydropyrimidinase-related protein 3 | RIVAPPGGR |
| Q9UHL4 | Dipeptidyl peptidase 2 | DLTQLFMFAR |
| Q16832 | DisCoidin domain-Containing reCeptor 2 | KLLTFK |
| Q16531 | DNA damage-binding protein 1 | QGQGQLVTCSGAFK |
| Q16531 | DNA damage-binding protein 1 | IEVQDTSGGTTALRPSASTQALSSSVSSSK |
| Q16531 | DNA damage-binding protein 1 | LLASINSTVR |
| P09884 | DNA polymerase alpha CatalytiC subunit | IKGPCWLEVK |
| Q14566 | DNA repliCation liCensing faCtor MCM6 | RGVLLMLFGGVPK |
| P27695 | DNA-(apuriniC or apyrimidiniC site) lyase | QGFGELLQAVPLADSFR |
| P27695 | DNA-(apuriniC or apyrimidiniC site) lyase | GLDWVKEEAPDILCLQETK |
| P27695 | DNA-(apuriniC or apyrimidiniC site) lyase | ICSWNVDGLR |
| P27695 | DNA-(apuriniC or apyrimidiniC site) lyase | LDYFLLSHSLLPALCDSK |
| P27695 | DNA-(apuriniC or apyrimidiniC site) lyase | EAAGEGPALYEDPPDQK |
| P27695 | DNA-(apuriniC or apyrimidiniC site) lyase | KPLVLCGDLNVAHEEIDLRNPK |
| P27695 | DNA-(apuriniC or apyrimidiniC site) lyase | ALGSDHCPITLYLAL |
| O75937 | DnaJ homolog subfamily C member 8 | LTRPGSSYFNLNPFEVLQIDPEVTDEEIKK |
| O75937 | DnaJ homolog subfamily C member 8 | LTRPGSSYFNLNPFEVLQIDPEVTDEEIK |
| P39656 | DoliChyl-diphOSphooligOSaCCharide--protein glyCOSyltransferase 48 kDa subunit | GVGMVADPDNPLVLDILTGSSTSYSFFPDKPITQYPHAVGK |
| P04844 | DoliChyl-diphOSphooligOSaCCharide--protein glyCOSyltransferase subunit 2 | SIVEEIEDLVAR |
| Q9UJU6 | Drebrin-like protein | NRNEQESAVHPR |
| Q02750 | Dual speCifiCity mitogen-aCtivated protein kinase kinase 1 | LCDFGVSGQLIDSMANSFVGTR |
| Q13561 | DynaCtin subunit 2 | CDQDAQNPLSAGLQGACLMETVELLQAK |
| Q8IVF4 | Dynein heavy Chain 10, axonemal | SSQFWK |
| P63167 | Dynein light Chain 1, CytoplasmiC | KYNPTWHCIVGR |
| Q14118 | DystroglyCan | ATSITVTGSGSCR |
| Q14258 | E3 ubiquitin/ISG15 ligase TRIM25 | NTVLCNVVEQFLQADLAR |
| Q13822 | ECtonuCleotide pyrophOSphatase/phOSphodiesterase family member 2 | GRCFELQEAGPPDCR |
| Q13822 | ECtonuCleotide pyrophOSphatase/phOSphodiesterase family member 2 | SYTSCCHDFDELCLK |
| Q13822 | ECtonuCleotide pyrophOSphatase/phOSphodiesterase family member 2 | IVGQLMDGLK |
| Q9H4M9 | EH domain-Containing protein 1 | MQELLQTQDFSK |
| Q9H4M9 | EH domain-Containing protein 1 | FMCAQLPNPVLDSISIIDTPGILSGEK |
| P68104 | Elongation faCtor 1-alpha 1 | NMITGTSQADCAVLIVAAGVGEFEAGISK |
| P68104 | Elongation faCtor 1-alpha 1 | VETGVLKPGMVVTFAPVNVTTEVK |
| P68104 | Elongation faCtor 1-alpha 1 | EHALLAYTLGVK |
| P68104 | Elongation faCtor 1-alpha 1 | SGDAAIVDMVPGKPMCVESFSDYPPLGR |
| P68104 | Elongation faCtor 1-alpha 1 | IGGIGTVPVGR |
| P68104 | Elongation faCtor 1-alpha 1 | YYVTIIDAPGHR |
| P68104 | Elongation faCtor 1-alpha 1 | THINIVVIGHVDSGK |
| P68104 | Elongation faCtor 1-alpha 1 | QTVAVGVIK |
| P68104 | Elongation faCtor 1-alpha 1 | IGGIGTVPVGRVETGVLKPGMVVTFAPVNVTTEVK |
| P68104 | Elongation faCtor 1-alpha 1 | STTTGHLIYK |
| P24534 | Elongation faCtor 1-beta | SPAGLQVLNDYLADK |
| P24534 | Elongation faCtor 1-beta | LAQYESKK |
| P24534 | Elongation faCtor 1-beta | AKKPALVAK |
| P29692 | Elongation faCtor 1-delta | IASLEVENQSLR |
| P29692 | Elongation faCtor 1-delta | GVVQELQQAISK |
| P26641 | Elongation faCtor 1-gamma | ALIAAQYSGAQVR |
| P26641 | Elongation faCtor 1-gamma | KLDPGSEETQTLVR |
| P26641 | Elongation faCtor 1-gamma | ILGLLDAYLK |
| P26641 | Elongation faCtor 1-gamma | TFLVGER |
| P26641 | Elongation faCtor 1-gamma | AKDPFAHLPK |
| P13639 | Elongation faCtor 2 | IWCFGPDGTGPNILTDITK |
| P13639 | Elongation faCtor 2 | TFCQLILDPIFK |
| P13639 | Elongation faCtor 2 | AYLPVNESFGFTADLR |
| P13639 | Elongation faCtor 2 | STAISLFYELSENDLNFIK |
| P13639 | Elongation faCtor 2 | YVEPIEDVPCGNIVGLVGVDQFLVK |
| P13639 | Elongation faCtor 2 | LMEPIYLVEIQCPEQVVGGIYGVLNR |
| P13639 | Elongation faCtor 2 | VFSGLVSTGLK |
| P13639 | Elongation faCtor 2 | EGIPALDNFLDKL |
| P13639 | Elongation faCtor 2 | EGALCEENMR |
| P13639 | Elongation faCtor 2 | VNFTVDQIR |
| P13639 | Elongation faCtor 2 | ETVSEESNVLCLSK |
| P13639 | Elongation faCtor 2 | ALLELQLEPEELYQTFQR |
| P13639 | Elongation faCtor 2 | CLYASVLTAQPR |
| P13639 | Elongation faCtor 2 | ARPFPDGLAEDIDKGEVSAR |
| P13639 | Elongation faCtor 2 | NMSVIAHVDHGK |
| P13639 | Elongation faCtor 2 | YRCELLYEGPPDDEAAMGIK |
| P13639 | Elongation faCtor 2 | CELLYEGPPDDEAAMGIK |
| P13639 | Elongation faCtor 2 | STLTDSLVCK |
| P13639 | Elongation faCtor 2 | KIWCFGPDGTGPNILTDITK |
| P13639 | Elongation faCtor 2 | FAAKGEGQLGPAER |
| P13639 | Elongation faCtor 2 | SDPVVSYRETVSEESNVLCLSK |
| P13639 | Elongation faCtor 2 | WLPAGDALLQMITIHLPSPVTAQK |
| P13639 | Elongation faCtor 2 | DKEGKPLLK |
| P13639 | Elongation faCtor 2 | GVQYLNEIKDSVVAGFQWATK |
| P13639 | Elongation faCtor 2 | FSVSPVVR |
| P30040 | EndoplasmiC retiCulum resident protein 29 | GQGVYLGMPGCLPVYDALAGEFIR |
| P14625 | Endoplasmin | FAFQAEVNR |
| P14625 | Endoplasmin | LIINSLYK |
| P14625 | Endoplasmin | FQSSHHPTDITSLDQYVER |
| P14625 | Endoplasmin | LGVIEDHSNR |
| O60869 | Endothelial differentiation-related faCtor 1 | INEKPQVIADYESGR |
| P30084 | Enoyl-CoA hydratase, mitoChondrial | ICPVETLVEEAIQCAEK |
| P61916 | Epididymal seCretory protein E1 | EVNVSPCPTQPCQLSK |
| P61916 | Epididymal seCretory protein E1 | AVVHGILMGVPVPFPIPEPDGCK |
| P27105 | ErythroCyte band 7 integral membrane protein | VQNATLAVANITNADSATR |
| P27105 | ErythroCyte band 7 integral membrane protein | EASMVITESPAALQLR |
| P27105 | ErythroCyte band 7 integral membrane protein | VIAAEGEmNASR |
| P27105 | ErythroCyte band 7 integral membrane protein | YLQTLTTIAAEK |
| P27105 | ErythroCyte band 7 integral membrane protein | IPVQLQR |
| P27105 | ErythroCyte band 7 integral membrane protein | AMAAEAEASR |
| P27105 | ErythroCyte band 7 integral membrane protein | NSTIVFPLPIDMLQGIIGAK |
| P27105 | ErythroCyte band 7 integral membrane protein | GPGLFFILPCTDSFIK |
| P27105 | ErythroCyte band 7 integral membrane protein | AMAAEAEASREAR |
| P27105 | ErythroCyte band 7 integral membrane protein | DVKLPVQLQR |
| P27105 | ErythroCyte band 7 integral membrane protein | LLAQTTLR |
| P16452 | ErythroCyte membrane protein band 4.2 | VVTTFASAQGTGGR |
| Q9H0W9 | Ester hydrolase C11orf54 | IAEVGGVPYLLPLVNQK |
| Q9H0W9 | Ester hydrolase C11orf54 | DNFADVQVSVVDCPDLTKEPFTFPVK |
| Q9H0W9 | Ester hydrolase C11orf54 | TGPLNFVTCMR |
| Q9H0W9 | Ester hydrolase C11orf54 | APLVCLPVFVSR |
| P60842 | EukaryotiC initiation faCtor 4A-I | LQMEAPHIIVGTPGR |
| P60842 | EukaryotiC initiation faCtor 4A-I | MFVLDEADEMLSR |
| P60842 | EukaryotiC initiation faCtor 4A-I | VLITTDLLAR |
| P41091 | EukaryotiC translation initiation faCtor 2 subunit 3 | IVLTNPVCTEVGEK |
| P55884 | EukaryotiC translation initiation faCtor 3 subunit B | AQAVSEDAGGNEGR |
| O15372 | EukaryotiC translation initiation faCtor 3 subunit H | EGTGSTATSSSSTAGAAGK |
| Q13347 | EukaryotiC translation initiation faCtor 3 subunit I | HVLTGSADNSCR |
| P23588 | EukaryotiC translation initiation faCtor 4B | SQSSDTEQQSPTSGGGK |
| P23588 | EukaryotiC translation initiation faCtor 4B | SPPYTAFLGNLPYDVTEESIK |
| Q15056 | EukaryotiC translation initiation faCtor 4H | GFCYVEFDEVDSLK |
| Q15056 | EukaryotiC translation initiation faCtor 4H | EALTYDGALLGDR |
| Q15056 | EukaryotiC translation initiation faCtor 4H | ELPTEPPYTAYVGNLPFNTVQGDIDAIFK |
| Q9GZV4 | EukaryotiC translation initiation faCtor 5A-2 | VHLVGIDIFTGK |
| Q9GZV4 | EukaryotiC translation initiation faCtor 5A-2 | KYEDICPSTHNMDVPNIK |
| Q9GZV4 | EukaryotiC translation initiation faCtor 5A-2 | IVEMSTSK |
| P56537 | EukaryotiC translation initiation faCtor 6 | HGLLVPNNTTDQELQHIR |
| P56537 | EukaryotiC translation initiation faCtor 6 | LNEAQPSTIATSMRDSLIDSLT |
| P56537 | EukaryotiC translation initiation faCtor 6 | TSIEDQDELSSLLQVPLVAGTVNR |
| P56537 | EukaryotiC translation initiation faCtor 6 | LNEAQPSTIATSMR |
| Q16610 | ExtraCellular matrix protein 1 | ACPSHQPDISSGLELPFPPGVPTLDNIK |
| Q16610 | ExtraCellular matrix protein 1 | VTPNLMGHLCGNQR |
| Q16610 | ExtraCellular matrix protein 1 | EVGPPLPQEAVPLQK |
| Q16610 | ExtraCellular matrix protein 1 | FSCFQEEAPQPHYQLR |
| Q16610 | ExtraCellular matrix protein 1 | SLPMDHPDSSQHGPPFEGQSQVQPPPSQEATPLQQEK |
| P08294 | ExtraCellular superoxide dismutase [Cu-Zn] | RLACCVVGVCGPGLWER |
| P08294 | ExtraCellular superoxide dismutase [Cu-Zn] | LACCVVGVCGPGLWER |
| P08294 | ExtraCellular superoxide dismutase [Cu-Zn] | AGLAASLAGPHSIVGR |
| P08294 | ExtraCellular superoxide dismutase [Cu-Zn] | VTGVVLFR |
| P08294 | ExtraCellular superoxide dismutase [Cu-Zn] | DDDGALHAACQVQPSATLDAAQPR |
| P15311 | Ezrin | SQEQLAAELAEYTAK |
| P15311 | Ezrin | FYPEDVAEELIQDITQK |
| P15311 | Ezrin | IALLEEAR |
| P15311 | Ezrin | ELSEQIQR |
| P15311 | Ezrin | IAQDLEMYGINYFEIK |
| P15311 | Ezrin | EGILSDEIYCPPETAVLLGSYAVQAK |
| P15311 | Ezrin | DNAMLEYLK |
| P15311 | Ezrin | SGYLSSER |
| P15311 | Ezrin | THNDIIHNENMR |
| P52907 | F-aCtin-Capping protein subunit alpha-1 | EASDPQPEEADGGLK |
| P52907 | F-aCtin-Capping protein subunit alpha-1 | FITHAPPGEFNEVFNDVR |
| P52907 | F-aCtin-Capping protein subunit alpha-1 | FTITPPTAQVVGVLK |
| P52907 | F-aCtin-Capping protein subunit alpha-1 | IIENAENEYQTAISENYQTMSDTTFK |
| P47755 | F-aCtin-Capping protein subunit alpha-2 | IVEAAENEYQTAISENYQTMSDTTFK |
| P47755 | F-aCtin-Capping protein subunit alpha-2 | FTITPSTTQVVGILK |
| Q96AE4 | Far upstream element-binding protein 1 | SCMLTGTPESVQSAK |
| Q96AE4 | Far upstream element-binding protein 1 | SVMTEEYKVPDGMVGFIIGR |
| Q96AE4 | Far upstream element-binding protein 1 | IQIAPDSGGLPER |
| Q92945 | Far upstream element-binding protein 2 | IGGGIDVPVPR |
| Q92945 | Far upstream element-binding protein 2 | VQISPDSGGLPER |
| Q92945 | Far upstream element-binding protein 2 | MILIQDGSQNTNVDKPLR |
| Q92945 | Far upstream element-binding protein 2 | IINDLLQSLR |
| P14324 | Farnesyl pyrophOSphate synthase | KQDADSLQR |
| Q9UNN5 | FAS-assoCiated faCtor 1 | SLHLPK |
| P15090 | Fatty aCid-binding protein, adipoCyte | NTEISFILGQEFDEVTADDRK |
| P15090 | Fatty aCid-binding protein, adipoCyte | LVSSENFDDYMK |
| Q5SYB0 | FERM and PDZ domain-Containing protein 1 | DIILTVK |
| P02794 | Ferritin heavy Chain | MGAPESGLAEYLFDK |
| P02794 | Ferritin heavy Chain | QINLELYASYVYLSMSYYFDRDDVALK |
| P02794 | Ferritin heavy Chain | IFLQDIKKPDCDDWESGLNAMECALHLEK |
| P02794 | Ferritin heavy Chain | NVNQSLLELHK |
| P02794 | Ferritin heavy Chain | QNYHQDSEAAINR |
| P02794 | Ferritin heavy Chain | KPDCDDWESGLNAMECALHLEK |
| P02794 | Ferritin heavy Chain | YFLHQSHEEREHAEK |
| P02794 | Ferritin heavy Chain | LATDKNDPHLCDFIETHYLNEQVK |
| P02794 | Ferritin heavy Chain | ELGDHVTNLRK |
| P02794 | Ferritin heavy Chain | HTLGDSDNES |
| P02792 | Ferritin light Chain | LGGPEAGLGEYLFER |
| P02792 | Ferritin light Chain | KLNQALLDLHALGSAR |
| P02792 | Ferritin light Chain | KMGDHLTNLHR |
| P02792 | Ferritin light Chain | AAMALEK |
| P02792 | Ferritin light Chain | ALFQDIK |
| Q86WI1 | FibroCystin-L | DLSQSMTPFTYAVSLTPLITAVSPK |
| O75636 | FiColin-3 | ALPVFCDMDTEGGGWLVFQR |
| O75636 | FiColin-3 | YAVSEAAAHK |
| O75369 | Filamin-B | VLFASQEIPASPFR |
| O75369 | Filamin-B | VQAQGPGLK |
| Q12841 | Follistatin-related protein 1 | ICANVFCGAGR |
| Q12841 | Follistatin-related protein 1 | ICANVFCGAGRECAVTEK |
| Q12841 | Follistatin-related protein 1 | LSFQEFLK |
| Q12841 | Follistatin-related protein 1 | KCALEDETYADGAETEVDCNR |
| P09467 | FruCtOSe-1,6-bisphOSphatase 1 | APVILGSPDDVLEFLK |
| P09467 | FruCtOSe-1,6-bisphOSphatase 1 | LLYECNPMAYVMEK |
| P09467 | FruCtOSe-1,6-bisphOSphatase 1 | GTGELTQLLNSLCTAVK |
| P04075 | FruCtOSe-bisphOSphate aldolase A | YTPSGQAGAAASESLFVSNHAY |
| P04075 | FruCtOSe-bisphOSphate aldolase A | GVVPLAGTNGETTTQGLDGLSER |
| P04075 | FruCtOSe-bisphOSphate aldolase A | GILAADESTGSIAK |
| P04075 | FruCtOSe-bisphOSphate aldolase A | IGEHTPSALAIMENANVLAR |
| P04075 | FruCtOSe-bisphOSphate aldolase A | AAQEEYVK |
| P04075 | FruCtOSe-bisphOSphate aldolase A | QLLLTADDRVNPCIGGVILFHETLYQK |
| P04075 | FruCtOSe-bisphOSphate aldolase A | ALSDHHIYLEGTLLKPNMVTPGHACTQK |
| P04075 | FruCtOSe-bisphOSphate aldolase A | ELSDIAHR |
| P04075 | FruCtOSe-bisphOSphate aldolase A | ALQASALK |
| P04075 | FruCtOSe-bisphOSphate aldolase A | FSHEEIAMATVTALRR |
| P04075 | FruCtOSe-bisphOSphate aldolase A | GGVVGIKVDK |
| P04075 | FruCtOSe-bisphOSphate aldolase A | RALANSLACQGK |
| P04075 | FruCtOSe-bisphOSphate aldolase A | YASICQQNGIVPIVEPEILPDGDHDLK |
| P04075 | FruCtOSe-bisphOSphate aldolase A | AAQEEYVKR |
| P04075 | FruCtOSe-bisphOSphate aldolase A | ALANSLACQGK |
| P04075 | FruCtOSe-bisphOSphate aldolase A | GILAADESTGSIAKR |
| P04075 | FruCtOSe-bisphOSphate aldolase A | TVPPAVTGITFLSGGQSEEEASINLNAINK |
| P04075 | FruCtOSe-bisphOSphate aldolase A | ADDGRPFPQVIK |
| P04075 | FruCtOSe-bisphOSphate aldolase A | PYQYPALTPEQKK |
| P04075 | FruCtOSe-bisphOSphate aldolase A | VDKGVVPLAGTNGETTTQGLDGLSER |
| P04075 | FruCtOSe-bisphOSphate aldolase A | PYQYPALTPEQK |
| P04075 | FruCtOSe-bisphOSphate aldolase A | KELSDIAHR |
| P04075 | FruCtOSe-bisphOSphate aldolase A | SKGGVVGIK |
| P04075 | FruCtOSe-bisphOSphate aldolase A | QLLLTADDR |
| P04075 | FruCtOSe-bisphOSphate aldolase A | CPLLKPWALTFSYGR |
| P09972 | FruCtOSe-bisphOSphate aldolase C | GVVPLAGTDGETTTQGLDGLSER |
| P09972 | FruCtOSe-bisphOSphate aldolase C | VDKGVVPLAGTDGETTTQGLDGLSER |
| P16930 | FumarylaCetoaCetase | SFGTTVSPWVVPMDALMPFAVPNPK |
| Q08380 | GaleCtin-3-binding protein | AAFGQGSGPIMLDEVQCTGTEASLADCK |
| Q08380 | GaleCtin-3-binding protein | GQWGTVCDNLWDLTDASVVCR |
| Q08380 | GaleCtin-3-binding protein | IYTSPTWSAFVTDSSWSAR |
| Q08380 | GaleCtin-3-binding protein | LADGGATNQGR |
| Q08380 | GaleCtin-3-binding protein | ALMLCEGLFVADVTDFEGWK |
| Q08380 | GaleCtin-3-binding protein | ELSEALGQIFDSQR |
| Q08380 | GaleCtin-3-binding protein | TLQALEFHTVPFQLLAR |
| Q08380 | GaleCtin-3-binding protein | ASHEEVEGLVEK |
| Q08380 | GaleCtin-3-binding protein | AVDTWSWGER |
| Q08380 | GaleCtin-3-binding protein | RVSWSLVYLPTIQSCWNYGFSCSSDELPVLGLTK |
| Q08380 | GaleCtin-3-binding protein | SDLAVPSELALLK |
| Q08380 | GaleCtin-3-binding protein | STHTLDLSR |
| Q08380 | GaleCtin-3-binding protein | RIDITLSSVK |
| Q08380 | GaleCtin-3-binding protein | LADGGATNQGRVEIFYR |
| Q08380 | GaleCtin-3-binding protein | SGGSDRTIAYENK |
| Q08380 | GaleCtin-3-binding protein | YYPYQSFQTPQHPSFLFQDK |
| Q08380 | GaleCtin-3-binding protein | YSSDYFQAPSDYR |
| Q08380 | GaleCtin-3-binding protein | STSSFPCPAGHFNGFR |
| Q08380 | GaleCtin-3-binding protein | LASAYGAR |
| Q08380 | GaleCtin-3-binding protein | IDITLSSVK |
| Q08380 | GaleCtin-3-binding protein | SQLVYQSR |
| Q08380 | GaleCtin-3-binding protein | KSQLVYQSR |
| O00182 | GaleCtin-9 | FEDGGYVVCNTR |
| P09104 | Gamma-enolase | VNQIGSVTEAIQACK |
| P09104 | Gamma-enolase | DATNVGDEGGFAPNILENSEALELVK |
| P09104 | Gamma-enolase | IEEELGDEAR |
| O75223 | Gamma-glutamylCyClotransferase | DVTGPDEESFLYFAYGSNLLTER |
| O75223 | Gamma-glutamylCyClotransferase | SNLNSLDEQEGVK |
| O75223 | Gamma-glutamylCyClotransferase | TSQTWHGGIATIFQSPGDEVWGVVWK |
| O75223 | Gamma-glutamylCyClotransferase | VATQEGKEITCR |
| O75223 | Gamma-glutamylCyClotransferase | VSEEIEDIIK |
| O75223 | Gamma-glutamylCyClotransferase | NPSAAFFCVAR |
| O75223 | Gamma-glutamylCyClotransferase | AIEPNDYTGKVSEEIEDIIK |
| P13284 | Gamma-interferon-induCible lysOSomal thiol reduCtase | GMQLMHANAQR |
| P13284 | Gamma-interferon-induCible lysOSomal thiol reduCtase | SLPLCLQLYAPGLSPDTIMECAMGDR |
| P13284 | Gamma-interferon-induCible lysOSomal thiol reduCtase | AFLIR |
| O00451 | GDNF family reCeptor alpha-2 | MLFCSCQDQACAER |
| O00451 | GDNF family reCeptor alpha-2 | ANELCAAESNCSSR |
| Q13630 | GDP-L-fuCOSe synthase | DADLTDTAQTR |
| Q13630 | GDP-L-fuCOSe synthase | VVADGAGLPGEDWVFVSSK |
| O60547 | GDP-mannOSe 4,6 dehydratase | YYRPTEVDFLQGDCTK |
| O60547 | GDP-mannOSe 4,6 dehydratase | FYQASTSELYGK |
| O60547 | GDP-mannOSe 4,6 dehydratase | IINEVKPTEIYNLGAQSHVK |
| O60547 | GDP-mannOSe 4,6 dehydratase | IYLGQLECFSLGNLDAK |
| P46926 | GluCOSamine-6-phOSphate isomerase 1 | TLAMDTILANAR |
| P46926 | GluCOSamine-6-phOSphate isomerase 1 | AFALYK |
| Q06210 | GluCOSamine--fruCtOSe-6-phOSphate aminotransferase [isomerizing] 1 | AVQTLQMELQQIMK |
| Q06210 | GluCOSamine--fruCtOSe-6-phOSphate aminotransferase [isomerizing] 1 | CQNALQQVVAR |
| Q06210 | GluCOSamine--fruCtOSe-6-phOSphate aminotransferase [isomerizing] 1 | ETDCGVHINAGPEIGVASTK |
| P06744 | GluCOSe-6-phOSphate isomerase | ILLANFLAQTEALMR |
| P06744 | GluCOSe-6-phOSphate isomerase | IFVQGIIWDINSFDQWGVELGK |
| P06744 | GluCOSe-6-phOSphate isomerase | TFTTQETITNAETAK |
| P06744 | GluCOSe-6-phOSphate isomerase | TLAQLNPESSLFIIASK |
| P06744 | GluCOSe-6-phOSphate isomerase | FAAYFQQGDMESNGK |
| P06744 | GluCOSe-6-phOSphate isomerase | NAPVLLALLGIWYINCFGCETHAMLPYDQYLHR |
| P06744 | GluCOSe-6-phOSphate isomerase | ELQAAGKSPEDLER |
| P06744 | GluCOSe-6-phOSphate isomerase | VWYVSNIDGTHIAK |
| P06744 | GluCOSe-6-phOSphate isomerase | NLVTEDVMR |
| P06744 | GluCOSe-6-phOSphate isomerase | TITDVINIGIGGSDLGPLMVTEALKPYSSGGPR |
| P06744 | GluCOSe-6-phOSphate isomerase | INYTEGR |
| P06744 | GluCOSe-6-phOSphate isomerase | HFVALSTNTTK |
| P06744 | GluCOSe-6-phOSphate isomerase | EWFLQAAK |
| P06744 | GluCOSe-6-phOSphate isomerase | SNTPILVDGKDVMPEVNK |
| P06744 | GluCOSe-6-phOSphate isomerase | EWFLQAAKDPSAVAK |
| P06744 | GluCOSe-6-phOSphate isomerase | KIEPELDGSAQVTSHDASTNGLINFIK |
| P06744 | GluCOSe-6-phOSphate isomerase | AVLHVALR |
| P06744 | GluCOSe-6-phOSphate isomerase | SPEDLER |
| P14314 | GluCOSidase 2 subunit beta | SLEDQVEMLR |
| P14314 | GluCOSidase 2 subunit beta | TVKEEAEKPER |
| P14314 | GluCOSidase 2 subunit beta | LWEEQLAAAK |
| P35754 | Glutaredoxin-1 | DCIGGCSDLVSLQQSGELLTR |
| P00390 | Glutathione reduCtase, mitoChondrial | SFDSMISTNCTEELENAGVEVLK |
| P00390 | Glutathione reduCtase, mitoChondrial | TYSTSFTPMYHAVTK |
| P00390 | Glutathione reduCtase, mitoChondrial | GIYAVGDVCGK |
| P00390 | Glutathione reduCtase, mitoChondrial | MGATKADFDNTVAIHPTSSEELVTLR |
| P00390 | Glutathione reduCtase, mitoChondrial | LGGTCVNVGCVPK |
| P00390 | Glutathione reduCtase, mitoChondrial | ALLTPVAIAAGR |
| P00390 | Glutathione reduCtase, mitoChondrial | RAAELGAR |
| P00390 | Glutathione reduCtase, mitoChondrial | ADFDNTVAIHPTSSEELVTLR |
| P00390 | Glutathione reduCtase, mitoChondrial | GHAAFTSDPKPTIEVSGK |
| P21266 | Glutathione S-transferase Mu 3 | IAAYLQSDQFCK |
| P21266 | Glutathione S-transferase Mu 3 | ITQSNAILR |
| P21266 | Glutathione S-transferase Mu 3 | KHNMCGETEEEK |
| P21266 | Glutathione S-transferase Mu 3 | LCYSSDHEK |
| P78417 | Glutathione S-transferase omega-1 | LNECVDHTPK |
| P78417 | Glutathione S-transferase omega-1 | VPSLVGSFIR |
| P78417 | Glutathione S-transferase omega-1 | LWMAAMKEDPTVSALLTSEK |
| P78417 | Glutathione S-transferase omega-1 | HEVININLK |
| P78417 | Glutathione S-transferase omega-1 | MILELFSK |
| P78417 | Glutathione S-transferase omega-1 | GIRHEVININLK |
| P48637 | Glutathione synthetase | AWELYGSPNALVLLIAQEK |
| P48637 | Glutathione synthetase | EGIAQTVFLGLNR |
| P48637 | Glutathione synthetase | AIENELLAR |
| P48637 | Glutathione synthetase | ALAEGVLLR |
| P48637 | Glutathione synthetase | HVLSVLSK |
| P48637 | Glutathione synthetase | ATFAGLYSLDVGEEGDQAIAEALAAPSR |
| P48637 | Glutathione synthetase | IILER |
| P48637 | Glutathione synthetase | GLALGIAK |
| P48637 | Glutathione synthetase | QYSLQNWEAR |
| P48637 | Glutathione synthetase | EGGGNNLYGEEMVQALK |
| P48637 | Glutathione synthetase | SADGSPALK |
| P48637 | Glutathione synthetase | VQQELSRPGMLEMLLPGQPEAVAR |
| P04406 | GlyCeraldehyde-3-phOSphate dehydrogenase | WGDAGAEYVVESTGVFTTMEK |
| P04406 | GlyCeraldehyde-3-phOSphate dehydrogenase | VIHDNFGIVEGLMTTVHAITATQK |
| P04406 | GlyCeraldehyde-3-phOSphate dehydrogenase | IISNASCTTNCLAPLAK |
| P04406 | GlyCeraldehyde-3-phOSphate dehydrogenase | IKWGDAGAEYVVESTGVFTTMEK |
| P04406 | GlyCeraldehyde-3-phOSphate dehydrogenase | LVINGNPITIFQERDPSK |
| P04406 | GlyCeraldehyde-3-phOSphate dehydrogenase | LISWYDNEFGYSNR |
| P04406 | GlyCeraldehyde-3-phOSphate dehydrogenase | VPTANVSVVDLTCR |
| P04406 | GlyCeraldehyde-3-phOSphate dehydrogenase | RVIISAPSADAPMFVMGVNHEK |
| P04406 | GlyCeraldehyde-3-phOSphate dehydrogenase | GILGYTEHQVVSSDFNSDTHSSTFDAGAGIALNDHFVK |
| P04406 | GlyCeraldehyde-3-phOSphate dehydrogenase | VVDLMAHMASKE |
| P04406 | GlyCeraldehyde-3-phOSphate dehydrogenase | VGVNGFGR |
| P04406 | GlyCeraldehyde-3-phOSphate dehydrogenase | GALQNIIPASTGAAK |
| P04406 | GlyCeraldehyde-3-phOSphate dehydrogenase | VDIVAINDPFIDLNYMVYMFQYDSTHGK |
| P04406 | GlyCeraldehyde-3-phOSphate dehydrogenase | LTGMAFRVPTANVSVVDLTCR |
| P04406 | GlyCeraldehyde-3-phOSphate dehydrogenase | VIISAPSADAPMFVMGVNHEK |
| P04406 | GlyCeraldehyde-3-phOSphate dehydrogenase | LVINGNPITIFQER |
| P04406 | GlyCeraldehyde-3-phOSphate dehydrogenase | AGAHLQGGAK |
| P04406 | GlyCeraldehyde-3-phOSphate dehydrogenase | DGRGALQNIIPASTGAAK |
| P04406 | GlyCeraldehyde-3-phOSphate dehydrogenase | AGAHLQGGAKR |
| P04406 | GlyCeraldehyde-3-phOSphate dehydrogenase | VPTANVSVVDLTCRLEKPAK |
| P04406 | GlyCeraldehyde-3-phOSphate dehydrogenase | VKVGVNGFGR |
| P04406 | GlyCeraldehyde-3-phOSphate dehydrogenase | LEKPAKYDDIK |
| P04406 | GlyCeraldehyde-3-phOSphate dehydrogenase | FHGTVKAENGK |
| P04406 | GlyCeraldehyde-3-phOSphate dehydrogenase | QASEGPLK |
| P06737 | GlyCogen phOSphorylase, liver form | GAGTVFDAFPDQVAIQLNDTHPALAIPELMR |
| P02724 | GlyCophorin-A | KSPSDVKPLPSPDTDVPLSSVEIENPETSDQ |
| P30419 | GlyCylpeptide N-tetradeCanoyltransferase 1 | GFDVFNALDLMENK |
| P41250 | GlyCyl-tRNA synthetase | LGDAVEQGVINNTVLGYFIGR |
| P41250 | GlyCyl-tRNA synthetase | SPITGNDLSPPVSFNLMFK |
| P41250 | GlyCyl-tRNA synthetase | TFFSFPAVVAPFK |
| P41250 | GlyCyl-tRNA synthetase | TLYVEEVVPNVIEPSFGLGR |
| Q9HC38 | Glyoxalase domain-Containing protein 4 | ILTPLVSLDTPGK |
| Q9HC38 | Glyoxalase domain-Containing protein 4 | SLNYWCNLLGMK |
| Q9HC38 | Glyoxalase domain-Containing protein 4 | VTLAVSDLQK |
| Q9HC38 | Glyoxalase domain-Containing protein 4 | LGNDFMGITLASSQAVSNAR |
| Q9HC38 | Glyoxalase domain-Containing protein 4 | GGVDHAAAFGR |
| Q9HC38 | Glyoxalase domain-Containing protein 4 | LLDDAMAADKSDEWFAK |
| Q9UBQ7 | Glyoxylate reduCtase/hydroxypyruvate reduCtase | GDVVNQDDLYQALASGK |
| Q9UBQ7 | Glyoxylate reduCtase/hydroxypyruvate reduCtase | IAAAGLDVTSPEPLPTNHPLLTLK |
| Q9UBQ7 | Glyoxylate reduCtase/hydroxypyruvate reduCtase | ILDAAGANLK |
| Q9UBQ7 | Glyoxylate reduCtase/hydroxypyruvate reduCtase | LKPFGVQR |
| Q9P2T1 | GMP reduCtase 2 | VGIGPGSVCTTR |
| Q9NQ84 | G-protein Coupled reCeptor family C group 5 member C | VPSEGAYDIILPR |
| Q9NQ84 | G-protein Coupled reCeptor family C group 5 member C | SSPEQSYQGDMYPTR |
| Q9NQ84 | G-protein Coupled reCeptor family C group 5 member C | AFSMDEPVAAK |
| P30047 | GTP CyClohydrolase 1 feedbaCk regulatory protein | MEVGPTMVGDEQSDPELMQHLGASK |
| P62826 | GTP-binding nuClear protein Ran | KYVATLGVEVHPLVFHTNR |
| P62826 | GTP-binding nuClear protein Ran | FNVWDTAGQEK |
| P62826 | GTP-binding nuClear protein Ran | SNYNFEKPFLWLAR |
| P62826 | GTP-binding nuClear protein Ran | LVLVGDGGTGK |
| P62826 | GTP-binding nuClear protein Ran | RHLTGEFEK |
| Q9BX10 | GTP-binding protein 2 | VCSIQR |
| Q9Y2T3 | Guanine deaminase | IVFLEEASQQEK |
| Q9Y2T3 | Guanine deaminase | ASDSPIDLFYGDFFGDISEAVIQK |
| Q9Y2T3 | Guanine deaminase | FSLSCSETLMGELGNIAK |
| P04899 | Guanine nuCleotide-binding protein G(i) subunit alpha-2 | AVVYSNTIQSIMAIVK |
| P62873 | Guanine nuCleotide-binding protein G(I)/G(S)/G(T) subunit beta-1 | AGVLAGHDNR |
| P62873 | Guanine nuCleotide-binding protein G(I)/G(S)/G(T) subunit beta-1 | VSCLGVTDDGMAVATGSWDSFLK |
| P62879 | Guanine nuCleotide-binding protein G(I)/G(S)/G(T) subunit beta-2 | ACGDSTLTQITAGLDPVGR |
| P62879 | Guanine nuCleotide-binding protein G(I)/G(S)/G(T) subunit beta-2 | VSCLGVTDDGMAVATGSWDSFLK |
| P62879 | Guanine nuCleotide-binding protein G(I)/G(S)/G(T) subunit beta-2 | VHAIPLR |
| Q14344 | Guanine nuCleotide-binding protein subunit alpha-13 | ILLLGAGESGK |
| P63244 | Guanine nuCleotide-binding protein subunit beta-2-like 1 | FSPNSSNPIIVSCGWDK |
| P63244 | Guanine nuCleotide-binding protein subunit beta-2-like 1 | IWDLEGK |
| Q9H0R4 | HaloaCid dehalogenase-like hydrolase domain-Containing protein 2 | DGLALGPGPFVTALEYATDTK |
| Q9H0R4 | HaloaCid dehalogenase-like hydrolase domain-Containing protein 2 | LLLDGAPLIAIHK |
| Q9H0R4 | HaloaCid dehalogenase-like hydrolase domain-Containing protein 2 | ATVVGKPEK |
| P00739 | Haptoglobin-related protein | VGYVSGWGQSDNFK |
| P00739 | Haptoglobin-related protein | YQEDTCYGDAGSAFAVHDLEEDTWYAAGILSFDK |
| P00739 | Haptoglobin-related protein | SPVGVQPILNEHTFCVGMSK |
| P00739 | Haptoglobin-related protein | VTSIQHWVQK |
| P34932 | Heat shoCk 70 kDa protein 4 | GCALQCAILSPAFK |
| P34932 | Heat shoCk 70 kDa protein 4 | VLATAFDTTLGGR |
| P34932 | Heat shoCk 70 kDa protein 4 | NAVEEYVYEMR |
| P34932 | Heat shoCk 70 kDa protein 4 | SNLAYDIVQLPTGLTGIK |
| P34932 | Heat shoCk 70 kDa protein 4 | WNSPAEEGSSDCEVFSK |
| P34932 | Heat shoCk 70 kDa protein 4 | SVMDATQIAGLNCLR |
| P34932 | Heat shoCk 70 kDa protein 4 | STNEAMEWMNNK |
| P34932 | Heat shoCk 70 kDa protein 4 | ELSTTLNADEAVTR |
| P34932 | Heat shoCk 70 kDa protein 4 | AGGIETIANEYSDR |
| P34932 | Heat shoCk 70 kDa protein 4 | LKETAESVLK |
| P34932 | Heat shoCk 70 kDa protein 4 | EFSITDVVPYPISLR |
| P34932 | Heat shoCk 70 kDa protein 4 | EPFTLEAYYSSPQDLPYPDPAIAQFSVQK |
| P34932 | Heat shoCk 70 kDa protein 4 | LMSANASDLPLSIECFMNDVDVSGTMNR |
| P34932 | Heat shoCk 70 kDa protein 4 | NLGQPIK |
| P34932 | Heat shoCk 70 kDa protein 4 | MQVDQEEPHVEEQQQQTPAENKAESEEMETSQAGSK |
| P34932 | Heat shoCk 70 kDa protein 4 | AFSDPFVEAEK |
| P34932 | Heat shoCk 70 kDa protein 4 | NKEDQYDHLDAADMTK |
| P11142 | Heat shoCk Cognate 71 kDa protein | NSLESYAFNMK |
| P11142 | Heat shoCk Cognate 71 kDa protein | RFDDAVVQSDMK |
| P11142 | Heat shoCk Cognate 71 kDa protein | NQVAMNPTNTVFDAK |
| P11142 | Heat shoCk Cognate 71 kDa protein | DAGTIAGLNVLR |
| P11142 | Heat shoCk Cognate 71 kDa protein | CNEIINWLDK |
| P11142 | Heat shoCk Cognate 71 kDa protein | SFYPEEVSSMVLTK |
| P11142 | Heat shoCk Cognate 71 kDa protein | SQIHDIVLVGGSTR |
| P11142 | Heat shoCk Cognate 71 kDa protein | NQTAEKEEFEHQQK |
| P11142 | Heat shoCk Cognate 71 kDa protein | LLQDFFNGK |
| P11142 | Heat shoCk Cognate 71 kDa protein | GPAVGIDLGTTYSCVGVFQHGK |
| P11142 | Heat shoCk Cognate 71 kDa protein | ARFEELNADLFR |
| P11142 | Heat shoCk Cognate 71 kDa protein | EIAEAYLGK |
| P11142 | Heat shoCk Cognate 71 kDa protein | IINEPTAAAIAYGLDK |
| P11142 | Heat shoCk Cognate 71 kDa protein | IINEPTAAAIAYGLDKK |
| P11142 | Heat shoCk Cognate 71 kDa protein | NQVAMNPTNTVFDAKR |
| P11142 | Heat shoCk Cognate 71 kDa protein | QTQTFTTYSDNQPGVLIQVYEGER |
| P11142 | Heat shoCk Cognate 71 kDa protein | LSKEDIER |
| P11142 | Heat shoCk Cognate 71 kDa protein | SINPDEAVAYGAAVQAAILSGDK |
| P11142 | Heat shoCk Cognate 71 kDa protein | MVNHFIAEFK |
| P11142 | Heat shoCk Cognate 71 kDa protein | FDDAVVQSDMK |
| P11142 | Heat shoCk Cognate 71 kDa protein | MKEIAEAYLGK |
| P11142 | Heat shoCk Cognate 71 kDa protein | FEELNADLFR |
| P11142 | Heat shoCk Cognate 71 kDa protein | VQVEYKGETK |
| P11142 | Heat shoCk Cognate 71 kDa protein | STAGDTHLGGEDFDNR |
| O75506 | Heat shoCk faCtor-binding protein 1 | TVQDLTSVVQTLLQQMQDK |
| Q92598 | Heat shoCk protein 105 kDa | VLGTAFDPFLGGK |
| P07900 | Heat shoCk protein HSP 90-alpha | NPDDITNEEYGEFYK |
| P07900 | Heat shoCk protein HSP 90-alpha | HLEINPDHSIIETLR |
| P07900 | Heat shoCk protein HSP 90-alpha | LVTSPCCIVTSTYGWTANMER |
| P07900 | Heat shoCk protein HSP 90-alpha | YYTSASGDEMVSLK |
| P07900 | Heat shoCk protein HSP 90-alpha | DQVANSAFVER |
| P07900 | Heat shoCk protein HSP 90-alpha | TDTGEPMGR |
| P07900 | Heat shoCk protein HSP 90-alpha | TKPIWTRNPDDITNEEYGEFYK |
| P07900 | Heat shoCk protein HSP 90-alpha | ELISNSSDALDK |
| P07900 | Heat shoCk protein HSP 90-alpha | DLVILLYETALLSSGFSLEDPQTHANR |
| P07900 | Heat shoCk protein HSP 90-alpha | RVFIMDNCEELIPEYLNFIR |
| P07900 | Heat shoCk protein HSP 90-alpha | HIYYITGETK |
| P07900 | Heat shoCk protein HSP 90-alpha | LGIHEDSQNR |
| P07900 | Heat shoCk protein HSP 90-alpha | DNSTMGYMAAK |
| P08238 | Heat shoCk protein HSP 90-beta | YHTSQSGDEMTSLSEYVSR |
| P08238 | Heat shoCk protein HSP 90-beta | LVSSPCCIVTSTYGWTANMER |
| P08238 | Heat shoCk protein HSP 90-beta | DLVVLLFETALLSSGFSLEDPQTHSNR |
| P08238 | Heat shoCk protein HSP 90-beta | HLEINPDHPIVETLR |
| P08238 | Heat shoCk protein HSP 90-beta | RGFEVVYMTEPIDEYCVQQLK |
| P08238 | Heat shoCk protein HSP 90-beta | FYEAFSK |
| P08238 | Heat shoCk protein HSP 90-beta | ALLFIPR |
| P08238 | Heat shoCk protein HSP 90-beta | EQVANSAFVER |
| P08238 | Heat shoCk protein HSP 90-beta | NPDDITQEEYGEFYK |
| P08238 | Heat shoCk protein HSP 90-beta | ELISNASDALDK |
| P08238 | Heat shoCk protein HSP 90-beta | ADHGEPIGR |
| P08238 | Heat shoCk protein HSP 90-beta | DNSTMGYMMAK |
| Q9UK76 | HematologiCal and neurologiCal expressed 1 protein | SSGGREDLESSGLQR |
| Q9UK76 | HematologiCal and neurologiCal expressed 1 protein | VLRPPGGGSNFSLGFDEPTEQPVRK |
| Q9H910 | HematologiCal and neurologiCal expressed 1-like protein | MASNIFGPTEEPQNIPK |
| Q9H910 | HematologiCal and neurologiCal expressed 1-like protein | GSGIFDESTPVQTR |
| Q9Y5Z4 | Heme-binding protein 2 | VYYTAGYNSPVK |
| Q9Y5Z4 | Heme-binding protein 2 | NQEQLLTLASILR |
| Q9Y5Z4 | Heme-binding protein 2 | APEDAGPQPGSYEIR |
| Q9Y5Z4 | Heme-binding protein 2 | SFDGFSSAQK |
| Q9Y5Z4 | Heme-binding protein 2 | LNSYIQGK |
| P69905 | Hemoglobin subunit alpha | KVADALTNAVAHVDDMPNALSALSDLHAHK |
| P69905 | Hemoglobin subunit alpha | FLASVSTVLTSK |
| P69905 | Hemoglobin subunit alpha | VGAHAGEYGAEALER |
| P69905 | Hemoglobin subunit alpha | LLSHCLLVTLAAHLPAEFTPAVHASLDK |
| P69905 | Hemoglobin subunit alpha | VADALTNAVAHVDDMPNALSALSDLHAHK |
| P69905 | Hemoglobin subunit alpha | VLSPADKTNVK |
| P69905 | Hemoglobin subunit alpha | TYFPHFDLSHGSAQVK |
| P69905 | Hemoglobin subunit alpha | LRVDPVNFK |
| P69905 | Hemoglobin subunit alpha | LLSHCLLVTLAAHLPAEFTPAVHASLDKFLASVSTVLTSK |
| P69905 | Hemoglobin subunit alpha | MFLSFPTTK |
| P69905 | Hemoglobin subunit alpha | FLASVSTVLTSKYR |
| P69905 | Hemoglobin subunit alpha | VADALTNAVAHVDDMPNALSALSDLHAHKLR |
| P68871 | Hemoglobin subunit beta | VNVDEVGGEALGR |
| P68871 | Hemoglobin subunit beta | FFESFGDLSTPDAVMGNPK |
| P68871 | Hemoglobin subunit beta | GTFATLSELHCDK |
| P68871 | Hemoglobin subunit beta | SAVTALWGK |
| P68871 | Hemoglobin subunit beta | LLGNVLVCVLAHHFGK |
| P68871 | Hemoglobin subunit beta | EFTPPVQAAYQK |
| P68871 | Hemoglobin subunit beta | GTFATLSELHCDKLHVDPENFR |
| P68871 | Hemoglobin subunit beta | VHLTPEEKSAVTALWGK |
| P02042 | Hemoglobin subunit delta | FFESFGDLSSPDAVMGNPK |
| P02042 | Hemoglobin subunit delta | LLGNVLVCVLAR |
| P02042 | Hemoglobin subunit delta | VNVDAVGGEALGR |
| P02042 | Hemoglobin subunit delta | GTFSQLSELHCDK |
| P02042 | Hemoglobin subunit delta | EFTPQMQAAYQK |
| P02042 | Hemoglobin subunit delta | TAVNALWGK |
| P02042 | Hemoglobin subunit delta | GTFSQLSELHCDKLHVDPENFR |
| P02100 | Hemoglobin subunit epsilon | LSELHCDK |
| P69891 | Hemoglobin subunit gamma-1 | FFDSFGNLSSASAIMGNPK |
| P69891 | Hemoglobin subunit gamma-1 | VNVEDAGGETLGR |
| P69891 | Hemoglobin subunit gamma-1 | EFTPEVQASWQK |
| P69891 | Hemoglobin subunit gamma-1 | MVTAVASALSSR |
| P69891 | Hemoglobin subunit gamma-1 | ATITSLWGK |
| P69891 | Hemoglobin subunit gamma-1 | VLTSLGDAIK |
| P05546 | Heparin CofaCtor 2 | GGETAQSADPQWEQLNNK |
| P05546 | Heparin CofaCtor 2 | GNFLAANDQELDCDILQLEYVGGISMLIVVPHK |
| P05546 | Heparin CofaCtor 2 | DALENIDPATQMMILNCIYFK |
| P05546 | Heparin CofaCtor 2 | SVNDLYIQK |
| P05546 | Heparin CofaCtor 2 | NYNLVESLK |
| P05546 | Heparin CofaCtor 2 | IAIDLFK |
| P51858 | Hepatoma-derived growth faCtor | CGDLVFAK |
| P51858 | Hepatoma-derived growth faCtor | YQVFFFGTHETAFLGPK |
| P51858 | Hepatoma-derived growth faCtor | EAATLEVERPLPMEVEK |
| P51858 | Hepatoma-derived growth faCtor | EAENPEGEEKEAATLEVERPLPMEVEK |
| P51858 | Hepatoma-derived growth faCtor | EAENPEGEEK |
| P51858 | Hepatoma-derived growth faCtor | KGFSEGLWEIENNPTVK |
| P51858 | Hepatoma-derived growth faCtor | AGDLLEDSPK |
| P51858 | Hepatoma-derived growth faCtor | GFSEGLWEIENNPTVK |
| P51858 | Hepatoma-derived growth faCtor | IDEMPEAAVK |
| Q99729 | Heterogeneous nuClear ribonuCleoprotein A/B | EVYQQQQYGSGGR |
| O60812 | Heterogeneous nuClear ribonuCleoprotein C-like 1 | VFIGNLNTLVVK |
| O14979 | Heterogeneous nuClear ribonuCleoprotein D-like | FGEVVDCTIKTDPVTGR |
| P52597 | Heterogeneous nuClear ribonuCleoprotein F | ITGEAFVQFASQELAEK |
| P38159 | Heterogeneous nuClear ribonuCleoprotein G | ALEAVFGK |
| P38159 | Heterogeneous nuClear ribonuCleoprotein G | LFIGGLNTETNEK |
| P61978 | Heterogeneous nuClear ribonuCleoprotein K | IILDLISESPIK |
| P61978 | Heterogeneous nuClear ribonuCleoprotein K | GSYGDLGGPIITTQVTIPK |
| P61978 | Heterogeneous nuClear ribonuCleoprotein K | GSDFDCELR |
| P61978 | Heterogeneous nuClear ribonuCleoprotein K | LLIHQSLAGGIIGVK |
| P61978 | Heterogeneous nuClear ribonuCleoprotein K | DLAGSIIGK |
| P61978 | Heterogeneous nuClear ribonuCleoprotein K | IIPTLEEGLQLPSPTATSQLPLESDAVECLNYQHYK |
| P61978 | Heterogeneous nuClear ribonuCleoprotein K | NTDEMVELR |
| P61978 | Heterogeneous nuClear ribonuCleoprotein K | IDEPLEGSEDRIITITGTQDQIQNAQYLLQNSVK |
| P61978 | Heterogeneous nuClear ribonuCleoprotein K | TDYNASVSVPDSSGPER |
| P61978 | Heterogeneous nuClear ribonuCleoprotein K | VVLIGGKPDR |
| P52272 | Heterogeneous nuClear ribonuCleoprotein M | AFITNIPFDVK |
| O60506 | Heterogeneous nuClear ribonuCleoprotein Q | EFNEDGALAVLQQFK |
| O60506 | Heterogeneous nuClear ribonuCleoprotein Q | DLFEDELVPLFEK |
| O60506 | Heterogeneous nuClear ribonuCleoprotein Q | NLANTVTEEILEK |
| O60506 | Heterogeneous nuClear ribonuCleoprotein Q | VADSSKGPDEAK |
| O60506 | Heterogeneous nuClear ribonuCleoprotein Q | LMMDPLTGLNR |
| O60506 | Heterogeneous nuClear ribonuCleoprotein Q | TGYTLDVTTGQR |
| O43390 | Heterogeneous nuClear ribonuCleoprotein R | DLYEDELVPLFEK |
| Q00839 | Heterogeneous nuClear ribonuCleoprotein U | LQAALDDEEAGGRPAMEPGNGSLDLGGDSAGR |
| Q00839 | Heterogeneous nuClear ribonuCleoprotein U | LSASSLTMESFAFLWAGGR |
| Q00839 | Heterogeneous nuClear ribonuCleoprotein U | YNILGTNTIMDK |
| Q00839 | Heterogeneous nuClear ribonuCleoprotein U | NFILDQTNVSAAAQR |
| P07910 | Heterogeneous nuClear ribonuCleoproteins C1/C2 | MIAGQVLDINLAAEPK |
| P07910 | Heterogeneous nuClear ribonuCleoproteins C1/C2 | VFIGNLNTLVVK |
| P07910 | Heterogeneous nuClear ribonuCleoproteins C1/C2 | VPPPPPIAR |
| P07910 | Heterogeneous nuClear ribonuCleoproteins C1/C2 | NDKSEEEQSSSSVK |
| P82970 | High mobility group nuCleOSome-binding domain-Containing protein 5 | LSAMLVPVTPEVKPK |
| P09429 | High mobility group protein B1 | IKGEHPGLSIGDVAK |
| P09429 | High mobility group protein B1 | LGEMWNNTAADDKQPYEK |
| P09429 | High mobility group protein B1 | KHPDASVNFSEFSK |
| P09429 | High mobility group protein B1 | FKDPNAPK |
| P09429 | High mobility group protein B1 | TYIPPKGETK |
| P26583 | High mobility group protein B2 | KLGEMWSEQSAK |
| P26583 | High mobility group protein B2 | IKSEHPGLSIGDTAK |
| P49773 | Histidine triad nuCleotide-binding protein 1 | AQVARPGGDTIFGK |
| P04196 | Histidine-riCh glyCoprotein | DSPVLIDFFEDTER |
| P04196 | Histidine-riCh glyCoprotein | ADLFYDVEALDLESPK |
| P04196 | Histidine-riCh glyCoprotein | YKEENDDFASFR |
| P04196 | Histidine-riCh glyCoprotein | SGFPQVSMFFTHTFPK |
| P04196 | Histidine-riCh glyCoprotein | ALDLINK |
| P04196 | Histidine-riCh glyCoprotein | RPSEIVIGQCK |
| P04196 | Histidine-riCh glyCoprotein | VRGGEGTGYFVDFSVR |
| P04196 | Histidine-riCh glyCoprotein | DGYLFQLLR |
| P04196 | Histidine-riCh glyCoprotein | QIGSVYR |
| P04196 | Histidine-riCh glyCoprotein | KGEVLPLPEANFPSFPLPHHK |
| P04196 | Histidine-riCh glyCoprotein | ALDLINKR |
| P04196 | Histidine-riCh glyCoprotein | HSHESQDLR |
| P04196 | Histidine-riCh glyCoprotein | GGEGTGYFVDFSVR |
| P04196 | Histidine-riCh glyCoprotein | HPLKPDNQPFPQSVSESCPGK |
| P04196 | Histidine-riCh glyCoprotein | YWNDCEPPDSR |
| O14607 | Histone demethylase UTY | ALSAYQR |
| P22492 | Histone H1t | ALAAAGYDVEK |
| Q96KK5 | Histone H2A type 1-H | VTIAQGGVLPNIQAVLLPK |
| Q96KK5 | Histone H2A type 1-H | AGLQFPVGR |
| O60814 | Histone H2B type 1-K | AMGIMNSFVNDIFER |
| O60814 | Histone H2B type 1-K | LAHYNKR |
| O60814 | Histone H2B type 1-K | LLLPGELAK |
| P68431 | Histone H3.1 | FQSSAVMALQEACEAYLVGLFEDTNLCAIHAK |
| Q71DI3 | Histone H3.2 | FQSSAVMALQEASEAYLVGLFEDTNLCAIHAK |
| P84243 | Histone H3.3 | FQSAAIGALQEASEAYLVGLFEDTNLCAIHAK |
| Q6NXT2 | Histone H3.3C | STELLIR |
| P62805 | Histone H4 | TVTAMDVVYALK |
| P62805 | Histone H4 | VFLENVIR |
| P62805 | Histone H4 | VFLENVIRDAVTYTEHAK |
| P62805 | Histone H4 | DAVTYTEHAK |
| Q09028 | Histone-binding protein RBBP4 | TPSSDVLVFDYTK |
| Q09028 | Histone-binding protein RBBP4 | INHEGEVNR |
| P28067 | HLA Class II histoCompatibility antigen, DM alpha Chain | VPRLPEFADWAQEQGDAPAILFDK |
| P28067 | HLA Class II histoCompatibility antigen, DM alpha Chain | LPEFADWAQEQGDAPAILFDK |
| P28068 | HLA Class II histoCompatibility antigen, DM beta Chain | TRPPSVQVAK |
| P01903 | HLA Class II histoCompatibility antigen, DR alpha Chain | ANLEIMTK |
| P01911 | HLA Class II histoCompatibility antigen, DRB1-15 beta Chain | SGEVYTCQVEHPSVTSPLTVEWR |
| Q93099 | Homogentisate 1,2-dioxygenase | MLVQPNEICVIQR |
| Q16543 | Hsp90 Co-Chaperone CdC37 | LGPGGLDPVEVYESLPEELQK |
| Q96S86 | Hyaluronan and proteoglyCan link protein 3 | EACQEDDATIAK |
| P00492 | Hypoxanthine-guanine phOSphoribOSyltransferase | NVLIVEDIIDTGK |
| P00492 | Hypoxanthine-guanine phOSphoribOSyltransferase | FFADLLDYIK |
| P00492 | Hypoxanthine-guanine phOSphoribOSyltransferase | TMQTLLSLVR |
| P00492 | Hypoxanthine-guanine phOSphoribOSyltransferase | VIGGDDLSTLTGK |
| P00492 | Hypoxanthine-guanine phOSphoribOSyltransferase | SYCNDQSTGDIK |
| P01876 | Ig alpha-1 Chain C region | DASGVTFTWTPSSGK |
| P01876 | Ig alpha-1 Chain C region | DLCGCYSVSSVLPGCAEPWNHGK |
| P01876 | Ig alpha-1 Chain C region | TFTCTAAYPESKTPLTATLSK |
| P01876 | Ig alpha-1 Chain C region | VFPLSLCSTQPDGNVVIACLVQGFFPQEPLSVTWSESGQGVTAR |
| P01876 | Ig alpha-1 Chain C region | SAVQGPPERDLCGCYSVSSVLPGCAEPWNHGK |
| P01876 | Ig alpha-1 Chain C region | TFTCTAAYPESK |
| P01876 | Ig alpha-1 Chain C region | NFPPSQDASGDLYTTSSQLTLPATQCLAGK |
| P01876 | Ig alpha-1 Chain C region | DASGVTFTWTPSSGKSAVQGPPER |
| P01876 | Ig alpha-1 Chain C region | TPLTATLSK |
| P01877 | Ig alpha-2 Chain C region | HYTNPSQDVTVPCPVPPPPPCCHPR |
| P01877 | Ig alpha-2 Chain C region | DASGATFTWTPSSGK |
| P01877 | Ig alpha-2 Chain C region | DLCGCYSVSSVLPGCAQPWNHGETFTCTAAHPELK |
| P01877 | Ig alpha-2 Chain C region | NFPPSQDASGDLYTTSSQLTLPATQCPDGK |
| P01877 | Ig alpha-2 Chain C region | SAVQGPPERDLCGCYSVSSVLPGCAQPWNHGETFTCTAAHPELK |
| P01857 | Ig gamma-1 Chain C region | STSGGTAALGCLVK |
| P01857 | Ig gamma-1 Chain C region | FNWYVDGVEVHNAK |
| P01857 | Ig gamma-1 Chain C region | TTPPVLDSDGSFFLYSK |
| P01857 | Ig gamma-1 Chain C region | TPEVTCVVVDVSHEDPEVK |
| P01859 | Ig gamma-2 Chain C region | VVSVLTVVHQDWLNGK |
| P01859 | Ig gamma-2 Chain C region | TTPPmLDSDGSFFLYSK |
| P01859 | Ig gamma-2 Chain C region | STSESTAALGCLVK |
| P01860 | Ig gamma-3 Chain C region | TPEVTCVVVDVSHEDPEVQFK |
| P01860 | Ig gamma-3 Chain C region | SCDTPPPCPR |
| P01861 | Ig gamma-4 Chain C region | TTPPVLDSDGSFFLYSR |
| P01861 | Ig gamma-4 Chain C region | YGPPCPSCPAPEFLGGPSVFLFPPKPK |
| P01743 | Ig heavy Chain V-I region HG3 | SEDTAVYYCAR |
| P23083 | Ig heavy Chain V-I region V35 | DTSISTAYMELSR |
| P23083 | Ig heavy Chain V-I region V35 | QAPGQGLEWMGR |
| P01825 | Ig heavy Chain V-II region NEWM | LSSVTAADTAVYYCAR |
| P01814 | Ig heavy Chain V-II region OU | ALEWLAR |
| P01766 | Ig heavy Chain V-III region BRO | EVQLVESGGGLVQPGGSLR |
| P01766 | Ig heavy Chain V-III region BRO | AEDTAVYYCAR |
| P01767 | Ig heavy Chain V-III region BUT | bTVYLQMbSLR |
| P01767 | Ig heavy Chain V-III region BUT | EVQLVETGGGLIQPGGSLR |
| P01767 | Ig heavy Chain V-III region BUT | bTVYLQMbSLRAEDTAVYYCAR |
| P01768 | Ig heavy Chain V-III region CAM | bTLYLQMNSLRAEbTAVYYCAR |
| P01768 | Ig heavy Chain V-III region CAM | NTLYLQMNSLR |
| P01768 | Ig heavy Chain V-III region CAM | YYAbSVK |
| P01782 | Ig heavy Chain V-III region DOB | GRFAISR |
| P01781 | Ig heavy Chain V-III region GAL | NSLYLQMNSLR |
| P01781 | Ig heavy Chain V-III region GAL | GLEWVANIK |
| P01781 | Ig heavy Chain V-III region GAL | VEDTALYYCAR |
| P01781 | Ig heavy Chain V-III region GAL | FTISRDNAK |
| P01771 | Ig heavy Chain V-III region HIL | TEDTAVYYCAR |
| P01780 | Ig heavy Chain V-III region JON | DVQLVESGGGLVKPGGSLR |
| P01777 | Ig heavy Chain V-III region TEI | EVQLVESGGGLVQPGGSLR |
| P01777 | Ig heavy Chain V-III region TEI | NDSKNTLYLQMLSLEPzbTAVYYCAR |
| P01765 | Ig heavy Chain V-III region TIL | EVQLLESGGGLVQPGGSLR |
| P01765 | Ig heavy Chain V-III region TIL | AEDTAVYYCAK |
| P01779 | Ig heavy Chain V-III region TUR | LSCAASGFTFSR |
| P01779 | Ig heavy Chain V-III region TUR | EVQLLESGGGLVQPGGSLR |
| P01764 | Ig heavy Chain V-III region VH26 | LSCAASGFTFSSYAMSWVR |
| P01764 | Ig heavy Chain V-III region VH26 | NTLYLQMNSLR |
| P01764 | Ig heavy Chain V-III region VH26 | AEDTAVYYCAK |
| P01776 | Ig heavy Chain V-III region WAS | LEAzbTAVYYCAR |
| P01834 | Ig kappa Chain C region | VDNALQSGNSQESVTEQDSK |
| P01834 | Ig kappa Chain C region | VDNALQSGNSQESVTEQDSKDSTYSLSSTLTLSK |
| P01834 | Ig kappa Chain C region | VYACEVTHQGLSSPVTK |
| P01834 | Ig kappa Chain C region | VQWKVDNALQSGNSQESVTEQDSK |
| P01834 | Ig kappa Chain C region | SGTASVVCLLNNFYPR |
| P01834 | Ig kappa Chain C region | DSTYSLSSTLTLSK |
| P01834 | Ig kappa Chain C region | TVAAPSVFIFPPSDEQLK |
| P01834 | Ig kappa Chain C region | DSTYSLSSTLTLSKADYEK |
| P01834 | Ig kappa Chain C region | SFNRGEC |
| P01834 | Ig kappa Chain C region | HKVYACEVTHQGLSSPVTK |
| P01593 | Ig kappa Chain V-I region AG | DIQMTQSPSSLSASVGDR |
| P01593 | Ig kappa Chain V-I region AG | ILIYDASNLETGVPSR |
| P01596 | Ig kappa Chain V-I region CAR | DIQMTQSPSTLSASVGDR |
| P01596 | Ig kappa Chain V-I region CAR | VLIYK |
| P01597 | Ig kappa Chain V-I region DEE | bIzMTQSPSSLSASVGDRVTITCR |
| P01597 | Ig kappa Chain V-I region DEE | DIQMTQSPSSLSASVGDR |
| P01598 | Ig kappa Chain V-I region EU | DIQMTQSPSTLSASVGDRVTITCR |
| P01598 | Ig kappa Chain V-I region EU | DIQMTQSPSTLSASVGDR |
| P01598 | Ig kappa Chain V-I region EU | ASSLESGVPSR |
| P01602 | Ig kappa Chain V-I region HK102 (Fragment) | LLIYDASSLESGVPSR |
| P01605 | Ig kappa Chain V-I region Lay | LLIYGASTR |
| P01611 | Ig kappa Chain V-I region Wes | DIQMTQSPSSVSASVGDR |
| P06310 | Ig kappa Chain V-II region RPMI 6410 | SSQSLVYSDGNTYLNWFQQRPGQSPR |
| P01617 | Ig kappa Chain V-II region TEW | DIVMTQSPLSLPVTPGEPASISCR |
| P01617 | Ig kappa Chain V-II region TEW | FSGSGSGTDFTLK |
| P01617 | Ig kappa Chain V-II region TEW | ASGVPDRFSGSGSGTDFTLK |
| P01619 | Ig kappa Chain V-III region B6 | FSGSGSGADFTLTISR |
| P01619 | Ig kappa Chain V-III region B6 | ATGIPDRFSGSGSGADFTLTISR |
| P04207 | Ig kappa Chain V-III region CLL | FSGSGSGTEFTLTISR |
| P18135 | Ig kappa Chain V-III region HAH | ASQSVSSSYLAWYQQKPGQAPR |
| P01621 | Ig kappa Chain V-III region NG9 (Fragment) | ASQSVSSSYLAWYQQKPGQAPR |
| P01621 | Ig kappa Chain V-III region NG9 (Fragment) | LLIYGATSR |
| P01620 | Ig kappa Chain V-III region SIE | EIVLTQSPGTLSLSPGER |
| P01620 | Ig kappa Chain V-III region SIE | FSGSGSGTDFTLTISR |
| P01620 | Ig kappa Chain V-III region SIE | ATGIPDRFSGSGSGTDFTLTISR |
| P01620 | Ig kappa Chain V-III region SIE | LLIYGASSR |
| P01620 | Ig kappa Chain V-III region SIE | ASQSVSNSYLAWYQQKPGQAPR |
| P04433 | Ig kappa Chain V-III region VG (Fragment) | ASQSVSSYLAWYQQKPGQAPR |
| P04433 | Ig kappa Chain V-III region VG (Fragment) | LLIYDASNR |
| P06312 | Ig kappa Chain V-IV region (Fragment) | SSQSVLYSSNNK |
| P01625 | Ig kappa Chain V-IV region Len | DIVMTQSPDSLAVSLGER |
| P83593 | Ig kappa Chain V-IV region STH (Fragment) | SSQSVLYSSNNK |
| P04211 | Ig lambda Chain V region 4A | FSGSLLGGK |
| P04208 | Ig lambda Chain V-I region WAH | SGTSASLAISGLR |
| P04208 | Ig lambda Chain V-I region WAH | IILYK |
| P80748 | Ig lambda Chain V-III region LOI | FSGSNSGNTATLTISR |
| P80748 | Ig lambda Chain V-III region LOI | LTVLSQPK |
| P01714 | Ig lambda Chain V-III region SH | SELTQDPAVSVALGQTVR |
| P01714 | Ig lambda Chain V-III region SH | ITCQGDSLR |
| P01715 | Ig lambda Chain V-IV region Bau | RPSGIPER |
| P01717 | Ig lambda Chain V-IV region Hil | SYELTQPPSVSVSPGQTAR |
| P0CG04 | Ig lambda-1 Chain C regions | ANPTVTLFPPSSEELQANK |
| P0CG04 | Ig lambda-1 Chain C regions | AGVETTKPSK |
| P0CG04 | Ig lambda-1 Chain C regions | AGVETTKPSKQSNNK |
| P0CG05 | Ig lambda-2 Chain C regions | ATLVCLISDFYPGAVTVAWKADSSPVK |
| P0CG05 | Ig lambda-2 Chain C regions | AGVETTTPSK |
| P0CG05 | Ig lambda-2 Chain C regions | AAPSVTLFPPSSEELQANK |
| P0CG05 | Ig lambda-2 Chain C regions | AGVETTTPSKQSNNK |
| A0M8Q6 | Ig lambda-7 Chain C region | VGVETTKPSK |
| A0M8Q6 | Ig lambda-7 Chain C region | VTHEGSTVEK |
| P01871 | Ig mu Chain C region | YAATSQVLLPSK |
| P01871 | Ig mu Chain C region | QVGSGVTTDQVQAEAK |
| P01871 | Ig mu Chain C region | DVMQGTDEHVVCK |
| P01871 | Ig mu Chain C region | NVPLPVIAELPPK |
| P01871 | Ig mu Chain C region | STGKPTLYNVSLVMSDTAGTCY |
| P01871 | Ig mu Chain C region | LICQATGFSPR |
| P01871 | Ig mu Chain C region | SKLICQATGFSPR |
| P01871 | Ig mu Chain C region | ESATITCLVTGFSPADVFVQWMQR |
| P01871 | Ig mu Chain C region | VFAIPPSFASIFLTK |
| P01871 | Ig mu Chain C region | QIQVSWLR |
| P01871 | Ig mu Chain C region | FTCTVTHTDLPSPLK |
| P01871 | Ig mu Chain C region | YVTSAPmPEPQAPGR |
| P01871 | Ig mu Chain C region | GVALHRPDVYLLPPAR |
| P01871 | Ig mu Chain C region | LTCLVTDLTTYDSVTISWTR |
| P01871 | Ig mu Chain C region | EGKQVGSGVTTDQVQAEAK |
| P01871 | Ig mu Chain C region | QVGSGVTTDQVQAEAKESGPTTYK |
| P01871 | Ig mu Chain C region | YAATSQVLLPSKDVMQGTDEHVVCK |
| P01871 | Ig mu Chain C region | GGKYAATSQVLLPSK |
| P01871 | Ig mu Chain C region | ESDWLGQSMFTCR |
| P01871 | Ig mu Chain C region | ESGPTTYKVTSTLTIK |
| P01871 | Ig mu Chain C region | VTSTLTIK |
| P01871 | Ig mu Chain C region | DGFFGNPR |
| P01871 | Ig mu Chain C region | VSVFVPPR |
| P01871 | Ig mu Chain C region | ESGPTTYK |
| P04220 | Ig mu heavy Chain disease protein | YFAHSILTVSEEEWNTGETYTCVVAHEALPNR |
| P04220 | Ig mu heavy Chain disease protein | YFAHSILTVSEEEWNTGETYTCVVAHEALPNRVTER |
| Q9Y6R7 | IgGFC-binding protein | LRVPAAYAASLCGLCGNYNQDPADDLK |
| Q9Y6R7 | IgGFC-binding protein | AGCVAESTAVCR |
| Q9Y6R7 | IgGFC-binding protein | ALASYVAACQAAGVVIEDWR |
| Q9Y6R7 | IgGFC-binding protein | GATTSPGVYELSSR |
| Q9Y6R7 | IgGFC-binding protein | LRVPAAYAGSLCGLCGNYNQDPADDLK |
| Q9Y6R7 | IgGFC-binding protein | SLAAYTAACQAAGVAVKPWR |
| Q9Y6R7 | IgGFC-binding protein | KFDFQGTCNYVLATTGCPGVSTQGLTPFTVTTK |
| Q9Y6R7 | IgGFC-binding protein | GSQAVSYTR |
| Q9Y6R7 | IgGFC-binding protein | NQNRGNPAVSYVR |
| Q9Y6R7 | IgGFC-binding protein | ISVTQGASK |
| Q9Y6R7 | IgGFC-binding protein | VNGVLTALPVSVADGR |
| Q9Y6R7 | IgGFC-binding protein | VRVNGVLTALPVSVADGR |
| Q9Y6R7 | IgGFC-binding protein | ISVAQGASK |
| Q9Y6R7 | IgGFC-binding protein | NAAGDLQR |
| Q9Y6R7 | IgGFC-binding protein | TPDGSLLVR |
| Q9Y6R7 | IgGFC-binding protein | FQDQVCGLCGNYNGDPADDFLTPDGALAPDAVEFASSWK |
| P01591 | Immunoglobulin J Chain | KCDPTEVELDNQIVTATQSNICDEDSATETCYTYDR |
| P01591 | Immunoglobulin J Chain | SSEDPNEDIVER |
| P01591 | Immunoglobulin J Chain | FVYHLSDLCK |
| P01591 | Immunoglobulin J Chain | CYTAVVPLVYGGETK |
| P01591 | Immunoglobulin J Chain | IIRSSEDPNEDIVER |
| P01591 | Immunoglobulin J Chain | IVLVDNK |
| P01591 | Immunoglobulin J Chain | MVETALTPDACYPD |
| P01591 | Immunoglobulin J Chain | CDPTEVELDNQIVTATQSNICDEDSATETCYTYDRNK |
| P01591 | Immunoglobulin J Chain | CDPTEVELDNQIVTATQSNICDEDSATETCYTYDR |
| P15814 | Immunoglobulin lambda-like polypeptide 1 | YAASSYLSLTPEQWR |
| B9A064 | Immunoglobulin lambda-like polypeptide 5 | ATLVCLISDFYPGAVTVAWKADGSPVK |
| B9A064 | Immunoglobulin lambda-like polypeptide 5 | ANPTVTLFPPSSEELQANK |
| B9A064 | Immunoglobulin lambda-like polypeptide 5 | VTVLGQPK |
| B9A064 | Immunoglobulin lambda-like polypeptide 5 | AGVETTKPSK |
| Q14974 | Importin subunit beta-1 | VLANPGNSQVAR |
| Q14974 | Importin subunit beta-1 | AAVENLPTFLVELSR |
| Q13308 | InaCtive tyrOSine-protein kinase 7 | VVLAPQDVVVAR |
| Q15181 | InorganiC pyrophOSphatase | VIAINVDDPDAANYNDINDVK |
| Q15181 | InorganiC pyrophOSphatase | DVFHMVVEVPR |
| Q15181 | InorganiC pyrophOSphatase | AAPFSLEYR |
| Q15181 | InorganiC pyrophOSphatase | DKDFAIDIIK |
| Q15181 | InorganiC pyrophOSphatase | GYIWNYGAIPQTWEDPGHNDK |
| Q15181 | InorganiC pyrophOSphatase | GISCMNTTLSESPFKCDPDAAR |
| Q15181 | InorganiC pyrophOSphatase | VCARGEIIGVK |
| Q15181 | InorganiC pyrophOSphatase | GISCMNTTLSESPFK |
| Q15181 | InorganiC pyrophOSphatase | HTGCCGDNDPIDVCEIGSK |
| Q15181 | InorganiC pyrophOSphatase | GQYISPFHDIPIYADK |
| Q15181 | InorganiC pyrophOSphatase | VIAINVDDPDAANYNDINDVKR |
| P29218 | InOSitol monophOSphatase 1 | SILTDNPTWIIDPIDGTTNFVHR |
| Q9NPH2 | InOSitol-3-phOSphate synthase 1 | SVLVDFLIGSGLK |
| Q9NPH2 | InOSitol-3-phOSphate synthase 1 | SNVVDDMVQSNPVLYTPGEEPDHCVVIK |
| P18065 | Insulin-like growth faCtor-binding protein 2 | LEGEACGVYTPR |
| P18065 | Insulin-like growth faCtor-binding protein 2 | TPCQQELDQVLER |
| P18065 | Insulin-like growth faCtor-binding protein 2 | MPCAELVREPGCGCCSVCAR |
| Q16270 | Insulin-like growth faCtor-binding protein 7 | AGAAAGGPGVSGVCVCK |
| Q16270 | Insulin-like growth faCtor-binding protein 7 | SRYPVCGSDGTTYPSGCQLR |
| Q16270 | Insulin-like growth faCtor-binding protein 7 | DACGCCPmCAR |
| Q16270 | Insulin-like growth faCtor-binding protein 7 | GEGEPCGGGGAGR |
| P35858 | Insulin-like growth faCtor-binding protein Complex aCid labile subunit | NLIAAVAPGAFLGLK |
| P35858 | Insulin-like growth faCtor-binding protein Complex aCid labile subunit | VAGLLEDTFPGLLGLR |
| P35858 | Insulin-like growth faCtor-binding protein Complex aCid labile subunit | LAYLQPALFSGLAELR |
| P19827 | Inter-alpha-trypsin inhibitor heavy Chain H1 | GSLVQASEANLQAAQDFVR |
| P19827 | Inter-alpha-trypsin inhibitor heavy Chain H1 | FAHYVVTSQVVNTANEAR |
| P19827 | Inter-alpha-trypsin inhibitor heavy Chain H1 | TAFISDFAVTADGNAFIGDIK |
| P19827 | Inter-alpha-trypsin inhibitor heavy Chain H1 | ILGDMQPGDYFDLVLFGTR |
| P19827 | Inter-alpha-trypsin inhibitor heavy Chain H1 | GFSLDEATNLNGGLLR |
| P19827 | Inter-alpha-trypsin inhibitor heavy Chain H1 | GHVLFRPTVSQQQSCPTCSTSLLNGHFK |
| P19827 | Inter-alpha-trypsin inhibitor heavy Chain H1 | FAHYVVTSQVVNTANEAREVAFDLEIPK |
| P19827 | Inter-alpha-trypsin inhibitor heavy Chain H1 | RNHMQYEIVIK |
| P19827 | Inter-alpha-trypsin inhibitor heavy Chain H1 | RQAVDTAVDGVFIR |
| P19827 | Inter-alpha-trypsin inhibitor heavy Chain H1 | ADVQAHGEGQEFSITCLVDEEEMKK |
| P19827 | Inter-alpha-trypsin inhibitor heavy Chain H1 | QAVDTAVDGVFIR |
| P19827 | Inter-alpha-trypsin inhibitor heavy Chain H1 | AAISGENAGLVR |
| P19827 | Inter-alpha-trypsin inhibitor heavy Chain H1 | KAAISGENAGLVR |
| P19827 | Inter-alpha-trypsin inhibitor heavy Chain H1 | GHMLENHVER |
| P19827 | Inter-alpha-trypsin inhibitor heavy Chain H1 | IADNKQSSFK |
| P19827 | Inter-alpha-trypsin inhibitor heavy Chain H1 | LDAQASFLPK |
| P19827 | Inter-alpha-trypsin inhibitor heavy Chain H1 | EVAFDLEIPK |
| P19827 | Inter-alpha-trypsin inhibitor heavy Chain H1 | GMADQDGLKPTIDKPSEDSPPLEMLGPR |
| P19827 | Inter-alpha-trypsin inhibitor heavy Chain H1 | VTYDVSRDK |
| P19827 | Inter-alpha-trypsin inhibitor heavy Chain H1 | TMEQFTIHLTVNPQSK |
| P19827 | Inter-alpha-trypsin inhibitor heavy Chain H1 | VTFQLTYEEVLK |
| P19827 | Inter-alpha-trypsin inhibitor heavy Chain H1 | QYYEGSEIVVAGR |
| P19827 | Inter-alpha-trypsin inhibitor heavy Chain H1 | GIEILNQVQESLPELSNHASILIMLTDGDPTEGVTDR |
| Q06033 | Inter-alpha-trypsin inhibitor heavy Chain H3 | EVSFDVELPK |
| P20591 | Interferon-induCed GTP-binding protein Mx1 | DVPDLTLIDLPGITR |
| P20591 | Interferon-induCed GTP-binding protein Mx1 | ALGVEQDLALPAIAVIGDQSSGK |
| P32455 | Interferon-induCed guanylate-binding protein 1 | TLSGGIQVNGPR |
| P32455 | Interferon-induCed guanylate-binding protein 1 | VKAESAQASAK |
| P32455 | Interferon-induCed guanylate-binding protein 1 | DFSLDLEADGQPLTPDEYLTYSLK |
| P32455 | Interferon-induCed guanylate-binding protein 1 | LQEQEQLLK |
| Q12905 | Interleukin enhanCer-binding faCtor 2 | AQDPSEVLTMLTNETGFEISSSDATVK |
| Q12905 | Interleukin enhanCer-binding faCtor 2 | ILPTLEAVAALGNK |
| Q12905 | Interleukin enhanCer-binding faCtor 2 | RNQDLAPNSAEQASILSLVTK |
| Q12905 | Interleukin enhanCer-binding faCtor 2 | VLQSALAAIR |
| P40189 | Interleukin-6 reCeptor subunit beta | ILDYEVTLTR |
| P40189 | Interleukin-6 reCeptor subunit beta | SVIILK |
| Q2TAA2 | Isoamyl aCetate-hydrolyzing esterase 1 homolog | IILPR |
| Q2TAA2 | Isoamyl aCetate-hydrolyzing esterase 1 homolog | VILITPTPLCETAWEEQCIIQGCK |
| Q96CN7 | IsoChorismatase domain-Containing protein 1 | GLGSTVQEIDLTGVK |
| Q96CN7 | IsoChorismatase domain-Containing protein 1 | ILGIPVIVTEQYPK |
| Q96CN7 | IsoChorismatase domain-Containing protein 1 | FSMVLPEVEAALAEIPGVR |
| O75874 | IsoCitrate dehydrogenase [NADP] CytoplasmiC | GQETSTNPIASIFAWTR |
| O75874 | IsoCitrate dehydrogenase [NADP] CytoplasmiC | ISGGSVVEMQGDEMTR |
| O75874 | IsoCitrate dehydrogenase [NADP] CytoplasmiC | LIDDMVAQAMK |
| O75874 | IsoCitrate dehydrogenase [NADP] CytoplasmiC | TVEAEAAHGTVTR |
| O75874 | IsoCitrate dehydrogenase [NADP] CytoplasmiC | SEGGFIWACK |
| O75874 | IsoCitrate dehydrogenase [NADP] CytoplasmiC | SDYLNTFEFMDK |
| O75874 | IsoCitrate dehydrogenase [NADP] CytoplasmiC | VTYLVHNFEEGGGVAMGMYNQDK |
| O75874 | IsoCitrate dehydrogenase [NADP] CytoplasmiC | LVSGWVKPIIIGR |
| O75874 | IsoCitrate dehydrogenase [NADP] CytoplasmiC | ATDFVVPGPGKVEITYTPSDGTQK |
| O75874 | IsoCitrate dehydrogenase [NADP] CytoplasmiC | KISGGSVVEMQGDEMTR |
| O75874 | IsoCitrate dehydrogenase [NADP] CytoplasmiC | SIEDFAHSSFQMALSK |
| O75874 | IsoCitrate dehydrogenase [NADP] CytoplasmiC | NYDGDVQSDSVAQGYGSLGMMTSVLVCPDGKTVEAEAAHGTVTR |
| O75874 | IsoCitrate dehydrogenase [NADP] CytoplasmiC | DAAEAIKK |
| O75874 | IsoCitrate dehydrogenase [NADP] CytoplasmiC | FKDIFQEIYDK |
| O75874 | IsoCitrate dehydrogenase [NADP] CytoplasmiC | VEITYTPSDGTQK |
| O75874 | IsoCitrate dehydrogenase [NADP] CytoplasmiC | DIFQEIYDK |
| O75874 | IsoCitrate dehydrogenase [NADP] CytoplasmiC | GWPLYLSTK |
| O75874 | IsoCitrate dehydrogenase [NADP] CytoplasmiC | NYDGDVQSDSVAQGYGSLGMMTSVLVCPDGK |
| O75874 | IsoCitrate dehydrogenase [NADP] CytoplasmiC | LGENLK |
| O75874 | IsoCitrate dehydrogenase [NADP] CytoplasmiC | ATDFVVPGPGK |
| O75874 | IsoCitrate dehydrogenase [NADP] CytoplasmiC | LAQAKL |
| O75874 | IsoCitrate dehydrogenase [NADP] CytoplasmiC | HAYGDQYR |
| O75874 | IsoCitrate dehydrogenase [NADP] CytoplasmiC | DLAACIK |
| O75874 | IsoCitrate dehydrogenase [NADP] CytoplasmiC | NILGGTVFR |
| O75874 | IsoCitrate dehydrogenase [NADP] CytoplasmiC | CATITPDEK |
| O75874 | IsoCitrate dehydrogenase [NADP] CytoplasmiC | SPNGTIR |
| O75874 | IsoCitrate dehydrogenase [NADP] CytoplasmiC | CATITPDEKR |
| O75874 | IsoCitrate dehydrogenase [NADP] CytoplasmiC | SQFEAQK |
| O75874 | IsoCitrate dehydrogenase [NADP] CytoplasmiC | NTILKK |
| O75874 | IsoCitrate dehydrogenase [NADP] CytoplasmiC | IIWELIK |
| P53990 | IST1 homolog | FGLIQSMK |
| P53990 | IST1 homolog | ELDSGLAESVSTLIWAAPR |
| P53990 | IST1 homolog | YLIEIAK |
| P14923 | JunCtion plakoglobin | ILVNQLSVDDVNVLTCATGTLSNLTCNNSK |
| Q9UBX7 | Kallikrein-11 | LLCGATLIAPR |
| Q9UBX7 | Kallikrein-11 | YVDWIQETmK |
| P29622 | Kallistatin | WADLSGITK |
| P13645 | Keratin, type I CytOSkeletal 10 | GSLGGGFSSGGFSGGSFSR |
| P13645 | Keratin, type I CytOSkeletal 10 | ALEESNYELEGK |
| P13645 | Keratin, type I CytOSkeletal 10 | NVSTGDVNVEMNAAPGVDLTQLLNNMR |
| P13645 | Keratin, type I CytOSkeletal 10 | TIDDLKNQILNLTTDNANILLQIDNAR |
| P13645 | Keratin, type I CytOSkeletal 10 | VRALEESNYELEGK |
| P13645 | Keratin, type I CytOSkeletal 10 | VTmQNLNDR |
| P13645 | Keratin, type I CytOSkeletal 10 | YCVQLSQIQAQISALEEQLQQIR |
| P13645 | Keratin, type I CytOSkeletal 10 | NQILNLTTDNANILLQIDNAR |
| P13645 | Keratin, type I CytOSkeletal 10 | DAEAWFNEK |
| P13645 | Keratin, type I CytOSkeletal 10 | IRLENEIQTYR |
| P13645 | Keratin, type I CytOSkeletal 10 | VLDELTLTK |
| P13645 | Keratin, type I CytOSkeletal 10 | VLDELTLTKADLEMQIESLTEELAYLK |
| P13645 | Keratin, type I CytOSkeletal 10 | SQYEQLAEQNRK |
| Q04695 | Keratin, type I CytOSkeletal 17 | QFTSSSSIK |
| P08727 | Keratin, type I CytOSkeletal 19 | VLDELTLAR |
| P08727 | Keratin, type I CytOSkeletal 19 | AALEDTLAETEAR |
| P08727 | Keratin, type I CytOSkeletal 19 | ILGATIENSR |
| P08727 | Keratin, type I CytOSkeletal 19 | SRLEQEIATYR |
| P08727 | Keratin, type I CytOSkeletal 19 | TKFETEQALR |
| P35527 | Keratin, type I CytOSkeletal 9 | GGSGGSYGGGGSGGGYGGGSGSR |
| P35527 | Keratin, type I CytOSkeletal 9 | FSSSSGYGGGSSR |
| P35527 | Keratin, type I CytOSkeletal 9 | EIETYHNLLEGGQEDFESSGAGK |
| P35527 | Keratin, type I CytOSkeletal 9 | TLLDIDNTR |
| P35527 | Keratin, type I CytOSkeletal 9 | GGGGSFGYSYGGGSGGGFSASSLGGGFGGGSR |
| P35527 | Keratin, type I CytOSkeletal 9 | VQALEEANNDLENK |
| P35527 | Keratin, type I CytOSkeletal 9 | STMQELNSR |
| P35527 | Keratin, type I CytOSkeletal 9 | DIENQYETQITQIEHEVSSSGQEVQSSAK |
| P35527 | Keratin, type I CytOSkeletal 9 | SGGGGGGGLGSGGSIR |
| P35527 | Keratin, type I CytOSkeletal 9 | QGVDADINGLR |
| P04264 | Keratin, type II CytOSkeletal 1 | GGGGGGYGSGGSSYGSGGGSYGSGGGGGGGR |
| P04264 | Keratin, type II CytOSkeletal 1 | SLDLDSIIAEVK |
| P04264 | Keratin, type II CytOSkeletal 1 | SKAEAESLYQSK |
| P04264 | Keratin, type II CytOSkeletal 1 | GGSGGGGGGSSGGRGSGGGSSGGSIGGR |
| P04264 | Keratin, type II CytOSkeletal 1 | TNAENEFVTIKK |
| P04264 | Keratin, type II CytOSkeletal 1 | YEELQITAGR |
| P04264 | Keratin, type II CytOSkeletal 1 | SLVNLGGSK |
| P04264 | Keratin, type II CytOSkeletal 1 | AQYEDIAQK |
| P04264 | Keratin, type II CytOSkeletal 1 | FLEQQNQVLQTKWELLQQVDTSTR |
| P04264 | Keratin, type II CytOSkeletal 1 | QISNLQQSISDAEQRGENALK |
| P04264 | Keratin, type II CytOSkeletal 1 | FSSCGGGGGSFGAGGGFGSR |
| P04264 | Keratin, type II CytOSkeletal 1 | SLNNQFASFIDK |
| P04264 | Keratin, type II CytOSkeletal 1 | THNLEPYFESFINNLR |
| P35908 | Keratin, type II CytOSkeletal 2 epidermal | GGGFGGGSSFGGGSGFSGGGFGGGGFGGGR |
| P35908 | Keratin, type II CytOSkeletal 2 epidermal | GGSGGGGSISGGGYGSGGGSGGR |
| P35908 | Keratin, type II CytOSkeletal 2 epidermal | GSSSGGGYSSGSSSYGSGGR |
| P35908 | Keratin, type II CytOSkeletal 2 epidermal | GGSSSGGGYGSGGGGSSSVK |
| P35908 | Keratin, type II CytOSkeletal 2 epidermal | VDLLNQEIEFLK |
| P35908 | Keratin, type II CytOSkeletal 2 epidermal | AAFGGSGGRGSSSGGGYSSGSSSYGSGGR |
| P35908 | Keratin, type II CytOSkeletal 2 epidermal | NLDLDSIIAEVK |
| P35908 | Keratin, type II CytOSkeletal 2 epidermal | GGSISGGGYGSGGGK |
| P35908 | Keratin, type II CytOSkeletal 2 epidermal | GFSSGSAVVSGGSR |
| P08729 | Keratin, type II CytOSkeletal 7 | SLDLDGIIAEVK |
| P08729 | Keratin, type II CytOSkeletal 7 | SSRLPDIFEAQIAGLR |
| P08729 | Keratin, type II CytOSkeletal 7 | FETLQAQAGK |
| P33176 | Kinesin-1 heavy Chain | LITDLQDQNQK |
| Q04760 | LaCtoylglutathione lyase | DFLLQQTMLR |
| Q04760 | LaCtoylglutathione lyase | GLAFIQDPDGYWIEILNPNK |
| Q04760 | LaCtoylglutathione lyase | RFEELGVK |
| Q04760 | LaCtoylglutathione lyase | VLGMTLIQK |
| Q04760 | LaCtoylglutathione lyase | CDFPIMK |
| Q04760 | LaCtoylglutathione lyase | FVKKPDDGK |
| Q04760 | LaCtoylglutathione lyase | SLDFYTR |
| Q9Y2S2 | Lambda-Crystallin homolog | LVEEGIVSPSDLDLVMSEGLGMR |
| P07942 | Laminin subunit beta-1 | NFLTQDSADLDSIEAVANEVLK |
| P07942 | Laminin subunit beta-1 | ELDSLQTEAESLDNTVK |
| P11047 | Laminin subunit gamma-1 | EVVCTNCPTGTTGK |
| P11047 | Laminin subunit gamma-1 | VSVPLIAQGNSYPSETTVK |
| P11047 | Laminin subunit gamma-1 | NTIEETGNLAEQAR |
| P11047 | Laminin subunit gamma-1 | DGFFGNPLAPNPADK |
| P11047 | Laminin subunit gamma-1 | LNTFGDEVFNDPK |
| P11047 | Laminin subunit gamma-1 | LGNNEACSSCHCSPVGSLSTQCDSYGR |
| P11047 | Laminin subunit gamma-1 | TGQCECQPGITGQHCER |
| P11047 | Laminin subunit gamma-1 | LIEIASR |
| P11047 | Laminin subunit gamma-1 | ACNCNLYGTMK |
| P11047 | Laminin subunit gamma-1 | STGHGGHCTNCQDNTDGAHCER |
| Q7L266 | L-asparaginase | TVEEAADLSLGYMK |
| Q7L266 | L-asparaginase | DLSAGAVSAVQCIANPIK |
| Q7L266 | L-asparaginase | VGDSPCLGAGGYADNDIGAVSTTGHGESILK |
| Q7L266 | L-asparaginase | GLGGLIVVSK |
| Q7L266 | L-asparaginase | GNVAYATSTGGIVNK |
| Q7L266 | L-asparaginase | LTLFHIEQGK |
| Q7L266 | L-asparaginase | AATVGYGILR |
| Q7L266 | L-asparaginase | WTSTSMPWAAAK |
| Q7L266 | L-asparaginase | TGDWVAK |
| Q7L266 | L-asparaginase | LHFGIDPDDTTITDLP |
| Q7L266 | L-asparaginase | TPHCFLTDQGAAQFAAAMGVPEIPGEK |
| Q7L266 | L-asparaginase | VHQGMVR |
| Q8N2S1 | Latent-transforming growth faCtor beta-binding protein 4 | GPGAPCQDVDECAR |
| Q8N2S1 | Latent-transforming growth faCtor beta-binding protein 4 | SRGPGAPCQDVDECAR |
| Q8N2S1 | Latent-transforming growth faCtor beta-binding protein 4 | AGPDLASCLDVDECRER |
| Q9BS40 | Latexin | FAVEEIIQK |
| P02750 | LeuCine-riCh alpha-2-glyCoprotein | VAAGAFQGLR |
| P02750 | LeuCine-riCh alpha-2-glyCoprotein | LQELHLSSNGLESLSPEFLRPVPQLR |
| P02750 | LeuCine-riCh alpha-2-glyCoprotein | ENQLEVLEVSWLHGLK |
| P02750 | LeuCine-riCh alpha-2-glyCoprotein | NALTGLPPGLFQASATLDTLVLK |
| P02750 | LeuCine-riCh alpha-2-glyCoprotein | DLLLPQPDLR |
| P02750 | LeuCine-riCh alpha-2-glyCoprotein | GPLQLER |
| P02750 | LeuCine-riCh alpha-2-glyCoprotein | DGFDISGNPWICDQNLSDLYR |
| P02750 | LeuCine-riCh alpha-2-glyCoprotein | TLDLGENQLETLPPDLLR |
| P02750 | LeuCine-riCh alpha-2-glyCoprotein | TLDLGENQLETLPPDLLRGPLQLER |
| P02750 | LeuCine-riCh alpha-2-glyCoprotein | DCQVFR |
| P02750 | LeuCine-riCh alpha-2-glyCoprotein | GQTLLAVAK |
| P02750 | LeuCine-riCh alpha-2-glyCoprotein | ALGHLDLSGNR |
| P02750 | LeuCine-riCh alpha-2-glyCoprotein | LQVLGK |
| P02750 | LeuCine-riCh alpha-2-glyCoprotein | LHLEGNKLQVLGK |
| Q32MZ4 | LeuCine-riCh repeat flightless-interaCting protein 1 | SAVEAQNEVTENPK |
| Q9P2J5 | LeuCyl-tRNA synthetase, CytoplasmiC | LALADAGDTVEDANFVEAMADAGILR |
| P30740 | LeukoCyte elastase inhibitor | LGVQDLFNSSK |
| P30740 | LeukoCyte elastase inhibitor | TYNFLPEFLVSTQK |
| P30740 | LeukoCyte elastase inhibitor | TFHFNTVEEVHSR |
| P30740 | LeukoCyte elastase inhibitor | FQSLNADINKR |
| P30740 | LeukoCyte elastase inhibitor | TYGADLASVDFQHASEDAR |
| P30740 | LeukoCyte elastase inhibitor | KIEEQLTLEK |
| P30740 | LeukoCyte elastase inhibitor | GQTEGKIPELLASGMVDNMTK |
| Q08722 | LeukoCyte surfaCe antigen CD47 | IEVSQLLK |
| P09960 | Leukotriene A-4 hydrolase | PEIVDTCSLASPASVCR |
| P09960 | Leukotriene A-4 hydrolase | GSPMEISLPIALSK |
| P09960 | Leukotriene A-4 hydrolase | LVVDLTDIDPDVAYSSVPYEK |
| P09960 | Leukotriene A-4 hydrolase | MQEVYNFNAINNSEIR |
| P09960 | Leukotriene A-4 hydrolase | SAYEFSETESMLK |
| P09960 | Leukotriene A-4 hydrolase | LTYTAEVSVPK |
| P09960 | Leukotriene A-4 hydrolase | WITAKEDDLNSFNATDLK |
| P09960 | Leukotriene A-4 hydrolase | TFGETHPFTK |
| P09960 | Leukotriene A-4 hydrolase | TLTGTAALTVQSQEDNLR |
| P09960 | Leukotriene A-4 hydrolase | WEDAIPLALK |
| Q14847 | LIM and SH3 domain protein 1 | QSFTMVADTPENLR |
| Q14847 | LIM and SH3 domain protein 1 | QQSELQSQVR |
| Q14847 | LIM and SH3 domain protein 1 | TGDTGMLPANYVEAI |
| Q14847 | LIM and SH3 domain protein 1 | GFSVVADTPELQR |
| Q14847 | LIM and SH3 domain protein 1 | TQDQISNIK |
| Q14847 | LIM and SH3 domain protein 1 | ACFHCETCK |
| Q14847 | LIM and SH3 domain protein 1 | YHEEFEK |
| P07195 | L-laCtate dehydrogenase B Chain | GMYGIENEVFLSLPCILNAR |
| P07195 | L-laCtate dehydrogenase B Chain | HRVIGSGCNLDSAR |
| P07195 | L-laCtate dehydrogenase B Chain | SLADELALVDVLEDK |
| P07195 | L-laCtate dehydrogenase B Chain | LIAPVAEEEATVPNNK |
| P07195 | L-laCtate dehydrogenase B Chain | ITVVGVGQVGMACAISILGK |
| P07195 | L-laCtate dehydrogenase B Chain | YSPDCIIIVVSNPVDILTYVTWK |
| P07195 | L-laCtate dehydrogenase B Chain | mVVESAYEVIK |
| P07195 | L-laCtate dehydrogenase B Chain | LKDDEVAQLK |
| P07195 | L-laCtate dehydrogenase B Chain | DYSVTANSK |
| P07195 | L-laCtate dehydrogenase B Chain | LKGEMMDLQHGSLFLQTPK |
| P07195 | L-laCtate dehydrogenase B Chain | YLMAEK |
| P07195 | L-laCtate dehydrogenase B Chain | FIIPQIVK |
| P07195 | L-laCtate dehydrogenase B Chain | EKLIAPVAEEEATVPNNK |
| P07195 | L-laCtate dehydrogenase B Chain | IVVVTAGVR |
| P07195 | L-laCtate dehydrogenase B Chain | IVADKDYSVTANSK |
| P07195 | L-laCtate dehydrogenase B Chain | SADTLWDIQK |
| P07195 | L-laCtate dehydrogenase B Chain | GLTSVINQK |
| P07195 | L-laCtate dehydrogenase B Chain | NVNVFK |
| P07195 | L-laCtate dehydrogenase B Chain | LIAPVAEEEATVPNNKITVVGVGQVGMACAISILGK |
| Q8TDL5 | Long palate, lung and nasal epithelium CarCinoma-assoCiated protein 1 | ILTQDTPEFFIDQGHAK |
| P24666 | Low moleCular weight phOSphotyrOSine protein phOSphatase | HGIPMSHVAR |
| P24666 | Low moleCular weight phOSphotyrOSine protein phOSphatase | IELLGSYDPQK |
| P51884 | LumiCan | SLEYLDLSFNQIAR |
| P51884 | LumiCan | LPSGLPVSLLTLYLDNNK |
| P51884 | LumiCan | NIPTVNENLENYYLEVNQLEK |
| P51884 | LumiCan | SLEDLQLTHNK |
| P51884 | LumiCan | NNQIDHIDEK |
| P51884 | LumiCan | RFNALQYLR |
| P51884 | LumiCan | FNALQYLR |
| P51884 | LumiCan | ILGPLSYSK |
| P05455 | Lupus La protein | LTTDFNVIVEALSK |
| P05455 | Lupus La protein | SKAELMEISEDK |
| P05455 | Lupus La protein | ICHQIEYYFGDFNLPR |
| P05455 | Lupus La protein | GSIFVVFDSIESAK |
| P05455 | Lupus La protein | IGCLLK |
| P05455 | Lupus La protein | KIIEDQQESLNK |
| P05455 | Lupus La protein | EVTWEVLEGEVEK |
| P10253 | LysOSomal alpha-gluCOSidase | AITQEQCEAR |
| P11279 | LysOSome-assoCiated membrane glyCoprotein 1 | FFLQGIQLNTILPDARDPAFK |
| P13473 | LysOSome-assoCiated membrane glyCoprotein 2 | IPLNDLFR |
| P61626 | Lysozyme C | STDYGIFQINSR |
| P61626 | Lysozyme C | ATNYNAGDRSTDYGIFQINSR |
| P61626 | Lysozyme C | GISLANWMCLAK |
| P61626 | Lysozyme C | ATNYNAGDR |
| P61626 | Lysozyme C | TPGAVNACHLSCSALLQDNIADAVACAK |
| P61626 | Lysozyme C | AWVAWR |
| Q15046 | Lysyl-tRNA synthetase | LVGEFLEVTCINPTFICDHPQIMSPLAK |
| Q15046 | Lysyl-tRNA synthetase | SQAIHQLK |
| P40925 | Malate dehydrogenase, CytoplasmiC | VIVVGNPANTNCLTASK |
| P40925 | Malate dehydrogenase, CytoplasmiC | EVGVYEALKDDSWLK |
| P40925 | Malate dehydrogenase, CytoplasmiC | DVIATDKEDVAFK |
| P40925 | Malate dehydrogenase, CytoplasmiC | GEFVTTVQQR |
| P40925 | Malate dehydrogenase, CytoplasmiC | DLDVAILVGSMPR |
| P40925 | Malate dehydrogenase, CytoplasmiC | LGVTANDVK |
| P40925 | Malate dehydrogenase, CytoplasmiC | LSSAMSAAK |
| P40925 | Malate dehydrogenase, CytoplasmiC | AQIALK |
| P40925 | Malate dehydrogenase, CytoplasmiC | ELTEEKESAFEFLSSA |
| P40925 | Malate dehydrogenase, CytoplasmiC | KLSSAMSAAK |
| P40925 | Malate dehydrogenase, CytoplasmiC | ENFSCLTR |
| P40925 | Malate dehydrogenase, CytoplasmiC | ESAFEFLSSA |
| P40925 | Malate dehydrogenase, CytoplasmiC | MDLTAK |
| P40925 | Malate dehydrogenase, CytoplasmiC | FVEGLPINDFSR |
| P40926 | Malate dehydrogenase, mitoChondrial | VAVLGASGGIGQPLSLLLK |
| P40926 | Malate dehydrogenase, mitoChondrial | LTLYDIAHTPGVAADLSHIETK |
| Q9ULC4 | Malignant T Cell-amplified sequenCe 1 | LYPAAVDTIVAIMAEGK |
| Q9ULC4 | Malignant T Cell-amplified sequenCe 1 | FVLSGANIMCPGLTSPGAK |
| Q9ULC4 | Malignant T Cell-amplified sequenCe 1 | NQLIEQFPGIEPWLNQIMPK |
| Q96IJ6 | MannOSe-1-phOSphate guanyltransferase alpha | LLPAITILGCR |
| Q9Y5P6 | MannOSe-1-phOSphate guanyltransferase beta | IGQNCSIGPNVSLGPGVVVEDGVCIRR |
| Q9Y5P6 | MannOSe-1-phOSphate guanyltransferase beta | LCSGPGIVGNVLVDPSAR |
| P34949 | MannOSe-6-phOSphate isomerase | VFPLSCAVQQYAWGK |
| P34949 | MannOSe-6-phOSphate isomerase | SQEDPYLSIYDPPVPDFTIMK |
| P49006 | MARCKS-related protein | AAATPESQEPQAK |
| P49006 | MARCKS-related protein | GDVTAEEAAGASPAK |
| P43243 | Matrin-3 | DLSAAGIGLLAAATQSLSMPASLGR |
| Q9UNF1 | Melanoma-assoCiated antigen D2 | DSSSMMQTLLTVTQNVEVPETPK |
| P08582 | Melanotransferrin | HSTVLENTDGK |
| O00264 | Membrane-assoCiated progesterone reCeptor Component 1 | EALKDEYDDLSDLTAAQQETLSDWESQFTFK |
| O00264 | Membrane-assoCiated progesterone reCeptor Component 1 | IVRGDQPAASGDSDDDEPPPLPR |
| O00264 | Membrane-assoCiated progesterone reCeptor Component 1 | FYGPEGPYGVFAGR |
| O00264 | Membrane-assoCiated progesterone reCeptor Component 1 | GLATFCLDK |
| P55145 | MesenCephaliC astroCyte-derived neurotrophiC faCtor | LCYYIGATDDAATK |
| P02795 | Metallothionein-2 | SCCSCCPVGCAK |
| P02795 | Metallothionein-2 | CAQGCICK |
| Q9NZL9 | Methionine adenOSyltransferase 2 subunit beta | VLVTGATGLLGR |
| P53582 | Methionine aminopeptidase 1 | VCETDGCSSEAK |
| P22033 | Methylmalonyl-CoA mutase, mitoChondrial | AVAEGIPKLR |
| P46821 | MiCrotubule-assoCiated protein 1B | AAEAVAAAVGTGATTAAVMAAAGIAAIGPAK |
| P27816 | MiCrotubule-assoCiated protein 4 | GISEDSHLESLQDVGQSAAPTFMISPETVTGTGKK |
| P27816 | MiCrotubule-assoCiated protein 4 | KPTAIKTEGKPAEVK |
| P27816 | MiCrotubule-assoCiated protein 4 | TTTAAAVASTGPSSR |
| P27816 | MiCrotubule-assoCiated protein 4 | KPTSAKPSSTTPR |
| P27816 | MiCrotubule-assoCiated protein 4 | NVVLPTETEVAPAK |
| P27816 | MiCrotubule-assoCiated protein 4 | KTKPLATTQPAK |
| P27816 | MiCrotubule-assoCiated protein 4 | DVKPKPIADAK |
| Q15691 | MiCrotubule-assoCiated protein RP/EB family member 1 | LRNIELICQENEGENDPVLQR |
| P98088 | MuCin-5AC (Fragments) | MCLNYEVR |
| P98088 | MuCin-5AC (Fragments) | RPEEITR |
| P98088 | MuCin-5AC (Fragments) | AESFPNTPLADLGQDVICSHTEGLICLNK |
| P98088 | MuCin-5AC (Fragments) | NQDQQGPFK |
| P98088 | MuCin-5AC (Fragments) | VLCCETPR |
| Q6W4X9 | MuCin-6 | CEGACISAASFNIITQQVDAR |
| Q6W4X9 | MuCin-6 | VCAETHCSMLLR |
| Q6W4X9 | MuCin-6 | VTNEFVSEEGK |
| Q6W4X9 | MuCin-6 | CHATVNPAPFYK |
| Q6W4X9 | MuCin-6 | ILTENVICGNSGVTCSR |
| Q6W4X9 | MuCin-6 | DAFPTFSVQLR |
| Q6W4X9 | MuCin-6 | VYHLPYYEACVR |
| Q6W4X9 | MuCin-6 | DACGCDSGGDCECLCDAVAAYAQACLDK |
| Q6W4X9 | MuCin-6 | AQCPCILEGYK |
| Q6W4X9 | MuCin-6 | CSVINSQTFATCHSK |
| P22234 | MultifunCtional protein ADE2 | ITSCIFQLLQEAGIK |
| P22234 | MultifunCtional protein ADE2 | ACGNFGIPCELR |
| P22234 | MultifunCtional protein ADE2 | LPSGLGCSTVLSPEGSAQFAAQIFGLSNHLVWSK |
| O43196 | MutS protein homolog 5 | ALGGLLK |
| P02689 | Myelin P2 protein | LVSSENFDDYMK |
| P24158 | Myeloblastin | LVNVVLGAHNVR |
| P24158 | Myeloblastin | TQEPTQQHFSVAQVFLNNYDAENK |
| P24158 | Myeloblastin | GNPGSHFCGGTLIHPSFVLTAAHCLR |
| Q8WXC6 | Myeloma-overexpressed gene 2 protein | AVHADFFNDFEDLFDDDDIQ |
| P60660 | MyOSin light polypeptide 6 | VLDFEHFLPMLQTVAK |
| P60660 | MyOSin light polypeptide 6 | ILYSQCGDVMR |
| P60660 | MyOSin light polypeptide 6 | ALGQNPTNAEVLK |
| P60660 | MyOSin light polypeptide 6 | NKDQGTYEDYVEGLR |
| P60660 | MyOSin light polypeptide 6 | HVLVTLGEK |
| P60660 | MyOSin light polypeptide 6 | EAFQLFDR |
| P60660 | MyOSin light polypeptide 6 | SDEMNVK |
| P19105 | MyOSin regulatory light Chain 12A | ATSNVFAMFDQSQIQEFK |
| P19105 | MyOSin regulatory light Chain 12A | GNFNYIEFTR |
| P35579 | MyOSin-9 | IAEFTTNLTEEEEK |
| P35579 | MyOSin-9 | KLEEEQIILEDQNCK |
| P35579 | MyOSin-9 | KVEAQLQELQVK |
| P35579 | MyOSin-9 | NFINNPLAQADWAAK |
| P35579 | MyOSin-9 | ANLQIDQINTDLNLER |
| P35579 | MyOSin-9 | TQLEELEDELQATEDAK |
| P35579 | MyOSin-9 | QLLQANPILEAFGNAK |
| P35579 | MyOSin-9 | IAQLEEELEEEQGNTELINDRLK |
| P35579 | MyOSin-9 | ASITALEAK |
| P35579 | MyOSin-9 | IAQLEEELEEEQGNTELINDR |
| P35579 | MyOSin-9 | SMEAEMIQLQEELAAAER |
| P35579 | MyOSin-9 | DLEAHIDSANK |
| P35579 | MyOSin-9 | NSFREQLEEEEEAK |
| P35579 | MyOSin-9 | TRLQQELDDLLVDLDHQR |
| P35579 | MyOSin-9 | KLEGDSTDLSDQIAELQAQIAELK |
| P35579 | MyOSin-9 | DFSALESQLQDTQELLQEENRQK |
| P35579 | MyOSin-9 | RALEQQVEEMK |
| P35579 | MyOSin-9 | HSQAVEELAEQLEQTK |
| P35579 | MyOSin-9 | KFDQLLAEEK |
| P35579 | MyOSin-9 | DLQGRDEQSEEK |
| P35579 | MyOSin-9 | GALALEEK |
| P35579 | MyOSin-9 | KLVWVPSDK |
| P35579 | MyOSin-9 | IAQLEEQLDNETK |
| P35579 | MyOSin-9 | ELEDATETADAMNR |
| P35579 | MyOSin-9 | DFSALESQLQDTQELLQEENR |
| P35579 | MyOSin-9 | IIGLDQVAGMSETALPGAFK |
| P35579 | MyOSin-9 | NTDQASMPDNTAAQK |
| P35579 | MyOSin-9 | ALEEAMEQKAELER |
| P35579 | MyOSin-9 | QAQQERDELADEIANSSGK |
| P35579 | MyOSin-9 | VISGVLQLGNIVFK |
| P35579 | MyOSin-9 | KEEELQAALAR |
| P35579 | MyOSin-9 | RQLEEAEEEAQR |
| P35579 | MyOSin-9 | IRELESQISELQEDLESER |
| P35579 | MyOSin-9 | LEGDSTDLSDQIAELQAQIAELK |
| P35579 | MyOSin-9 | NLPIYSEEIVEMYK |
| P35579 | MyOSin-9 | ALEQQVEEMKTQLEELEDELQATEDAK |
| P35579 | MyOSin-9 | VMQEQGTHPK |
| P35579 | MyOSin-9 | SVHELEK |
| P35579 | MyOSin-9 | VKPLLQVSR |
| P58546 | Myotrophin | GPDGLTAFEATDNQAIK |
| P58546 | Myotrophin | TVKGPDGLTAFEATDNQAIK |
| P58546 | Myotrophin | DYVAKGEDVNR |
| O94760 | N(G),N(G)-dimethylarginine dimethylaminohydrolase 1 | SFCSMAGPNLIAIGSSESAQK |
| O94760 | N(G),N(G)-dimethylarginine dimethylaminohydrolase 1 | GAEILADTFK |
| O94760 | N(G),N(G)-dimethylarginine dimethylaminohydrolase 1 | LGLQVVELPADESLPDCVFVEDVAVVCEETALITRPGAPSR |
| O94760 | N(G),N(G)-dimethylarginine dimethylaminohydrolase 1 | VDGLLTCCSVLINK |
| O94760 | N(G),N(G)-dimethylarginine dimethylaminohydrolase 1 | LTVPDDIAANCIYLNIPNK |
| O94760 | N(G),N(G)-dimethylarginine dimethylaminohydrolase 1 | SAKGEEVDVAR |
| O95865 | N(G),N(G)-dimethylarginine dimethylaminohydrolase 2 | GGGDLPNSQEALQK |
| O95865 | N(G),N(G)-dimethylarginine dimethylaminohydrolase 2 | IVEIGDENATLDGTDVLFTGR |
| O95865 | N(G),N(G)-dimethylarginine dimethylaminohydrolase 2 | LSDVTLVPVSCSELEK |
| O95865 | N(G),N(G)-dimethylarginine dimethylaminohydrolase 2 | AGAGLSSLCLVLSTRPHS |
| O95865 | N(G),N(G)-dimethylarginine dimethylaminohydrolase 2 | ALQDLGLR |
| O95865 | N(G),N(G)-dimethylarginine dimethylaminohydrolase 2 | RPEVDGVRK |
| O95865 | N(G),N(G)-dimethylarginine dimethylaminohydrolase 2 | LGLQLLELPPEESLPLGPLLGDTAVIQGDTALITRPWSPAR |
| O95865 | N(G),N(G)-dimethylarginine dimethylaminohydrolase 2 | TVVAGSSDAAQK |
| O95865 | N(G),N(G)-dimethylarginine dimethylaminohydrolase 2 | GAEIVADTFRDFAVSTVPVSGPSHLR |
| O14745 | Na(+)/H(+) exChange regulatory CofaCtor NHE-RF1 | LLVVDPETDEQLQK |
| O14745 | Na(+)/H(+) exChange regulatory CofaCtor NHE-RF1 | SVDPDSPAEASGLR |
| O14745 | Na(+)/H(+) exChange regulatory CofaCtor NHE-RF1 | AALNAVR |
| O14745 | Na(+)/H(+) exChange regulatory CofaCtor NHE-RF1 | LGVQVR |
| O14745 | Na(+)/H(+) exChange regulatory CofaCtor NHE-RF1 | EALAEAALESPRPALVR |
| Q9UJ70 | N-aCetyl-D-gluCOSamine kinase | IVFDSIDNLEAAPHDIGYVK |
| Q9UJ70 | N-aCetyl-D-gluCOSamine kinase | SLGLSLSGGDQEDAGR |
| Q9UJ70 | N-aCetyl-D-gluCOSamine kinase | IGLPILCVGSVWK |
| Q96PD5 | N-aCetylmuramoyl-L-alanine amidase | TDCPGDALFDLLR |
| Q96PD5 | N-aCetylmuramoyl-L-alanine amidase | EYGVVLAPDGSTVAVEPLLAGLEAGLQGR |
| Q96PD5 | N-aCetylmuramoyl-L-alanine amidase | AGLLRPDYALLGHR |
| Q96PD5 | N-aCetylmuramoyl-L-alanine amidase | GSQTQSHPDLGTEGCWDQLSAPR |
| P48163 | NADP-dependent maliC enzyme | QITDNIFLTTAEVIAQQVSDK |
| Q13765 | NasCent polypeptide-assoCiated Complex subunit alpha | IEDLSQQAQLAAAEK |
| Q13765 | NasCent polypeptide-assoCiated Complex subunit alpha | SPASDTYIVFGEAKIEDLSQQAQLAAAEK |
| Q13765 | NasCent polypeptide-assoCiated Complex subunit alpha | SPASDTYIVFGEAK |
| Q13765 | NasCent polypeptide-assoCiated Complex subunit alpha | NILFVITKPDVYK |
| Q13765 | NasCent polypeptide-assoCiated Complex subunit alpha | NNSNDIVNAIMELTM |
| Q15843 | NEDD8 | EIEIDIEPTDKVER |
| Q8TBC4 | NEDD8-aCtivating enzyme E1 CatalytiC subunit | IIPAVASTNAVIAAVCATEVFK |
| Q8TBC4 | NEDD8-aCtivating enzyme E1 CatalytiC subunit | ELGLVDGQELAVADVTTPQTVLFK |
| Q13564 | NEDD8-aCtivating enzyme E1 regulatory subunit | DAAAVGNHVAK |
| Q13564 | NEDD8-aCtivating enzyme E1 regulatory subunit | LLCSNSAFLR |
| Q6ZS30 | NeurobeaChin-like protein 1 | FIALQIK |
| Q09666 | Neuroblast differentiation-assoCiated protein AHNAK | ISMPDVDLHLK |
| Q09666 | Neuroblast differentiation-assoCiated protein AHNAK | ISMPDIDLNLK |
| Q09666 | Neuroblast differentiation-assoCiated protein AHNAK | ADLDVSGPKVDIDVPDVNIEGPEGK |
| Q09666 | Neuroblast differentiation-assoCiated protein AHNAK | AEGPEVDVNLPK |
| Q09666 | Neuroblast differentiation-assoCiated protein AHNAK | FKMPEMNIK |
| Q09666 | Neuroblast differentiation-assoCiated protein AHNAK | VDIDAPDVDVHGPDWHLK |
| A2RRP1 | Neuroblastoma-amplified sequenCe | SIDLVLAASR |
| Q92823 | Neuronal Cell adhesion moleCule | YIVSGTPTFVPYLIK |
| Q92823 | Neuronal Cell adhesion moleCule | AAPYWITAPQNLVLSPGEDGTLICR |
| Q92823 | Neuronal Cell adhesion moleCule | DSTGTYTCVAR |
| P51674 | Neuronal membrane glyCoprotein M6-a | YEDIK |
| O14786 | Neuropilin-1 | FVTAVGTQGAISK |
| Q99574 | NeurOSerpin | DFDAATYLALINAVYFK |
| Q99574 | NeurOSerpin | ALGITEIFIK |
| Q14697 | Neutral alpha-gluCOSidase AB | FSFSGNTLVSSSADPEGHFETPIWIER |
| Q14697 | Neutral alpha-gluCOSidase AB | VTEGGEPYR |
| Q14697 | Neutral alpha-gluCOSidase AB | QYASLTGTQALPPLFSLGYHQSR |
| Q14697 | Neutral alpha-gluCOSidase AB | SLLLSVNAR |
| P59665 | Neutrophil defensin 1 | IPACIAGER |
| P59665 | Neutrophil defensin 1 | YGTCIYQGR |
| P08246 | Neutrophil elastase | RSNVCTLVR |
| P08246 | Neutrophil elastase | QAGVCFGDSGSPLVCNGLIHGIASFVR |
| P08246 | Neutrophil elastase | VVLGAHNLSR |
| P08246 | Neutrophil elastase | SNVCTLVR |
| P08246 | Neutrophil elastase | QVFAVQR |
| Q96TA1 | Niban-like protein 1 | ILTSVDQYLELIGNSLPGTTAK |
| Q6XQN6 | NiCotinate phOSphoribOSyltransferase | LCLQQGQLCEPLPSLAESR |
| Q6XQN6 | NiCotinate phOSphoribOSyltransferase | AAARPLLTDLYQATMALGYWR |
| Q6XQN6 | NiCotinate phOSphoribOSyltransferase | GVPVAGTLAHSFVTSFSGSEVPPDPMLAPAAGEGPGVDLAAK |
| Q6XQN6 | NiCotinate phOSphoribOSyltransferase | LLGSDGSPLMDMLQLAEEPVPQAGQELR |
| Q15274 | NiCotinate-nuCleotide pyrophOSphorylase [Carboxylating] | CSGIASAAAAAVEAAR |
| Q15274 | NiCotinate-nuCleotide pyrophOSphorylase [Carboxylating] | VEVECSSLQEAVQAAEAGADLVLLDNFKPEELHPTATVLK |
| Q15274 | NiCotinate-nuCleotide pyrophOSphorylase [Carboxylating] | DNHVVAAGGVEK |
| Q15274 | NiCotinate-nuCleotide pyrophOSphorylase [Carboxylating] | GPAHCLLLGER |
| P05204 | Non-histone ChromOSomal protein HMG-17 | LSAKPAPPKPEPKPK |
| Q9UNZ2 | NSFL1 CofaCtor p47 | LGAAPEEESAYVAGEK |
| P49321 | NuClear autoantigeniC sperm protein | HLVMGDIPAAVNAFQEAASLLGK |
| Q14980 | NuClear mitotiC apparatus protein 1 | LTAQVASLTSELTTLNATIQQQDQELAGLK |
| P61970 | NuClear transport faCtor 2 | NINDAWVCTNDMFR |
| P61970 | NuClear transport faCtor 2 | LALHNFG |
| Q9H1E3 | NuClear ubiquitous Casein and CyClin-dependent kinases substrate | VVDYSQFQESDDADEDYGRDSGPPTK |
| Q9H1E3 | NuClear ubiquitous Casein and CyClin-dependent kinases substrate | VGRPTASK |
| Q9H1E3 | NuClear ubiquitous Casein and CyClin-dependent kinases substrate | ATVTPSPVK |
| P67809 | NuClease-sensitive element-binding protein 1 | GAEAANVTGPGGVPVQGSK |
| P67809 | NuClease-sensitive element-binding protein 1 | NYQQNYQNSESGEK |
| P67809 | NuClease-sensitive element-binding protein 1 | NEGSESAPEGQAQQR |
| P67809 | NuClease-sensitive element-binding protein 1 | SVGDGETVEFDVVEGEK |
| P67809 | NuClease-sensitive element-binding protein 1 | AADPPAENSSAPEAEQGGAE |
| P67809 | NuClease-sensitive element-binding protein 1 | RPQYSNPPVQGEVMEGADNQGAGEQGRPVR |
| Q02818 | NuCleobindin-1 | LSQETEALGR |
| P80303 | NuCleobindin-2 | LHDVNSDGFLDEQELEALFTK |
| P06748 | NuCleophOSmin | MSVQPTVSLGGFEITPPVVLR |
| P06748 | NuCleophOSmin | GPSSVEDIK |
| P06748 | NuCleophOSmin | MTDQEAIQDLWQWRK |
| P06748 | NuCleophOSmin | SAPGGGSKVPQK |
| P06748 | NuCleophOSmin | VTLATLK |
| P06748 | NuCleophOSmin | MTDQEAIQDLWQWR |
| P06748 | NuCleophOSmin | TVSLGAGAKDELHIVEAEAMNYEGSPIK |
| P55209 | NuCleOSome assembly protein 1-like 1 | LDGLVETPTGYIESLPR |
| Q99733 | NuCleOSome assembly protein 1-like 4 | NVDMLSELVQEYDEPILK |
| Q99733 | NuCleOSome assembly protein 1-like 4 | GIPEFWFTIFR |
| Q99733 | NuCleOSome assembly protein 1-like 4 | LTDQVMQNPR |
| Q99733 | NuCleOSome assembly protein 1-like 4 | AAATAEEPDPK |
| Q9NTK5 | Obg-like ATPase 1 | IPAFLNVVDIAGLVK |
| Q9NTK5 | Obg-like ATPase 1 | YLEANMTQSALPK |
| Q9NTK5 | Obg-like ATPase 1 | IGIVGLPNVGK |
| Q9NY56 | Odorant-binding protein 2a | LIYLQELPGTDDYVFYCK |
| Q9NPH6 | Odorant-binding protein 2b | GLSEEDIFTPLQTGSCVPEH |
| Q9NQR4 | Omega-amidase NIT2 | LYNTCAVFGPDGTLLAK |
| Q9NQR4 | Omega-amidase NIT2 | VGLGICYDMR |
| Q92882 | OSteoClast-stimulating faCtor 1 | GYADIVQLLLAK |
| P20962 | ParathymOSin | SVEAAAELSAK |
| P20962 | ParathymOSin | AAEEEDEADPKR |
| P20962 | ParathymOSin | RAAEEEDEADPK |
| P20962 | ParathymOSin | AAEEEDEADPK |
| Q9BRP8 | Partner of Y14 and mago | AAPTAASDQPDSAATTEK |
| Q9BRP8 | Partner of Y14 and mago | ALEEELEDLELGL |
| O00151 | PDZ and LIM domain protein 1 | QSTSFLVLQEILESEEK |
| O00151 | PDZ and LIM domain protein 1 | AALANLCIGDVITAIDGENTSNMTHLEAQNR |
| O00151 | PDZ and LIM domain protein 1 | QELNEPPKQSTSFLVLQEILESEEK |
| O00151 | PDZ and LIM domain protein 1 | GCTDNLTLTVAR |
| O00151 | PDZ and LIM domain protein 1 | SAMPFTASPASSTTAR |
| Q96HC4 | PDZ and LIM domain protein 5 | GCTGSLNMTLQR |
| Q96HC4 | PDZ and LIM domain protein 5 | IGDVVLSIDGINAQGMTHLEAQNK |
| Q96HC4 | PDZ and LIM domain protein 5 | ASAAPKPEPVPVQKGEPK |
| P19021 | Peptidyl-glyCine alpha-amidating monooxygenase | IPVDEEAFVIDFKPR |
| P19021 | Peptidyl-glyCine alpha-amidating monooxygenase | ANILYAWAR |
| P19021 | Peptidyl-glyCine alpha-amidating monooxygenase | QSDTYFCMSMR |
| P19021 | Peptidyl-glyCine alpha-amidating monooxygenase | QSPQLPQAFYPVGHPVDVSFGDLLAAR |
| P23284 | Peptidyl-prolyl Cis-trans isomerase B | DTNGSQFFITTVK |
| P23284 | Peptidyl-prolyl Cis-trans isomerase B | VLEGMEVVR |
| P23284 | Peptidyl-prolyl Cis-trans isomerase B | TVDNFVALATGEK |
| P23284 | Peptidyl-prolyl Cis-trans isomerase B | IGDEDVGRVIFGLFGK |
| P23284 | Peptidyl-prolyl Cis-trans isomerase B | HYGPGWVSMANAGK |
| P23284 | Peptidyl-prolyl Cis-trans isomerase B | VIFGLFGK |
| P23284 | Peptidyl-prolyl Cis-trans isomerase B | IGDEDVGR |
| P23284 | Peptidyl-prolyl Cis-trans isomerase B | DKPLKDVIIADCGK |
| P23284 | Peptidyl-prolyl Cis-trans isomerase B | DFMIQGGDFTR |
| P23284 | Peptidyl-prolyl Cis-trans isomerase B | IEVEKPFAIAKE |
| P62942 | Peptidyl-prolyl Cis-trans isomerase FKBP1A | RGQTCVVHYTGMLEDGK |
| P62942 | Peptidyl-prolyl Cis-trans isomerase FKBP1A | GVQVETISPGDGR |
| P62942 | Peptidyl-prolyl Cis-trans isomerase FKBP1A | LTISPDYAYGATGHPGIIPPHATLVFDVELLKLE |
| P62942 | Peptidyl-prolyl Cis-trans isomerase FKBP1A | GWEEGVAQMSVGQR |
| Q00688 | Peptidyl-prolyl Cis-trans isomerase FKBP3 | FKGTESISK |
| Q00688 | Peptidyl-prolyl Cis-trans isomerase FKBP3 | FLQEHGSDSFLAEHK |
| Q02790 | Peptidyl-prolyl Cis-trans isomerase FKBP4 | LQAFSAAIESCNK |
| Q02790 | Peptidyl-prolyl Cis-trans isomerase FKBP4 | SNTAGSQSQVETEA |
| Q02790 | Peptidyl-prolyl Cis-trans isomerase FKBP4 | AEASSGDHPTDTEMKEEQK |
| Q02790 | Peptidyl-prolyl Cis-trans isomerase FKBP4 | LASHLNLAMCHLK |
| Q02790 | Peptidyl-prolyl Cis-trans isomerase FKBP4 | RGEAHLAVNDFELAR |
| Q9Y237 | Peptidyl-prolyl Cis-trans isomerase NIMA-interaCting 4 | FNEVAAQYSEDK |
| Q9Y237 | Peptidyl-prolyl Cis-trans isomerase NIMA-interaCting 4 | GSMVGPFQEAAFALPVSGMDKPVFTDPPVK |
| Q9H2H8 | Peptidyl-prolyl Cis-trans isomerase-like 3 | VIDGLETLDELEKLPVNEK |
| O95613 | PeriCentrin | LCVALK |
| O60664 | Perilipin-3 | SVVTGGVQSVMGSR |
| O60664 | Perilipin-3 | VSGAQEMVSSAK |
| O60664 | Perilipin-3 | IATSLDGFDVASVQQQR |
| O60664 | Perilipin-3 | LGQMVLSGVDTVLGK |
| O60664 | Perilipin-3 | GLDKLEENLPILQQPTEK |
| O60664 | Perilipin-3 | SEEWADNHLPLTDAELAR |
| O60664 | Perilipin-3 | DIAQQLQATCTSLGSSIQGLPTNVKDQVQQAR |
| O60664 | Perilipin-3 | DIAQQLQATCTSLGSSIQGLPTNVK |
| O60664 | Perilipin-3 | TLTAAAVSGAQPILSK |
| P32119 | Peroxiredoxin-2 | ATAVVDGAFK |
| P32119 | Peroxiredoxin-2 | EGGLGPLNIPLLADVTR |
| P32119 | Peroxiredoxin-2 | GKYVVLFFYPLDFTFVCPTEIIAFSNR |
| P32119 | Peroxiredoxin-2 | LSEDYGVLKTDEGIAYR |
| P32119 | Peroxiredoxin-2 | LGCEVLGVSVDSQFTHLAWINTPRK |
| P32119 | Peroxiredoxin-2 | TDEGIAYR |
| P32119 | Peroxiredoxin-2 | KEGGLGPLNIPLLADVTR |
| P32119 | Peroxiredoxin-2 | LVQAFQYTDEHGEVCPAGWKPGSDTIKPNVDDSK |
| P32119 | Peroxiredoxin-2 | ATAVVDGAFKEVK |
| P32119 | Peroxiredoxin-2 | LSEDYGVLK |
| P32119 | Peroxiredoxin-2 | EGGLGPLNIPLLADVTRR |
| P32119 | Peroxiredoxin-2 | LGCEVLGVSVDSQFTHLAWINTPR |
| P32119 | Peroxiredoxin-2 | SVDEALR |
| P32119 | Peroxiredoxin-2 | RLSEDYGVLK |
| Q8WW12 | PEST proteolytiC signal-Containing nuClear protein | RSAEEEAADLPTKPTK |
| P48739 | PhOSphatidylinOSitol transfer protein beta isoform | SQVEPADYKADEDPALFQSVK |
| O95394 | PhOSphoaCetylgluCOSamine mutase | IATLISSFLK |
| O95394 | PhOSphoaCetylgluCOSamine mutase | LVDPLGEMLAPSWEEHATCLANAEEQDMQR |
| O95394 | PhOSphoaCetylgluCOSamine mutase | QASCSGDEYR |
| P36871 | PhOSphogluComutase-1 | ADNFEYSDPVDGSISR |
| P36871 | PhOSphogluComutase-1 | FNISNGGPAPEAITDK |
| P36871 | PhOSphogluComutase-1 | LSGTGSAGATIR |
| P36871 | PhOSphogluComutase-1 | INQDPQVMLAPLISIALK |
| P36871 | PhOSphogluComutase-1 | FKPFTVEIVDSVEAYATMLR |
| P36871 | PhOSphogluComutase-1 | HGFFVNPSDSVAVIAANIFSIPYFQQTGVR |
| P36871 | PhOSphogluComutase-1 | TIEEYAVCPDLK |
| P36871 | PhOSphogluComutase-1 | TQAYQDQKPGTSGLR |
| P36871 | PhOSphogluComutase-1 | IALYETPTGWK |
| P36871 | PhOSphogluComutase-1 | VDLGVLGK |
| P36871 | PhOSphogluComutase-1 | IFQISK |
| P36871 | PhOSphogluComutase-1 | VFQSSANYAENFIQSIISTVEPAQR |
| P36871 | PhOSphogluComutase-1 | SIFDFSALK |
| P36871 | PhOSphogluComutase-1 | LIFTDGSR |
| P36871 | PhOSphogluComutase-1 | FFGNLMDASK |
| Q96G03 | PhOSphogluComutase-2 | DGVSAAVISAELASFLATK |
| Q96G03 | PhOSphogluComutase-2 | LAATTFISQGIPVYLFSDITPTPFVPFTVSHLK |
| Q96G03 | PhOSphogluComutase-2 | MNDLTIIQTTQGFCR |
| Q96G03 | PhOSphogluComutase-2 | NSLTLEAVKR |
| P00558 | PhOSphoglyCerate kinase 1 | ACANPAAGSVILLENLR |
| P00558 | PhOSphoglyCerate kinase 1 | GCITIIGGGDTATCCAK |
| P00558 | PhOSphoglyCerate kinase 1 | VSHVSTGGGASLELLEGK |
| P00558 | PhOSphoglyCerate kinase 1 | QIVWNGPVGVFEWEAFAR |
| P00558 | PhOSphoglyCerate kinase 1 | ITLPVDFVTADKFDENAK |
| P00558 | PhOSphoglyCerate kinase 1 | ALESPERPFLAILGGAK |
| P00558 | PhOSphoglyCerate kinase 1 | AHSSMVGVNLPQK |
| P00558 | PhOSphoglyCerate kinase 1 | VLNNMEIGTSLFDEEGAK |
| P00558 | PhOSphoglyCerate kinase 1 | AGGFLMKK |
| P00558 | PhOSphoglyCerate kinase 1 | VLPGVDALSNI |
| P00558 | PhOSphoglyCerate kinase 1 | TGQATVASGIPAGWMGLDCGPESSK |
| P00558 | PhOSphoglyCerate kinase 1 | LGDVYVNDAFGTAHR |
| P00558 | PhOSphoglyCerate kinase 1 | DCVGPEVEK |
| P00558 | PhOSphoglyCerate kinase 1 | LTLDKLDVK |
| P00558 | PhOSphoglyCerate kinase 1 | DVLFLK |
| P00558 | PhOSphoglyCerate kinase 1 | WNTEDKVSHVSTGGGASLELLEGK |
| P00558 | PhOSphoglyCerate kinase 1 | AEPAKIEAFR |
| P00558 | PhOSphoglyCerate kinase 1 | VADKIQLINNMLDK |
| P00558 | PhOSphoglyCerate kinase 1 | NNQITNNQR |
| P00558 | PhOSphoglyCerate kinase 1 | TGQATVASGIPAGWMGLDCGPESSKK |
| P00558 | PhOSphoglyCerate kinase 1 | YAEAVTR |
| P00558 | PhOSphoglyCerate kinase 1 | FHVEEEGKGK |
| P00558 | PhOSphoglyCerate kinase 1 | SLLGKDVLFLK |
| P00558 | PhOSphoglyCerate kinase 1 | KYAEAVTR |
| P00558 | PhOSphoglyCerate kinase 1 | VDFNVPMK |
| P00558 | PhOSphoglyCerate kinase 1 | FHVEEEGK |
| P00558 | PhOSphoglyCerate kinase 1 | VNEMIIGGGMAFTFLK |
| P00558 | PhOSphoglyCerate kinase 1 | SVVLMSHLGRPDGVPMPDKYSLEPVAVELK |
| P00558 | PhOSphoglyCerate kinase 1 | FCLDNGAK |
| P18669 | PhOSphoglyCerate mutase 1 | FSGWYDADLSPAGHEEAK |
| P18669 | PhOSphoglyCerate mutase 1 | YADLTEDQLPSCESLK |
| P18669 | PhOSphoglyCerate mutase 1 | ALPFWNEEIVPQIK |
| P18669 | PhOSphoglyCerate mutase 1 | AMEAVAAQGK |
| P18669 | PhOSphoglyCerate mutase 1 | HGESAWNLENR |
| P18669 | PhOSphoglyCerate mutase 1 | LVLIRHGESAWNLENR |
| P18669 | PhOSphoglyCerate mutase 1 | HLEGLSEEAIMELNLPTGIPIVYELDK |
| P18669 | PhOSphoglyCerate mutase 1 | NLKPIKPMQFLGDEETVRK |
| P18669 | PhOSphoglyCerate mutase 1 | RVLIAAHGNSLR |
| P18669 | PhOSphoglyCerate mutase 1 | HYGGLTGLNKAETAAK |
| P18669 | PhOSphoglyCerate mutase 1 | YADLTEDQLPSCESLKDTIAR |
| P18669 | PhOSphoglyCerate mutase 1 | KAMEAVAAQGK |
| P18669 | PhOSphoglyCerate mutase 1 | HYGGLTGLNK |
| P18669 | PhOSphoglyCerate mutase 1 | VLIAAHGNSLR |
| P18669 | PhOSphoglyCerate mutase 1 | RSYDVPPPPMEPDHPFYSNISK |
| P18669 | PhOSphoglyCerate mutase 1 | NLKPIKPMQFLGDEETVR |
| P36969 | PhOSpholipid hydroperoxide glutathione peroxidase, mitoChondrial | YGPMEEPLVIEK |
| P55058 | PhOSpholipid transfer protein | AVEPQLQEEER |
| P55058 | PhOSpholipid transfer protein | FLEQELETITIPDLR |
| P55058 | PhOSpholipid transfer protein | TGLELSRDPAGR |
| O15067 | PhOSphoribOSylformylglyCinamidine synthase | LGGTALAQCFSQLGEHPPDLDLPENLVR |
| O15067 | PhOSphoribOSylformylglyCinamidine synthase | SVGGLVAQQQCVGPLQTPLADVAVVALSHEELIGAATALGEQPVK |
| Q9Y617 | PhOSphOSerine aminotransferase | ASLYNAVTIEDVQK |
| Q9Y617 | PhOSphOSerine aminotransferase | FGVIFAGAQK |
| Q9Y617 | PhOSphOSerine aminotransferase | IINNTENLVR |
| Q9Y617 | PhOSphOSerine aminotransferase | ALELNMLSLK |
| Q9Y617 | PhOSphOSerine aminotransferase | SQTIYEIIDNSQGFYVCPVEPQNR |
| Q9Y617 | PhOSphOSerine aminotransferase | GAVLVCDMSSNFLSKPVDVSK |
| Q9Y617 | PhOSphOSerine aminotransferase | VIFLQGGGCGQFSAVPLNLIGLK |
| P36955 | Pigment epithelium-derived faCtor | TSLEDFYLDEER |
| P36955 | Pigment epithelium-derived faCtor | YGLDSDLSCK |
| P36955 | Pigment epithelium-derived faCtor | LQSLFDSPDFSK |
| P36955 | Pigment epithelium-derived faCtor | ALYYDLISSPDIHGTYK |
| P36955 | Pigment epithelium-derived faCtor | TVQAVLTVPK |
| P36955 | Pigment epithelium-derived faCtor | SSTSPTTNVLLSPLSVATALSALSLGAEQR |
| P36955 | Pigment epithelium-derived faCtor | LAAAVSNFGYDLYR |
| P36955 | Pigment epithelium-derived faCtor | LTQVEHR |
| P36955 | Pigment epithelium-derived faCtor | LDLQEINNWVQAQMK |
| O00625 | Pirin | NLDPFLLFDEFK |
| O00625 | Pirin | MNPGDLQWMTAGR |
| Q8WU39 | Plasma Cell-induCed resident endoplasmiC retiCulum protein | AVAYQMWQNLAK |
| P03952 | Plasma kallikrein | IAYGTQGSSGYSLR |
| P03952 | Plasma kallikrein | GVNVCQETCTK |
| P03952 | Plasma kallikrein | LCNTGDNSVCTTK |
| P03952 | Plasma kallikrein | VSSVEECQKR |
| P03952 | Plasma kallikrein | LVGITSWGEGCAR |
| P03952 | Plasma kallikrein | TGAVSGHSLK |
| P03952 | Plasma kallikrein | LSMDGSPTR |
| P05155 | Plasma protease C1 inhibitor | LVLLNAIYLSAK |
| P05155 | Plasma protease C1 inhibitor | TNLESILSYPK |
| P05155 | Plasma protease C1 inhibitor | VTTSQDMLSIMEK |
| P05155 | Plasma protease C1 inhibitor | HRLEDMEQALSPSVFK |
| P05155 | Plasma protease C1 inhibitor | DFTCVHQALK |
| P05155 | Plasma protease C1 inhibitor | LLDSLPSDTR |
| P05155 | Plasma protease C1 inhibitor | LYHAFSAMK |
| P05155 | Plasma protease C1 inhibitor | TRMEPFHFK |
| P05155 | Plasma protease C1 inhibitor | LLDSLPSDTRLVLLNAIYLSAK |
| P05155 | Plasma protease C1 inhibitor | FQPTLLTLPR |
| P05155 | Plasma protease C1 inhibitor | TLYSSSPR |
| P05155 | Plasma protease C1 inhibitor | TLLVFEVQQPFLFVLWDQQHK |
| P05155 | Plasma protease C1 inhibitor | LEDMEQALSPSVFK |
| P05155 | Plasma protease C1 inhibitor | IKVTTSQDMLSIMEK |
| P05155 | Plasma protease C1 inhibitor | GVTSVSQIFHSPDLAIR |
| P05155 | Plasma protease C1 inhibitor | AIMEKLEMSK |
| P05155 | Plasma protease C1 inhibitor | KYPVAHFIDQTLK |
| P05155 | Plasma protease C1 inhibitor | NSVIKVPMMNSK |
| P05155 | Plasma protease C1 inhibitor | FPVFMGR |
| P05154 | Plasma serine protease inhibitor | NLDSNAVVIMVNYIFFK |
| P05154 | Plasma serine protease inhibitor | MQQVENGLSEK |
| P05154 | Plasma serine protease inhibitor | IVDLIK |
| P05154 | Plasma serine protease inhibitor | AAAATGTIFTFR |
| P05154 | Plasma serine protease inhibitor | FSIEGSYQLEK |
| P05154 | Plasma serine protease inhibitor | AVVEVDESGTR |
| P05154 | Plasma serine protease inhibitor | TLYLADTFPTNFR |
| P05154 | Plasma serine protease inhibitor | DFTFDLYR |
| P05154 | Plasma serine protease inhibitor | QLELYLPK |
| P05154 | Plasma serine protease inhibitor | MQILEGLGLNLQK |
| Q8NC51 | Plasminogen aCtivator inhibitor 1 RNA-binding protein | SAAQAAAQTNSNAAGK |
| Q8NC51 | Plasminogen aCtivator inhibitor 1 RNA-binding protein | EAGGGGVGGPGAK |
| P00747 | Plasminogen | VQSTELCAGHLAGGTDSCQGDSGGPLVCFEK |
| P00747 | Plasminogen | RATTVTGTPCQDWAAQEPHR |
| P00747 | Plasminogen | QLGAGSIEECAAKCEEDEEFTCR |
| P00747 | Plasminogen | CTTPPPSSGPTYQCLK |
| P00747 | Plasminogen | LYDYCDVPQCAAPSFDCGKPQVEPK |
| P00747 | Plasminogen | FSPATHPSEGLEENYCRNPDNDPQGPWCYTTDPEK |
| P00747 | Plasminogen | NPDGDVGGPWCYTTNPR |
| P00747 | Plasminogen | KLYDYCDVPQCAAPSFDCGKPQVEPK |
| P00747 | Plasminogen | NYCRNPDGDVGGPWCYTTNPR |
| P00747 | Plasminogen | NLDENYCRNPDGK |
| P00747 | Plasminogen | HSIFTPETNPR |
| P00747 | Plasminogen | LSSPAVITDK |
| P00747 | Plasminogen | VILGAHQEVNLEPHVQEIEVSR |
| P00747 | Plasminogen | NPDADKGPWCFTTDPSVR |
| P00747 | Plasminogen | YDYCDILECEEECMHCSGENYDGK |
| P00747 | Plasminogen | SSIIIR |
| Q14651 | Plastin-1 | SISTSLPVLDLIDAIAPNAVR |
| P13796 | Plastin-2 | ISTSLPVLDLIDAIQPGSINYDLLK |
| P13796 | Plastin-2 | EGICAIGGTSEQSSVGTQHSYSEEEK |
| P13796 | Plastin-2 | QFVTATDVVR |
| P13796 | Plastin-2 | KIENCNYAVELGK |
| P13796 | Plastin-2 | IKVPVDWNR |
| P13796 | Plastin-2 | ISTSLPVLDLIDAIQPGSINYDLLKTENLNDDEK |
| P13796 | Plastin-2 | FSLVGIGGQDLNEGNR |
| P13796 | Plastin-2 | YTLNILEEIGGGQK |
| P13796 | Plastin-2 | HVIPMNPNTNDLFNAVGDGIVLCK |
| P13796 | Plastin-2 | GSVSDEEMMELR |
| P13796 | Plastin-2 | NWMNSLGVNPR |
| P13796 | Plastin-2 | VYALPEDLVEVNPK |
| P13796 | Plastin-2 | VNDDIIVNWVNETLR |
| P13796 | Plastin-2 | AECMLQQAER |
| P13796 | Plastin-2 | TENLNDDEK |
| P13797 | Plastin-3 | SGNLTEDDKHNNAK |
| P13797 | Plastin-3 | IKVPVDWSK |
| P13797 | Plastin-3 | EGICALGGTSELSSEGTQHSYSEEEK |
| P13797 | Plastin-3 | YAVSMAR |
| P13797 | Plastin-3 | VYALPEDLVEVKPK |
| P13797 | Plastin-3 | QFVTPADVVSGNPK |
| P43034 | Platelet-aCtivating faCtor aCetylhydrolase IB subunit alpha | MVRPNQDGTLIASCSNDQTVR |
| P43034 | Platelet-aCtivating faCtor aCetylhydrolase IB subunit alpha | LWDFQGFECIR |
| P43034 | Platelet-aCtivating faCtor aCetylhydrolase IB subunit alpha | TAPYVVTGSVDQTVK |
| P68402 | Platelet-aCtivating faCtor aCetylhydrolase IB subunit beta | DKEPDVLFVGDSMVQLMQQYEIWR |
| P68402 | Platelet-aCtivating faCtor aCetylhydrolase IB subunit beta | ELFSPLHALNFGIGGDTTR |
| P68402 | Platelet-aCtivating faCtor aCetylhydrolase IB subunit beta | VIVVWVGTNNHENTAEEVAGGIEAIVQLINTR |
| P68402 | Platelet-aCtivating faCtor aCetylhydrolase IB subunit beta | VNQILK |
| P68402 | Platelet-aCtivating faCtor aCetylhydrolase IB subunit beta | FVLDCK |
| P68402 | Platelet-aCtivating faCtor aCetylhydrolase IB subunit beta | LKNGELENIKPK |
| Q15102 | Platelet-aCtivating faCtor aCetylhydrolase IB subunit gamma | VVVLGLLPR |
| O00592 | PodoCalyxin | LASVPGSQTVVVK |
| O00592 | PodoCalyxin | CEDLETQTQSEK |
| O00592 | PodoCalyxin | LPAKDVYER |
| O00592 | PodoCalyxin | LISLICR |
| O00592 | PodoCalyxin | ATFNPAQDK |
| Q15365 | Poly(rC)-binding protein 1 | LVVPATQCGSLIGK |
| Q15365 | Poly(rC)-binding protein 1 | AFAMIIDKLEEDINSSMTNSTAASRPPVTLR |
| Q15365 | Poly(rC)-binding protein 1 | IITLTGPTNAIFK |
| Q15365 | Poly(rC)-binding protein 1 | VMTIPYQPMPASSPVICAGGQDR |
| Q15365 | Poly(rC)-binding protein 1 | AITIAGVPQSVTECVK |
| Q15365 | Poly(rC)-binding protein 1 | QGANINEIR |
| Q15365 | Poly(rC)-binding protein 1 | QVTITGSAASISLAQYLINAR |
| Q15366 | Poly(rC)-binding protein 2 | AITIAGIPQSIIECVK |
| Q15366 | Poly(rC)-binding protein 2 | LVVPASQCGSLIGK |
| Q15366 | Poly(rC)-binding protein 2 | INISEGNCPER |
| Q15366 | Poly(rC)-binding protein 2 | IITLAGPTNAIFK |
| P11940 | Polyadenylate-binding protein 1 | ALYDTFSAFGNILSCK |
| P11940 | Polyadenylate-binding protein 1 | GFGFVCFSSPEEATK |
| P11940 | Polyadenylate-binding protein 1 | FSPAGPILSIR |
| P11940 | Polyadenylate-binding protein 1 | FGPALSVK |
| P26599 | Polypyrimidine traCt-binding protein 1 | IIVENLFYPVTLDVLHQIFSK |
| P26599 | Polypyrimidine traCt-binding protein 1 | KLPIDVTEGEVISLGLPFGK |
| P26599 | Polypyrimidine traCt-binding protein 1 | LSLDGQNIYNACCTLR |
| P26599 | Polypyrimidine traCt-binding protein 1 | EGQEDQGLTK |
| Q9UKA9 | Polypyrimidine traCt-binding protein 2 | VTNILMLK |
| P0CG47 | Polyubiquitin-B | TITLEVEPSDTIENVK |
| P0CG47 | Polyubiquitin-B | IQDKEGIPPDQQR |
| P0CG47 | Polyubiquitin-B | ESTLHLVLR |
| P0CG47 | Polyubiquitin-B | TLSDYNIQK |
| P0CG48 | Polyubiquitin-C | QLEDGRTLSDYNIQK |
| P0CG48 | Polyubiquitin-C | ESTLHLVLR |
| P0CG48 | Polyubiquitin-C | TITLEVEPSDTIENVK |
| P0CG48 | Polyubiquitin-C | TLSDYNIQK |
| P08397 | Porphobilinogen deaminase | GPQLAAQNLGISLANLLLSK |
| Q9UHV9 | Prefoldin subunit 2 | IIETLTQQLQAK |
| Q99471 | Prefoldin subunit 5 | NQLDQEVEFLSTSIAQLK |
| P20742 | PregnanCy zone protein | GSFALSFPVESDVAPIAR |
| P20742 | PregnanCy zone protein | VVVQTESGGR |
| P20742 | PregnanCy zone protein | IQHPFTVEEFVLPK |
| P07602 | ProaCtivator polypeptide | EIVDSYLPVILDIIK |
| P07602 | ProaCtivator polypeptide | LGPGMADICK |
| P07602 | ProaCtivator polypeptide | QEILAALEK |
| Q8NDH3 | Probable aminopeptidase NPEPL1 | DLGADIILDMATLTGAQGIATGK |
| Q92841 | Probable ATP-dependent RNA heliCase DDX17 | SSQSSSQQFSGIGR |
| Q9H6S0 | Probable ATP-dependent RNA heliCase YTHDC2 | IDEEVK |
| Q9UJV3 | Probable E3 ubiquitin-protein ligase MID2 | QMIAVKIK |
| P09668 | Pro-Cathepsin H | NMCGLAACASYPIPLV |
| P09668 | Pro-Cathepsin H | NQGACGSCWTFSTTGALESAIAIATGK |
| P07737 | Profilin-1 | DSLLQDGEFSMDLR |
| P07737 | Profilin-1 | DRSSFYVNGLTLGGQK |
| P07737 | Profilin-1 | TLVLLMGK |
| P07737 | Profilin-1 | TFVNITPAEVGVLVGK |
| P07737 | Profilin-1 | STGGAPTFNVTVTKTDK |
| P07737 | Profilin-1 | CSVIRDSLLQDGEFSMDLR |
| P07737 | Profilin-1 | EGVHGGLINKK |
| P07737 | Profilin-1 | DSPSVWAAVPGK |
| P07737 | Profilin-1 | TFVNITPAEVGVLVGKDR |
| P07737 | Profilin-1 | SSFYVNGLTLGGQK |
| P07737 | Profilin-1 | CYEMASHLR |
| P07737 | Profilin-1 | STGGAPTFNVTVTK |
| P35080 | Profilin-2 | SQGGEPTYNVAVGR |
| Q8WUM4 | Programmed Cell death 6-interaCting protein | NLATAYDNFVELVANLK |
| Q8WUM4 | Programmed Cell death 6-interaCting protein | LANQAADYFGDAFK |
| Q8WUM4 | Programmed Cell death 6-interaCting protein | FYNELTEILVR |
| Q8WUM4 | Programmed Cell death 6-interaCting protein | SCVLFNCAALASQIAAEQNLDNDEGLK |
| Q8WUM4 | Programmed Cell death 6-interaCting protein | LALASLGYEK |
| Q8WUM4 | Programmed Cell death 6-interaCting protein | FLTALAQDGVINEEALSVTELDRVYGGLTTK |
| Q8WUM4 | Programmed Cell death 6-interaCting protein | LQHAAELIK |
| Q8WUM4 | Programmed Cell death 6-interaCting protein | NIQVSHQEFSK |
| Q9BUL8 | Programmed Cell death protein 10 | VNLSAAQTLR |
| O14737 | Programmed Cell death protein 5 | HGDPGDAAQQEAK |
| O14737 | Programmed Cell death protein 5 | AVENYLIQMAR |
| O75340 | Programmed Cell death protein 6 | AGVNFSEFTGVWK |
| P12273 | ProlaCtin-induCible protein | TYLISSIPLQGAFNYK |
| P12273 | ProlaCtin-induCible protein | YTACLCDDNPK |
| Q9UQ80 | Proliferation-assoCiated protein 2G4 | AFFSEVER |
| Q9UQ80 | Proliferation-assoCiated protein 2G4 | ALLQSSASR |
| Q9UQ80 | Proliferation-assoCiated protein 2G4 | LVKPGNQNTQVTEAWNK |
| O94903 | Proline synthase Co-transCribed baCterial homolog protein | LMAVPNLFMLETVDSVK |
| P48147 | Prolyl endopeptidase | VFLDPNILSDDGTVALR |
| P48147 | Prolyl endopeptidase | VLYVQDSLEGEAR |
| P48147 | Prolyl endopeptidase | ITVPFLEQCPIR |
| Q15185 | PrOStaglandin E synthase 3 | KGESGQSWPR |
| Q15185 | PrOStaglandin E synthase 3 | SKLTFSCLGGSDNFK |
| Q15185 | PrOStaglandin E synthase 3 | HLNEIDLFHCIDPNDSK |
| Q15185 | PrOStaglandin E synthase 3 | LTFSCLGGSDNFK |
| Q14914 | PrOStaglandin reduCtase 1 | GGETVMVNAAAGAVGSVVGQIAK |
| Q14914 | PrOStaglandin reduCtase 1 | ASPDGYDCYFDNVGGEFSNTVIGQMK |
| Q14914 | PrOStaglandin reduCtase 1 | TGPLPPGPPPEIVIYQELR |
| Q14914 | PrOStaglandin reduCtase 1 | EYIIEGFENMPAAFMGMLK |
| Q14914 | PrOStaglandin reduCtase 1 | VVGAVGSDEK |
| Q14914 | PrOStaglandin reduCtase 1 | MEAFVVYR |
| P41222 | PrOStaglandin-H2 D-isomerase | AQGFTEDTIVFLPQTDK |
| P41222 | PrOStaglandin-H2 D-isomerase | TMLLQPAGSLGSYSYR |
| Q16651 | PrOStasin | NRPGVYTLASSYASWIQSK |
| Q16651 | PrOStasin | LGAHQLDSYSEDAK |
| Q16651 | PrOStasin | DIIPHPSYLQEGSQGDIALLQLSRPITFSR |
| Q16651 | PrOStasin | VTELQPR |
| Q16186 | Proteasomal ubiquitin reCeptor ADRM1 | SQSAAVTPSSTTSSTR |
| Q06323 | Proteasome aCtivator Complex subunit 1 | NAYAVLYDIILK |
| Q06323 | Proteasome aCtivator Complex subunit 1 | EPALNEANLSNLK |
| Q06323 | Proteasome aCtivator Complex subunit 1 | IEDGNNFGVAVQEK |
| Q9UL46 | Proteasome aCtivator Complex subunit 2 | VLSLLALVKPEVWTLK |
| Q9UL46 | Proteasome aCtivator Complex subunit 2 | IIYLNQLLQEDSLNVADLTSLR |
| P25786 | Proteasome subunit alpha type-1 | ALRETLPAEQDLTTK |
| P25786 | Proteasome subunit alpha type-1 | NQYDNDVTVWSPQGR |
| P25786 | Proteasome subunit alpha type-1 | QGSATVGLK |
| P25786 | Proteasome subunit alpha type-1 | AQSELAAHQKK |
| P25786 | Proteasome subunit alpha type-1 | AQSELAAHQK |
| P25786 | Proteasome subunit alpha type-1 | AMSIGAR |
| P25786 | Proteasome subunit alpha type-1 | IHQIEYAMEAVK |
| P25786 | Proteasome subunit alpha type-1 | LVSLIGSK |
| P25786 | Proteasome subunit alpha type-1 | RAQSELAAHQK |
| P25786 | Proteasome subunit alpha type-1 | LLCNFMR |
| P25787 | Proteasome subunit alpha type-2 | GYSFSLTTFSPSGK |
| P25787 | Proteasome subunit alpha type-2 | HIGLVYSGMGPDYR |
| P25787 | Proteasome subunit alpha type-2 | LVQIEYALAAVAGGAPSVGIK |
| P25787 | Proteasome subunit alpha type-2 | ESFEGQMTEDNIEVGICNEAGFRR |
| P25787 | Proteasome subunit alpha type-2 | RYNEDLELEDAIHTAILTLK |
| P25787 | Proteasome subunit alpha type-2 | YNEDLELEDAIHTAILTLK |
| P25787 | Proteasome subunit alpha type-2 | SVHKVEPITK |
| P25787 | Proteasome subunit alpha type-2 | KLAQQYYLVYQEPIPTAQLVQR |
| P25788 | Proteasome subunit alpha type-3 | AVENSSTAIGIR |
| P25788 | Proteasome subunit alpha type-3 | VFQVEYAMK |
| P25788 | Proteasome subunit alpha type-3 | SLADIAREEASNFR |
| P25788 | Proteasome subunit alpha type-3 | SNFGYNIPLK |
| P25788 | Proteasome subunit alpha type-3 | LYEEGSNKR |
| P25789 | Proteasome subunit alpha type-4 | LNEDMACSVAGITSDANVLTNELR |
| P25789 | Proteasome subunit alpha type-4 | ATCIGNNSAAAVSMLK |
| P25789 | Proteasome subunit alpha type-4 | LLDEVFFSEK |
| P25789 | Proteasome subunit alpha type-4 | VEIATLTR |
| P25789 | Proteasome subunit alpha type-4 | SALALAIK |
| P25789 | Proteasome subunit alpha type-4 | QAYTQFGGK |
| P25789 | Proteasome subunit alpha type-4 | YLLQYQEPIPCEQLVTALCDIK |
| P25789 | Proteasome subunit alpha type-4 | HYGFQLYQSDPSGNYGGWK |
| P25789 | Proteasome subunit alpha type-4 | IYKLNEDMACSVAGITSDANVLTNELR |
| P25789 | Proteasome subunit alpha type-4 | LSAEKVEIATLTR |
| P28066 | Proteasome subunit alpha type-5 | LFQVEYAIEAIK |
| P28066 | Proteasome subunit alpha type-5 | AIGSASEGAQSSLQEVYHK |
| P28066 | Proteasome subunit alpha type-5 | GPQLFHMDPSGTFVQCDAR |
| P28066 | Proteasome subunit alpha type-5 | SSLIILK |
| P28066 | Proteasome subunit alpha type-5 | ITSPLMEPSSIEK |
| P28066 | Proteasome subunit alpha type-5 | RITSPLMEPSSIEK |
| P28066 | Proteasome subunit alpha type-5 | SEYDRGVNTFSPEGR |
| P28066 | Proteasome subunit alpha type-5 | GVNTFSPEGR |
| P60900 | Proteasome subunit alpha type-6 | AINQGGLTSVAVR |
| P60900 | Proteasome subunit alpha type-6 | ITENIGCVMTGMTADSR |
| P60900 | Proteasome subunit alpha type-6 | LYQVEYAFK |
| P60900 | Proteasome subunit alpha type-6 | DCAVIVTQK |
| P60900 | Proteasome subunit alpha type-6 | LLDSSTVTHLFK |
| P60900 | Proteasome subunit alpha type-6 | HITIFSPEGR |
| P60900 | Proteasome subunit alpha type-6 | GKDCAVIVTQK |
| P60900 | Proteasome subunit alpha type-6 | ILTEAEIDAHLVALAERD |
| P60900 | Proteasome subunit alpha type-6 | GSSAGFDR |
| O14818 | Proteasome subunit alpha type-7 | NYTDEAIETDDLTIK |
| O14818 | Proteasome subunit alpha type-7 | ICALDDNVCMAFAGLTADAR |
| O14818 | Proteasome subunit alpha type-7 | ALLEVVQSGGK |
| O14818 | Proteasome subunit alpha type-7 | LYQTDPSGTYHAWK |
| O14818 | Proteasome subunit alpha type-7 | LTVEDPVTVEYITR |
| O14818 | Proteasome subunit alpha type-7 | GRDIVVLGVEK |
| O14818 | Proteasome subunit alpha type-7 | ILNPEEIEK |
| O14818 | Proteasome subunit alpha type-7 | ARVECQSHR |
| O14818 | Proteasome subunit alpha type-7 | AITVFSPDGHLFQVEYAQEAVK |
| P20618 | Proteasome subunit beta type-1 | GAVYSFDPVGSYQR |
| P20618 | Proteasome subunit beta type-1 | AMTTGAIAAMLSTILYSR |
| P20618 | Proteasome subunit beta type-1 | DVFISAAERDVYTGDALR |
| P20618 | Proteasome subunit beta type-1 | AGGSASAMLQPLLDNQVGFK |
| P20618 | Proteasome subunit beta type-1 | TVIGCSGFHGDCLTLTK |
| P20618 | Proteasome subunit beta type-1 | NMQNVEHVPLSLDR |
| P40306 | Proteasome subunit beta type-10 | YHFVPGTTAVLTQTVKPLTLELVEETVQAMEVE |
| P40306 | Proteasome subunit beta type-10 | LPFTALGSGQDAALAVLEDR |
| P40306 | Proteasome subunit beta type-10 | ATNDSVVADK |
| P49721 | Proteasome subunit beta type-2 | NGYELSPTAAANFTR |
| P49721 | Proteasome subunit beta type-2 | FILNLPTFSVR |
| P49721 | Proteasome subunit beta type-2 | NLADCLR |
| P49720 | Proteasome subunit beta type-3 | LYIGLAGLATDVQTVAQR |
| P49720 | Proteasome subunit beta type-3 | FRLNLYELK |
| P49720 | Proteasome subunit beta type-3 | NCVAIAADRR |
| P49720 | Proteasome subunit beta type-3 | RFGPYYTEPVIAGLDPK |
| P49720 | Proteasome subunit beta type-3 | FGIQAQMVTTDFQK |
| P49720 | Proteasome subunit beta type-3 | FGPYYTEPVIAGLDPK |
| P49720 | Proteasome subunit beta type-3 | LNLYELK |
| P28070 | Proteasome subunit beta type-4 | VNNSTMLGASGDYADFQYLK |
| P28070 | Proteasome subunit beta type-4 | GVEIEGPLSTETNWDIAHMISGFE |
| P28070 | Proteasome subunit beta type-4 | QVLGQMVIDEELLGDGHSYSPR |
| P28070 | Proteasome subunit beta type-4 | FEGGVVIAADMLGSYGSLAR |
| P28070 | Proteasome subunit beta type-4 | FQIATVTEK |
| P28074 | Proteasome subunit beta type-5 | VIEINPYLLGTMAGGAADCSFWER |
| P28074 | Proteasome subunit beta type-5 | LLANMVYQYK |
| P28074 | Proteasome subunit beta type-5 | HGVIVAADSR |
| P28074 | Proteasome subunit beta type-5 | ATAGAYIASQTVK |
| P28072 | Proteasome subunit beta type-6 | TTTGSYIANR |
| P28072 | Proteasome subunit beta type-6 | QVLLGDQIPK |
| P28072 | Proteasome subunit beta type-6 | DGSSGGVIR |
| Q99436 | Proteasome subunit beta type-7 | ITPLEIEVLEETVQTMDTS |
| P28062 | Proteasome subunit beta type-8 | KGPGLYYVDEHGTR |
| P28062 | Proteasome subunit beta type-8 | FQHGVIAAVDSR |
| P28062 | Proteasome subunit beta type-8 | LLSNMMCQYR |
| P28062 | Proteasome subunit beta type-8 | VIEINPYLLGTMSGCAADCQYWER |
| P28062 | Proteasome subunit beta type-8 | ASAGSYISALR |
| P28065 | Proteasome subunit beta type-9 | FTTDAIALAMSR |
| P28065 | Proteasome subunit beta type-9 | VILGNELPK |
| P28065 | Proteasome subunit beta type-9 | VSAGEAVVNR |
| P02760 | Protein AMBP | AFIQLWAFDAVK |
| P02760 | Protein AMBP | GVCEETSGAYEK |
| P02760 | Protein AMBP | VVAQGVGIPEDSIFTMADRGECVPGEQEPEPILIPR |
| P02760 | Protein AMBP | WYNLAIGSTCPWLK |
| P02760 | Protein AMBP | EYCGVPGDGDEELLRFSN |
| P02760 | Protein AMBP | TVAACNLPIVR |
| P02760 | Protein AMBP | ECLQTCR |
| P02760 | Protein AMBP | CVLFPYGGCQGNGNK |
| P02760 | Protein AMBP | GVCEETSGAYEKTDTDGK |
| P02760 | Protein AMBP | EDSCQLGYSAGPCMGMTSR |
| Q99873 | Protein arginine N-methyltransferase 1 | DVAIKEPLVDVVDPK |
| Q9Y2B0 | Protein Canopy homolog 2 | INPDGSQSVVEVPYAR |
| Q9UKY7 | Protein CDV3 homolog | AASAAGAAGSAGGSSGAAGAAGGGAGAGTRPGDGGTASAGAAGPGAATK |
| O60888 | Protein CutA | TQSSLVPALTDFVR |
| O60888 | Protein CutA | SVHPYEVAEVIALPVEQGNFPYLQWVR |
| O60888 | Protein CutA | LAACVNLIPQITSIYEWK |
| P30101 | Protein disulfide-isomerase A3 | VVVAENFDEIVNNENK |
| P30101 | Protein disulfide-isomerase A3 | VDCTANTNTCNK |
| P30101 | Protein disulfide-isomerase A3 | MDATANDVPSPYEVR |
| P30101 | Protein disulfide-isomerase A3 | RLAPEYEAAATR |
| P30101 | Protein disulfide-isomerase A3 | FLQDYFDGNLK |
| P30101 | Protein disulfide-isomerase A3 | ELSDFISYLQR |
| P30101 | Protein disulfide-isomerase A3 | GFPTIYFSPANK |
| P30101 | Protein disulfide-isomerase A3 | LSKDPNIVIAK |
| P30101 | Protein disulfide-isomerase A3 | FISDKDASIVGFFDDSFSEAHSEFLK |
| P30101 | Protein disulfide-isomerase A3 | IFRDGEEAGAYDGPR |
| P30101 | Protein disulfide-isomerase A3 | TFSHELSDFGLESTAGEIPVVAIR |
| P30101 | Protein disulfide-isomerase A3 | FVMQEEFSR |
| P30101 | Protein disulfide-isomerase A3 | LNFAVASR |
| P30101 | Protein disulfide-isomerase A3 | EATNPPVIQEEKPK |
| P30101 | Protein disulfide-isomerase A3 | FIQENIFGICPHMTEDNK |
| P13667 | Protein disulfide-isomerase A4 | IDATSASVLASR |
| P13667 | Protein disulfide-isomerase A4 | VDATAETDLAK |
| P13667 | Protein disulfide-isomerase A4 | MDATANDVPSDR |
| Q15084 | Protein disulfide-isomerase A6 | GSFSEQGINEFLR |
| Q15084 | Protein disulfide-isomerase A6 | ALDLFSDNAPPPELLEIINEDIAKR |
| Q99497 | Protein DJ-1 | GPGTSFEFALAIVEALNGK |
| Q99497 | Protein DJ-1 | VTVAGLAGKDPVQCSR |
| Q99497 | Protein DJ-1 | GAEEMETVIPVDVMR |
| Q99497 | Protein DJ-1 | RALVILAK |
| Q99497 | Protein DJ-1 | EGPYDVVVLPGGNLGAQNLSESAAVK |
| Q99497 | Protein DJ-1 | DGLILTSRGPGTSFEFALAIVEALNGK |
| Q99497 | Protein DJ-1 | APLVLKD |
| Q99497 | Protein DJ-1 | GLIAAICAGPTALLAHEIGFGSK |
| Q99497 | Protein DJ-1 | DGLILTSR |
| Q99497 | Protein DJ-1 | EGPYDVVVLPGGNLGAQNLSESAAVKEILK |
| Q99497 | Protein DJ-1 | VTTHPLAK |
| Q99497 | Protein DJ-1 | EILKEQENR |
| Q99497 | Protein DJ-1 | ALVILAK |
| Q99497 | Protein DJ-1 | DVVICPDASLEDAKK |
| Q9C005 | Protein dpy-30 homolog | ERPPNPIEFLASYLLK |
| Q9C005 | Protein dpy-30 homolog | AYLDQTVVPILLQGLAVLAK |
| Q9NUQ9 | Protein FAM49B | DQPPNSVEGLLNALR |
| Q9NUQ9 | Protein FAM49B | DAEGILEDLQSYR |
| P49354 | Protein farnesyltransferase/geranylgeranyltransferase type-1 subunit alpha | DPSQELEFIADILNQDAK |
| Q8WZA0 | Protein LZIC | LTADDEAFLSANAGAILSQFEK |
| Q96C90 | Protein phOSphatase 1 regulatory subunit 14B | ELLVDCYKPTEAFISGLLDK |
| Q9UD71 | Protein phOSphatase 1 regulatory subunit 1B | LSEHSSPEEEASPHQR |
| Q15435 | Protein phOSphatase 1 regulatory subunit 7 | AIENIDTLTNLESLFLGK |
| Q9P258 | Protein RCC2 | TKDGQILPVPNVVVR |
| P23297 | Protein S100-A1 | ELLQTELSGFLDAQK |
| P23297 | Protein S100-A1 | ELLQTELSGFLDAQKDVDAVDK |
| P80511 | Protein S100-A12 | GHFDTLSKGELK |
| Q99584 | Protein S100-A13 | SLDVNQDSELK |
| Q99584 | Protein S100-A13 | DSLSVNEFK |
| Q99584 | Protein S100-A13 | FNEYWR |
| Q99584 | Protein S100-A13 | LIGELAK |
| P31151 | Protein S100-A7 | KIDFSEFLSLLGDIATDYHK |
| P06702 | Protein S100-A9 | QLSFEEFIMLMAR |
| P06702 | Protein S100-A9 | VIEHIMEDLDTNADK |
| P06702 | Protein S100-A9 | NIETIINTFHQYSVK |
| P06702 | Protein S100-A9 | LGHPDTLNQGEFK |
| P06702 | Protein S100-A9 | DLQNFLKK |
| P25815 | Protein S100-P | YSGSEGSTQTLTK |
| Q01105 | Protein SET | IPNFWVTTFVNHPQVSALLGEEDEEALHYLTR |
| Q01105 | Protein SET | IDFYFDENPYFENK |
| Q01105 | Protein SET | VEVTEFEDIK |
| Q01105 | Protein SET | EFHLNESGDPSSK |
| Q01105 | Protein SET | LRQPFFQK |
| Q8N114 | Protein shisa-5 | GLSLFPESCPDFCCGTCDDQYCCSDVLK |
| Q92734 | Protein TFG | LLSNDEVTIK |
| Q15437 | Protein transport protein SeC23B | MVVPLACLLTPLK |
| P00734 | Prothrombin | LAACLEGNCAEGLGTNYR |
| P00734 | Prothrombin | RQECSIPVCGQDQVTVAMTPR |
| P00734 | Prothrombin | YTACETAR |
| P00734 | Prothrombin | IVEGSDAEIGMSPWQVMLFR |
| P00734 | Prothrombin | TATSEYQTFFNPR |
| P00734 | Prothrombin | LAVTTHGLPCLAWASAQAK |
| P00734 | Prothrombin | SGIECQLWR |
| P00734 | Prothrombin | DKLAACLEGNCAEGLGTNYR |
| P00734 | Prothrombin | GQPSVLQVVNLPIVERPVCK |
| P00734 | Prothrombin | SEGSSVNLSPPLEQCVPDRGQQYQGR |
| P00734 | Prothrombin | NPDSSTTGPWCYTTDPTVR |
| P00734 | Prothrombin | SEGSSVNLSPPLEQCVPDR |
| P00734 | Prothrombin | SLEDKTER |
| P06454 | ProthymOSin alpha | AAEDDEDDDVDTK |
| P06454 | ProthymOSin alpha | AAEDDEDDDVDTKK |
| P06454 | ProthymOSin alpha | RAAEDDEDDDVDTK |
| Q08623 | Pseudouridine-5'-monophOSphatase | ALEAAQIIIDVLQLPMSKEELVEESQTK |
| P61457 | Pterin-4-alpha-Carbinolamine dehydratase | AFGFMTR |
| P61457 | Pterin-4-alpha-Carbinolamine dehydratase | LSAEERDQLLPNLR |
| P61457 | Pterin-4-alpha-Carbinolamine dehydratase | AVGWNELEGR |
| Q14671 | Pumilio homolog 1 | AMGEQLLPGK |
| P00491 | Purine nuCleOSide phOSphorylase | NTAEWLLSHTK |
| P00491 | Purine nuCleOSide phOSphorylase | QAAQKLEQFVSILMASIPLPDK |
| P00491 | Purine nuCleOSide phOSphorylase | LGADAVGMSTVPEVIVAR |
| P00491 | Purine nuCleOSide phOSphorylase | ANHEEVLAAGK |
| P00491 | Purine nuCleOSide phOSphorylase | VFGFSLITNK |
| P00491 | Purine nuCleOSide phOSphorylase | ELQEGTYVMVAGPSFETVAECR |
| P00491 | Purine nuCleOSide phOSphorylase | FGDRFPAMSDAYDR |
| P55786 | PuromyCin-sensitive aminopeptidase | VLGATLLPDLIQK |
| P55786 | PuromyCin-sensitive aminopeptidase | VLTFALSEEVRPQDTVSVIGGVAGGSK |
| P55786 | PuromyCin-sensitive aminopeptidase | NAATEDLWESLENASGKPIAAVMNTWTK |
| P55786 | PuromyCin-sensitive aminopeptidase | QMGFPLIYVEAEQVEDDRLLR |
| P55786 | PuromyCin-sensitive aminopeptidase | LGLQNDLFSLAR |
| P55786 | PuromyCin-sensitive aminopeptidase | TQYSSAMLESLLPGIR |
| P55786 | PuromyCin-sensitive aminopeptidase | ATFDISLVVPK |
| P55786 | PuromyCin-sensitive aminopeptidase | IDFVGELNDK |
| P55786 | PuromyCin-sensitive aminopeptidase | LNLGTVGFYR |
| P55786 | PuromyCin-sensitive aminopeptidase | FALEVAAK |
| P55786 | PuromyCin-sensitive aminopeptidase | DYFNVPYPLPK |
| P55786 | PuromyCin-sensitive aminopeptidase | QILSADLR |
| P55786 | PuromyCin-sensitive aminopeptidase | SKDGVCVR |
| Q5T1J5 | Putative Coiled-Coil-helix-Coiled-Coil-helix domain-Containing protein CHCHD2P9, mitoChondrial | AAPRPAPVAQPPAAAPPSAVGSSAAAPR |
| A6NGU5 | Putative gamma-glutamyltranspeptidase 3 | FVDVTEVVR |
| A6NGU5 | Putative gamma-glutamyltranspeptidase 3 | LFQPSIQLAR |
| P0C6E5 | Putative high mobility group protein B3-like protein | mSAYAFFVQTCR |
| P0C6E5 | Putative high mobility group protein B3-like protein | mSAYAFFVQTCREEHK |
| P01893 | Putative HLA Class I histoCompatibility antigen, alpha Chain H | DGEDQTQDTELVETRPAGDGTFQK |
| A8K0S8 | Putative homeobox protein Meis3-like 2 | CELATCSPR |
| Q9Y303 | Putative N-aCetylgluCOSamine-6-phOSphate deaCetylase | GICVSLGHSVADLR |
| O43143 | Putative pre-mRNA-spliCing faCtor ATP-dependent RNA heliCase DHX15 | TLATDILMGVLK |
| Q8IZP2 | Putative protein FAM10A4 | AIEINPDSAQPYK |
| Q8IZP2 | Putative protein FAM10A4 | AIDLFTDAIK |
| Q8IZP2 | Putative protein FAM10A4 | VAAIEALNDGELQK |
| Q8IZP2 | Putative protein FAM10A4 | SEENTKEEKPDSK |
| Q8IZP2 | Putative protein FAM10A4 | LAILYAK |
| Q92928 | Putative Ras-related protein Rab-1C | NATNVEQAFMTMAAEIK |
| Q92928 | Putative Ras-related protein Rab-1C | EFADSLGIPFLETSAK |
| Q92928 | Putative Ras-related protein Rab-1C | IQIWDTAGQER |
| Q2M238 | Putative RRN3-like protein FLJ77916 | QLLSGNLK |
| A6NL28 | Putative tropomyOSin alpha-3 Chain-like protein | IQVLQQQADDAEERAER |
| O00764 | Pyridoxal kinase | GQVLNSDELQELYEGLR |
| O00764 | Pyridoxal kinase | DIEDPEIVVQATVL |
| O00764 | Pyridoxal kinase | VVPLADIITPNQFEAELLSGR |
| Q08257 | Quinone oxidoreduCtase | DLSLLSHGGR |
| Q08257 | Quinone oxidoreduCtase | AGESVLVHGASGGVGLAACQIAR |
| Q08257 | Quinone oxidoreduCtase | VFEFGGPEVLK |
| Q08257 | Quinone oxidoreduCtase | VAEAHENIIHGSGATGK |
| Q08257 | Quinone oxidoreduCtase | KPLLPYTPGSDVAGVIEAVGDNASAFK |
| Q53FA7 | Quinone oxidoreduCtase PIG3 | QMLVNAFTEQILPHFSTEGPQR |
| P31150 | Rab GDP dissoCiation inhibitor alpha | KQNDVFGEAEQ |
| P31150 | Rab GDP dissoCiation inhibitor alpha | FQLLEGPPESMGR |
| P31150 | Rab GDP dissoCiation inhibitor alpha | YIAIASTTVETTDPEKEVEPALELLEPIDQK |
| P31150 | Rab GDP dissoCiation inhibitor alpha | IICILSHPIK |
| P31150 | Rab GDP dissoCiation inhibitor alpha | TDDYLDQPCLETVNR |
| P31150 | Rab GDP dissoCiation inhibitor alpha | IYKVPSTETEALASNLMGMFEK |
| P50395 | Rab GDP dissoCiation inhibitor beta | SPYLYPLYGLGELPQGFAR |
| P50395 | Rab GDP dissoCiation inhibitor beta | KFDLGQDVIDFTGHALALYR |
| P50395 | Rab GDP dissoCiation inhibitor beta | DLGTESQIFISR |
| P50395 | Rab GDP dissoCiation inhibitor beta | NTNDANSCQIIIPQNQVNR |
| P50395 | Rab GDP dissoCiation inhibitor beta | TDDYLDQPCYETINR |
| P50395 | Rab GDP dissoCiation inhibitor beta | YIAIVSTTVETKEPEK |
| P50395 | Rab GDP dissoCiation inhibitor beta | TYDATTHFETTCDDIK |
| P50395 | Rab GDP dissoCiation inhibitor beta | KSDIYVCMISFAHNVAAQGK |
| P50395 | Rab GDP dissoCiation inhibitor beta | LSAIYGGTYMLNKPIEEIIVQNGK |
| P50395 | Rab GDP dissoCiation inhibitor beta | IYKVPSTEAEALASSLMGLFEK |
| P50395 | Rab GDP dissoCiation inhibitor beta | MLLYTEVTR |
| P50395 | Rab GDP dissoCiation inhibitor beta | FLMANGQLVK |
| P50395 | Rab GDP dissoCiation inhibitor beta | EIRPALELLEPIEQK |
| P50395 | Rab GDP dissoCiation inhibitor beta | RMTGSEFDFEEMK |
| P50395 | Rab GDP dissoCiation inhibitor beta | FVSISDLLVPK |
| P50395 | Rab GDP dissoCiation inhibitor beta | MTGSEFDFEEMKR |
| P50395 | Rab GDP dissoCiation inhibitor beta | VPSTEAEALASSLMGLFEK |
| P50395 | Rab GDP dissoCiation inhibitor beta | SEGEIAR |
| P50395 | Rab GDP dissoCiation inhibitor beta | NPYYGGESASITPLEDLYKR |
| P50395 | Rab GDP dissoCiation inhibitor beta | QLICDPSYVK |
| P50395 | Rab GDP dissoCiation inhibitor beta | TFEGIDPK |
| P50395 | Rab GDP dissoCiation inhibitor beta | FKIPGSPPESMGR |
| P50395 | Rab GDP dissoCiation inhibitor beta | VGQVIR |
| P50395 | Rab GDP dissoCiation inhibitor beta | NPYYGGESASITPLEDLYK |
| P35241 | Radixin | EAILNDEIYCPPETAVLLASYAVQAK |
| P35241 | Radixin | KTQNDVLHAENVK |
| P43487 | Ran-speCifiC GTPase-aCtivating protein | FASENDLPEWK |
| P43487 | Ran-speCifiC GTPase-aCtivating protein | TLEEDEEELFK |
| P46940 | Ras GTPase-aCtivating-like protein IQGAP1 | ILAIGLINEALDEGDAQK |
| P46940 | Ras GTPase-aCtivating-like protein IQGAP1 | MLQHAASNK |
| Q15404 | Ras suppressor protein 1 | NLEVLNFFNNQIEELPTQISSLQK |
| P63000 | Ras-related C3 botulinum toxin substrate 1 | KLTPITYPQGLAMAK |
| P62491 | Ras-related protein Rab-11A | VVLIGDSGVGK |
| P62491 | Ras-related protein Rab-11A | STIGVEFATR |
| P59190 | Ras-related protein Rab-15 | IQIWDTAGQER |
| P62820 | Ras-related protein Rab-1A | NATNVEQSFMTMAAEIK |
| P62820 | Ras-related protein Rab-1A | EFADSLGIPFLETSAK |
| P62820 | Ras-related protein Rab-1A | IQIWDTAGQER |
| P62820 | Ras-related protein Rab-1A | QWLQEIDRYASENVNK |
| P62820 | Ras-related protein Rab-1A | MGPGATAGGAEK |
| P61019 | Ras-related protein Rab-2A | TASNVEEAFINTAK |
| P61019 | Ras-related protein Rab-2A | IQEGVFDINNEANGIK |
| P61019 | Ras-related protein Rab-2A | LQIWDTAGQESFR |
| P20339 | Ras-related protein Rab-5A | LVLLGESAVGK |
| P51148 | Ras-related protein Rab-5C | TAMNVNEIFMAIAK |
| P51148 | Ras-related protein Rab-5C | QASPNIVIALAGNK |
| P51148 | Ras-related protein Rab-5C | LVLLGESAVGK |
| P51149 | Ras-related protein Rab-7a | FQSLGVAFYR |
| P51149 | Ras-related protein Rab-7a | ATIGADFLTK |
| P61224 | Ras-related protein Rap-1b | LVVLGSGGVGK |
| A6NIZ1 | Ras-related protein Rap-1b-like protein | INVNEIFYDLVR |
| Q15262 | ReCeptor-type tyrOSine-protein phOSphatase kappa | VLLTRPGEGGTGLPGPPLITR |
| Q5VT52 | Regulation of nuClear pre-mRNA domain-Containing protein 2 | KLDQLK |
| Q15293 | RetiCuloCalbin-1 | LTKEEILENWNMFVGSQATNYGEDLTK |
| Q15293 | RetiCuloCalbin-1 | TFDQLTPDESK |
| Q15293 | RetiCuloCalbin-1 | HWILPQDYDHAQAEAR |
| Q8NFJ5 | RetinoiC aCid-induCed protein 3 | AYSQEEITQGFEETGDTLYAPYSTHFQLQNQPPQK |
| P02753 | Retinol-binding protein 4 | LLNNWDVCADMVGTFTDTEDPAK |
| P02753 | Retinol-binding protein 4 | ARFSGTWYAMAK |
| P02753 | Retinol-binding protein 4 | LLNLDGTCADSYSFVFSRDPNGLPPEAQK |
| P02753 | Retinol-binding protein 4 | YWGVASFLQK |
| P02753 | Retinol-binding protein 4 | GNDDHWIVDTDYDTYAVQYSCR |
| P02753 | Retinol-binding protein 4 | LLNLDGTCADSYSFVFSR |
| P02753 | Retinol-binding protein 4 | FSGTWYAMAK |
| P02753 | Retinol-binding protein 4 | LIVHNGYCDGR |
| P02753 | Retinol-binding protein 4 | QRQEELCLAR |
| P02753 | Retinol-binding protein 4 | VKENFDK |
| P02753 | Retinol-binding protein 4 | KDPEGLFLQDNIVAEFSVDETGQMSATAK |
| P52565 | Rho GDP-dissoCiation inhibitor 1 | AEEYEFLTPVEEAPK |
| P52565 | Rho GDP-dissoCiation inhibitor 1 | IDKTDYMVGSYGPR |
| P52565 | Rho GDP-dissoCiation inhibitor 1 | YKEALLGR |
| P52565 | Rho GDP-dissoCiation inhibitor 1 | VAVSADPNVPNVVVTGLTLVCSSAPGPLELDLTGDLESFKK |
| P52566 | Rho GDP-dissoCiation inhibitor 2 | TLLGDGPVVTDPK |
| P52566 | Rho GDP-dissoCiation inhibitor 2 | ELQEMDKDDESLIK |
| P52566 | Rho GDP-dissoCiation inhibitor 2 | APNVVVTR |
| P52566 | Rho GDP-dissoCiation inhibitor 2 | ATFMVGSYGPRPEEYEFLTPVEEAPK |
| P52566 | Rho GDP-dissoCiation inhibitor 2 | TLLGDGPVVTDPKAPNVVVTR |
| Q07960 | Rho GTPase-aCtivating protein 1 | FLLDHQGELFPSPDPSGL |
| P08134 | Rho-related GTP-binding protein RhoC | KLVIVGDGACGK |
| P08134 | Rho-related GTP-binding protein RhoC | ISAFGYLECSAK |
| P13489 | RibonuClease inhibitor | VLWLADCDVSDSSCSSLAATLLANHSLR |
| P13489 | RibonuClease inhibitor | LQLEYCSLSAASCEPLASVLR |
| P13489 | RibonuClease inhibitor | ELTVSNNDINEAGVR |
| P13489 | RibonuClease inhibitor | LSLQNCCLTGAGCGVLSSTLR |
| P13489 | RibonuClease inhibitor | ELSLAGNELGDEGAR |
| P13489 | RibonuClease inhibitor | VNPALAELNLR |
| P13489 | RibonuClease inhibitor | LGDVGMAELCPGLLHPSSR |
| P13489 | RibonuClease inhibitor | SNELGDVGVHCVLQGLQTPSCK |
| P13489 | RibonuClease inhibitor | ELDLSNNCLGDAGILQLVESVR |
| P13489 | RibonuClease inhibitor | LDDCGLTEAR |
| P13489 | RibonuClease inhibitor | WAELLPLLQQCQVVR |
| P13489 | RibonuClease inhibitor | ELCQGLGQPGSVLR |
| P13489 | RibonuClease inhibitor | DLCGIVASK |
| P13489 | RibonuClease inhibitor | SCSFTAACCSHFSSVLAQNR |
| O00584 | RibonuClease T2 | HGTCAAQVDALNSQKK |
| O00584 | RibonuClease T2 | HGTCAAQVDALNSQK |
| O00584 | RibonuClease T2 | LGIKPSINYYQVADFK |
| O00584 | RibonuClease T2 | SLELYRELDLNSVLLK |
| O00584 | RibonuClease T2 | ELDLNSVLLK |
| P49247 | RibOSe-5-phOSphate isomerase | GIPIEVIPMAYVPVSR |
| P60891 | RibOSe-phOSphate pyrophOSphokinase 1 | NCTIVSPDAGGAK |
| P60891 | RibOSe-phOSphate pyrophOSphokinase 1 | VTAVIPCFPYAR |
| P11908 | RibOSe-phOSphate pyrophOSphokinase 2 | VTAVIPCFPYAR |
| Q9Y3A5 | RibOSome maturation protein SBDS | LTNVAVVR |
| Q9P2E9 | RibOSome-binding protein 1 | HLEEIVEK |
| Q9P2E9 | RibOSome-binding protein 1 | DALNQATSQVESK |
| Q9P2E9 | RibOSome-binding protein 1 | KGEGAPIQGK |
| Q96AT9 | RibulOSe-phOSphate 3-epimerase | IGPSILNSDLANLGAECLR |
| Q96AT9 | RibulOSe-phOSphate 3-epimerase | SVINLLR |
| Q96AT9 | RibulOSe-phOSphate 3-epimerase | NVCSEAAQKR |
| P35637 | RNA-binding protein FUS | AAIDWFDGK |
| P31153 | S-adenOSylmethionine synthase isoform type-2 | TQVTVQYMQDR |
| P31153 | S-adenOSylmethionine synthase isoform type-2 | VACETVAK |
| Q9Y3Z3 | SAM domain and HD domain-Containing protein 1 | VGNIIDTMITDAFLK |
| Q9Y3Z3 | SAM domain and HD domain-Containing protein 1 | EQIVGPLESPVEDSLWPYK |
| Q9Y3Z3 | SAM domain and HD domain-Containing protein 1 | EWNDSTSVQNPTR |
| Q9Y3Z3 | SAM domain and HD domain-Containing protein 1 | LTDNIFLEILYSTDPK |
| Q86VB7 | SCavenger reCeptor Cysteine-riCh type 1 protein M130 | LQEGPTSCSGR |
| O95969 | SeCretoglobin family 1D member 2 | SLIAEVLVK |
| Q13228 | Selenium-binding protein 1 | NTGTEAPDYLATVDVDPK |
| Q13228 | Selenium-binding protein 1 | GGFVLLDGETFEVK |
| Q13228 | Selenium-binding protein 1 | GGPVQVLEDEELK |
| Q13228 | Selenium-binding protein 1 | IYVVDVGSEPR |
| Q13228 | Selenium-binding protein 1 | GTWERPGGAAPLGYDFWYQPR |
| Q13228 | Selenium-binding protein 1 | FLHNPDAAQGFVGCALSSTIQR |
| Q13228 | Selenium-binding protein 1 | RVAGGPQMIQLSLDGK |
| Q13228 | Selenium-binding protein 1 | HEIVQTLSLK |
| Q13228 | Selenium-binding protein 1 | DGFNPADVEAGLYGSHLYVWDWQR |
| Q13228 | Selenium-binding protein 1 | LTGQLFLGGSIVK |
| Q13228 | Selenium-binding protein 1 | GGPVQVLEDEELKSQPEPLVVK |
| Q13228 | Selenium-binding protein 1 | EGSVMLQVDVDTVK |
| Q13228 | Selenium-binding protein 1 | NEGGTWSVEK |
| Q13228 | Selenium-binding protein 1 | LVLPSLISSR |
| Q13228 | Selenium-binding protein 1 | DGLIPLEIR |
| Q13228 | Selenium-binding protein 1 | SPQYCQVIHR |
| Q13228 | Selenium-binding protein 1 | LNPNFLVDFGKEPLGPALAHELR |
| P49908 | Selenoprotein P | LPTDSELAPR |
| Q9NVA2 | Septin-11 | STSQGFCFNILCVGETGIGK |
| Q9NVA2 | Septin-11 | AAAQLLQSQAQQSGAQQTKK |
| Q15019 | Septin-2 | ASIPFSVVGSNQLIEAK |
| Q15019 | Septin-2 | LTVVDTPGYGDAINCR |
| Q15019 | Septin-2 | STLINSLFLTDLYPER |
| Q15019 | Septin-2 | TMLITHMQDLQEVTQDLHYENFR |
| Q15019 | Septin-2 | ADTLTLK |
| Q15019 | Septin-2 | MQAQMQMQMQGGDGDGGALGHHV |
| Q16181 | Septin-7 | STLINSLFLTDLYSPEYPGPSHR |
| Q16181 | Septin-7 | VNIIPIIAK |
| Q16181 | Septin-7 | ILEQQNSSR |
| Q9UHD8 | Septin-9 | QVENAGAIGPSR |
| Q9UHD8 | Septin-9 | RVELSGPK |
| Q9UHD8 | Septin-9 | VVNIVPVIAK |
| Q9UHD8 | Septin-9 | STLINTLFK |
| Q9UHD8 | Septin-9 | APVDFGYVGIDSILEQMR |
| Q01130 | Serine/arginine-riCh spliCing faCtor 2 | VGDVYIPR |
| P63151 | Serine/threonine-protein phOSphatase 2A 55 kDa regulatory subunit B alpha isoform | SFFSEIISSISDVK |
| P63151 | Serine/threonine-protein phOSphatase 2A 55 kDa regulatory subunit B alpha isoform | VVIFQQEQENK |
| Q00005 | Serine/threonine-protein phOSphatase 2A 55 kDa regulatory subunit B beta isoform | SFFSEIISSISDVK |
| Q15257 | Serine/threonine-protein phOSphatase 2A aCtivator | LDEEAENLVATVVPTHLAAAVPEVAVYLK |
| P67775 | Serine/threonine-protein phOSphatase 2A CatalytiC subunit alpha isoform | ELDQWIEQLNECK |
| P67775 | Serine/threonine-protein phOSphatase 2A CatalytiC subunit alpha isoform | YSFLQFDPAPR |
| P67775 | Serine/threonine-protein phOSphatase 2A CatalytiC subunit alpha isoform | RGEPHVTR |
| P62714 | Serine/threonine-protein phOSphatase 2A CatalytiC subunit beta isoform | ELDQWVEQLNECK |
| P62714 | Serine/threonine-protein phOSphatase 2A CatalytiC subunit beta isoform | YSFLQFDPAPR |
| P53041 | Serine/threonine-protein phOSphatase 5 | AIAGDEHKR |
| P62140 | Serine/threonine-protein phOSphatase PP1-beta CatalytiC subunit | EIFLSQPILLELEAPLK |
| P62140 | Serine/threonine-protein phOSphatase PP1-beta CatalytiC subunit | IVQMTEAEVR |
| P62140 | Serine/threonine-protein phOSphatase PP1-beta CatalytiC subunit | SREIFLSQPILLELEAPLK |
| P62140 | Serine/threonine-protein phOSphatase PP1-beta CatalytiC subunit | GNHECASINR |
| P62140 | Serine/threonine-protein phOSphatase PP1-beta CatalytiC subunit | AHQVVEDGYEFFAK |
| P36873 | Serine/threonine-protein phOSphatase PP1-gamma CatalytiC subunit | TFTDCFNCLPIAAIVDEK |
| P36873 | Serine/threonine-protein phOSphatase PP1-gamma CatalytiC subunit | EIFLSQPILLELEAPLK |
| P36873 | Serine/threonine-protein phOSphatase PP1-gamma CatalytiC subunit | GNHECASINR |
| P35237 | Serpin B6 | TYIGEIFTQILVLPYVGK |
| P35237 | Serpin B6 | GNTAAQMAQILSFNK |
| P35237 | Serpin B6 | FYQAEMEELDFISAVEK |
| P35237 | Serpin B6 | IAELLSPGSVDPLTR |
| P35237 | Serpin B6 | ADFSGMSQTDLSLSK |
| P35237 | Serpin B6 | NLGMTDAFELGK |
| P35237 | Serpin B6 | SCDFLSSFR |
| P35542 | Serum amyloid A-4 protein | GNYDAAQRGPGGVWAAK |
| P35542 | Serum amyloid A-4 protein | EALQGVGDMGR |
| P35542 | Serum amyloid A-4 protein | GPGGVWAAK |
| P35542 | Serum amyloid A-4 protein | AYWDIMISNHQNSNR |
| P02743 | Serum amyloid P-Component | ERVGEYSLYIGR |
| P02743 | Serum amyloid P-Component | IVLGQEQDSYGGKFDR |
| P02743 | Serum amyloid P-Component | GYVIIKPLVWV |
| P02743 | Serum amyloid P-Component | QGYFVEAQPK |
| P02743 | Serum amyloid P-Component | IVLGQEQDSYGGK |
| P02743 | Serum amyloid P-Component | VGEYSLYIGR |
| P27169 | Serum paraoxonase/arylesterase 1 | IFFYDSENPPASEVLR |
| P27169 | Serum paraoxonase/arylesterase 1 | GIETGSEDLEILPNGLAFISSGLK |
| P27169 | Serum paraoxonase/arylesterase 1 | VVAEGFDFANGINISPDGK |
| P27169 | Serum paraoxonase/arylesterase 1 | ILLMDLNEEDPTVLELGITGSK |
| P27169 | Serum paraoxonase/arylesterase 1 | SLDFNTLVDNISVDPETGDLWVGCHPNGMK |
| P27169 | Serum paraoxonase/arylesterase 1 | SLLHIK |
| P27169 | Serum paraoxonase/arylesterase 1 | IQNILTEEPK |
| P27169 | Serum paraoxonase/arylesterase 1 | EVQPVELPNCNLVK |
| P49591 | Seryl-tRNA synthetase, CytoplasmiC | ELVSCSNCTDYQAR |
| P49591 | Seryl-tRNA synthetase, CytoplasmiC | TICAILENYQTEK |
| P49591 | Seryl-tRNA synthetase, CytoplasmiC | KEPVGDDESVPENVLSFDDLTADALANLK |
| P49591 | Seryl-tRNA synthetase, CytoplasmiC | VLDLDLFR |
| P49591 | Seryl-tRNA synthetase, CytoplasmiC | LLIDEAILK |
| P49591 | Seryl-tRNA synthetase, CytoplasmiC | FKDPGLVDQLVK |
| P04278 | Sex hormone-binding globulin | VVLSSGSGPGLDLPLVLGLPLQLK |
| P10768 | S-formylglutathione hydrolase | SVSAFAPICNPVLCPWGK |
| P10768 | S-formylglutathione hydrolase | MYSYVTEELPQLINANFPVDPQR |
| P10768 | S-formylglutathione hydrolase | AFSGYLGTDQSK |
| P10768 | S-formylglutathione hydrolase | KAFSGYLGTDQSK |
| P10768 | S-formylglutathione hydrolase | VFEHDSVELNCK |
| P10768 | S-formylglutathione hydrolase | SGYHQSASEHGLVVIAPDTSPR |
| P10768 | S-formylglutathione hydrolase | AYDATHLVK |
| P10768 | S-formylglutathione hydrolase | CFGGLQK |
| P10768 | S-formylglutathione hydrolase | AETGKCPALYWLSGLTCTEQNFISK |
| P10768 | S-formylglutathione hydrolase | CPALYWLSGLTCTEQNFISK |
| Q9H299 | SH3 domain-binding glutamiC aCid-riCh-like protein 3 | VYSTSVTGSR |
| O75368 | SH3 domain-binding glutamiC aCid-riCh-like protein | VYIASSSGSTAIK |
| O75368 | SH3 domain-binding glutamiC aCid-riCh-like protein | ENNAVYAFLGLTAPPGSK |
| O75368 | SH3 domain-binding glutamiC aCid-riCh-like protein | VYIASSSGSTAIKK |
| O75368 | SH3 domain-binding glutamiC aCid-riCh-like protein | GDYDAFFEAR |
| O75368 | SH3 domain-binding glutamiC aCid-riCh-like protein | DIAANEENRK |
| Q9NR45 | SialiC aCid synthase | QVYQIVKPLNPNFCFLQCTSAYPLQPEDVNLR |
| Q9NR45 | SialiC aCid synthase | VKIPEGTILTMDMLTVK |
| Q9NR45 | SialiC aCid synthase | PLELELCPGR |
| Q9NR45 | SialiC aCid synthase | VGSGDTNNFPYLEK |
| Q9NR45 | SialiC aCid synthase | QLLPCEMACNEK |
| Q9NR45 | SialiC aCid synthase | GSDHSASLEPGELAELVR |
| P37108 | Signal reCognition partiCle 14 kDa protein | VLLESEQFLTELTR |
| P49458 | Signal reCognition partiCle 9 kDa protein | PQYQTWEEFSR |
| P49458 | Signal reCognition partiCle 9 kDa protein | YRHSDGNLCVK |
| P42224 | Signal transduCer and aCtivator of transCription 1-alpha/beta | VMAAENIPENPLK |
| P42224 | Signal transduCer and aCtivator of transCription 1-alpha/beta | GLNVDQLNMLGEK |
| P42224 | Signal transduCer and aCtivator of transCription 1-alpha/beta | ELSAVTFPDIIR |
| P42224 | Signal transduCer and aCtivator of transCription 1-alpha/beta | LLGPNASPDGLIPWTR |
| P62304 | Small nuClear ribonuCleoprotein E | VMVQPINLIFR |
| A8MWD9 | Small nuClear ribonuCleoprotein G-like protein | HVQGILR |
| A8MWD9 | Small nuClear ribonuCleoprotein G-like protein | GNSIIMLEALERV |
| P62314 | Small nuClear ribonuCleoprotein Sm D1 | YFILPDSLPLDTLLVDVEPK |
| P62316 | Small nuClear ribonuCleoprotein Sm D2 | NNTQVLINCR |
| P62316 | Small nuClear ribonuCleoprotein Sm D2 | HCNMVLENVK |
| P62318 | Small nuClear ribonuCleoprotein Sm D3 | VLHEAEGHIVTCETNTGEVYR |
| O75691 | Small subunit proCessome Component 20 homolog | LISIWSR |
| P63165 | Small ubiquitin-related modifier 1 | IADNHTPK |
| P61956 | Small ubiquitin-related modifier 2 | VAGQDGSVVQFK |
| P61956 | Small ubiquitin-related modifier 2 | RHTPLSK |
| Q13126 | S-methyl-5'-thioadenOSine phOSphorylase | GTMVTIEGPR |
| Q13126 | S-methyl-5'-thioadenOSine phOSphorylase | TWGADVINMTTVPEVVLAK |
| Q13126 | S-methyl-5'-thioadenOSine phOSphorylase | IGIIGGTGLDDPEILEGRTEK |
| Q13126 | S-methyl-5'-thioadenOSine phOSphorylase | IGIIGGTGLDDPEILEGR |
| O95436 | Sodium-dependent phOSphate transport protein 2B | YLEGAAGQQPTAPDK |
| Q00796 | Sorbitol dehydrogenase | AMGAAQVVVTDLSATR |
| Q00796 | Sorbitol dehydrogenase | LPDNVTFEEGALIEPLSVGIHACR |
| Q00796 | Sorbitol dehydrogenase | YCNTWPVAISMLASK |
| Q00796 | Sorbitol dehydrogenase | VLVCGAGPIGMVTLLVAK |
| Q00796 | Sorbitol dehydrogenase | KPMVLGHEASGTVEK |
| Q00796 | Sorbitol dehydrogenase | YNLSPSIFFCATPPDDGNLCR |
| Q00796 | Sorbitol dehydrogenase | ALEAFETFKK |
| Q00796 | Sorbitol dehydrogenase | SGGNLVLVGLGSEMTTVPLLHAAIR |
| Q00796 | Sorbitol dehydrogenase | IGNFIVK |
| Q00796 | Sorbitol dehydrogenase | RGGVTLGHK |
| Q00796 | Sorbitol dehydrogenase | LENYPIPEPGPNEVLLR |
| Q00796 | Sorbitol dehydrogenase | EIGADLVLQISK |
| Q00796 | Sorbitol dehydrogenase | VAIEPGAPR |
| Q00796 | Sorbitol dehydrogenase | SVNVKPLVTHR |
| Q01082 | SpeCtrin beta Chain, brain 1 | IIYIR |
| P52788 | Spermine synthase | EGREFDYVINDLTAVPISTSPEEDSTWEFLR |
| P52788 | Spermine synthase | RLPPIVR |
| P63208 | S-phase kinase-assoCiated protein 1 | VDQGTLFELILAANYLDIK |
| P63208 | S-phase kinase-assoCiated protein 1 | TMLEDLGMDDEGDDDPVPLPNVNAAILKK |
| Q9HCB6 | Spondin-1 | MIQMEPQFGGAPCPETVQR |
| Q9HCB6 | Spondin-1 | SLAELGDCNEDLEQVEK |
| Q9HCB6 | Spondin-1 | AAPSAEFSVDR |
| Q9HCB6 | Spondin-1 | IRPLTSLDHPQSPFYDPEGGSITQVAR |
| Q9HCB6 | Spondin-1 | IIYFQDEGSLTK |
| Q9HCB6 | Spondin-1 | VVQDLIPWDAGTDSGVTYESPNKPTIPQEK |
| Q9HCB6 | Spondin-1 | AQWPAWQPLNVR |
| Q9HCB6 | Spondin-1 | QQSDEVLTVIK |
| Q9HCB6 | Spondin-1 | LCGGGIQER |
| Q9HCB6 | Spondin-1 | VTLSAAPPSYFR |
| Q9HCB6 | Spondin-1 | VEGDPDFYKPGTSYR |
| Q9HCB6 | Spondin-1 | MNPADGSMCK |
| Q14247 | SrC substrate CortaCtin | ASAGHAVSIAQDDAGADDWETDPDFVNDVSEK |
| Q14247 | SrC substrate CortaCtin | SAVGHEYQSK |
| Q14247 | SrC substrate CortaCtin | HCSQVDSVR |
| O76061 | StannioCalCin-2 | HDLCAAAQENTR |
| P31948 | Stress-induCed-phOSphoprotein 1 | ELDPTNMTYITNQAAVYFEK |
| P31948 | Stress-induCed-phOSphoprotein 1 | LMDVGLIAIR |
| P31948 | Stress-induCed-phOSphoprotein 1 | AMADPEVQQIMSDPAMR |
| P31948 | Stress-induCed-phOSphoprotein 1 | FMNPFNMPNLYQK |
| P31948 | Stress-induCed-phOSphoprotein 1 | RTYEEGLK |
| P31948 | Stress-induCed-phOSphoprotein 1 | ALSVGNIDDALQCYSEAIK |
| P31948 | Stress-induCed-phOSphoprotein 1 | LAYINPDLALEEK |
| P31948 | Stress-induCed-phOSphoprotein 1 | HEANNPQLK |
| P31948 | Stress-induCed-phOSphoprotein 1 | AAALEAMK |
| P31948 | Stress-induCed-phOSphoprotein 1 | NPVIAQK |
| O00391 | Sulfhydryl oxidase 1 | LAGAPSEDPQFPK |
| O00391 | Sulfhydryl oxidase 1 | FGVTDFPSCYLLFR |
| Q9UBE0 | SUMO-aCtivating enzyme subunit 1 | YCFSEMAPVCAVVGGILAQEIVK |
| Q9UBE0 | SUMO-aCtivating enzyme subunit 1 | NDVLDSLGISPDLLPEDFVR |
| Q9UBT2 | SUMO-aCtivating enzyme subunit 2 | VLVVGAGGIGCELLK |
| P63279 | SUMO-Conjugating enzyme UBC9 | DWRPAITIK |
| P63279 | SUMO-Conjugating enzyme UBC9 | NPDGTMNLMNWECAIPGKK |
| P63279 | SUMO-Conjugating enzyme UBC9 | QILLGIQELLNEPNIQDPAQAEAYTIYCQNR |
| Q99536 | SynaptiC vesiCle membrane protein VAT-1 homolog | LQSRPAAPPAPGPGQLTLR |
| O00445 | Synaptotagmin-5 | NEAIGR |
| O00560 | Syntenin-1 | SIDNGIFVQLVQANSPASLVGLR |
| O00560 | Syntenin-1 | FGDQVLQINGENCAGWSSDK |
| O00560 | Syntenin-1 | VIQAQTAFSANPANPAILSEASAPIPHDGNLYPR |
| O00560 | Syntenin-1 | ANVAVVSGAPLQGQLVARPSSINYMVAPVTGNDVGIR |
| O00560 | Syntenin-1 | DSTGHVGFIFK |
| O00560 | Syntenin-1 | NGLLTEHNICEINGQNVIGLK |
| Q9Y490 | Talin-1 | EGISQEALHTQMLTAVQEISHLIEPLANAAR |
| Q9Y490 | Talin-1 | VLVQNAAGSQEK |
| Q9Y490 | Talin-1 | AVSSAIAQLLGEVAQGNENYAGIAAR |
| Q9Y490 | Talin-1 | GVAALTSDPAVQAIVLDTASDVLDK |
| Q9Y490 | Talin-1 | VSQMAQYFEPLTLAAVGAASK |
| Q9Y490 | Talin-1 | AATAPLLEAVDNLSAFASNPEFSSIPAQISPEGR |
| Q9Y490 | Talin-1 | GTEWVDPEDPTVIAENELLGAAAAIEAAAK |
| Q9Y490 | Talin-1 | GVGAAATAVTQALNELLQHVK |
| P17987 | T-Complex protein 1 subunit alpha | VLCELADLQDKEVGDGTTSVVIIAAELLK |
| P17987 | T-Complex protein 1 subunit alpha | YPVNSVNILK |
| P17987 | T-Complex protein 1 subunit alpha | MLVDDIGDVTITNDGATILK |
| P17987 | T-Complex protein 1 subunit alpha | SLLVIPNTLAVNAAQDSTDLVAK |
| P78371 | T-Complex protein 1 subunit beta | LTSFIGAIAIGDLVK |
| P78371 | T-Complex protein 1 subunit beta | QLIYNYPEQLFGAAGVMAIEHADFAGVER |
| P78371 | T-Complex protein 1 subunit beta | GATQQILDEAER |
| P78371 | T-Complex protein 1 subunit beta | MLPTIIADNAGYDSADLVAQLR |
| P78371 | T-Complex protein 1 subunit beta | VAEIEHAEK |
| P50991 | T-Complex protein 1 subunit delta | AYILNLVK |
| P48643 | T-Complex protein 1 subunit epsilon | LGFAGLVQEISFGTTK |
| P48643 | T-Complex protein 1 subunit epsilon | WVGGPEIELIAIATGGR |
| P48643 | T-Complex protein 1 subunit epsilon | RSLHDALCVIR |
| P48643 | T-Complex protein 1 subunit epsilon | ETGANLAICQWGFDDEANHLLLQNNLPAVR |
| P48643 | T-Complex protein 1 subunit epsilon | IADGYEQAAR |
| P48643 | T-Complex protein 1 subunit epsilon | AFADALEVIPMALSENSGMNPIQTMTEVR |
| P49368 | T-Complex protein 1 subunit gamma | MLLDPMGGIVMTNDGNAILR |
| P49368 | T-Complex protein 1 subunit gamma | TLIQNCGASTIR |
| P49368 | T-Complex protein 1 subunit gamma | IPGGIIEDSCVLR |
| P49368 | T-Complex protein 1 subunit gamma | NVLLDPQLVPGGGASEMAVAHALTEK |
| P49368 | T-Complex protein 1 subunit gamma | WSSLACNIALDAVK |
| P49368 | T-Complex protein 1 subunit gamma | IVLLDSSLEYK |
| P49368 | T-Complex protein 1 subunit gamma | KVQSGNINAAK |
| P49368 | T-Complex protein 1 subunit gamma | ELGIWEPLAVK |
| P50990 | T-Complex protein 1 subunit theta | AIADTGANVVVTGGK |
| P50990 | T-Complex protein 1 subunit theta | LFVTNDAATILR |
| P50990 | T-Complex protein 1 subunit theta | FAEAFEAIPR |
| P50990 | T-Complex protein 1 subunit theta | LATNAAVTVLR |
| P40227 | T-Complex protein 1 subunit zeta | VLAQNSGFDLQETLVK |
| P40227 | T-Complex protein 1 subunit zeta | NAIDDGCVVPGAGAVEVAMAEALIK |
| P40227 | T-Complex protein 1 subunit zeta | GRAQLGVQAFADALLIIPK |
| P40227 | T-Complex protein 1 subunit zeta | VHAELADVLTEAVVDSILAIK |
| P40227 | T-Complex protein 1 subunit zeta | MLVSGAGDIK |
| P40227 | T-Complex protein 1 subunit zeta | VATAQDDITGDGTTSNVLIIGELLK |
| P40227 | T-Complex protein 1 subunit zeta | GIDPFSLDALSK |
| Q9BXT5 | Testis-expressed sequenCe 15 protein | KILQLK |
| P05452 | TetraneCtin | EQQALQTVCLK |
| P05452 | TetraneCtin | SRLDTLAQEVALLK |
| P05452 | TetraneCtin | DQLPYICQFGIV |
| P05452 | TetraneCtin | LDTLAQEVALLK |
| P05452 | TetraneCtin | TFHEASEDCISR |
| O60635 | Tetraspanin-1 | VEGCFNQLLYDIR |
| Q5R3I4 | TetratriCopeptide repeat protein 38 | DVGLPLCQALVEAEDGNPDRVLELLLPIR |
| Q9BRA2 | Thioredoxin domain-Containing protein 17 | SWCPDCVQAEPVVR |
| Q9BRA2 | Thioredoxin domain-Containing protein 17 | VTAVPTLLK |
| Q9BRA2 | Thioredoxin domain-Containing protein 17 | LVESECLQANLVEMLFSED |
| P10599 | Thioredoxin | LVVVDFSATWCGPCK |
| P10599 | Thioredoxin | TAFQEALDAAGDK |
| P10599 | Thioredoxin | VGEFSGANK |
| P10599 | Thioredoxin | TAFQEALDAAGDKLVVVDFSATWCGPCK |
| P10599 | Thioredoxin | MIKPFFHSLSEK |
| P10599 | Thioredoxin | EKLEATINELV |
| P10599 | Thioredoxin | LEATINELV |
| Q16881 | Thioredoxin reduCtase 1, CytoplasmiC | WGLGGTCVNVGCIPK |
| Q16881 | Thioredoxin reduCtase 1, CytoplasmiC | VMVLDFVTPTPLGTR |
| O43396 | Thioredoxin-like protein 1 | VGVKPVGSDPDFQPELSGAGSR |
| O43396 | Thioredoxin-like protein 1 | VRIDQYQGADAVGLEEK |
| O43396 | Thioredoxin-like protein 1 | IKQHLENDPGSNEDTDIPK |
| O43396 | Thioredoxin-like protein 1 | GYMDLMPFINK |
| O43396 | Thioredoxin-like protein 1 | IFINLPR |
| O43396 | Thioredoxin-like protein 1 | AGCECLNESDEHGFDNCLR |
| P26639 | Threonyl-tRNA synthetase, CytoplasmiC | IYGISFPDPK |
| P26639 | Threonyl-tRNA synthetase, CytoplasmiC | QVMVVPVGPTCDEYAQK |
| P26639 | Threonyl-tRNA synthetase, CytoplasmiC | MGGEEKPIGAGEEK |
| P26639 | Threonyl-tRNA synthetase, CytoplasmiC | TTPYQIACGISQGLADNTVIAK |
| P63313 | ThymOSin beta-10 | NTLPTKETIEQEK |
| P63313 | ThymOSin beta-10 | ETIEQEKR |
| A8MW06 | ThymOSin beta-4-like protein 3 | NPLPSKETIEQEK |
| A8MW06 | ThymOSin beta-4-like protein 3 | TETQEKNPLPSK |
| A8MW06 | ThymOSin beta-4-like protein 3 | ETIEQEKQAGES |
| P05543 | Thyroxine-binding globulin | EGQMESVEAAMSSK |
| P05543 | Thyroxine-binding globulin | NALALFVLPK |
| P05543 | Thyroxine-binding globulin | AQWANPFDPSKTEDSSSFLIDK |
| P05543 | Thyroxine-binding globulin | GWVDLFVPK |
| P05543 | Thyroxine-binding globulin | MGIQHAYSENADFSGLTEDNGLK |
| P05543 | Thyroxine-binding globulin | SILFLGK |
| P05543 | Thyroxine-binding globulin | FSISATYDLGATLLK |
| P37837 | Transaldolase | ALAGCDFLTISPK |
| P37837 | Transaldolase | LLGELLQDNAK |
| P37837 | Transaldolase | LFVLFGAEILK |
| P37837 | Transaldolase | AAQASDLEK |
| P37837 | Transaldolase | FAADAVKLER |
| P37837 | Transaldolase | VSTEVDAR |
| P37837 | Transaldolase | MESALDQLK |
| P37837 | Transaldolase | WLHNEDQMAVEK |
| P37837 | Transaldolase | QRMESALDQLK |
| P37837 | Transaldolase | LSDGIRK |
| P37837 | Transaldolase | TIVMGASFR |
| P37837 | Transaldolase | SYEPLEDPGVK |
| P37837 | Transaldolase | KFAADAVK |
| P37837 | Transaldolase | LIELYK |
| P37837 | Transaldolase | KLGGSQEDQIK |
| Q969E4 | TransCription elongation faCtor A protein-like 3 | SEGEGKPQGEGKPASQAKPESQPR |
| Q15370 | TransCription elongation faCtor B polypeptide 2 | TLGECGFTSQTARPQAPATVGLAFR |
| Q15370 | TransCription elongation faCtor B polypeptide 2 | IVEGILK |
| Q96K17 | TransCription faCtor BTF3 homolog 4 | APKPEDIDEEDDDVPDLVENFDEASKNEAN |
| Q15582 | Transforming growth faCtor-beta-induCed protein ig-h3 | GCPAALPLSNLYETLGVVGSTTTQLYTDRTEK |
| Q15582 | Transforming growth faCtor-beta-induCed protein ig-h3 | LTLLAPLNSVFK |
| Q15582 | Transforming growth faCtor-beta-induCed protein ig-h3 | VISTITNNIQQIIEIEDTFETLR |
| P61586 | Transforming protein RhoA | TCLLIVFSK |
| P61586 | Transforming protein RhoA | IGAFGYMECSAK |
| P61586 | Transforming protein RhoA | DQFPEVYVPTVFENYVADIEVDGK |
| P61586 | Transforming protein RhoA | KLVIVGDGACGK |
| P61586 | Transforming protein RhoA | LVIVGDGACGK |
| P61586 | Transforming protein RhoA | HFCPNVPIILVGNKK |
| P61586 | Transforming protein RhoA | EVFEMATR |
| P37802 | Transgelin-2 | YGINTTDIFQTVDLWEGK |
| P37802 | Transgelin-2 | QMEQISQFLQAAER |
| P37802 | Transgelin-2 | DDGLFSGDPNWFPK |
| P37802 | Transgelin-2 | NFSDNQLQEGK |
| P37802 | Transgelin-2 | NVIGLQMGTNR |
| P37802 | Transgelin-2 | QYDADLEQILIQWITTQCRK |
| P37802 | Transgelin-2 | DGTVLCELINALYPEGQAPVKK |
| P37802 | Transgelin-2 | QYDADLEQILIQWITTQCR |
| P37802 | Transgelin-2 | GASQAGMTGYGMPR |
| P37802 | Transgelin-2 | TLMNLGGLAVAR |
| P37802 | Transgelin-2 | IQASTMAFK |
| P37802 | Transgelin-2 | NMACVQR |
| P29401 | Transketolase | NMAEQIIQEIYSQIQSK |
| P29401 | Transketolase | QAFTDVATGSLGQGLGAACGMAYTGK |
| P29401 | Transketolase | ILATPPQEDAPSVDIANIR |
| P29401 | Transketolase | GHAAPILYAVWAEAGFLAEAELLNLRK |
| P29401 | Transketolase | TSRPENAIIYNNNEDFQVGQAK |
| P29401 | Transketolase | MAAISESNINLCGSHCGVSIGEDGPSQMALEDLAMFR |
| P29401 | Transketolase | TVPFCSTFAAFFTR |
| P29401 | Transketolase | AVELAANTK |
| P29401 | Transketolase | SGKPAELLK |
| P29401 | Transketolase | LGHASDRIIALDGDTK |
| P29401 | Transketolase | MFGIDRDAIAQAVR |
| P29401 | Transketolase | GRGITGVEDK |
| P29401 | Transketolase | VYCLLGDGELSEGSVWEAMAFASIYK |
| P29401 | Transketolase | LDNLVAILDINR |
| P29401 | Transketolase | LGQSDPAPLQHQMDIYQK |
| P29401 | Transketolase | ISSDLDGHPVPK |
| P29401 | Transketolase | KLILDSAR |
| P29401 | Transketolase | LQALKDTANR |
| P29401 | Transketolase | KISSDLDGHPVPK |
| P29401 | Transketolase | SVPTSTVFYPSDGVATEK |
| P29401 | Transketolase | KAYGQALAK |
| P29401 | Transketolase | HQPTAIIAK |
| P29401 | Transketolase | GITGVEDKESWHGKPLPK |
| P29401 | Transketolase | SKDDQVTVIGAGVTLHEALAAAELLK |
| P29401 | Transketolase | ILTVEDHYYEGGIGEAVSSAVVGEPGITVTHLAVNR |
| P29401 | Transketolase | AYGQALAK |
| P13693 | Translationally-Controlled tumor protein | EIADGLCLEVEGK |
| P13693 | Translationally-Controlled tumor protein | DLISHDEMFSDIYK |
| P13693 | Translationally-Controlled tumor protein | VKPFMTGAAEQIK |
| P13693 | Translationally-Controlled tumor protein | TEGNIDDSLIGGNASAEGPEGEGTESTVITGVDIVMNHHLQETSFTK |
| P13693 | Translationally-Controlled tumor protein | GKLEEQRPER |
| Q15631 | Translin | ETAAACVEK |
| Q15631 | Translin | VVQSLEQTAR |
| Q15631 | Translin | GFNKETAAACVEK |
| Q15631 | Translin | EILTLLQGVHQGAGFQDIPKR |
| Q99598 | Translin-assoCiated protein X | ITSAPDMEDILTESEIKLDGVR |
| Q24JP5 | Transmembrane protein 132A | AEELVNTAPLTGVPQHVPVR |
| P22102 | TrifunCtional purine biOSynthetiC protein adenOSine-3 | FGDPECQVILPLLK |
| O14773 | Tripeptidyl-peptidase 1 | VPIPWVSGTSASTPVFGGILSLINEHR |
| P23381 | Tryptophanyl-tRNA synthetase, CytoplasmiC | PNSEPASLLELFNSIATQGELVR |
| P23381 | Tryptophanyl-tRNA synthetase, CytoplasmiC | DLTLDQAYSYAVENAK |
| P23381 | Tryptophanyl-tRNA synthetase, CytoplasmiC | ISFPAIQAAPSFSNSFPQIFR |
| P23381 | Tryptophanyl-tRNA synthetase, CytoplasmiC | AGNASKDEIDSAVK |
| P23381 | Tryptophanyl-tRNA synthetase, CytoplasmiC | AAAGEDYKADCPPGNPAPTSNHGPDATEAEEDFVDPWTVQTSSAK |
| P23381 | Tryptophanyl-tRNA synthetase, CytoplasmiC | MSASDPNSSIFLTDTAK |
| P23381 | Tryptophanyl-tRNA synthetase, CytoplasmiC | DRTDIQCLIPCAIDQDPYFR |
| Q15714 | TSC22 domain family protein 1 | NSQLEQENNLLK |
| Q71U36 | Tubulin alpha-1A Chain | LIGQIVSSITASLR |
| Q71U36 | Tubulin alpha-1A Chain | RTIQFVDWCPTGFK |
| Q71U36 | Tubulin alpha-1A Chain | AVCMLSNTTAIAEAWAR |
| Q71U36 | Tubulin alpha-1A Chain | AYHEQLSVAEITNACFEPANQMVK |
| P68363 | Tubulin alpha-1B Chain | RSIQFVDWCPTGFK |
| P68363 | Tubulin alpha-1B Chain | LISQIVSSITASLR |
| Q9BQE3 | Tubulin alpha-1C Chain | AVCMLSNTTAVAEAWAR |
| Q9BQE3 | Tubulin alpha-1C Chain | AYHEQLTVAEITNACFEPANQMVK |
| Q9BQE3 | Tubulin alpha-1C Chain | LISQIVSSITASLR |
| P07437 | Tubulin beta Chain | MAVTFIGNSTAIQELFK |
| P07437 | Tubulin beta Chain | FWEVISDEHGIDPTGTYHGDSDLQLDR |
| P07437 | Tubulin beta Chain | ISVYYNEATGGK |
| P07437 | Tubulin beta Chain | ALTVPELTQQVFDAK |
| P07437 | Tubulin beta Chain | AILVDLEPGTMDSVR |
| P07437 | Tubulin beta Chain | MAVTFIGNSTAIQELFKR |
| P68371 | Tubulin beta-2C Chain | MSATFIGNSTAIQELFKR |
| P68371 | Tubulin beta-2C Chain | INVYYNEATGGK |
| Q13509 | Tubulin beta-3 Chain | LHFFMPGFAPLTAR |
| Q13509 | Tubulin beta-3 Chain | ECENCDCLQGFQLTHSLGGGTGSGMGTLLISK |
| P04350 | Tubulin beta-4 Chain | mAATFIGNSTAIQELFK |
| Q9BUF5 | Tubulin beta-6 Chain | mASTFIGNSTAIQELFK |
| Q9BW30 | Tubulin polymerization-promoting protein family member 3 | QDILDDSGYVSAYK |
| O75347 | Tubulin-speCifiC Chaperone A | LVLDSVKLEA |
| Q9Y275 | Tumor neCrOSis faCtor ligand superfamily member 13B | GSALEEKENK |
| P55327 | Tumor protein D52 | VGGTKPAGGDFGEVLNSAANASATTTEPLPEK |
| P55327 | Tumor protein D52 | ASAAFSSVGSVITK |
| P55327 | Tumor protein D52 | VEEEIQTLSQVLAAK |
| P55327 | Tumor protein D52 | TSETLSQAGQK |
| P55327 | Tumor protein D52 | GWQDVTATSAYKK |
| P55327 | Tumor protein D52 | TDPVPEEGEDVAATISATETLSEEEQEELRR |
| P55327 | Tumor protein D52 | ELAKVEEEIQTLSQVLAAK |
| Q16890 | Tumor protein D53 | ATAAFSNVGTAISK |
| Q16890 | Tumor protein D53 | LGMNLMNELK |
| O43399 | Tumor protein D54 | GLLSDSMTDVPVDTGVAAR |
| O43399 | Tumor protein D54 | TQETLSQAGQK |
| O43399 | Tumor protein D54 | LGLSTLGELK |
| Q6IBS0 | Twinfilin-2 | AVLPLLDAQQPCYLLYR |
| O15042 | U2 snRNP-assoCiated SURP motif-Containing protein | FGPLASVK |
| P62310 | U6 snRNA-assoCiated Sm-like protein LSm3 | GDGVVLVAPPLRVG |
| P45974 | Ubiquitin Carboxyl-terminal hydrolase 5 | IGEWELIQESGVPLKPLFGPGYTGIR |
| P45974 | Ubiquitin Carboxyl-terminal hydrolase 5 | IVILPDYLEIAR |
| P45974 | Ubiquitin Carboxyl-terminal hydrolase 5 | AQVPFSSCLEAYGAPEQVDDFWSTALQAK |
| Q96FW1 | Ubiquitin thioesterase OTUB1 | EYAEDDNIYQQK |
| Q96FW1 | Ubiquitin thioesterase OTUB1 | AFGFSHLEALLDDSK |
| Q96FW1 | Ubiquitin thioesterase OTUB1 | EFCQQEVEPMCK |
| Q96FW1 | Ubiquitin thioesterase OTUB1 | LLTSGYLQR |
| P68036 | Ubiquitin-Conjugating enzyme E2 L3 | IEINFPAEYPFKPPK |
| P68036 | Ubiquitin-Conjugating enzyme E2 L3 | NIQVDEANLLTWQGLIVPDNPPYDK |
| P68036 | Ubiquitin-Conjugating enzyme E2 L3 | TDQVIQSLIALVNDPQPEHPLR |
| P61088 | Ubiquitin-Conjugating enzyme E2 N | TNEAQAIETAR |
| P61088 | Ubiquitin-Conjugating enzyme E2 N | ICLDILK |
| Q15819 | Ubiquitin-Conjugating enzyme E2 variant 2 | LLEELEEGQK |
| P61960 | Ubiquitin-fold modifier 1 | FAAEEFKVPAATSAIITNDGIGINPAQTAGNVFLK |
| P61960 | Ubiquitin-fold modifier 1 | VLSVPESTPFTAVLK |
| P22314 | Ubiquitin-like modifier-aCtivating enzyme 1 | YFLVGAGAIGCELLK |
| P22314 | Ubiquitin-like modifier-aCtivating enzyme 1 | VLGPYTFSICDTSNFSDYIR |
| P22314 | Ubiquitin-like modifier-aCtivating enzyme 1 | LAYVAAGDLAPINAFIGGLAAQEVMK |
| P22314 | Ubiquitin-like modifier-aCtivating enzyme 1 | AAVATFLQSVQVPEFTPK |
| P22314 | Ubiquitin-like modifier-aCtivating enzyme 1 | KPLLESGTLGTK |
| P22314 | Ubiquitin-like modifier-aCtivating enzyme 1 | DNPGVVTCLDEAR |
| P22314 | Ubiquitin-like modifier-aCtivating enzyme 1 | VGPDTERIYDDDFFQNLDGVANALDNVDAR |
| P22314 | Ubiquitin-like modifier-aCtivating enzyme 1 | SLVASLAEPDFVVTDFAK |
| P22314 | Ubiquitin-like modifier-aCtivating enzyme 1 | IYDDDFFQNLDGVANALDNVDAR |
| P22314 | Ubiquitin-like modifier-aCtivating enzyme 1 | NEEDAAELVALAQAVNAR |
| P22314 | Ubiquitin-like modifier-aCtivating enzyme 1 | IHVSDQELQSANASVDDSRLEELK |
| P22314 | Ubiquitin-like modifier-aCtivating enzyme 1 | ALPAVQQNNLDEDLIR |
| P22314 | Ubiquitin-like modifier-aCtivating enzyme 1 | YDGQVAVFGSDLQEK |
| P22314 | Ubiquitin-like modifier-aCtivating enzyme 1 | LAGTQPLEVLEAVQR |
| P22314 | Ubiquitin-like modifier-aCtivating enzyme 1 | LQTSSVLVSGLR |
| P22314 | Ubiquitin-like modifier-aCtivating enzyme 1 | DEFEGLFKQPAENVNQYLTDPK |
| P22314 | Ubiquitin-like modifier-aCtivating enzyme 1 | LKSDTAAAAVR |
| P22314 | Ubiquitin-like modifier-aCtivating enzyme 1 | GNVQVVIPFLTESYSSSQDPPEK |
| P22314 | Ubiquitin-like modifier-aCtivating enzyme 1 | QNRYDGQVAVFGSDLQEK |
| P22314 | Ubiquitin-like modifier-aCtivating enzyme 1 | VGEFCHNR |
| P22314 | Ubiquitin-like modifier-aCtivating enzyme 1 | LDQPMTEIVSR |
| P22314 | Ubiquitin-like modifier-aCtivating enzyme 1 | NIILGGVK |
| P22314 | Ubiquitin-like modifier-aCtivating enzyme 1 | NRAEVSQPR |
| P22314 | Ubiquitin-like modifier-aCtivating enzyme 1 | ALPAVQQNNLDEDLIRK |
| P22314 | Ubiquitin-like modifier-aCtivating enzyme 1 | RLQTSSVLVSGLR |
| P22314 | Ubiquitin-like modifier-aCtivating enzyme 1 | KLAYVAAGDLAPINAFIGGLAAQEVMK |
| P22314 | Ubiquitin-like modifier-aCtivating enzyme 1 | NGFLNLALPFFGFSEPLAAPR |
| P22314 | Ubiquitin-like modifier-aCtivating enzyme 1 | GLGVEIAK |
| P05161 | Ubiquitin-like protein ISG15 | IGVHAFQQR |
| P05161 | Ubiquitin-like protein ISG15 | SSTYEVR |
| P05161 | Ubiquitin-like protein ISG15 | LTQTVAHLK |
| O60701 | UDP-gluCOSe 6-dehydrogenase | LAANAFLAQR |
| O60701 | UDP-gluCOSe 6-dehydrogenase | INAWNSPTLPIYEPGLK |
| O60701 | UDP-gluCOSe 6-dehydrogenase | ILTTNTWSSELSK |
| O60701 | UDP-gluCOSe 6-dehydrogenase | NLFFSTNIDDAIK |
| O60701 | UDP-gluCOSe 6-dehydrogenase | ISSINSISALCEATGADVEEVATAIGMDQR |
| O60701 | UDP-gluCOSe 6-dehydrogenase | VTVVDVNESR |
| O60701 | UDP-gluCOSe 6-dehydrogenase | VLIGGDETPEGQR |
| O60701 | UDP-gluCOSe 6-dehydrogenase | ICCIGAGYVGGPTCSVIAHMCPEIR |
| O60701 | UDP-gluCOSe 6-dehydrogenase | EQIVVDLSHPGVSEDDQVSR |
| O60701 | UDP-gluCOSe 6-dehydrogenase | YIEACAR |
| O60701 | UDP-gluCOSe 6-dehydrogenase | IIDSLFNTVTDKK |
| O60701 | UDP-gluCOSe 6-dehydrogenase | IFDANTKPNLNLQVLSNPEFLAEGTAIK |
| P30085 | UMP-CMP kinase | SVDEVFDEVVQIFDK |
| P30085 | UMP-CMP kinase | SVDEVFDEVVQIFDKEG |
| P30085 | UMP-CMP kinase | KNPDSQYGELIEK |
| P30085 | UMP-CMP kinase | EMDQTMAANAQK |
| P30085 | UMP-CMP kinase | SDDNRESLEK |
| P30085 | UMP-CMP kinase | FLIDGFPR |
| Q9H8K7 | UnCharaCterized protein C10orf88 | IILYK |
| Q9BQ61 | UnCharaCterized protein C19orf43 | EAPGPAGGGGGGSR |
| Q86XI8 | UnCharaCterized protein C19orf68 | GSQLEDQALR |
| Q6P047 | UnCharaCterized protein C8orf74 | RADVLLLK |
| Q9HB07 | UPF0160 protein MYG1, mitoChondrial | FQVDPSGEIVELAK |
| Q9HB07 | UPF0160 protein MYG1, mitoChondrial | ALVEEALAQR |
| Q9HB07 | UPF0160 protein MYG1, mitoChondrial | LPLPEPWR |
| Q9H993 | UPF0364 protein C6orf211 | FVDTDIWNQYLEYQQSLLNESDGK |
| Q9H993 | UPF0364 protein C6orf211 | HGEEGVEAEK |
| Q9H7C9 | UPF0366 protein C11orf67 | EYNALVAQGVR |
| Q969H8 | UPF0556 protein C19orf10 | SYLYFTQFK |
| P06132 | Uroporphyrinogen deCarboxylase | TVTLQGNLDPCALYASEEEIGQLVK |
| P54725 | UV exCision repair protein RAD23 homolog A | QVIQQNPALLPALLQQLGQENPQLLQQISR |
| P54727 | UV exCision repair protein RAD23 homolog B | NFVVVMVTKPK |
| P54727 | UV exCision repair protein RAD23 homolog B | MQVTLK |
| P54727 | UV exCision repair protein RAD23 homolog B | TLQQQTFKIDIDPEETVK |
| P54727 | UV exCision repair protein RAD23 homolog B | NENLAANFLLQQNFDED |
| P54727 | UV exCision repair protein RAD23 homolog B | QIIQQNPSLLPALLQQIGR |
| Q96QK1 | VaCuolar protein sorting-assoCiated protein 35 | PTTQQSPQDEQEK |
| Q96QK1 | VaCuolar protein sorting-assoCiated protein 35 | ESPESEGPIYEGLIL |
| Q9H9H4 | VaCuolar protein sorting-assoCiated protein 37B | FLDGELPLDSFIDVYQSK |
| P13611 | VersiCan Core protein | GRVSVPTHPEAVGDASLTVVK |
| P13611 | VersiCan Core protein | AQCGGGLLGVR |
| P13611 | VersiCan Core protein | LATVGELQAAWR |
| P13611 | VersiCan Core protein | LLASDAGLYR |
| P13611 | VersiCan Core protein | YTLNFEAAQK |
| P13611 | VersiCan Core protein | FTFEEAAKECENQDAR |
| P13611 | VersiCan Core protein | LQGAHLTSILSHEEQMFVNR |
| P13611 | VersiCan Core protein | LGEPNYGAEIR |
| P13611 | VersiCan Core protein | YEINSLIR |
| P13611 | VersiCan Core protein | SPQETYDVYCYVDHLDGDVFHLTVPSK |
| P18206 | VinCulin | AIPDLTAPVAAVQAAVSNLVR |
| P18206 | VinCulin | LVQAAQMLQSDPYSVPARDYLIDGSR |
| P18206 | VinCulin | AVAGNISDPGLQK |
| P18206 | VinCulin | AQQVSQGLDVLTAK |
| P18206 | VinCulin | MTGLVDEAIDTK |
| P18206 | VinCulin | TNISDEESEQATEMLVHNAQNLMQSVK |
| P18206 | VinCulin | MLGQMTDQVADLR |
| P18206 | VinCulin | GWLRDPSASPGDAGEQAIR |
| P18206 | VinCulin | VMLVNSMNTVK |
| P18206 | VinCulin | ALASIDSK |
| P18206 | VinCulin | QQELTHQEHR |
| P18206 | VinCulin | AGEVINQPMMMAAR |
| P18206 | VinCulin | MQEAMTQEVSDVFSDTTTPIK |
| P18206 | VinCulin | STVEGIQASVK |
| P18206 | VinCulin | ELLPVLISAMK |
| P18206 | VinCulin | MSAEINEIIR |
| P18206 | VinCulin | GQGSSPVAMQK |
| P18206 | VinCulin | KLEAMTNSK |
| P18206 | VinCulin | ALASQLQDSLK |
| P18206 | VinCulin | QVATALQNLQTK |
| P18206 | VinCulin | LLAVAATAPPDAPNREEVFDER |
| P07225 | Vitamin K-dependent protein S | HCLVTVEK |
| P07225 | Vitamin K-dependent protein S | KVESELIKPINPR |
| P07225 | Vitamin K-dependent protein S | ITTGGDVINNGLWNMVSVEELEHSISIK |
| P04004 | VitroneCtin | DVWGIEGPIDAAFTR |
| P04004 | VitroneCtin | LIRDVWGIEGPIDAAFTR |
| P04004 | VitroneCtin | TSAGTRQPQFISR |
| P04004 | VitroneCtin | IYISGMAPRPSLAK |
| P04004 | VitroneCtin | GQYCYELDEK |
| P04004 | VitroneCtin | GRCTEGFNVDK |
| P04004 | VitroneCtin | CQCDELCSYYQSCCTDYTAECKPQVTR |
| P04004 | VitroneCtin | SIAQYWLGCPAPGHL |
| P04004 | VitroneCtin | DWHGVPGQVDAAMAGR |
| P21281 | V-type proton ATPase subunit B, brain isoform | IYPEEMIQTGISAIDGMNSIAR |
| O75083 | WD repeat-Containing protein 1 | LATGSDDNCAAFFEGPPFK |
| O75083 | WD repeat-Containing protein 1 | YAPSGFYIASGDVSGK |
| O75083 | WD repeat-Containing protein 1 | MTVDESGQLISCSMDDTVR |
| O75083 | WD repeat-Containing protein 1 | GPVTDVAYSHDGAFLAVCDASK |
| O75083 | WD repeat-Containing protein 1 | LYSILGTTLKDEGK |
| O75083 | WD repeat-Containing protein 1 | CFSIDNPGYEPEVVAVHPGGDTVAIGGVDGNVR |
| O75083 | WD repeat-Containing protein 1 | VINSVDIK |
| O75083 | WD repeat-Containing protein 1 | IAVVGEGR |
| O75083 | WD repeat-Containing protein 1 | VFASLPQVER |
| O75083 | WD repeat-Containing protein 1 | IIGGDPK |
| O75083 | WD repeat-Containing protein 1 | CVILR |
| O75083 | WD repeat-Containing protein 1 | LDVQPK |
| Q9NQW7 | Xaa-Pro aminopeptidase 1 | GSLTFEPLTLVPIQTK |
| P12955 | Xaa-Pro dipeptidase | GVNTDSGSVCR |
| P12955 | Xaa-Pro dipeptidase | VPLALFALNR |
| P12955 | Xaa-Pro dipeptidase | YAVDDVQYVDEIASVLTSQKPSVLLTLR |
| A4UGR9 | Xin aCtin-binding repeat-Containing protein 2 | CLEVK |
| P13010 | X-ray repair CrOSs-Complementing protein 5 | YAPTEAQLNAVDALIDSMSLAK |
| P13010 | X-ray repair CrOSs-Complementing protein 5 | LTIGSNLSIR |
| P13010 | X-ray repair CrOSs-Complementing protein 5 | DKPSGDTAAVFEEGGDVDDLLDMI |
| P13010 | X-ray repair CrOSs-Complementing protein 5 | EEASGSSVTAEEAK |
| P13010 | X-ray repair CrOSs-Complementing protein 5 | HLMLPDFDLLEDIESK |
| P13010 | X-ray repair CrOSs-Complementing protein 5 | KYAPTEAQLNAVDALIDSMSLAK |
| P12956 | X-ray repair CrOSs-Complementing protein 6 | NLEALALDLMEPEQAVDLTLPK |
| P46937 | Yorkie homolog | AHSSPASLQLGAVSPGTLTPTGVVSGPAATPTAQHLR |
| Q9Y5A9 | YTH domain family protein 2 | LDAAYR |
| P25311 | ZinC-alpha-2-glyCoprotein | HVEDVPAFQALGSLNDLQFFR |
| P25311 | ZinC-alpha-2-glyCoprotein | YSLTYIYTGLSK |
| P25311 | ZinC-alpha-2-glyCoprotein | AYLEEECPATLRK |
| P25311 | ZinC-alpha-2-glyCoprotein | NILDRQDPPSVVVTSHQAPGEK |
| P25311 | ZinC-alpha-2-glyCoprotein | QKWEAEPVYVQR |
| P25311 | ZinC-alpha-2-glyCoprotein | CLAYDFYPGK |
| P25311 | ZinC-alpha-2-glyCoprotein | IDVHWTR |
| P25311 | ZinC-alpha-2-glyCoprotein | QVEGMEDWKQDSQLQK |
| P25311 | ZinC-alpha-2-glyCoprotein | AYLEEECPATLR |
| P25311 | ZinC-alpha-2-glyCoprotein | SSGAFWK |
| Q15942 | Zyxin | FSPGAPGGSGSQPNQK |
| Q15942 | Zyxin | QNVAVNELCGR |
| Q15942 | Zyxin | EVEELEQLTQQLMQDMEHPQR |
| Q15942 | Zyxin | QHPVPPPAQNQNQVR |
| *** In bold, the 158 proteins identified out of the initial list of 506 biomarker candidates.** | | |

**Supplementary Table 2.** List of precursors and related fragment ions extracted from PRM acquisition.

| **Protein Name** | **Peptide Sequence** | **Light/Heavy peptide** | **Precursor Mz** | **Precursor Charge** | **Product Mz** | **Product Charge** | **Fragment Ion** |
| --- | --- | --- | --- | --- | --- | --- | --- |
| sp|P17931|LEG3_HUMAN | IALDFQR | Light | 431,7 | 2 | 749,39 | 1 | y6 |
| sp|P17931|LEG3_HUMAN | IALDFQR | Light | 431,7 | 2 | 678,36 | 1 | y5 |
| sp|P17931|LEG3_HUMAN | IALDFQR | Light | 431,7 | 2 | 565,27 | 1 | y4 |
| sp|P17931|LEG3_HUMAN | IALDFQR | Light | 431,7 | 2 | 450,25 | 1 | y3 |
| sp|P17931|LEG3_HUMAN | IALDFQR | Light | 431,7 | 2 | 339,68 | 2 | y5 |
| sp|P17931|LEG3_HUMAN | IALDFQR | Heavy | 436,7 | 2 | 759,40 | 1 | y6 |
| sp|P17931|LEG3_HUMAN | IALDFQR | Heavy | 436,7 | 2 | 688,37 | 1 | y5 |
| sp|P17931|LEG3_HUMAN | IALDFQR | Heavy | 436,7 | 2 | 575,28 | 1 | y4 |
| sp|P17931|LEG3_HUMAN | IALDFQR | Heavy | 436,7 | 2 | 460,25 | 1 | y3 |
| sp|P17931|LEG3_HUMAN | IALDFQR | Heavy | 436,7 | 2 | 344,69 | 2 | y5 |
| sp|P17931|LEG3_HUMAN | GNDVAFHFNPR | Light | 425,2 | 3 | 551,78 | 2 | y9 |
| sp|P17931|LEG3_HUMAN | GNDVAFHFNPR | Light | 425,2 | 3 | 494,26 | 2 | y8 |
| sp|P17931|LEG3_HUMAN | GNDVAFHFNPR | Light | 425,2 | 3 | 444,73 | 2 | y7 |
| sp|P17931|LEG3_HUMAN | GNDVAFHFNPR | Light | 425,2 | 3 | 409,21 | 2 | y6 |
| sp|P17931|LEG3_HUMAN | GNDVAFHFNPR | Light | 425,2 | 3 | 335,67 | 2 | y5 |
| sp|P17931|LEG3_HUMAN | GNDVAFHFNPR | Heavy | 428,5 | 3 | 556,78 | 2 | y9 |
| sp|P17931|LEG3_HUMAN | GNDVAFHFNPR | Heavy | 428,5 | 3 | 499,27 | 2 | y8 |
| sp|P17931|LEG3_HUMAN | GNDVAFHFNPR | Heavy | 428,5 | 3 | 449,73 | 2 | y7 |
| sp|P17931|LEG3_HUMAN | GNDVAFHFNPR | Heavy | 428,5 | 3 | 414,21 | 2 | y6 |
| sp|P17931|LEG3_HUMAN | GNDVAFHFNPR | Heavy | 428,5 | 3 | 340,68 | 2 | y5 |
| sp|P10451|OSTP_HUMAN | AIPVAQDLNAPSDWDSR | Light | 928,0 | 2 | 1047,45 | 1 | y9 |
| sp|P10451|OSTP_HUMAN | AIPVAQDLNAPSDWDSR | Light | 928,0 | 2 | 933,41 | 1 | y8 |
| sp|P10451|OSTP_HUMAN | AIPVAQDLNAPSDWDSR | Light | 928,0 | 2 | 862,37 | 1 | y7 |
| sp|P10451|OSTP_HUMAN | AIPVAQDLNAPSDWDSR | Light | 928,0 | 2 | 835,89 | 2 | y15 |
| sp|P10451|OSTP_HUMAN | AIPVAQDLNAPSDWDSR | Light | 928,0 | 2 | 993,54 | 1 | b10 |
| sp|P10451|OSTP_HUMAN | AIPVAQDLNAPSDWDSR | Heavy | 933,0 | 2 | 1057,46 | 1 | y9 |
| sp|P10451|OSTP_HUMAN | AIPVAQDLNAPSDWDSR | Heavy | 933,0 | 2 | 943,41 | 1 | y8 |
| sp|P10451|OSTP_HUMAN | AIPVAQDLNAPSDWDSR | Heavy | 933,0 | 2 | 872,38 | 1 | y7 |
| sp|P10451|OSTP_HUMAN | AIPVAQDLNAPSDWDSR | Heavy | 933,0 | 2 | 840,90 | 2 | y15 |
| sp|P10451|OSTP_HUMAN | AIPVAQDLNAPSDWDSR | Heavy | 933,0 | 2 | 993,54 | 1 | b10 |
| sp|P10451|OSTP_HUMAN | ANDESNEHSDVIDSQELSK | Light | 706,3 | 3 | 919,47 | 1 | y8 |
| sp|P10451|OSTP_HUMAN | ANDESNEHSDVIDSQELSK | Light | 706,3 | 3 | 806,39 | 1 | y7 |
| sp|P10451|OSTP_HUMAN | ANDESNEHSDVIDSQELSK | Light | 706,3 | 3 | 908,91 | 2 | y16 |
| sp|P10451|OSTP_HUMAN | ANDESNEHSDVIDSQELSK | Light | 706,3 | 3 | 844,39 | 2 | y15 |
| sp|P10451|OSTP_HUMAN | ANDESNEHSDVIDSQELSK | Light | 706,3 | 3 | 301,11 | 1 | b3 |
| sp|P10451|OSTP_HUMAN | ANDESNEHSDVIDSQELSK | Heavy | 709,0 | 3 | 927,49 | 1 | y8 |
| sp|P10451|OSTP_HUMAN | ANDESNEHSDVIDSQELSK | Heavy | 709,0 | 3 | 814,40 | 1 | y7 |
| sp|P10451|OSTP_HUMAN | ANDESNEHSDVIDSQELSK | Heavy | 709,0 | 3 | 912,92 | 2 | y16 |
| sp|P10451|OSTP_HUMAN | ANDESNEHSDVIDSQELSK | Heavy | 709,0 | 3 | 848,40 | 2 | y15 |
| sp|P10451|OSTP_HUMAN | ANDESNEHSDVIDSQELSK | Heavy | 709,0 | 3 | 301,11 | 1 | b3 |
| sp|P35222|CTNB1_HUMAN | LLNDEDQVVVNK | Light | 693,4 | 2 | 1159,56 | 1 | y10 |
| sp|P35222|CTNB1_HUMAN | LLNDEDQVVVNK | Light | 693,4 | 2 | 1045,52 | 1 | y9 |
| sp|P35222|CTNB1_HUMAN | LLNDEDQVVVNK | Light | 693,4 | 2 | 459,29 | 1 | y4 |
| sp|P35222|CTNB1_HUMAN | LLNDEDQVVVNK | Light | 693,4 | 2 | 360,22 | 1 | y3 |
| sp|P35222|CTNB1_HUMAN | LLNDEDQVVVNK | Light | 693,4 | 2 | 580,28 | 2 | y10 |
| sp|P35222|CTNB1_HUMAN | LLNDEDQVVVNK | Heavy | 697,4 | 2 | 1167,57 | 1 | y10 |
| sp|P35222|CTNB1_HUMAN | LLNDEDQVVVNK | Heavy | 697,4 | 2 | 1053,53 | 1 | y9 |
| sp|P35222|CTNB1_HUMAN | LLNDEDQVVVNK | Heavy | 697,4 | 2 | 467,31 | 1 | y4 |
| sp|P35222|CTNB1_HUMAN | LLNDEDQVVVNK | Heavy | 697,4 | 2 | 368,24 | 1 | y3 |
| sp|P35222|CTNB1_HUMAN | LLNDEDQVVVNK | Heavy | 697,4 | 2 | 584,29 | 2 | y10 |
| sp|P35222|CTNB1_HUMAN | LVQLLVR | Light | 420,8 | 2 | 727,48 | 1 | y6 |
| sp|P35222|CTNB1_HUMAN | LVQLLVR | Light | 420,8 | 2 | 628,41 | 1 | y5 |
| sp|P35222|CTNB1_HUMAN | LVQLLVR | Light | 420,8 | 2 | 500,36 | 1 | y4 |
| sp|P35222|CTNB1_HUMAN | LVQLLVR | Light | 420,8 | 2 | 387,27 | 1 | y3 |
| sp|P35222|CTNB1_HUMAN | LVQLLVR | Light | 420,8 | 2 | 341,22 | 1 | b3 |
| sp|P35222|CTNB1_HUMAN | LVQLLVR | Heavy | 425,8 | 2 | 737,49 | 1 | y6 |
| sp|P35222|CTNB1_HUMAN | LVQLLVR | Heavy | 425,8 | 2 | 638,42 | 1 | y5 |
| sp|P35222|CTNB1_HUMAN | LVQLLVR | Heavy | 425,8 | 2 | 510,36 | 1 | y4 |
| sp|P35222|CTNB1_HUMAN | LVQLLVR | Heavy | 425,8 | 2 | 397,28 | 1 | y3 |
| sp|P35222|CTNB1_HUMAN | LVQLLVR | Heavy | 425,8 | 2 | 341,22 | 1 | b3 |
| sp|P30041|PRDX6_HUMAN | DFTPVCTTELGR | Light | 698,3 | 2 | 1032,51 | 1 | y9 |
| sp|P30041|PRDX6_HUMAN | DFTPVCTTELGR | Light | 698,3 | 2 | 836,39 | 1 | y7 |
| sp|P30041|PRDX6_HUMAN | DFTPVCTTELGR | Light | 698,3 | 2 | 567,28 | 2 | y10 |
| sp|P30041|PRDX6_HUMAN | DFTPVCTTELGR | Light | 698,3 | 2 | 516,76 | 2 | y9 |
| sp|P30041|PRDX6_HUMAN | DFTPVCTTELGR | Light | 698,3 | 2 | 364,15 | 1 | b3 |
| sp|P30041|PRDX6_HUMAN | DFTPVCTTELGR | Heavy | 703,3 | 2 | 1042,52 | 1 | y9 |
| sp|P30041|PRDX6_HUMAN | DFTPVCTTELGR | Heavy | 703,3 | 2 | 846,40 | 1 | y7 |
| sp|P30041|PRDX6_HUMAN | DFTPVCTTELGR | Heavy | 703,3 | 2 | 572,29 | 2 | y10 |
| sp|P30041|PRDX6_HUMAN | DFTPVCTTELGR | Heavy | 703,3 | 2 | 521,76 | 2 | y9 |
| sp|P30041|PRDX6_HUMAN | DFTPVCTTELGR | Heavy | 703,3 | 2 | 364,15 | 1 | b3 |
| sp|P30041|PRDX6_HUMAN | LSILYPATTGR | Light | 596,3 | 2 | 765,39 | 1 | y7 |
| sp|P30041|PRDX6_HUMAN | LSILYPATTGR | Light | 596,3 | 2 | 602,33 | 1 | y6 |
| sp|P30041|PRDX6_HUMAN | LSILYPATTGR | Light | 596,3 | 2 | 314,21 | 1 | b3 |
| sp|P30041|PRDX6_HUMAN | LSILYPATTGR | Light | 596,3 | 2 | 427,29 | 1 | b4 |
| sp|P30041|PRDX6_HUMAN | LSILYPATTGR | Heavy | 601,3 | 2 | 775,40 | 1 | y7 |
| sp|P30041|PRDX6_HUMAN | LSILYPATTGR | Heavy | 601,3 | 2 | 612,33 | 1 | y6 |
| sp|P30041|PRDX6_HUMAN | LSILYPATTGR | Heavy | 601,3 | 2 | 314,21 | 1 | b3 |
| sp|P30041|PRDX6_HUMAN | LSILYPATTGR | Heavy | 601,3 | 2 | 427,29 | 1 | b4 |
| sp|O00299|CLIC1_HUMAN | LAALNPESNTAGLDIFAK | Light | 923,0 | 2 | 1476,73 | 1 | y14 |
| sp|O00299|CLIC1_HUMAN | LAALNPESNTAGLDIFAK | Light | 923,0 | 2 | 1362,69 | 1 | y13 |
| sp|O00299|CLIC1_HUMAN | LAALNPESNTAGLDIFAK | Light | 923,0 | 2 | 681,85 | 2 | y13 |
| sp|O00299|CLIC1_HUMAN | LAALNPESNTAGLDIFAK | Light | 923,0 | 2 | 256,17 | 1 | b3 |
| sp|O00299|CLIC1_HUMAN | LAALNPESNTAGLDIFAK | Light | 923,0 | 2 | 483,29 | 1 | b5 |
| sp|O00299|CLIC1_HUMAN | LAALNPESNTAGLDIFAK | Heavy | 927,0 | 2 | 1484,75 | 1 | y14 |
| sp|O00299|CLIC1_HUMAN | LAALNPESNTAGLDIFAK | Heavy | 927,0 | 2 | 1370,70 | 1 | y13 |
| sp|O00299|CLIC1_HUMAN | LAALNPESNTAGLDIFAK | Heavy | 927,0 | 2 | 685,86 | 2 | y13 |
| sp|O00299|CLIC1_HUMAN | LAALNPESNTAGLDIFAK | Heavy | 927,0 | 2 | 256,17 | 1 | b3 |
| sp|O00299|CLIC1_HUMAN | LAALNPESNTAGLDIFAK | Heavy | 927,0 | 2 | 483,29 | 1 | b5 |
| sp|O00299|CLIC1_HUMAN | NSNPALNDNLEK | Light | 664,8 | 2 | 1127,57 | 1 | y10 |
| sp|O00299|CLIC1_HUMAN | NSNPALNDNLEK | Light | 664,8 | 2 | 1013,53 | 1 | y9 |
| sp|O00299|CLIC1_HUMAN | NSNPALNDNLEK | Light | 664,8 | 2 | 732,35 | 1 | y6 |
| sp|O00299|CLIC1_HUMAN | NSNPALNDNLEK | Light | 664,8 | 2 | 564,29 | 2 | y10 |
| sp|O00299|CLIC1_HUMAN | NSNPALNDNLEK | Light | 664,8 | 2 | 316,13 | 1 | b3 |
| sp|O00299|CLIC1_HUMAN | NSNPALNDNLEK | Heavy | 668,8 | 2 | 1135,58 | 1 | y10 |
| sp|O00299|CLIC1_HUMAN | NSNPALNDNLEK | Heavy | 668,8 | 2 | 1021,54 | 1 | y9 |
| sp|O00299|CLIC1_HUMAN | NSNPALNDNLEK | Heavy | 668,8 | 2 | 740,37 | 1 | y6 |
| sp|O00299|CLIC1_HUMAN | NSNPALNDNLEK | Heavy | 668,8 | 2 | 568,30 | 2 | y10 |
| sp|O00299|CLIC1_HUMAN | NSNPALNDNLEK | Heavy | 668,8 | 2 | 316,13 | 1 | b3 |
| sp|P14618|KPYM_HUMAN | NTGIICTIGPASR | Light | 680,4 | 2 | 974,51 | 1 | y9 |
| sp|P14618|KPYM_HUMAN | NTGIICTIGPASR | Light | 680,4 | 2 | 861,42 | 1 | y8 |
| sp|P14618|KPYM_HUMAN | NTGIICTIGPASR | Light | 680,4 | 2 | 487,26 | 1 | y5 |
| sp|P14618|KPYM_HUMAN | NTGIICTIGPASR | Light | 680,4 | 2 | 273,12 | 1 | b3 |
| sp|P14618|KPYM_HUMAN | NTGIICTIGPASR | Light | 680,4 | 2 | 386,20 | 1 | b4 |
| sp|P14618|KPYM_HUMAN | NTGIICTIGPASR | Heavy | 685,4 | 2 | 984,52 | 1 | y9 |
| sp|P14618|KPYM_HUMAN | NTGIICTIGPASR | Heavy | 685,4 | 2 | 871,43 | 1 | y8 |
| sp|P14618|KPYM_HUMAN | NTGIICTIGPASR | Heavy | 685,4 | 2 | 497,27 | 1 | y5 |
| sp|P14618|KPYM_HUMAN | NTGIICTIGPASR | Heavy | 685,4 | 2 | 273,12 | 1 | b3 |
| sp|P14618|KPYM_HUMAN | NTGIICTIGPASR | Heavy | 685,4 | 2 | 386,20 | 1 | b4 |
| sp|P14618|KPYM_HUMAN | APIIAVTR | Light | 420,8 | 2 | 559,36 | 1 | y5 |
| sp|P14618|KPYM_HUMAN | APIIAVTR | Light | 420,8 | 2 | 446,27 | 1 | y4 |
| sp|P14618|KPYM_HUMAN | APIIAVTR | Light | 420,8 | 2 | 375,24 | 1 | y3 |
| sp|P14618|KPYM_HUMAN | APIIAVTR | Light | 420,8 | 2 | 385,25 | 2 | y7 |
| sp|P14618|KPYM_HUMAN | APIIAVTR | Light | 420,8 | 2 | 282,18 | 1 | b3 |
| sp|P14618|KPYM_HUMAN | APIIAVTR | Heavy | 425,8 | 2 | 569,36 | 1 | y5 |
| sp|P14618|KPYM_HUMAN | APIIAVTR | Heavy | 425,8 | 2 | 456,28 | 1 | y4 |
| sp|P14618|KPYM_HUMAN | APIIAVTR | Heavy | 425,8 | 2 | 385,24 | 1 | y3 |
| sp|P14618|KPYM_HUMAN | APIIAVTR | Heavy | 425,8 | 2 | 390,25 | 2 | y7 |
| sp|P14618|KPYM_HUMAN | APIIAVTR | Heavy | 425,8 | 2 | 282,18 | 1 | b3 |
| sp|P07355|ANXA2_HUMAN | GVDEVTIVNILTNR | Light | 771,9 | 2 | 1142,69 | 1 | y10 |
| sp|P07355|ANXA2_HUMAN | GVDEVTIVNILTNR | Light | 771,9 | 2 | 1043,62 | 1 | y9 |
| sp|P07355|ANXA2_HUMAN | GVDEVTIVNILTNR | Light | 771,9 | 2 | 942,57 | 1 | y8 |
| sp|P07355|ANXA2_HUMAN | GVDEVTIVNILTNR | Light | 771,9 | 2 | 829,49 | 1 | y7 |
| sp|P07355|ANXA2_HUMAN | GVDEVTIVNILTNR | Heavy | 776,9 | 2 | 1152,70 | 1 | y10 |
| sp|P07355|ANXA2_HUMAN | GVDEVTIVNILTNR | Heavy | 776,9 | 2 | 1053,63 | 1 | y9 |
| sp|P07355|ANXA2_HUMAN | GVDEVTIVNILTNR | Heavy | 776,9 | 2 | 952,58 | 1 | y8 |
| sp|P07355|ANXA2_HUMAN | GVDEVTIVNILTNR | Heavy | 776,9 | 2 | 839,50 | 1 | y7 |
| sp|P07355|ANXA2_HUMAN | QDIAFAYQR | Light | 556,3 | 2 | 868,47 | 1 | y7 |
| sp|P07355|ANXA2_HUMAN | QDIAFAYQR | Light | 556,3 | 2 | 755,38 | 1 | y6 |
| sp|P07355|ANXA2_HUMAN | QDIAFAYQR | Light | 556,3 | 2 | 537,28 | 1 | y4 |
| sp|P07355|ANXA2_HUMAN | QDIAFAYQR | Light | 556,3 | 2 | 466,24 | 1 | y3 |
| sp|P07355|ANXA2_HUMAN | QDIAFAYQR | Light | 556,3 | 2 | 357,18 | 1 | b3 |
| sp|P07355|ANXA2_HUMAN | QDIAFAYQR | Heavy | 561,3 | 2 | 878,48 | 1 | y7 |
| sp|P07355|ANXA2_HUMAN | QDIAFAYQR | Heavy | 561,3 | 2 | 765,39 | 1 | y6 |
| sp|P07355|ANXA2_HUMAN | QDIAFAYQR | Heavy | 561,3 | 2 | 547,29 | 1 | y4 |
| sp|P07355|ANXA2_HUMAN | QDIAFAYQR | Heavy | 561,3 | 2 | 476,25 | 1 | y3 |
| sp|P07355|ANXA2_HUMAN | QDIAFAYQR | Heavy | 561,3 | 2 | 357,18 | 1 | b3 |
| sp|P15941|MUC1_HUMAN | QGGFLGLSNIK | Light | 567,3 | 2 | 1005,57 | 1 | y10 |
| sp|P15941|MUC1_HUMAN | QGGFLGLSNIK | Light | 567,3 | 2 | 948,55 | 1 | y9 |
| sp|P15941|MUC1_HUMAN | QGGFLGLSNIK | Light | 567,3 | 2 | 744,46 | 1 | y7 |
| sp|P15941|MUC1_HUMAN | QGGFLGLSNIK | Light | 567,3 | 2 | 631,38 | 1 | y6 |
| sp|P15941|MUC1_HUMAN | QGGFLGLSNIK | Light | 567,3 | 2 | 461,27 | 1 | y4 |
| sp|P15941|MUC1_HUMAN | QGGFLGLSNIK | Heavy | 571,3 | 2 | 1013,59 | 1 | y10 |
| sp|P15941|MUC1_HUMAN | QGGFLGLSNIK | Heavy | 571,3 | 2 | 956,57 | 1 | y9 |
| sp|P15941|MUC1_HUMAN | QGGFLGLSNIK | Heavy | 571,3 | 2 | 752,48 | 1 | y7 |
| sp|P15941|MUC1_HUMAN | QGGFLGLSNIK | Heavy | 571,3 | 2 | 639,39 | 1 | y6 |
| sp|P15941|MUC1_HUMAN | QGGFLGLSNIK | Heavy | 571,3 | 2 | 469,29 | 1 | y4 |
| sp|P15941|MUC1_HUMAN | EGTINVHDVETQFNQYK | Light | 674,7 | 3 | 827,40 | 1 | y6 |
| sp|P15941|MUC1_HUMAN | EGTINVHDVETQFNQYK | Light | 674,7 | 3 | 699,35 | 1 | y5 |
| sp|P15941|MUC1_HUMAN | EGTINVHDVETQFNQYK | Light | 674,7 | 3 | 704,83 | 2 | y11 |
| sp|P15941|MUC1_HUMAN | EGTINVHDVETQFNQYK | Light | 674,7 | 3 | 464,73 | 2 | y7 |
| sp|P15941|MUC1_HUMAN | EGTINVHDVETQFNQYK | Heavy | 677,3 | 3 | 835,42 | 1 | y6 |
| sp|P15941|MUC1_HUMAN | EGTINVHDVETQFNQYK | Heavy | 677,3 | 3 | 707,36 | 1 | y5 |
| sp|P15941|MUC1_HUMAN | EGTINVHDVETQFNQYK | Heavy | 677,3 | 3 | 708,84 | 2 | y11 |
| sp|P15941|MUC1_HUMAN | EGTINVHDVETQFNQYK | Heavy | 677,3 | 3 | 468,74 | 2 | y7 |
| sp|P55060|XPO2_HUMAN | ANIVHLMLSSPEQIQK | Light | 603,3 | 3 | 916,47 | 1 | y8 |
| sp|P55060|XPO2_HUMAN | ANIVHLMLSSPEQIQK | Light | 603,3 | 3 | 742,41 | 1 | y6 |
| sp|P55060|XPO2_HUMAN | ANIVHLMLSSPEQIQK | Light | 603,3 | 3 | 415,22 | 2 | y7 |
| sp|P55060|XPO2_HUMAN | ANIVHLMLSSPEQIQK | Light | 603,3 | 3 | 371,71 | 2 | y6 |
| sp|P55060|XPO2_HUMAN | ANIVHLMLSSPEQIQK | Heavy | 606,0 | 3 | 924,49 | 1 | y8 |
| sp|P55060|XPO2_HUMAN | ANIVHLMLSSPEQIQK | Heavy | 606,0 | 3 | 750,42 | 1 | y6 |
| sp|P55060|XPO2_HUMAN | ANIVHLMLSSPEQIQK | Heavy | 606,0 | 3 | 419,23 | 2 | y7 |
| sp|P55060|XPO2_HUMAN | ANIVHLMLSSPEQIQK | Heavy | 606,0 | 3 | 375,72 | 2 | y6 |
| sp|P55060|XPO2_HUMAN | LLQTDDEEEAGLLELLK | Light | 965,0 | 2 | 1574,78 | 1 | y14 |
| sp|P55060|XPO2_HUMAN | LLQTDDEEEAGLLELLK | Light | 965,0 | 2 | 785,51 | 1 | y7 |
| sp|P55060|XPO2_HUMAN | LLQTDDEEEAGLLELLK | Light | 965,0 | 2 | 502,32 | 1 | y4 |
| sp|P55060|XPO2_HUMAN | LLQTDDEEEAGLLELLK | Light | 965,0 | 2 | 851,92 | 2 | y15 |
| sp|P55060|XPO2_HUMAN | LLQTDDEEEAGLLELLK | Light | 965,0 | 2 | 355,23 | 1 | b3 |
| sp|P55060|XPO2_HUMAN | LLQTDDEEEAGLLELLK | Heavy | 969,0 | 2 | 1582,79 | 1 | y14 |
| sp|P55060|XPO2_HUMAN | LLQTDDEEEAGLLELLK | Heavy | 969,0 | 2 | 793,53 | 1 | y7 |
| sp|P55060|XPO2_HUMAN | LLQTDDEEEAGLLELLK | Heavy | 969,0 | 2 | 510,34 | 1 | y4 |
| sp|P55060|XPO2_HUMAN | LLQTDDEEEAGLLELLK | Heavy | 969,0 | 2 | 855,93 | 2 | y15 |
| sp|P55060|XPO2_HUMAN | LLQTDDEEEAGLLELLK | Heavy | 969,0 | 2 | 355,23 | 1 | b3 |
| sp|P50454|SERPH_HUMAN | GVVEVTHDLQK | Light | 408,9 | 3 | 840,46 | 1 | y7 |
| sp|P50454|SERPH_HUMAN | GVVEVTHDLQK | Light | 408,9 | 3 | 741,39 | 1 | y6 |
| sp|P50454|SERPH_HUMAN | GVVEVTHDLQK | Light | 408,9 | 3 | 534,79 | 2 | y9 |
| sp|P50454|SERPH_HUMAN | GVVEVTHDLQK | Light | 408,9 | 3 | 485,25 | 2 | y8 |
| sp|P50454|SERPH_HUMAN | GVVEVTHDLQK | Light | 408,9 | 3 | 420,73 | 2 | y7 |
| sp|P50454|SERPH_HUMAN | GVVEVTHDLQK | Heavy | 411,6 | 3 | 848,47 | 1 | y7 |
| sp|P50454|SERPH_HUMAN | GVVEVTHDLQK | Heavy | 411,6 | 3 | 749,40 | 1 | y6 |
| sp|P50454|SERPH_HUMAN | GVVEVTHDLQK | Heavy | 411,6 | 3 | 538,79 | 2 | y9 |
| sp|P50454|SERPH_HUMAN | GVVEVTHDLQK | Heavy | 411,6 | 3 | 489,26 | 2 | y8 |
| sp|P50454|SERPH_HUMAN | GVVEVTHDLQK | Heavy | 411,6 | 3 | 424,74 | 2 | y7 |
| sp|P50454|SERPH_HUMAN | DTQSGSLLFIGR | Light | 647,3 | 2 | 949,55 | 1 | y9 |
| sp|P50454|SERPH_HUMAN | DTQSGSLLFIGR | Light | 647,3 | 2 | 862,51 | 1 | y8 |
| sp|P50454|SERPH_HUMAN | DTQSGSLLFIGR | Light | 647,3 | 2 | 605,38 | 1 | y5 |
| sp|P50454|SERPH_HUMAN | DTQSGSLLFIGR | Light | 647,3 | 2 | 492,29 | 1 | y4 |
| sp|P50454|SERPH_HUMAN | DTQSGSLLFIGR | Light | 647,3 | 2 | 345,22 | 1 | y3 |
| sp|P50454|SERPH_HUMAN | DTQSGSLLFIGR | Heavy | 652,3 | 2 | 959,55 | 1 | y9 |
| sp|P50454|SERPH_HUMAN | DTQSGSLLFIGR | Heavy | 652,3 | 2 | 872,52 | 1 | y8 |
| sp|P50454|SERPH_HUMAN | DTQSGSLLFIGR | Heavy | 652,3 | 2 | 615,39 | 1 | y5 |
| sp|P50454|SERPH_HUMAN | DTQSGSLLFIGR | Heavy | 652,3 | 2 | 502,30 | 1 | y4 |
| sp|P50454|SERPH_HUMAN | DTQSGSLLFIGR | Heavy | 652,3 | 2 | 355,23 | 1 | y3 |
| sp|P16035|TIMP2_HUMAN | EVDSGNDIYGNPIK | Light | 760,9 | 2 | 1292,61 | 1 | y12 |
| sp|P16035|TIMP2_HUMAN | EVDSGNDIYGNPIK | Light | 760,9 | 2 | 1090,55 | 1 | y10 |
| sp|P16035|TIMP2_HUMAN | EVDSGNDIYGNPIK | Light | 760,9 | 2 | 691,38 | 1 | y6 |
| sp|P16035|TIMP2_HUMAN | EVDSGNDIYGNPIK | Light | 760,9 | 2 | 528,31 | 1 | y5 |
| sp|P16035|TIMP2_HUMAN | EVDSGNDIYGNPIK | Light | 760,9 | 2 | 357,25 | 1 | y3 |
| sp|P16035|TIMP2_HUMAN | EVDSGNDIYGNPIK | Heavy | 764,9 | 2 | 1300,63 | 1 | y12 |
| sp|P16035|TIMP2_HUMAN | EVDSGNDIYGNPIK | Heavy | 764,9 | 2 | 1098,57 | 1 | y10 |
| sp|P16035|TIMP2_HUMAN | EVDSGNDIYGNPIK | Heavy | 764,9 | 2 | 699,39 | 1 | y6 |
| sp|P16035|TIMP2_HUMAN | EVDSGNDIYGNPIK | Heavy | 764,9 | 2 | 536,33 | 1 | y5 |
| sp|P16035|TIMP2_HUMAN | EVDSGNDIYGNPIK | Heavy | 764,9 | 2 | 365,26 | 1 | y3 |
| sp|P09382|LEG1_HUMAN | SFVLNLGK | Light | 439,3 | 2 | 643,41 | 1 | y6 |
| sp|P09382|LEG1_HUMAN | SFVLNLGK | Light | 439,3 | 2 | 544,35 | 1 | y5 |
| sp|P09382|LEG1_HUMAN | SFVLNLGK | Light | 439,3 | 2 | 431,26 | 1 | y4 |
| sp|P09382|LEG1_HUMAN | SFVLNLGK | Light | 439,3 | 2 | 322,21 | 2 | y6 |
| sp|P09382|LEG1_HUMAN | SFVLNLGK | Light | 439,3 | 2 | 334,18 | 1 | b3 |
| sp|P09382|LEG1_HUMAN | SFVLNLGK | Heavy | 443,3 | 2 | 651,43 | 1 | y6 |
| sp|P09382|LEG1_HUMAN | SFVLNLGK | Heavy | 443,3 | 2 | 552,36 | 1 | y5 |
| sp|P09382|LEG1_HUMAN | SFVLNLGK | Heavy | 443,3 | 2 | 439,28 | 1 | y4 |
| sp|P09382|LEG1_HUMAN | SFVLNLGK | Heavy | 443,3 | 2 | 326,22 | 2 | y6 |
| sp|P09382|LEG1_HUMAN | SFVLNLGK | Heavy | 443,3 | 2 | 334,18 | 1 | b3 |
| sp|P09382|LEG1_HUMAN | LPDGYEFK | Light | 484,7 | 2 | 758,34 | 1 | y6 |
| sp|P09382|LEG1_HUMAN | LPDGYEFK | Light | 484,7 | 2 | 643,31 | 1 | y5 |
| sp|P09382|LEG1_HUMAN | LPDGYEFK | Light | 484,7 | 2 | 428,20 | 2 | y7 |
| sp|P09382|LEG1_HUMAN | LPDGYEFK | Light | 484,7 | 2 | 675,30 | 1 | b6 |
| sp|P09382|LEG1_HUMAN | LPDGYEFK | Heavy | 488,7 | 2 | 766,35 | 1 | y6 |
| sp|P09382|LEG1_HUMAN | LPDGYEFK | Heavy | 488,7 | 2 | 651,32 | 1 | y5 |
| sp|P09382|LEG1_HUMAN | LPDGYEFK | Heavy | 488,7 | 2 | 432,20 | 2 | y7 |
| sp|P09382|LEG1_HUMAN | LPDGYEFK | Heavy | 488,7 | 2 | 675,30 | 1 | b6 |
| sp|Q13938|CAYP1_HUMAN | EAVIAAAFAK | Light | 495,8 | 2 | 790,48 | 1 | y8 |
| sp|Q13938|CAYP1_HUMAN | EAVIAAAFAK | Light | 495,8 | 2 | 691,41 | 1 | y7 |
| sp|Q13938|CAYP1_HUMAN | EAVIAAAFAK | Light | 495,8 | 2 | 578,33 | 1 | y6 |
| sp|Q13938|CAYP1_HUMAN | EAVIAAAFAK | Light | 495,8 | 2 | 507,29 | 1 | y5 |
| sp|Q13938|CAYP1_HUMAN | EAVIAAAFAK | Light | 495,8 | 2 | 300,16 | 1 | b3 |
| sp|Q13938|CAYP1_HUMAN | EAVIAAAFAK | Heavy | 499,8 | 2 | 798,50 | 1 | y8 |
| sp|Q13938|CAYP1_HUMAN | EAVIAAAFAK | Heavy | 499,8 | 2 | 699,43 | 1 | y7 |
| sp|Q13938|CAYP1_HUMAN | EAVIAAAFAK | Heavy | 499,8 | 2 | 586,34 | 1 | y6 |
| sp|Q13938|CAYP1_HUMAN | EAVIAAAFAK | Heavy | 499,8 | 2 | 515,31 | 1 | y5 |
| sp|Q13938|CAYP1_HUMAN | EAVIAAAFAK | Heavy | 499,8 | 2 | 300,16 | 1 | b3 |
| sp|Q13938|CAYP1_HUMAN | SGDGVVTVDDLR | Light | 616,8 | 2 | 817,44 | 1 | y7 |
| sp|Q13938|CAYP1_HUMAN | SGDGVVTVDDLR | Light | 616,8 | 2 | 518,26 | 1 | y4 |
| sp|Q13938|CAYP1_HUMAN | SGDGVVTVDDLR | Light | 616,8 | 2 | 317,11 | 1 | b4 |
| sp|Q13938|CAYP1_HUMAN | SGDGVVTVDDLR | Light | 616,8 | 2 | 416,18 | 1 | b5 |
| sp|Q13938|CAYP1_HUMAN | SGDGVVTVDDLR | Light | 616,8 | 2 | 515,25 | 1 | b6 |
| sp|Q13938|CAYP1_HUMAN | SGDGVVTVDDLR | Heavy | 621,8 | 2 | 827,45 | 1 | y7 |
| sp|Q13938|CAYP1_HUMAN | SGDGVVTVDDLR | Heavy | 621,8 | 2 | 528,27 | 1 | y4 |
| sp|Q13938|CAYP1_HUMAN | SGDGVVTVDDLR | Heavy | 621,8 | 2 | 317,11 | 1 | b4 |
| sp|Q13938|CAYP1_HUMAN | SGDGVVTVDDLR | Heavy | 621,8 | 2 | 416,18 | 1 | b5 |
| sp|Q13938|CAYP1_HUMAN | SGDGVVTVDDLR | Heavy | 621,8 | 2 | 515,25 | 1 | b6 |
| sp|P42574|CASP3_HUMAN | SGTDVDAANLR | Light | 559,8 | 2 | 873,44 | 1 | y8 |
| sp|P42574|CASP3_HUMAN | SGTDVDAANLR | Light | 559,8 | 2 | 758,42 | 1 | y7 |
| sp|P42574|CASP3_HUMAN | SGTDVDAANLR | Light | 559,8 | 2 | 659,35 | 1 | y6 |
| sp|P42574|CASP3_HUMAN | SGTDVDAANLR | Light | 559,8 | 2 | 544,32 | 1 | y5 |
| sp|P42574|CASP3_HUMAN | SGTDVDAANLR | Light | 559,8 | 2 | 361,14 | 1 | b4 |
| sp|P42574|CASP3_HUMAN | SGTDVDAANLR | Heavy | 564,8 | 2 | 883,45 | 1 | y8 |
| sp|P42574|CASP3_HUMAN | SGTDVDAANLR | Heavy | 564,8 | 2 | 768,42 | 1 | y7 |
| sp|P42574|CASP3_HUMAN | SGTDVDAANLR | Heavy | 564,8 | 2 | 669,36 | 1 | y6 |
| sp|P42574|CASP3_HUMAN | SGTDVDAANLR | Heavy | 564,8 | 2 | 554,33 | 1 | y5 |
| sp|P42574|CASP3_HUMAN | SGTDVDAANLR | Heavy | 564,8 | 2 | 361,14 | 1 | b4 |
| sp|P16949|STMN1_HUMAN | SHEAEVLK | Light | 456,7 | 2 | 688,39 | 1 | y6 |
| sp|P16949|STMN1_HUMAN | SHEAEVLK | Light | 456,7 | 2 | 559,34 | 1 | y5 |
| sp|P16949|STMN1_HUMAN | SHEAEVLK | Light | 456,7 | 2 | 413,23 | 2 | y7 |
| sp|P16949|STMN1_HUMAN | SHEAEVLK | Light | 456,7 | 2 | 653,29 | 1 | b6 |
| sp|P16949|STMN1_HUMAN | SHEAEVLK | Light | 456,7 | 2 | 766,37 | 1 | b7 |
| sp|P16949|STMN1_HUMAN | SHEAEVLK | Heavy | 460,7 | 2 | 696,40 | 1 | y6 |
| sp|P16949|STMN1_HUMAN | SHEAEVLK | Heavy | 460,7 | 2 | 567,36 | 1 | y5 |
| sp|P16949|STMN1_HUMAN | SHEAEVLK | Heavy | 460,7 | 2 | 417,23 | 2 | y7 |
| sp|P16949|STMN1_HUMAN | SHEAEVLK | Heavy | 460,7 | 2 | 653,29 | 1 | b6 |
| sp|P16949|STMN1_HUMAN | SHEAEVLK | Heavy | 460,7 | 2 | 766,37 | 1 | b7 |
| sp|P16949|STMN1_HUMAN | AIEENNNFSK | Light | 583,3 | 2 | 981,43 | 1 | y8 |
| sp|P16949|STMN1_HUMAN | AIEENNNFSK | Light | 583,3 | 2 | 852,38 | 1 | y7 |
| sp|P16949|STMN1_HUMAN | AIEENNNFSK | Light | 583,3 | 2 | 723,34 | 1 | y6 |
| sp|P16949|STMN1_HUMAN | AIEENNNFSK | Light | 583,3 | 2 | 491,22 | 2 | y8 |
| sp|P16949|STMN1_HUMAN | AIEENNNFSK | Light | 583,3 | 2 | 314,17 | 1 | b3 |
| sp|P16949|STMN1_HUMAN | AIEENNNFSK | Heavy | 587,3 | 2 | 989,44 | 1 | y8 |
| sp|P16949|STMN1_HUMAN | AIEENNNFSK | Heavy | 587,3 | 2 | 860,40 | 1 | y7 |
| sp|P16949|STMN1_HUMAN | AIEENNNFSK | Heavy | 587,3 | 2 | 731,36 | 1 | y6 |
| sp|P16949|STMN1_HUMAN | AIEENNNFSK | Heavy | 587,3 | 2 | 495,22 | 2 | y8 |
| sp|P16949|STMN1_HUMAN | AIEENNNFSK | Heavy | 587,3 | 2 | 314,17 | 1 | b3 |
| sp|P14780|MMP9_HUMAN | SLGPALLLLQK | Light | 576,9 | 2 | 952,62 | 1 | y9 |
| sp|P14780|MMP9_HUMAN | SLGPALLLLQK | Light | 576,9 | 2 | 895,60 | 1 | y8 |
| sp|P14780|MMP9_HUMAN | SLGPALLLLQK | Light | 576,9 | 2 | 614,42 | 1 | y5 |
| sp|P14780|MMP9_HUMAN | SLGPALLLLQK | Light | 576,9 | 2 | 476,81 | 2 | y9 |
| sp|P14780|MMP9_HUMAN | SLGPALLLLQK | Light | 576,9 | 2 | 448,30 | 2 | y8 |
| sp|P14780|MMP9_HUMAN | SLGPALLLLQK | Heavy | 580,9 | 2 | 960,63 | 1 | y9 |
| sp|P14780|MMP9_HUMAN | SLGPALLLLQK | Heavy | 580,9 | 2 | 903,61 | 1 | y8 |
| sp|P14780|MMP9_HUMAN | SLGPALLLLQK | Heavy | 580,9 | 2 | 622,44 | 1 | y5 |
| sp|P14780|MMP9_HUMAN | SLGPALLLLQK | Heavy | 580,9 | 2 | 480,82 | 2 | y9 |
| sp|P14780|MMP9_HUMAN | SLGPALLLLQK | Heavy | 580,9 | 2 | 452,31 | 2 | y8 |
| sp|P14780|MMP9_HUMAN | AFALWSAVTPLTFTR | Light | 841,0 | 2 | 1278,68 | 1 | y11 |
| sp|P14780|MMP9_HUMAN | AFALWSAVTPLTFTR | Light | 841,0 | 2 | 1092,60 | 1 | y10 |
| sp|P14780|MMP9_HUMAN | AFALWSAVTPLTFTR | Light | 841,0 | 2 | 934,54 | 1 | y8 |
| sp|P14780|MMP9_HUMAN | AFALWSAVTPLTFTR | Light | 841,0 | 2 | 734,42 | 1 | y6 |
| sp|P14780|MMP9_HUMAN | AFALWSAVTPLTFTR | Light | 841,0 | 2 | 290,15 | 1 | b3 |
| sp|P14780|MMP9_HUMAN | AFALWSAVTPLTFTR | Heavy | 846,0 | 2 | 1288,69 | 1 | y11 |
| sp|P14780|MMP9_HUMAN | AFALWSAVTPLTFTR | Heavy | 846,0 | 2 | 1102,61 | 1 | y10 |
| sp|P14780|MMP9_HUMAN | AFALWSAVTPLTFTR | Heavy | 846,0 | 2 | 944,54 | 1 | y8 |
| sp|P14780|MMP9_HUMAN | AFALWSAVTPLTFTR | Heavy | 846,0 | 2 | 744,43 | 1 | y6 |
| sp|P14780|MMP9_HUMAN | AFALWSAVTPLTFTR | Heavy | 846,0 | 2 | 290,15 | 1 | b3 |
| sp|P08670|VIME_HUMAN | ILLAELEQLK | Light | 585,4 | 2 | 943,55 | 1 | y8 |
| sp|P08670|VIME_HUMAN | ILLAELEQLK | Light | 585,4 | 2 | 830,46 | 1 | y7 |
| sp|P08670|VIME_HUMAN | ILLAELEQLK | Light | 585,4 | 2 | 759,42 | 1 | y6 |
| sp|P08670|VIME_HUMAN | ILLAELEQLK | Light | 585,4 | 2 | 472,28 | 2 | y8 |
| sp|P08670|VIME_HUMAN | ILLAELEQLK | Light | 585,4 | 2 | 340,26 | 1 | b3 |
| sp|P08670|VIME_HUMAN | ILLAELEQLK | Heavy | 589,4 | 2 | 951,56 | 1 | y8 |
| sp|P08670|VIME_HUMAN | ILLAELEQLK | Heavy | 589,4 | 2 | 838,48 | 1 | y7 |
| sp|P08670|VIME_HUMAN | ILLAELEQLK | Heavy | 589,4 | 2 | 767,44 | 1 | y6 |
| sp|P08670|VIME_HUMAN | ILLAELEQLK | Heavy | 589,4 | 2 | 476,28 | 2 | y8 |
| sp|P08670|VIME_HUMAN | ILLAELEQLK | Heavy | 589,4 | 2 | 340,26 | 1 | b3 |
| sp|P08670|VIME_HUMAN | ISLPLPNFSSLNLR | Light | 786,0 | 2 | 1257,69 | 1 | y11 |
| sp|P08670|VIME_HUMAN | ISLPLPNFSSLNLR | Light | 786,0 | 2 | 1047,56 | 1 | y9 |
| sp|P08670|VIME_HUMAN | ISLPLPNFSSLNLR | Light | 786,0 | 2 | 685,89 | 2 | y12 |
| sp|P08670|VIME_HUMAN | ISLPLPNFSSLNLR | Light | 786,0 | 2 | 629,35 | 2 | y11 |
| sp|P08670|VIME_HUMAN | ISLPLPNFSSLNLR | Light | 786,0 | 2 | 524,28 | 2 | y9 |
| sp|P08670|VIME_HUMAN | ISLPLPNFSSLNLR | Heavy | 791,0 | 2 | 1267,70 | 1 | y11 |
| sp|P08670|VIME_HUMAN | ISLPLPNFSSLNLR | Heavy | 791,0 | 2 | 1057,57 | 1 | y9 |
| sp|P08670|VIME_HUMAN | ISLPLPNFSSLNLR | Heavy | 791,0 | 2 | 690,90 | 2 | y12 |
| sp|P08670|VIME_HUMAN | ISLPLPNFSSLNLR | Heavy | 791,0 | 2 | 634,36 | 2 | y11 |
| sp|P08670|VIME_HUMAN | ISLPLPNFSSLNLR | Heavy | 791,0 | 2 | 529,29 | 2 | y9 |
| sp|P16070|CD44_HUMAN | TEAADLCK | Light | 454,2 | 2 | 806,37 | 1 | y7 |
| sp|P16070|CD44_HUMAN | TEAADLCK | Light | 454,2 | 2 | 677,33 | 1 | y6 |
| sp|P16070|CD44_HUMAN | TEAADLCK | Light | 454,2 | 2 | 606,29 | 1 | y5 |
| sp|P16070|CD44_HUMAN | TEAADLCK | Light | 454,2 | 2 | 535,25 | 1 | y4 |
| sp|P16070|CD44_HUMAN | TEAADLCK | Light | 454,2 | 2 | 302,13 | 1 | b3 |
| sp|P16070|CD44_HUMAN | TEAADLCK | Heavy | 458,2 | 2 | 814,39 | 1 | y7 |
| sp|P16070|CD44_HUMAN | TEAADLCK | Heavy | 458,2 | 2 | 685,34 | 1 | y6 |
| sp|P16070|CD44_HUMAN | TEAADLCK | Heavy | 458,2 | 2 | 614,31 | 1 | y5 |
| sp|P16070|CD44_HUMAN | TEAADLCK | Heavy | 458,2 | 2 | 543,27 | 1 | y4 |
| sp|P16070|CD44_HUMAN | TEAADLCK | Heavy | 458,2 | 2 | 302,13 | 1 | b3 |
| sp|P15328|FOLR1_HUMAN | TELLNVCMNAK | Light | 646,8 | 2 | 1062,54 | 1 | y9 |
| sp|P15328|FOLR1_HUMAN | TELLNVCMNAK | Light | 646,8 | 2 | 949,46 | 1 | y8 |
| sp|P15328|FOLR1_HUMAN | TELLNVCMNAK | Light | 646,8 | 2 | 836,38 | 1 | y7 |
| sp|P15328|FOLR1_HUMAN | TELLNVCMNAK | Light | 646,8 | 2 | 623,26 | 1 | y5 |
| sp|P15328|FOLR1_HUMAN | TELLNVCMNAK | Light | 646,8 | 2 | 344,18 | 1 | b3 |
| sp|P15328|FOLR1_HUMAN | TELLNVCMNAK | Heavy | 650,8 | 2 | 1070,56 | 1 | y9 |
| sp|P15328|FOLR1_HUMAN | TELLNVCMNAK | Heavy | 650,8 | 2 | 957,47 | 1 | y8 |
| sp|P15328|FOLR1_HUMAN | TELLNVCMNAK | Heavy | 650,8 | 2 | 844,39 | 1 | y7 |
| sp|P15328|FOLR1_HUMAN | TELLNVCMNAK | Heavy | 650,8 | 2 | 631,28 | 1 | y5 |
| sp|P15328|FOLR1_HUMAN | TELLNVCMNAK | Heavy | 650,8 | 2 | 344,18 | 1 | b3 |
| sp|P15328|FOLR1_HUMAN | FNWNHCGEMAPACK | Light | 574,6 | 3 | 546,27 | 1 | y5 |
| sp|P15328|FOLR1_HUMAN | FNWNHCGEMAPACK | Light | 574,6 | 3 | 475,23 | 1 | y4 |
| sp|P15328|FOLR1_HUMAN | FNWNHCGEMAPACK | Light | 574,6 | 3 | 787,82 | 2 | y13 |
| sp|P15328|FOLR1_HUMAN | FNWNHCGEMAPACK | Light | 574,6 | 3 | 730,80 | 2 | y12 |
| sp|P15328|FOLR1_HUMAN | FNWNHCGEMAPACK | Light | 574,6 | 3 | 637,76 | 2 | y11 |
| sp|P15328|FOLR1_HUMAN | FNWNHCGEMAPACK | Heavy | 577,2 | 3 | 554,28 | 1 | y5 |
| sp|P15328|FOLR1_HUMAN | FNWNHCGEMAPACK | Heavy | 577,2 | 3 | 483,25 | 1 | y4 |
| sp|P15328|FOLR1_HUMAN | FNWNHCGEMAPACK | Heavy | 577,2 | 3 | 791,83 | 2 | y13 |
| sp|P15328|FOLR1_HUMAN | FNWNHCGEMAPACK | Heavy | 577,2 | 3 | 734,80 | 2 | y12 |
| sp|P15328|FOLR1_HUMAN | FNWNHCGEMAPACK | Heavy | 577,2 | 3 | 641,76 | 2 | y11 |
| sp|O43852|CALU_HUMAN | TFDQLTPEESK | Light | 647,8 | 2 | 1046,50 | 1 | y9 |
| sp|O43852|CALU_HUMAN | TFDQLTPEESK | Light | 647,8 | 2 | 803,41 | 1 | y7 |
| sp|O43852|CALU_HUMAN | TFDQLTPEESK | Light | 647,8 | 2 | 589,28 | 1 | y5 |
| sp|O43852|CALU_HUMAN | TFDQLTPEESK | Light | 647,8 | 2 | 364,15 | 1 | b3 |
| sp|O43852|CALU_HUMAN | TFDQLTPEESK | Light | 647,8 | 2 | 492,21 | 1 | b4 |
| sp|O43852|CALU_HUMAN | TFDQLTPEESK | Heavy | 651,8 | 2 | 1054,51 | 1 | y9 |
| sp|O43852|CALU_HUMAN | TFDQLTPEESK | Heavy | 651,8 | 2 | 811,43 | 1 | y7 |
| sp|O43852|CALU_HUMAN | TFDQLTPEESK | Heavy | 651,8 | 2 | 597,30 | 1 | y5 |
| sp|O43852|CALU_HUMAN | TFDQLTPEESK | Heavy | 651,8 | 2 | 364,15 | 1 | b3 |
| sp|O43852|CALU_HUMAN | TFDQLTPEESK | Heavy | 651,8 | 2 | 492,21 | 1 | b4 |
| sp|P09603|CSF1_HUMAN | AFLLVQDIMEDTMR | Light | 841,4 | 2 | 1237,56 | 1 | y10 |
| sp|P09603|CSF1_HUMAN | AFLLVQDIMEDTMR | Light | 841,4 | 2 | 1138,49 | 1 | y9 |
| sp|P09603|CSF1_HUMAN | AFLLVQDIMEDTMR | Light | 841,4 | 2 | 1010,43 | 1 | y8 |
| sp|P09603|CSF1_HUMAN | AFLLVQDIMEDTMR | Light | 841,4 | 2 | 332,20 | 1 | b3 |
| sp|P09603|CSF1_HUMAN | AFLLVQDIMEDTMR | Light | 841,4 | 2 | 445,28 | 1 | b4 |
| sp|P09603|CSF1_HUMAN | AFLLVQDIMEDTMR | Heavy | 846,4 | 2 | 1247,56 | 1 | y10 |
| sp|P09603|CSF1_HUMAN | AFLLVQDIMEDTMR | Heavy | 846,4 | 2 | 1148,49 | 1 | y9 |
| sp|P09603|CSF1_HUMAN | AFLLVQDIMEDTMR | Heavy | 846,4 | 2 | 1020,44 | 1 | y8 |
| sp|P09603|CSF1_HUMAN | AFLLVQDIMEDTMR | Heavy | 846,4 | 2 | 332,20 | 1 | b3 |
| sp|P09603|CSF1_HUMAN | AFLLVQDIMEDTMR | Heavy | 846,4 | 2 | 445,28 | 1 | b4 |
| sp|P09603|CSF1_HUMAN | TFYETPLQLLEK | Light | 741,4 | 2 | 1233,67 | 1 | y10 |
| sp|P09603|CSF1_HUMAN | TFYETPLQLLEK | Light | 741,4 | 2 | 617,34 | 2 | y10 |
| sp|P09603|CSF1_HUMAN | TFYETPLQLLEK | Light | 741,4 | 2 | 412,19 | 1 | b3 |
| sp|P09603|CSF1_HUMAN | TFYETPLQLLEK | Light | 741,4 | 2 | 541,23 | 1 | b4 |
| sp|P09603|CSF1_HUMAN | TFYETPLQLLEK | Heavy | 745,4 | 2 | 1241,69 | 1 | y10 |
| sp|P09603|CSF1_HUMAN | TFYETPLQLLEK | Heavy | 745,4 | 2 | 621,35 | 2 | y10 |
| sp|P09603|CSF1_HUMAN | TFYETPLQLLEK | Heavy | 745,4 | 2 | 412,19 | 1 | b3 |
| sp|P09603|CSF1_HUMAN | TFYETPLQLLEK | Heavy | 745,4 | 2 | 541,23 | 1 | b4 |
| sp|Q06830|PRDX1_HUMAN | ADEGISFR | Light | 447,7 | 2 | 823,39 | 1 | y7 |
| sp|Q06830|PRDX1_HUMAN | ADEGISFR | Light | 447,7 | 2 | 708,37 | 1 | y6 |
| sp|Q06830|PRDX1_HUMAN | ADEGISFR | Light | 447,7 | 2 | 579,32 | 1 | y5 |
| sp|Q06830|PRDX1_HUMAN | ADEGISFR | Light | 447,7 | 2 | 522,30 | 1 | y4 |
| sp|Q06830|PRDX1_HUMAN | ADEGISFR | Light | 447,7 | 2 | 409,22 | 1 | y3 |
| sp|Q06830|PRDX1_HUMAN | ADEGISFR | Heavy | 452,7 | 2 | 833,40 | 1 | y7 |
| sp|Q06830|PRDX1_HUMAN | ADEGISFR | Heavy | 452,7 | 2 | 718,38 | 1 | y6 |
| sp|Q06830|PRDX1_HUMAN | ADEGISFR | Heavy | 452,7 | 2 | 589,33 | 1 | y5 |
| sp|Q06830|PRDX1_HUMAN | ADEGISFR | Heavy | 452,7 | 2 | 532,31 | 1 | y4 |
| sp|Q06830|PRDX1_HUMAN | ADEGISFR | Heavy | 452,7 | 2 | 419,23 | 1 | y3 |
| sp|Q06830|PRDX1_HUMAN | LVQAFQFTDK | Light | 598,8 | 2 | 984,48 | 1 | y8 |
| sp|Q06830|PRDX1_HUMAN | LVQAFQFTDK | Light | 598,8 | 2 | 856,42 | 1 | y7 |
| sp|Q06830|PRDX1_HUMAN | LVQAFQFTDK | Light | 598,8 | 2 | 785,38 | 1 | y6 |
| sp|Q06830|PRDX1_HUMAN | LVQAFQFTDK | Light | 598,8 | 2 | 492,74 | 2 | y8 |
| sp|Q06830|PRDX1_HUMAN | LVQAFQFTDK | Light | 598,8 | 2 | 341,22 | 1 | b3 |
| sp|Q06830|PRDX1_HUMAN | LVQAFQFTDK | Heavy | 602,8 | 2 | 992,49 | 1 | y8 |
| sp|Q06830|PRDX1_HUMAN | LVQAFQFTDK | Heavy | 602,8 | 2 | 864,43 | 1 | y7 |
| sp|Q06830|PRDX1_HUMAN | LVQAFQFTDK | Heavy | 602,8 | 2 | 793,40 | 1 | y6 |
| sp|Q06830|PRDX1_HUMAN | LVQAFQFTDK | Heavy | 602,8 | 2 | 496,75 | 2 | y8 |
| sp|Q06830|PRDX1_HUMAN | LVQAFQFTDK | Heavy | 602,8 | 2 | 341,22 | 1 | b3 |
| sp|O75556|SG2A1_HUMAN | TINSDISIPEYK | Light | 690,4 | 2 | 1165,57 | 1 | y10 |
| sp|O75556|SG2A1_HUMAN | TINSDISIPEYK | Light | 690,4 | 2 | 1051,53 | 1 | y9 |
| sp|O75556|SG2A1_HUMAN | TINSDISIPEYK | Light | 690,4 | 2 | 964,50 | 1 | y8 |
| sp|O75556|SG2A1_HUMAN | TINSDISIPEYK | Light | 690,4 | 2 | 736,39 | 1 | y6 |
| sp|O75556|SG2A1_HUMAN | TINSDISIPEYK | Light | 690,4 | 2 | 536,27 | 1 | y4 |
| sp|O75556|SG2A1_HUMAN | TINSDISIPEYK | Heavy | 694,4 | 2 | 1173,59 | 1 | y10 |
| sp|O75556|SG2A1_HUMAN | TINSDISIPEYK | Heavy | 694,4 | 2 | 1059,54 | 1 | y9 |
| sp|O75556|SG2A1_HUMAN | TINSDISIPEYK | Heavy | 694,4 | 2 | 972,51 | 1 | y8 |
| sp|O75556|SG2A1_HUMAN | TINSDISIPEYK | Heavy | 694,4 | 2 | 744,40 | 1 | y6 |
| sp|O75556|SG2A1_HUMAN | TINSDISIPEYK | Heavy | 694,4 | 2 | 544,29 | 1 | y4 |
| sp|O75556|SG2A1_HUMAN | ELLQEFIDSDAAAEAMGK | Light | 646,6 | 3 | 748,37 | 1 | y8 |
| sp|O75556|SG2A1_HUMAN | ELLQEFIDSDAAAEAMGK | Light | 646,6 | 3 | 677,33 | 1 | y7 |
| sp|O75556|SG2A1_HUMAN | ELLQEFIDSDAAAEAMGK | Light | 646,6 | 3 | 606,29 | 1 | y6 |
| sp|O75556|SG2A1_HUMAN | ELLQEFIDSDAAAEAMGK | Light | 646,6 | 3 | 535,25 | 1 | y5 |
| sp|O75556|SG2A1_HUMAN | ELLQEFIDSDAAAEAMGK | Light | 646,6 | 3 | 760,39 | 1 | b6 |
| sp|O75556|SG2A1_HUMAN | ELLQEFIDSDAAAEAMGK | Heavy | 649,3 | 3 | 756,38 | 1 | y8 |
| sp|O75556|SG2A1_HUMAN | ELLQEFIDSDAAAEAMGK | Heavy | 649,3 | 3 | 685,34 | 1 | y7 |
| sp|O75556|SG2A1_HUMAN | ELLQEFIDSDAAAEAMGK | Heavy | 649,3 | 3 | 614,31 | 1 | y6 |
| sp|O75556|SG2A1_HUMAN | ELLQEFIDSDAAAEAMGK | Heavy | 649,3 | 3 | 543,27 | 1 | y5 |
| sp|O75556|SG2A1_HUMAN | ELLQEFIDSDAAAEAMGK | Heavy | 649,3 | 3 | 760,39 | 1 | b6 |
| sp|P22626|ROA2_HUMAN | TLETVPLER | Light | 529,3 | 2 | 843,46 | 1 | y7 |
| sp|P22626|ROA2_HUMAN | TLETVPLER | Light | 529,3 | 2 | 714,41 | 1 | y6 |
| sp|P22626|ROA2_HUMAN | TLETVPLER | Light | 529,3 | 2 | 613,37 | 1 | y5 |
| sp|P22626|ROA2_HUMAN | TLETVPLER | Light | 529,3 | 2 | 514,30 | 1 | y4 |
| sp|P22626|ROA2_HUMAN | TLETVPLER | Light | 529,3 | 2 | 344,18 | 1 | b3 |
| sp|P22626|ROA2_HUMAN | TLETVPLER | Heavy | 534,3 | 2 | 853,47 | 1 | y7 |
| sp|P22626|ROA2_HUMAN | TLETVPLER | Heavy | 534,3 | 2 | 724,42 | 1 | y6 |
| sp|P22626|ROA2_HUMAN | TLETVPLER | Heavy | 534,3 | 2 | 623,38 | 1 | y5 |
| sp|P22626|ROA2_HUMAN | TLETVPLER | Heavy | 534,3 | 2 | 524,31 | 1 | y4 |
| sp|P22626|ROA2_HUMAN | TLETVPLER | Heavy | 534,3 | 2 | 344,18 | 1 | b3 |
| sp|P22626|ROA2_HUMAN | GGNFGFGDSR | Light | 507,2 | 2 | 785,36 | 1 | y7 |
| sp|P22626|ROA2_HUMAN | GGNFGFGDSR | Light | 507,2 | 2 | 638,29 | 1 | y6 |
| sp|P22626|ROA2_HUMAN | GGNFGFGDSR | Light | 507,2 | 2 | 581,27 | 1 | y5 |
| sp|P22626|ROA2_HUMAN | GGNFGFGDSR | Light | 507,2 | 2 | 434,20 | 1 | y4 |
| sp|P22626|ROA2_HUMAN | GGNFGFGDSR | Light | 507,2 | 2 | 376,16 | 1 | b4 |
| sp|P22626|ROA2_HUMAN | GGNFGFGDSR | Heavy | 512,2 | 2 | 795,37 | 1 | y7 |
| sp|P22626|ROA2_HUMAN | GGNFGFGDSR | Heavy | 512,2 | 2 | 648,30 | 1 | y6 |
| sp|P22626|ROA2_HUMAN | GGNFGFGDSR | Heavy | 512,2 | 2 | 591,28 | 1 | y5 |
| sp|P22626|ROA2_HUMAN | GGNFGFGDSR | Heavy | 512,2 | 2 | 444,21 | 1 | y4 |
| sp|P22626|ROA2_HUMAN | GGNFGFGDSR | Heavy | 512,2 | 2 | 376,16 | 1 | b4 |
| sp|P09211|GSTP1_HUMAN | ASCLYGQLPK | Light | 568,8 | 2 | 818,48 | 1 | y7 |
| sp|P09211|GSTP1_HUMAN | ASCLYGQLPK | Light | 568,8 | 2 | 542,33 | 1 | y5 |
| sp|P09211|GSTP1_HUMAN | ASCLYGQLPK | Light | 568,8 | 2 | 489,76 | 2 | y8 |
| sp|P09211|GSTP1_HUMAN | ASCLYGQLPK | Light | 568,8 | 2 | 319,11 | 1 | b3 |
| sp|P09211|GSTP1_HUMAN | ASCLYGQLPK | Heavy | 572,8 | 2 | 826,49 | 1 | y7 |
| sp|P09211|GSTP1_HUMAN | ASCLYGQLPK | Heavy | 572,8 | 2 | 550,34 | 1 | y5 |
| sp|P09211|GSTP1_HUMAN | ASCLYGQLPK | Heavy | 572,8 | 2 | 493,76 | 2 | y8 |
| sp|P09211|GSTP1_HUMAN | ASCLYGQLPK | Heavy | 572,8 | 2 | 319,11 | 1 | b3 |
| sp|P09211|GSTP1_HUMAN | TLGLYGK | Light | 376,2 | 2 | 650,39 | 1 | y6 |
| sp|P09211|GSTP1_HUMAN | TLGLYGK | Light | 376,2 | 2 | 480,28 | 1 | y4 |
| sp|P09211|GSTP1_HUMAN | TLGLYGK | Light | 376,2 | 2 | 367,20 | 1 | y3 |
| sp|P09211|GSTP1_HUMAN | TLGLYGK | Light | 376,2 | 2 | 325,70 | 2 | y6 |
| sp|P09211|GSTP1_HUMAN | TLGLYGK | Light | 376,2 | 2 | 272,16 | 1 | b3 |
| sp|P09211|GSTP1_HUMAN | TLGLYGK | Heavy | 380,2 | 2 | 658,40 | 1 | y6 |
| sp|P09211|GSTP1_HUMAN | TLGLYGK | Heavy | 380,2 | 2 | 488,30 | 1 | y4 |
| sp|P09211|GSTP1_HUMAN | TLGLYGK | Heavy | 380,2 | 2 | 375,21 | 1 | y3 |
| sp|P09211|GSTP1_HUMAN | TLGLYGK | Heavy | 380,2 | 2 | 329,70 | 2 | y6 |
| sp|P09211|GSTP1_HUMAN | TLGLYGK | Heavy | 380,2 | 2 | 272,16 | 1 | b3 |
| sp|Q01995|TAGL_HUMAN | TLMALGSLAVTK | Light | 602,9 | 2 | 990,57 | 1 | y10 |
| sp|Q01995|TAGL_HUMAN | TLMALGSLAVTK | Light | 602,9 | 2 | 859,52 | 1 | y9 |
| sp|Q01995|TAGL_HUMAN | TLMALGSLAVTK | Light | 602,9 | 2 | 788,49 | 1 | y8 |
| sp|Q01995|TAGL_HUMAN | TLMALGSLAVTK | Light | 602,9 | 2 | 675,40 | 1 | y7 |
| sp|Q01995|TAGL_HUMAN | TLMALGSLAVTK | Light | 602,9 | 2 | 417,22 | 1 | b4 |
| sp|Q01995|TAGL_HUMAN | TLMALGSLAVTK | Heavy | 606,9 | 2 | 998,58 | 1 | y10 |
| sp|Q01995|TAGL_HUMAN | TLMALGSLAVTK | Heavy | 606,9 | 2 | 867,54 | 1 | y9 |
| sp|Q01995|TAGL_HUMAN | TLMALGSLAVTK | Heavy | 606,9 | 2 | 796,50 | 1 | y8 |
| sp|Q01995|TAGL_HUMAN | TLMALGSLAVTK | Heavy | 606,9 | 2 | 683,42 | 1 | y7 |
| sp|Q01995|TAGL_HUMAN | TLMALGSLAVTK | Heavy | 606,9 | 2 | 417,22 | 1 | b4 |
| sp|Q01995|TAGL_HUMAN | EFTESQLQEGK | Light | 648,3 | 2 | 1019,50 | 1 | y9 |
| sp|Q01995|TAGL_HUMAN | EFTESQLQEGK | Light | 648,3 | 2 | 789,41 | 1 | y7 |
| sp|Q01995|TAGL_HUMAN | EFTESQLQEGK | Light | 648,3 | 2 | 574,32 | 1 | y5 |
| sp|Q01995|TAGL_HUMAN | EFTESQLQEGK | Light | 648,3 | 2 | 461,24 | 1 | y4 |
| sp|Q01995|TAGL_HUMAN | EFTESQLQEGK | Light | 648,3 | 2 | 333,18 | 1 | y3 |
| sp|Q01995|TAGL_HUMAN | EFTESQLQEGK | Heavy | 652,3 | 2 | 1027,51 | 1 | y9 |
| sp|Q01995|TAGL_HUMAN | EFTESQLQEGK | Heavy | 652,3 | 2 | 797,42 | 1 | y7 |
| sp|Q01995|TAGL_HUMAN | EFTESQLQEGK | Heavy | 652,3 | 2 | 582,33 | 1 | y5 |
| sp|Q01995|TAGL_HUMAN | EFTESQLQEGK | Heavy | 652,3 | 2 | 469,25 | 1 | y4 |
| sp|Q01995|TAGL_HUMAN | EFTESQLQEGK | Heavy | 652,3 | 2 | 341,19 | 1 | y3 |
| sp|P00441|SODC_HUMAN | AVCVLK | Light | 345,2 | 2 | 618,36 | 1 | y5 |
| sp|P00441|SODC_HUMAN | AVCVLK | Light | 345,2 | 2 | 519,30 | 1 | y4 |
| sp|P00441|SODC_HUMAN | AVCVLK | Light | 345,2 | 2 | 359,27 | 1 | y3 |
| sp|P00441|SODC_HUMAN | AVCVLK | Light | 345,2 | 2 | 309,69 | 2 | y5 |
| sp|P00441|SODC_HUMAN | AVCVLK | Light | 345,2 | 2 | 260,15 | 2 | y4 |
| sp|P00441|SODC_HUMAN | AVCVLK | Heavy | 349,2 | 2 | 626,38 | 1 | y5 |
| sp|P00441|SODC_HUMAN | AVCVLK | Heavy | 349,2 | 2 | 527,31 | 1 | y4 |
| sp|P00441|SODC_HUMAN | AVCVLK | Heavy | 349,2 | 2 | 367,28 | 1 | y3 |
| sp|P00441|SODC_HUMAN | AVCVLK | Heavy | 349,2 | 2 | 313,69 | 2 | y5 |
| sp|P00441|SODC_HUMAN | AVCVLK | Heavy | 349,2 | 2 | 264,16 | 2 | y4 |
| sp|P00441|SODC_HUMAN | TLVVHEK | Light | 413,2 | 2 | 611,35 | 1 | y5 |
| sp|P00441|SODC_HUMAN | TLVVHEK | Light | 413,2 | 2 | 512,28 | 1 | y4 |
| sp|P00441|SODC_HUMAN | TLVVHEK | Light | 413,2 | 2 | 362,72 | 2 | y6 |
| sp|P00441|SODC_HUMAN | TLVVHEK | Light | 413,2 | 2 | 306,18 | 2 | y5 |
| sp|P00441|SODC_HUMAN | TLVVHEK | Light | 413,2 | 2 | 314,21 | 1 | b3 |
| sp|P00441|SODC_HUMAN | TLVVHEK | Heavy | 417,3 | 2 | 619,37 | 1 | y5 |
| sp|P00441|SODC_HUMAN | TLVVHEK | Heavy | 417,3 | 2 | 520,30 | 1 | y4 |
| sp|P00441|SODC_HUMAN | TLVVHEK | Heavy | 417,3 | 2 | 366,73 | 2 | y6 |
| sp|P00441|SODC_HUMAN | TLVVHEK | Heavy | 417,3 | 2 | 310,19 | 2 | y5 |
| sp|P00441|SODC_HUMAN | TLVVHEK | Heavy | 417,3 | 2 | 314,21 | 1 | b3 |
| sp|P04083|ANXA1_HUMAN | GGPGSAVSPYPTFNPSSDVAALHK | Light | 786,1 | 3 | 965,49 | 2 | y18 |
| sp|P04083|ANXA1_HUMAN | GGPGSAVSPYPTFNPSSDVAALHK | Light | 786,1 | 3 | 915,95 | 2 | y17 |
| sp|P04083|ANXA1_HUMAN | GGPGSAVSPYPTFNPSSDVAALHK | Light | 786,1 | 3 | 872,44 | 2 | y16 |
| sp|P04083|ANXA1_HUMAN | GGPGSAVSPYPTFNPSSDVAALHK | Light | 786,1 | 3 | 512,77 | 2 | y10 |
| sp|P04083|ANXA1_HUMAN | GGPGSAVSPYPTFNPSSDVAALHK | Light | 786,1 | 3 | 526,26 | 1 | b7 |
| sp|P04083|ANXA1_HUMAN | GGPGSAVSPYPTFNPSSDVAALHK | Heavy | 788,7 | 3 | 969,50 | 2 | y18 |
| sp|P04083|ANXA1_HUMAN | GGPGSAVSPYPTFNPSSDVAALHK | Heavy | 788,7 | 3 | 919,96 | 2 | y17 |
| sp|P04083|ANXA1_HUMAN | GGPGSAVSPYPTFNPSSDVAALHK | Heavy | 788,7 | 3 | 876,45 | 2 | y16 |
| sp|P04083|ANXA1_HUMAN | GGPGSAVSPYPTFNPSSDVAALHK | Heavy | 788,7 | 3 | 516,78 | 2 | y10 |
| sp|P04083|ANXA1_HUMAN | GGPGSAVSPYPTFNPSSDVAALHK | Heavy | 788,7 | 3 | 526,26 | 1 | b7 |
| sp|P04083|ANXA1_HUMAN | DITSDTSGDFR | Light | 607,3 | 2 | 985,42 | 1 | y9 |
| sp|P04083|ANXA1_HUMAN | DITSDTSGDFR | Light | 607,3 | 2 | 884,37 | 1 | y8 |
| sp|P04083|ANXA1_HUMAN | DITSDTSGDFR | Light | 607,3 | 2 | 797,34 | 1 | y7 |
| sp|P04083|ANXA1_HUMAN | DITSDTSGDFR | Light | 607,3 | 2 | 493,21 | 2 | y9 |
| sp|P04083|ANXA1_HUMAN | DITSDTSGDFR | Light | 607,3 | 2 | 330,17 | 1 | b3 |
| sp|P04083|ANXA1_HUMAN | DITSDTSGDFR | Heavy | 612,3 | 2 | 995,43 | 1 | y9 |
| sp|P04083|ANXA1_HUMAN | DITSDTSGDFR | Heavy | 612,3 | 2 | 894,38 | 1 | y8 |
| sp|P04083|ANXA1_HUMAN | DITSDTSGDFR | Heavy | 612,3 | 2 | 807,35 | 1 | y7 |
| sp|P04083|ANXA1_HUMAN | DITSDTSGDFR | Heavy | 612,3 | 2 | 498,22 | 2 | y9 |
| sp|P04083|ANXA1_HUMAN | DITSDTSGDFR | Heavy | 612,3 | 2 | 330,17 | 1 | b3 |
| sp|Q01469|FABP5_HUMAN | ELGVGIALR | Light | 464,3 | 2 | 685,44 | 1 | y7 |
| sp|Q01469|FABP5_HUMAN | ELGVGIALR | Light | 464,3 | 2 | 628,41 | 1 | y6 |
| sp|Q01469|FABP5_HUMAN | ELGVGIALR | Light | 464,3 | 2 | 529,35 | 1 | y5 |
| sp|Q01469|FABP5_HUMAN | ELGVGIALR | Light | 464,3 | 2 | 472,32 | 1 | y4 |
| sp|Q01469|FABP5_HUMAN | ELGVGIALR | Light | 464,3 | 2 | 359,24 | 1 | y3 |
| sp|Q01469|FABP5_HUMAN | ELGVGIALR | Heavy | 469,3 | 2 | 695,44 | 1 | y7 |
| sp|Q01469|FABP5_HUMAN | ELGVGIALR | Heavy | 469,3 | 2 | 638,42 | 1 | y6 |
| sp|Q01469|FABP5_HUMAN | ELGVGIALR | Heavy | 469,3 | 2 | 539,35 | 1 | y5 |
| sp|Q01469|FABP5_HUMAN | ELGVGIALR | Heavy | 469,3 | 2 | 482,33 | 1 | y4 |
| sp|Q01469|FABP5_HUMAN | ELGVGIALR | Heavy | 469,3 | 2 | 369,25 | 1 | y3 |
| sp|Q01469|FABP5_HUMAN | LVVECVMNNVTCTR | Light | 847,9 | 2 | 995,44 | 1 | y8 |
| sp|Q01469|FABP5_HUMAN | LVVECVMNNVTCTR | Light | 847,9 | 2 | 864,40 | 1 | y7 |
| sp|Q01469|FABP5_HUMAN | LVVECVMNNVTCTR | Light | 847,9 | 2 | 537,24 | 1 | y4 |
| sp|Q01469|FABP5_HUMAN | LVVECVMNNVTCTR | Light | 847,9 | 2 | 436,20 | 1 | y3 |
| sp|Q01469|FABP5_HUMAN | LVVECVMNNVTCTR | Light | 847,9 | 2 | 741,83 | 2 | y12 |
| sp|Q01469|FABP5_HUMAN | LVVECVMNNVTCTR | Heavy | 852,9 | 2 | 1005,45 | 1 | y8 |
| sp|Q01469|FABP5_HUMAN | LVVECVMNNVTCTR | Heavy | 852,9 | 2 | 874,41 | 1 | y7 |
| sp|Q01469|FABP5_HUMAN | LVVECVMNNVTCTR | Heavy | 852,9 | 2 | 547,25 | 1 | y4 |
| sp|Q01469|FABP5_HUMAN | LVVECVMNNVTCTR | Heavy | 852,9 | 2 | 446,21 | 1 | y3 |
| sp|Q01469|FABP5_HUMAN | LVVECVMNNVTCTR | Heavy | 852,9 | 2 | 746,83 | 2 | y12 |
| sp|P08253|MMP2_HUMAN | IDAVYEAPQEEK | Light | 696,3 | 2 | 993,45 | 1 | y8 |
| sp|P08253|MMP2_HUMAN | IDAVYEAPQEEK | Light | 696,3 | 2 | 830,39 | 1 | y7 |
| sp|P08253|MMP2_HUMAN | IDAVYEAPQEEK | Light | 696,3 | 2 | 701,35 | 1 | y6 |
| sp|P08253|MMP2_HUMAN | IDAVYEAPQEEK | Light | 696,3 | 2 | 630,31 | 1 | y5 |
| sp|P08253|MMP2_HUMAN | IDAVYEAPQEEK | Light | 696,3 | 2 | 300,16 | 1 | b3 |
| sp|P08253|MMP2_HUMAN | IDAVYEAPQEEK | Heavy | 700,3 | 2 | 1001,47 | 1 | y8 |
| sp|P08253|MMP2_HUMAN | IDAVYEAPQEEK | Heavy | 700,3 | 2 | 838,40 | 1 | y7 |
| sp|P08253|MMP2_HUMAN | IDAVYEAPQEEK | Heavy | 700,3 | 2 | 709,36 | 1 | y6 |
| sp|P08253|MMP2_HUMAN | IDAVYEAPQEEK | Heavy | 700,3 | 2 | 638,32 | 1 | y5 |
| sp|P08253|MMP2_HUMAN | IDAVYEAPQEEK | Heavy | 700,3 | 2 | 300,16 | 1 | b3 |
| sp|P08253|MMP2_HUMAN | VDAAFNWSK | Light | 519,3 | 2 | 938,44 | 1 | y8 |
| sp|P08253|MMP2_HUMAN | VDAAFNWSK | Light | 519,3 | 2 | 823,41 | 1 | y7 |
| sp|P08253|MMP2_HUMAN | VDAAFNWSK | Light | 519,3 | 2 | 681,34 | 1 | y5 |
| sp|P08253|MMP2_HUMAN | VDAAFNWSK | Light | 519,3 | 2 | 286,14 | 1 | b3 |
| sp|P08253|MMP2_HUMAN | VDAAFNWSK | Heavy | 523,3 | 2 | 946,45 | 1 | y8 |
| sp|P08253|MMP2_HUMAN | VDAAFNWSK | Heavy | 523,3 | 2 | 831,42 | 1 | y7 |
| sp|P08253|MMP2_HUMAN | VDAAFNWSK | Heavy | 523,3 | 2 | 689,35 | 1 | y5 |
| sp|P08253|MMP2_HUMAN | VDAAFNWSK | Heavy | 523,3 | 2 | 286,14 | 1 | b3 |
| sp|P07237|PDIA1_HUMAN | ALAPEYAK | Light | 431,7 | 2 | 678,35 | 1 | y6 |
| sp|P07237|PDIA1_HUMAN | ALAPEYAK | Light | 431,7 | 2 | 607,31 | 1 | y5 |
| sp|P07237|PDIA1_HUMAN | ALAPEYAK | Light | 431,7 | 2 | 339,68 | 2 | y6 |
| sp|P07237|PDIA1_HUMAN | ALAPEYAK | Light | 431,7 | 2 | 304,16 | 2 | y5 |
| sp|P07237|PDIA1_HUMAN | ALAPEYAK | Light | 431,7 | 2 | 256,17 | 1 | b3 |
| sp|P07237|PDIA1_HUMAN | ALAPEYAK | Heavy | 435,7 | 2 | 686,36 | 1 | y6 |
| sp|P07237|PDIA1_HUMAN | ALAPEYAK | Heavy | 435,7 | 2 | 615,32 | 1 | y5 |
| sp|P07237|PDIA1_HUMAN | ALAPEYAK | Heavy | 435,7 | 2 | 343,68 | 2 | y6 |
| sp|P07237|PDIA1_HUMAN | ALAPEYAK | Heavy | 435,7 | 2 | 308,17 | 2 | y5 |
| sp|P07237|PDIA1_HUMAN | ALAPEYAK | Heavy | 435,7 | 2 | 256,17 | 1 | b3 |
| sp|P07237|PDIA1_HUMAN | ILEFFGLK | Light | 483,8 | 2 | 853,48 | 1 | y7 |
| sp|P07237|PDIA1_HUMAN | ILEFFGLK | Light | 483,8 | 2 | 740,40 | 1 | y6 |
| sp|P07237|PDIA1_HUMAN | ILEFFGLK | Light | 483,8 | 2 | 611,36 | 1 | y5 |
| sp|P07237|PDIA1_HUMAN | ILEFFGLK | Light | 483,8 | 2 | 464,29 | 1 | y4 |
| sp|P07237|PDIA1_HUMAN | ILEFFGLK | Light | 483,8 | 2 | 317,22 | 1 | y3 |
| sp|P07237|PDIA1_HUMAN | ILEFFGLK | Heavy | 487,8 | 2 | 861,50 | 1 | y7 |
| sp|P07237|PDIA1_HUMAN | ILEFFGLK | Heavy | 487,8 | 2 | 748,41 | 1 | y6 |
| sp|P07237|PDIA1_HUMAN | ILEFFGLK | Heavy | 487,8 | 2 | 619,37 | 1 | y5 |
| sp|P07237|PDIA1_HUMAN | ILEFFGLK | Heavy | 487,8 | 2 | 472,30 | 1 | y4 |
| sp|P07237|PDIA1_HUMAN | ILEFFGLK | Heavy | 487,8 | 2 | 325,23 | 1 | y3 |
| sp|P12830|CADH1_HUMAN | NLVQIK | Light | 357,7 | 2 | 600,41 | 1 | y5 |
| sp|P12830|CADH1_HUMAN | NLVQIK | Light | 357,7 | 2 | 388,26 | 1 | y3 |
| sp|P12830|CADH1_HUMAN | NLVQIK | Light | 357,7 | 2 | 300,71 | 2 | y5 |
| sp|P12830|CADH1_HUMAN | NLVQIK | Light | 357,7 | 2 | 327,20 | 1 | b3 |
| sp|P12830|CADH1_HUMAN | NLVQIK | Light | 357,7 | 2 | 200,14 | 1 | a2 |
| sp|P12830|CADH1_HUMAN | NLVQIK | Heavy | 361,7 | 2 | 608,42 | 1 | y5 |
| sp|P12830|CADH1_HUMAN | NLVQIK | Heavy | 361,7 | 2 | 396,27 | 1 | y3 |
| sp|P12830|CADH1_HUMAN | NLVQIK | Heavy | 361,7 | 2 | 304,71 | 2 | y5 |
| sp|P12830|CADH1_HUMAN | NLVQIK | Heavy | 361,7 | 2 | 327,20 | 1 | b3 |
| sp|P12830|CADH1_HUMAN | NLVQIK | Heavy | 361,7 | 2 | 200,14 | 1 | a2 |
| sp|P12830|CADH1_HUMAN | VFYSITGQGADTPPVGVFIIER | Light | 789,4 | 3 | 1126,66 | 1 | y10 |
| sp|P12830|CADH1_HUMAN | VFYSITGQGADTPPVGVFIIER | Light | 789,4 | 3 | 1029,61 | 1 | y9 |
| sp|P12830|CADH1_HUMAN | VFYSITGQGADTPPVGVFIIER | Light | 789,4 | 3 | 833,49 | 1 | y7 |
| sp|P12830|CADH1_HUMAN | VFYSITGQGADTPPVGVFIIER | Light | 789,4 | 3 | 677,40 | 1 | y5 |
| sp|P12830|CADH1_HUMAN | VFYSITGQGADTPPVGVFIIER | Light | 789,4 | 3 | 563,83 | 2 | y10 |
| sp|P12830|CADH1_HUMAN | VFYSITGQGADTPPVGVFIIER | Heavy | 792,8 | 3 | 1136,67 | 1 | y10 |
| sp|P12830|CADH1_HUMAN | VFYSITGQGADTPPVGVFIIER | Heavy | 792,8 | 3 | 1039,62 | 1 | y9 |
| sp|P12830|CADH1_HUMAN | VFYSITGQGADTPPVGVFIIER | Heavy | 792,8 | 3 | 843,50 | 1 | y7 |
| sp|P12830|CADH1_HUMAN | VFYSITGQGADTPPVGVFIIER | Heavy | 792,8 | 3 | 687,41 | 1 | y5 |
| sp|P12830|CADH1_HUMAN | VFYSITGQGADTPPVGVFIIER | Heavy | 792,8 | 3 | 568,84 | 2 | y10 |
| sp|P61604|CH10_HUMAN | FLPLFDR | Light | 454,3 | 2 | 647,35 | 1 | y5 |
| sp|P61604|CH10_HUMAN | FLPLFDR | Light | 454,3 | 2 | 550,30 | 1 | y4 |
| sp|P61604|CH10_HUMAN | FLPLFDR | Light | 454,3 | 2 | 437,21 | 1 | y3 |
| sp|P61604|CH10_HUMAN | FLPLFDR | Light | 454,3 | 2 | 380,72 | 2 | y6 |
| sp|P61604|CH10_HUMAN | FLPLFDR | Light | 454,3 | 2 | 324,18 | 2 | y5 |
| sp|P61604|CH10_HUMAN | FLPLFDR | Heavy | 459,3 | 2 | 657,36 | 1 | y5 |
| sp|P61604|CH10_HUMAN | FLPLFDR | Heavy | 459,3 | 2 | 560,31 | 1 | y4 |
| sp|P61604|CH10_HUMAN | FLPLFDR | Heavy | 459,3 | 2 | 447,22 | 1 | y3 |
| sp|P61604|CH10_HUMAN | FLPLFDR | Heavy | 459,3 | 2 | 385,73 | 2 | y6 |
| sp|P61604|CH10_HUMAN | FLPLFDR | Heavy | 459,3 | 2 | 329,18 | 2 | y5 |
| sp|P61604|CH10_HUMAN | VLLPEYGGTK | Light | 538,8 | 2 | 977,53 | 1 | y9 |
| sp|P61604|CH10_HUMAN | VLLPEYGGTK | Light | 538,8 | 2 | 864,45 | 1 | y8 |
| sp|P61604|CH10_HUMAN | VLLPEYGGTK | Light | 538,8 | 2 | 432,73 | 2 | y8 |
| sp|P61604|CH10_HUMAN | VLLPEYGGTK | Light | 538,8 | 2 | 376,18 | 2 | y7 |
| sp|P61604|CH10_HUMAN | VLLPEYGGTK | Light | 538,8 | 2 | 326,24 | 1 | b3 |
| sp|P61604|CH10_HUMAN | VLLPEYGGTK | Heavy | 542,8 | 2 | 985,54 | 1 | y9 |
| sp|P61604|CH10_HUMAN | VLLPEYGGTK | Heavy | 542,8 | 2 | 872,46 | 1 | y8 |
| sp|P61604|CH10_HUMAN | VLLPEYGGTK | Heavy | 542,8 | 2 | 436,73 | 2 | y8 |
| sp|P61604|CH10_HUMAN | VLLPEYGGTK | Heavy | 542,8 | 2 | 380,19 | 2 | y7 |
| sp|P61604|CH10_HUMAN | VLLPEYGGTK | Heavy | 542,8 | 2 | 326,24 | 1 | b3 |
| sp|P30086|PEBP1_HUMAN | VLTPTQVK | Light | 443,3 | 2 | 786,47 | 1 | y7 |
| sp|P30086|PEBP1_HUMAN | VLTPTQVK | Light | 443,3 | 2 | 673,39 | 1 | y6 |
| sp|P30086|PEBP1_HUMAN | VLTPTQVK | Light | 443,3 | 2 | 572,34 | 1 | y5 |
| sp|P30086|PEBP1_HUMAN | VLTPTQVK | Light | 443,3 | 2 | 337,20 | 2 | y6 |
| sp|P30086|PEBP1_HUMAN | VLTPTQVK | Light | 443,3 | 2 | 314,21 | 1 | b3 |
| sp|P30086|PEBP1_HUMAN | VLTPTQVK | Heavy | 447,3 | 2 | 794,49 | 1 | y7 |
| sp|P30086|PEBP1_HUMAN | VLTPTQVK | Heavy | 447,3 | 2 | 681,40 | 1 | y6 |
| sp|P30086|PEBP1_HUMAN | VLTPTQVK | Heavy | 447,3 | 2 | 580,35 | 1 | y5 |
| sp|P30086|PEBP1_HUMAN | VLTPTQVK | Heavy | 447,3 | 2 | 341,20 | 2 | y6 |
| sp|P30086|PEBP1_HUMAN | VLTPTQVK | Heavy | 447,3 | 2 | 314,21 | 1 | b3 |
| sp|P30086|PEBP1_HUMAN | LYEQLSGK | Light | 469,3 | 2 | 824,41 | 1 | y7 |
| sp|P30086|PEBP1_HUMAN | LYEQLSGK | Light | 469,3 | 2 | 661,35 | 1 | y6 |
| sp|P30086|PEBP1_HUMAN | LYEQLSGK | Light | 469,3 | 2 | 532,31 | 1 | y5 |
| sp|P30086|PEBP1_HUMAN | LYEQLSGK | Light | 469,3 | 2 | 291,17 | 1 | y3 |
| sp|P30086|PEBP1_HUMAN | LYEQLSGK | Light | 469,3 | 2 | 412,71 | 2 | y7 |
| sp|P30086|PEBP1_HUMAN | LYEQLSGK | Heavy | 473,3 | 2 | 832,43 | 1 | y7 |
| sp|P30086|PEBP1_HUMAN | LYEQLSGK | Heavy | 473,3 | 2 | 669,37 | 1 | y6 |
| sp|P30086|PEBP1_HUMAN | LYEQLSGK | Heavy | 473,3 | 2 | 540,32 | 1 | y5 |
| sp|P30086|PEBP1_HUMAN | LYEQLSGK | Heavy | 473,3 | 2 | 299,18 | 1 | y3 |
| sp|P30086|PEBP1_HUMAN | LYEQLSGK | Heavy | 473,3 | 2 | 416,72 | 2 | y7 |
| sp|P09466|PAEP_HUMAN | VLVEDDEIMQGFIR | Light | 832,4 | 2 | 1352,62 | 1 | y11 |
| sp|P09466|PAEP_HUMAN | VLVEDDEIMQGFIR | Light | 832,4 | 2 | 751,39 | 1 | y6 |
| sp|P09466|PAEP_HUMAN | VLVEDDEIMQGFIR | Light | 832,4 | 2 | 620,35 | 1 | y5 |
| sp|P09466|PAEP_HUMAN | VLVEDDEIMQGFIR | Light | 832,4 | 2 | 492,29 | 1 | y4 |
| sp|P09466|PAEP_HUMAN | VLVEDDEIMQGFIR | Heavy | 837,4 | 2 | 1362,62 | 1 | y11 |
| sp|P09466|PAEP_HUMAN | VLVEDDEIMQGFIR | Heavy | 837,4 | 2 | 761,40 | 1 | y6 |
| sp|P09466|PAEP_HUMAN | VLVEDDEIMQGFIR | Heavy | 837,4 | 2 | 630,36 | 1 | y5 |
| sp|P09466|PAEP_HUMAN | VLVEDDEIMQGFIR | Heavy | 837,4 | 2 | 502,30 | 1 | y4 |
| sp|P09466|PAEP_HUMAN | VHITSLLPTPEDNLEIVLHR | Light | 766,1 | 3 | 1030,57 | 2 | y18 |
| sp|P09466|PAEP_HUMAN | VHITSLLPTPEDNLEIVLHR | Light | 766,1 | 3 | 974,03 | 2 | y17 |
| sp|P09466|PAEP_HUMAN | VHITSLLPTPEDNLEIVLHR | Light | 766,1 | 3 | 766,91 | 2 | y13 |
| sp|P09466|PAEP_HUMAN | VHITSLLPTPEDNLEIVLHR | Light | 766,1 | 3 | 667,86 | 2 | y11 |
| sp|P09466|PAEP_HUMAN | VHITSLLPTPEDNLEIVLHR | Light | 766,1 | 3 | 350,22 | 1 | b3 |
| sp|P09466|PAEP_HUMAN | VHITSLLPTPEDNLEIVLHR | Heavy | 769,4 | 3 | 1035,58 | 2 | y18 |
| sp|P09466|PAEP_HUMAN | VHITSLLPTPEDNLEIVLHR | Heavy | 769,4 | 3 | 979,04 | 2 | y17 |
| sp|P09466|PAEP_HUMAN | VHITSLLPTPEDNLEIVLHR | Heavy | 769,4 | 3 | 771,91 | 2 | y13 |
| sp|P09466|PAEP_HUMAN | VHITSLLPTPEDNLEIVLHR | Heavy | 769,4 | 3 | 672,86 | 2 | y11 |
| sp|P09466|PAEP_HUMAN | VHITSLLPTPEDNLEIVLHR | Heavy | 769,4 | 3 | 350,22 | 1 | b3 |
| sp|P80188|NGAL_HUMAN | VPLQQNFQDNQFQGK | Light | 597,6 | 3 | 836,39 | 1 | y7 |
| sp|P80188|NGAL_HUMAN | VPLQQNFQDNQFQGK | Light | 597,6 | 3 | 479,26 | 1 | y4 |
| sp|P80188|NGAL_HUMAN | VPLQQNFQDNQFQGK | Light | 597,6 | 3 | 680,37 | 1 | b6 |
| sp|P80188|NGAL_HUMAN | VPLQQNFQDNQFQGK | Light | 597,6 | 3 | 827,44 | 1 | b7 |
| sp|P80188|NGAL_HUMAN | VPLQQNFQDNQFQGK | Light | 597,6 | 3 | 414,22 | 2 | b7 |
| sp|P80188|NGAL_HUMAN | VPLQQNFQDNQFQGK | Heavy | 600,3 | 3 | 844,40 | 1 | y7 |
| sp|P80188|NGAL_HUMAN | VPLQQNFQDNQFQGK | Heavy | 600,3 | 3 | 487,28 | 1 | y4 |
| sp|P80188|NGAL_HUMAN | VPLQQNFQDNQFQGK | Heavy | 600,3 | 3 | 680,37 | 1 | b6 |
| sp|P80188|NGAL_HUMAN | VPLQQNFQDNQFQGK | Heavy | 600,3 | 3 | 827,44 | 1 | b7 |
| sp|P80188|NGAL_HUMAN | VPLQQNFQDNQFQGK | Heavy | 600,3 | 3 | 414,22 | 2 | b7 |
| sp|P80188|NGAL_HUMAN | ELTSELK | Light | 410,2 | 2 | 577,32 | 1 | y5 |
| sp|P80188|NGAL_HUMAN | ELTSELK | Light | 410,2 | 2 | 476,27 | 1 | y4 |
| sp|P80188|NGAL_HUMAN | ELTSELK | Light | 410,2 | 2 | 389,24 | 1 | y3 |
| sp|P80188|NGAL_HUMAN | ELTSELK | Light | 410,2 | 2 | 345,71 | 2 | y6 |
| sp|P80188|NGAL_HUMAN | ELTSELK | Light | 410,2 | 2 | 289,16 | 2 | y5 |
| sp|P80188|NGAL_HUMAN | ELTSELK | Heavy | 414,2 | 2 | 585,33 | 1 | y5 |
| sp|P80188|NGAL_HUMAN | ELTSELK | Heavy | 414,2 | 2 | 484,29 | 1 | y4 |
| sp|P80188|NGAL_HUMAN | ELTSELK | Heavy | 414,2 | 2 | 397,25 | 1 | y3 |
| sp|P80188|NGAL_HUMAN | ELTSELK | Heavy | 414,2 | 2 | 349,71 | 2 | y6 |
| sp|P80188|NGAL_HUMAN | ELTSELK | Heavy | 414,2 | 2 | 293,17 | 2 | y5 |
| sp|Q14508|WFDC2_HUMAN | EGSCPQVNINFPQLGLCR | Light | 697,0 | 3 | 843,45 | 1 | y7 |
| sp|Q14508|WFDC2_HUMAN | EGSCPQVNINFPQLGLCR | Light | 697,0 | 3 | 618,34 | 1 | y5 |
| sp|Q14508|WFDC2_HUMAN | EGSCPQVNINFPQLGLCR | Light | 697,0 | 3 | 505,26 | 1 | y4 |
| sp|Q14508|WFDC2_HUMAN | EGSCPQVNINFPQLGLCR | Light | 697,0 | 3 | 495,76 | 2 | y8 |
| sp|Q14508|WFDC2_HUMAN | EGSCPQVNINFPQLGLCR | Light | 697,0 | 3 | 422,23 | 2 | y7 |
| sp|Q14508|WFDC2_HUMAN | EGSCPQVNINFPQLGLCR | Heavy | 700,3 | 3 | 853,46 | 1 | y7 |
| sp|Q14508|WFDC2_HUMAN | EGSCPQVNINFPQLGLCR | Heavy | 700,3 | 3 | 628,35 | 1 | y5 |
| sp|Q14508|WFDC2_HUMAN | EGSCPQVNINFPQLGLCR | Heavy | 700,3 | 3 | 515,26 | 1 | y4 |
| sp|Q14508|WFDC2_HUMAN | EGSCPQVNINFPQLGLCR | Heavy | 700,3 | 3 | 500,77 | 2 | y8 |
| sp|Q14508|WFDC2_HUMAN | EGSCPQVNINFPQLGLCR | Heavy | 700,3 | 3 | 427,23 | 2 | y7 |
| sp|Q14508|WFDC2_HUMAN | VSCVTPNF | Light | 462,2 | 2 | 478,23 | 1 | y4 |
| sp|Q14508|WFDC2_HUMAN | VSCVTPNF | Light | 462,2 | 2 | 377,18 | 1 | y3 |
| sp|Q14508|WFDC2_HUMAN | VSCVTPNF | Light | 462,2 | 2 | 347,14 | 1 | b3 |
| sp|Q14508|WFDC2_HUMAN | VSCVTPNF | Light | 462,2 | 2 | 446,21 | 1 | b4 |
[truncated: 154,034 more chars]
